# Genome-wide association mapping and genomic prediction for pre‑harvest sprouting resistance, low α-amylase and seed color in Iranian bread wheat

**DOI:** 10.1186/s12870-022-03628-3

**Published:** 2022-06-17

**Authors:** Ehsan Rabieyan, Mohammad Reza Bihamta, Mohsen Esmaeilzadeh Moghaddam, Valiollah Mohammadi, Hadi Alipour

**Affiliations:** 1 Department of Agronomy and Plant Breeding, Faculty of Agricultural Sciences and Engineering, University of Tehran, Karaj, Iran; 2 Cereal Department, Seed and Plant Improvement Institute, AREEO, Karaj, Iran; 3 Department of Plant Production and Genetics, Faculty of Agriculture, Urmia University, Urmia, Iran

**Keywords:** Cereal, Dormancy, GWAS, Pre-harvest sprouting

## Abstract

**Background:**

Pre-harvest sprouting (PHS) refers to a phenomenon, in which the physiologically mature seeds are germinated on the spike before or during the harvesting practice owing to high humidity or prolonged period of rainfall. Pre-harvest sprouting (PHS) remarkably decreases seed quality and yield in wheat; hence it is imperative to uncover genomic regions responsible for PHS tolerance to be used in wheat breeding. A genome-wide association study (GWAS) was carried out using 298 bread wheat landraces and varieties from Iran to dissect the genomic regions of PHS tolerance in a well-irrigated environment. Three different approaches (RRBLUP, GBLUP and BRR) were followed to estimate prediction accuracies in wheat genomic selection.

**Results:**

Genomes B, A, and D harbored the largest number of significant marker pairs (MPs) in both landraces (427,017, 328,006, 92,702 MPs) and varieties (370,359, 266,708, 63,924 MPs), respectively. However, the LD levels were found the opposite, i.e., genomes D, A, and B have the highest LD, respectively. Association mapping by using GLM and MLM models resulted in 572 and 598 marker-trait associations (MTAs) for imputed SNPs (− log10 *P* > 3), respectively. Gene ontology exhibited that the pleitropic MPs located on 1A control seed color, α-Amy activity, and PHS. RRBLUP model indicated genetic effects better than GBLUP and BRR, offering a favorable tool for wheat genomic selection.

**Conclusions:**

Gene ontology exhibited that the pleitropic MPs located on 1A can control seed color, α-Amy activity, and PHS. The verified markers in the current work can provide an opportunity to clone the underlying QTLs/genes, fine mapping, and genome-assisted selection.Our observations uncovered key MTAs related to seed color, α-Amy activity, and PHS that can be exploited in the genome-mediated development of novel varieties in wheat.

**Supplementary Information:**

The online version contains supplementary material available at 10.1186/s12870-022-03628-3.

## Background

Wheat (*Triticum aestivum* L.) has gradually become the global pioneer in supplying human nutrition and calories [1, 2]. The seeds of this crop are prone to sprouting at maturity when reiterated rainfall happens in the time of harvest in the field, leading to a remarkable decrease in flour quality and grain yield [3]. As a result, pre-harvest sprouting (PHS) is known as a detrimental restricting factor in wheat productivity [4]. Given this challenge, genetic improvements in PHS tolerance have become a serious focus of wheat breeders.

PHS tolerance depends on several factors, including i) environmental factors, such as relative humidity and temperature [4]; ii) biophysiological traits, such as germination-inhibitory compounds in the glumes, α-amylase (α-Amy) activity, grain structure and color, phytohormones, and seed dormancy [5]; iii) morphological traits, such as awn and spike structure [6]. Of these factors, grain color is genetically related to PHS tolerance, the red-grained genotypes are more tolerant to PHS than white ones [7]. Genes coding *MYB* transcriptional factors responsible for the flavonoid biosynthesis, *i.e., Tamyb10-1*, have been reported as candidates that determine grain color [8]. *Myb10* confers PHS resistance in wheat, which activates 9-cis-epoxycarotenoid dioxygenase (*NCED*) by biding the secondary wall MYB-responsive element (*SMRE*) to promote ABA biosynthesis in early wheat seed development stages [9–11]. Moreover, experimental evidence highlight seed dormancy is a key genetic component that determines PHS tolerance in wheat genotypes [2].

To date, numerous quantitative trait loci (QTLs) associated with PHS tolerance in wheat have been recognized in previous studies [12]. These works have either assayed PHS tolerance indirectly by germination testing of harvest-ripe grains in a controlled environment [13–15] and/or directly by evaluating spikes in the field or in misting chambers [15, 16]. Most identified genomic segments are mapped on chromosome 4A [17–19], followed by 3A, 3B, and 3D [20]. The PHS tolerance genes located on the chromosomes 3D, 3B, and 3A are known to be pleiotropic or closely linked with red coat controlled by allele R [20]. Several resistant genes such as *MKK3* [21], *Vp1*[22, 23], *PM19* [24, 25], *MFT* [26], *PHS1* [27], *PHS-3D* [9], *ABI5* [28], *FUS3* [29] and *DOG1 *[30], were characterized from wheat for grain dormancy. Recently, Torada et al. [21] cloned *MKK3* as the causal gene for grain dormancy. Further development of functional markers related to PHS tolerance is critical in wheat.

Genome-wide association study (GWAS) is an alternative tool to determine QTLs in natural populations [15]. The establishment of genotyping technologies, from SSRs to SNPs, could facilitate association studies for accurate and efficient exploring of potential loci involved in complex traits, including PHS resistance in wheat [7, 13, 31] and grain-associated traits [32, 33]. However, the molecular mechanisms of PHS resistance remain unclear. Genomic selection (GS) along with GWAS can dramatically accelerate genetic gain in breeding [34, 35]. Several methods, including SNP-BLUP, have been suggested for genomic prediction [36].

In this study, a total of 298 Iranian wheat genotypes were evaluated for genotyping-by-sequencing (GBS)-based GWAS to achieve two objectives: i) uncovering genetic loci associated with PHS resistance; (2) identifying the best model for estimating prediction accuracies in genomic selection.

## Results

### Phenotypic data summary

The results of descriptive statistics of traits related to pre‑harvest sprouting are shown in Table 1. Germination percentage occurred among Iranian wheat cultivars and landraces were ranged from zero to %100. The averages of germination percentage in landraces and cultivars were 71.31% and are 79.67%, respectively, which shows that native populations harbor more value of this trait. Sprouting index, sprouting score, and sprouting spike also confirm the lower pre‑harvest sprouting rate of native populations than cultivated varieties. The α-Amy enzyme activities in native populations and cultivars were 9.38 and 10.76, respectively, which indicates less activity of the enzyme in landraces than that of varieties. Color indices including L, a, and b do not differ significantly between cultivars and landraces.

**Table 1:** Descriptive findings on the studied traits for Iranian landraces and cultivars

Abb	Group	Mean	Minimum	Maximum	Coeff of Variation	Std Error	Std Dev	Skewness	Kurtosis
GP	Landrace	71.31	0.00	100.00	99.45	1.98	28.53	1.24	0.39
	Cultivar	79.67	0.00	100.00	115.74	2.48	23.54	1.84	3.02
	Total	73.84	0.00	100.00	104.53	1.58	27.35	1.39	0.91
SI	Landrace	77.04	0.00	100.00	36.20	1.93	27.88	-1.38	0.84
	Cultivar	83.51	0.00	100.00	26.61	2.34	22.22	-1.95	3.38
	Total	78.99	0.00	100.00	33.46	1.53	26.43	-1.53	1.38
SS	Landrace	6.85	1.00	8.90	29.47	0.14	2.02	-1.49	1.49
	Cultivar	7.45	1.00	9.00	22.21	0.17	1.66	-2.06	4.53
	Total	7.03	1.00	9.00	27.49	0.11	1.93	-1.63	2.09
SSp	Landrace	92.98	0.00	100.00	23.48	1.51	21.83	-3.49	11.24
	Cultivar	96.61	0.00	100.00	15.47	1.58	14.95	-5.54	31.90
	Total	94.08	0.00	100.00	21.31	1.16	20.05	-3.88	14.31
A.amy	Landrace	9.38	0.11	16.09	45.69	0.30	4.29	-0.65	-0.49
	Cultivar	10.76	0.12	16.59	35.10	0.40	3.78	-0.99	0.75
	Total	9.80	0.11	16.59	42.67	0.24	4.18	-0.75	-0.23
L	Landrace	59.90	45.07	72.24	10.44	0.43	6.25	-0.18	-0.83
	Cultivar	60.68	45.01	72.17	9.96	0.64	6.04	-0.11	-0.79
	Total	60.13	45.01	72.24	10.29	0.36	6.19	-0.17	-0.81
a	Landrace	3.40	1.09	6.90	33.95	0.08	1.15	0.22	-0.39
	Cultivar	3.29	1.17	5.48	32.03	0.11	1.05	-0.13	-0.92
	Total	3.36	1.09	6.90	33.39	0.07	1.12	0.15	-0.47
b	Landrace	21.16	15.50	26.76	11.48	0.17	2.43	-0.36	-0.67
	Cultivar	21.73	16.55	25.62	9.29	0.21	2.02	-0.58	-0.06
	Total	21.33	15.50	26.76	10.90	0.13	2.32	-0.45	-0.51
Chroma	Landrace	21.46	15.55	26.96	11.48	0.17	2.46	-0.39	-0.62
	Cultivar	22.00	16.62	25.72	9.05	0.21	1.99	-0.63	0.06
	Total	21.62	15.55	26.96	10.83	0.14	2.34	-0.48	-0.43
Hue	Landrace	1.41	1.28	1.57	3.66	0.00	0.05	-0.06	-0.41
	Cultivar	1.42	1.31	1.52	3.53	0.01	0.05	0.08	-0.84
	Total	1.41	1.28	1.57	3.62	0.00	0.05	-0.03	-0.52
WI	Landrace	54.28	41.87	65.04	8.81	0.33	4.78	-0.19	-0.66
	Cultivar	54.74	42.19	64.03	8.57	0.49	4.69	-0.09	-0.73
	Total	54.42	41.87	65.04	8.73	0.28	4.75	-0.17	-0.68

*GP* Germination Percentage, *SS* Sprouting Score, *SI* Sprouting Index, *SSp* Sprouting Spike, *A.amy* Alpha amylase, *WI* Whiteness Index

**Fig. 1:**
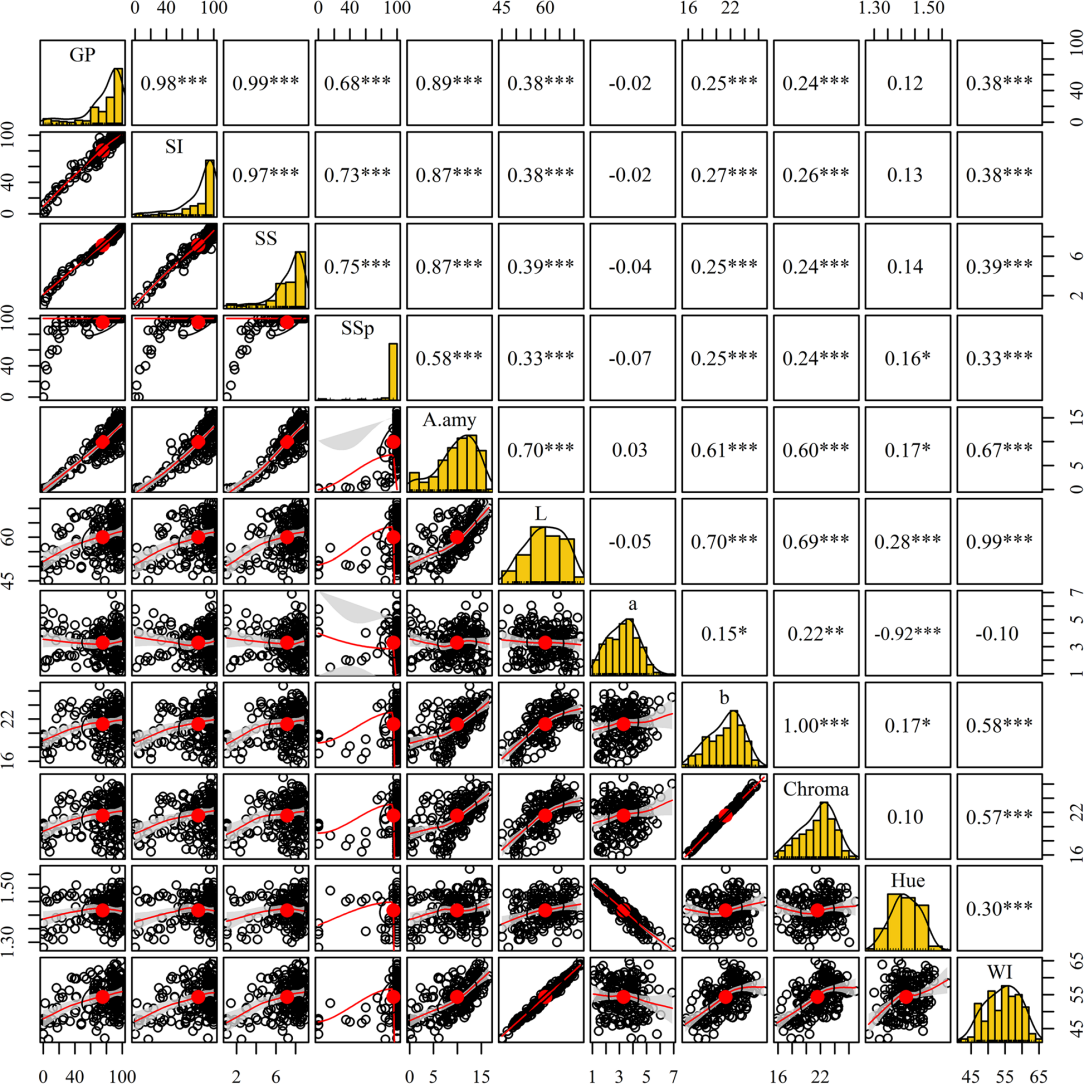
Correlation coefficients between the studied traits for Iranian wheat landraces and cultivars. Abbreviations: GP, Germination Percentage; SS, Sprouting Score; SI, Sprouting Index; SSp, Sprouting spike; A.amy, Alpha amylase; WI, Whiteness Index

From Fig. 1, wheat germination percentage (GP) indicated significant, negative correlations with most seed traits with coefficients ranging from 0.99 to 0.31 (*P* < 0.01). GP had the highest correlation with sprouting score (SS) (*r* = 0.99), followed by sprouting index (SI) (*r* = 0.98), α-Amy (A.amy) (*r* = 0.89), percentage of sprouted spike (SSp) (*r* = 0.68), color index L (*r* = 0.38), brightness index (WI) (*r* = 0.38), color index b, and Chroma (*r* = 0.25 and 0.24).

### Assessment of SNPs

**Fig. 2:**
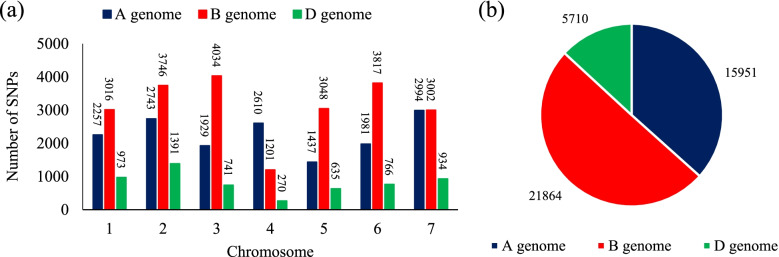
Number of imputed SNPs used in different chromosomes of the wheat genomes (**a**), number of imputed SNPs used in wheat genomes (**b**)

After eight Ion Proton runs, a total of 566,439,207 reads were identified with 458,363,607 (about 81%) high-quality barcoded reads. A total of 133,039 unique SNPs were called after filtering out duplicated reads. After imputation and discarding the SNPs with > 20% missing values, > 10% heterozygosity, and < 5% miner allele frequency, 43,525 SNPs were identified across all 21 wheat chromosomes. Out of them, 15,951, 21,864, and 5,710 SNPs were mapped to A, B, and D genomes, respectively, which included 36.7%, 50.2%, and 13.1% of total SNPs (Fig. 2). The highest and lowest numbers of SNPs were located on 3A (4034 SNPs) and 4D (270 SNPs), respectively.

### Population structure and kinship matrix

**Fig. 3:**
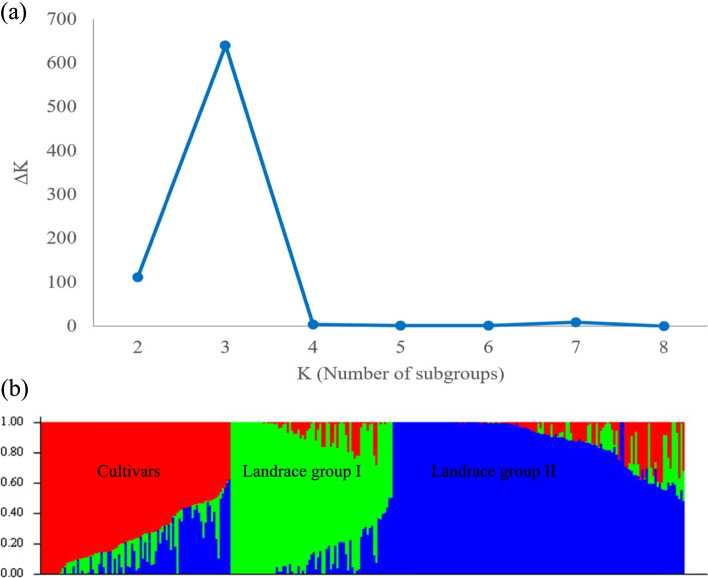
Determination of subpopulations number in wheat genotypes based on ΔK values (**a**), A structure plot of the 298 wheat genotypes and landraces determined by K = 3 (**b**)

**Fig. 4:**
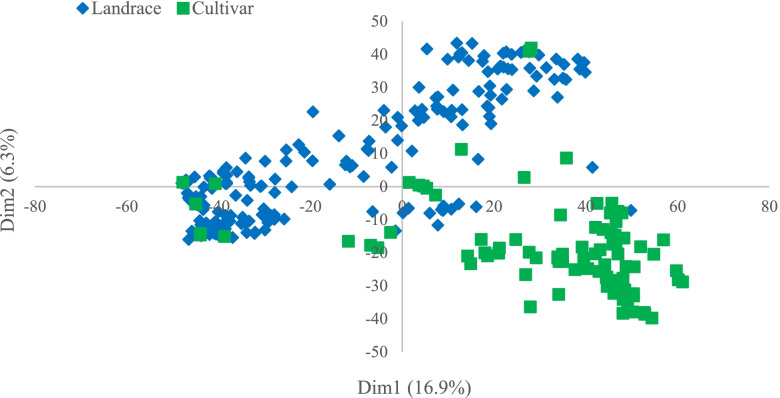
Principle component analysis (PCA) for 298 Iran bread wheat accessions using 43,525 markers

**Fig. 5:**
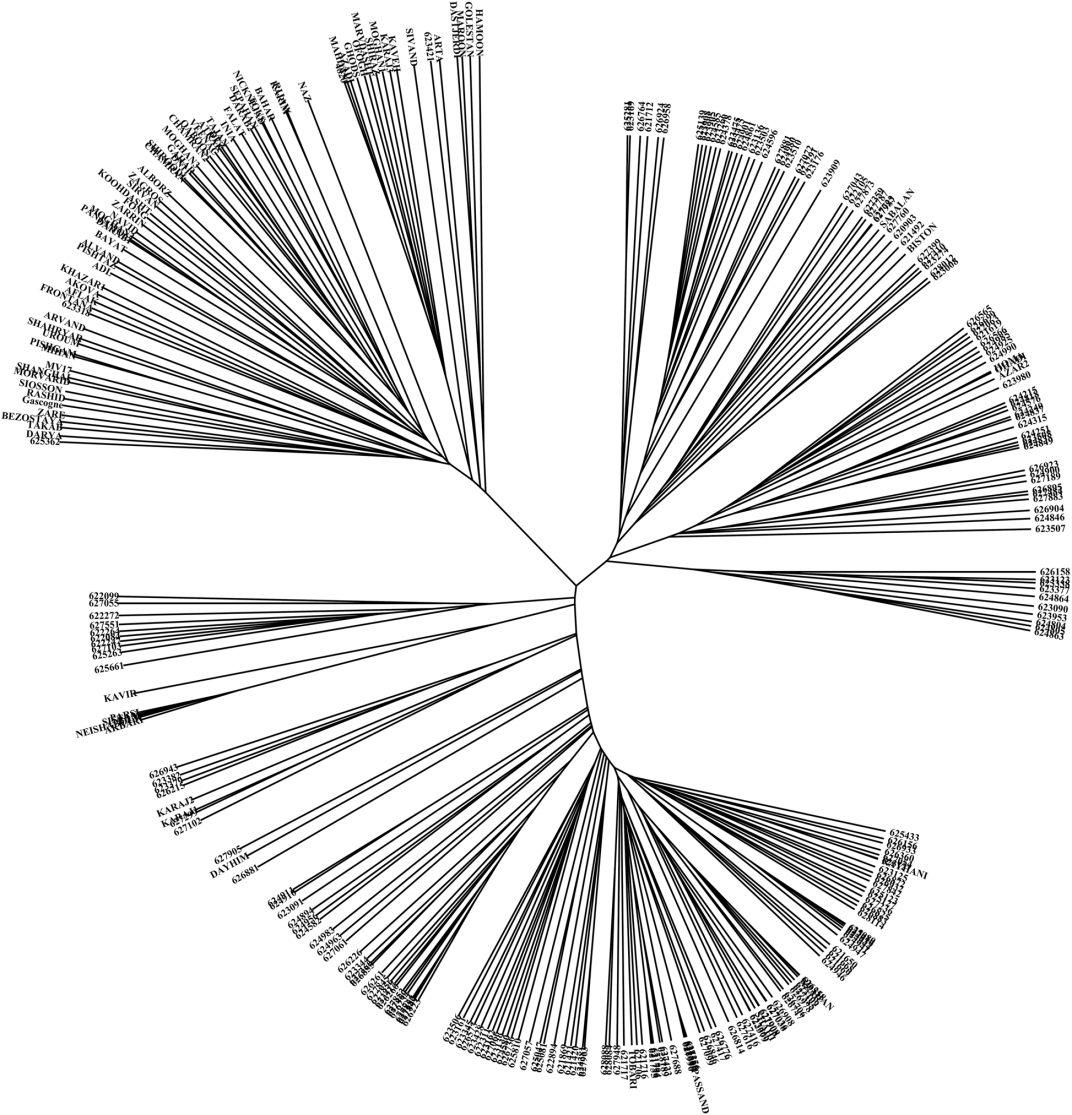
The dendrogram of Neighbor-Joining clustering constructed using 43,525 SNPs and 298 Iranian wheat accessions

In order to determine the appropriate number of subpopulations, the number of clusters was plotted (K) against ΔK. The largest ΔK value was observed at K = 3 suggesting the presence of three subpopulations (Fig. 3a). Using the structure software, the population of 298 accessions was structured into three subpopulations, Sub1, Sub2, and Sub_3 (Fig. 2). Sub_1 contains 113 accessions with 107 landraces and 6 varieties, Sub_2 contains 111 accessions with 97 landraces and 14 varieties; Sub_3 contains 74 studies with 70 varieties and 4 landraces (Fig. 3b). Molecular markers-based PCA showed that the first and second components justified 16.9% and 6.3% of total genetic variance occurred between wheat accessions. Thus, our study can distinguish favorably cultivars and native populations (Fig. 4). As expected, a population structure was identified in the Iranian wheat landraces, with the first five eigenvalues accounting for 30.5% of genetic diversity. From the clustering results, the native populations were divided into two subgroups. Clustering based on the nearest neighbor also indicated that cultivars and landraces were appropriately separated by using the imputed markers (Fig. 5).

### Linkage disequilibrium (LD)

**Fig. 6:**
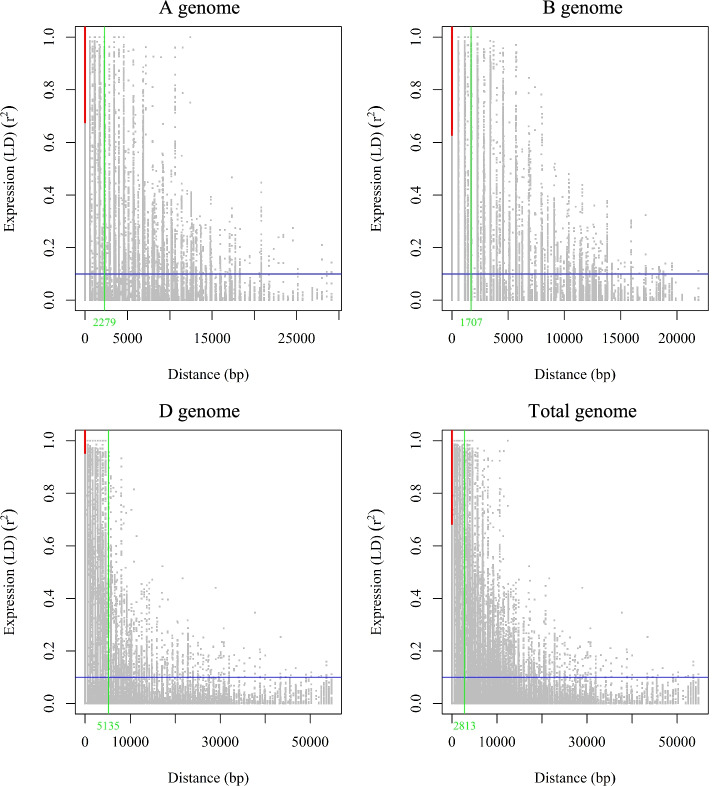
Overview of the linkage disequilibrium (LD) within the whole association panel per genome using imputed SNPs

The levels of LD in genomes A, B, and D were 2279, 1707, and 5135, respectively. This reflects that genomes D, A, and B have the highest LD, respectively (Fig. 6). An analysis on landraces identified a total of 1,867,575 marker pairs with *r*^*2*^ = 0.182, of which 847,725 (45.39%) harbored significant linkages at *P* < 0.001.

Similar to cultivars, marker pairs on chromosome 4A showed the strongest LD (*r*^*2*^ = 0.369). Moreover, most of the significant marker pairs were found at distance of < 10 cM. Genomes D and B possessed the lowest and highest number of marker pairs (92,702 and 427,017), respectively. A total of 1,858,425 marker pairs with *r*^*2*^ = 0.211 were identified in cultivars, of which 700,991 (37.72%) harbored significant linkages at *P* < 0.001. Based on the observations, most of the significant marker pairs were found at distance of < 10 cM. Genomes D and B possessed the lowest and highest number of significant marker pairs (63,924 and 370,359), respectively (Table 2; Fig. 6).

**Table 2:** A summary of LD observed among marker pairs and the number of significant marker pairs per genome and chromosome

Chromosome	Total				Landrace				Cultivar			
	TNSP	r^2^	Dis. (cM)	NSSP	TNSP	r^2^	Dis. (cM)	NSSP	TNSP	r^2^	Dis. (cM)	NSSP
1A	111,575	0.111829	1.333712	49,917 (44.74%)	94,575	0.116906	1.568634	34,895 (36.9%)	85,625	0.148069	1.736676	27,111 (31.66%)
2A	137,150	0.251605	0.856962	79,772 (58.16%)	125,450	0.289098	0.936772	68,972 (54.98%)	119,450	0.288518	0.972951	57,769 (48.36%)
3A	96,450	0.130453	2.27878	44,914 (46.57%)	74,950	0.134097	2.933748	28,787 (38.41%)	85,000	0.15728	2.574908	25,912 (30.48%)
4A	130,500	0.317779	1.378513	79,428 (60.86%)	110,850	0.369392	1.594492	66,016 (59.55%)	116,700	0.36745	1.50704	58,086 (49.77%)
5A	71,850	0.132927	2.005721	32,488 (45.22%)	60,100	0.146486	2.402626	24,483 (40.74%)	60,600	0.166755	2.38547	18,725 (30.9%)
6A	99,050	0.158856	1.296073	52,549 (53.05%)	85,850	0.178539	1.498357	40,739 (47.45%)	86,550	0.178744	1.486057	29,651 (34.26%)
7A	149,700	0.193545	1.164988	78,616 (52.52%)	128,550	0.211862	1.358487	64,114 (49.87%)	129,900	0.232161	1.343972	49,454 (38.07%)
1B	150,800	0.154279	0.932852	80,419 (53.33%)	135,600	0.154625	1.035051	64,442 (47.52%)	132,400	0.20421	1.063407	49,705 (37.54%)
2B	187,300	0.156885	0.764253	102,236 (54.58%)	157,350	0.176011	0.910909	79,057 (50.24%)	166,950	0.19665	0.858127	66,140 (39.62%)
3B	201,700	0.210733	0.771726	119,399 (59.2%)	173,200	0.220043	0.89872	90,266 (52.12%)	177,550	0.243607	0.876084	78,180 (44.03%)
4B	60,050	0.115027	2.20477	23,537 (39.2%)	44,800	0.09777	2.968273	12,423 (27.73%)	52,600	0.142347	2.516753	13,477 (25.62%)
5B	152,400	0.15014	1.292476	80,669 (52.93%)	136,300	0.14202	1.445522	57,252 (42%)	135,650	0.202818	1.431617	55,651 (41.03%)
6B	190,850	0.13708	0.658245	99,314 (52.04%)	167,500	0.135522	0.750676	71,975 (42.97%)	159,700	0.203568	0.787671	66,038 (41.35%)
7B	150,100	0.121987	0.987127	70,107 (46.71%)	127,550	0.12878	1.153868	51,602 (40.46%)	134,150	0.155388	1.102364	41,168 (30.69%)
1D	48,650	0.238268	3.477302	26,009 (53.46%)	42,500	0.226198	3.808863	20,075 (47.24%)	38,350	0.285881	4.409069	16,564 (43.19%)
2D	69,550	0.183692	1.586178	31,547 (45.36%)	55,400	0.163933	1.999469	21,117 (38.12%)	49,600	0.228564	2.23156	16,357 (32.98%)
3D	37,050	0.116765	4.639072	5460 (14.74%)	31,800	0.165445	5.245984	11,619 (36.54%)	26,800	0.137566	6.273779	5458 (20.37%)
4D	13,500	0.122822	9.104484	4560 (33.78%)	11,800	0.130958	10.56137	3577 (30.31%)	11,550	0.154924	10.56621	2312 (20.02%)
5D	31,750	0.130873	6.894582	12,308 (38.77%)	26,250	0.134737	8.311197	9238 (35.19%)	23,700	0.147915	9.317761	5518 (23.28%)
6D	38,300	0.123729	4.134238	15,652 (40.87%)	34,900	0.136001	4.545476	12,619 (36.16%)	29,750	0.137805	5.369092	6852 (23.03%)
7D	46,700	0.150286	4.409549	17,838 (38.2%)	42,300	0.147515	4.882439	14,457 (34.18%)	35,850	0.201644	5.778975	10,863 (30.3%)
A genome	796,275	0.195029	1.397647	417,684 (52.45%)	680,325	0.220024	1.631824	328,006 (48.21%)	683,825	0.232699	1.61945	266,708 (39%)
B genome	1,093,200	0.154972	0.95375	575,681 (52.66%)	942,300	0.1588	1.106081	427,017 (45.32%)	959,000	0.199661	1.084318	370,359 (38.62%)
D genome	285,500	0.162046	4.054108	113,374 (39.71%)	244,950	0.1634	4.684331	92,702 (37.85%)	215,600	0.197637	5.369609	63,924 (29.65%)
Whole genomes	2,174,975	0.170566	1.523235	1,106,739 (50.89%)	1,867,575	0.181706	1.766921	847,725 (45.39%)	1,858,425	0.211583	1.778371	700,991 (37.72%)

*r*^*2*^ average squared allele frequency correlation, *TNSP* Total number of SNP pairs, *NSSP* Number of significant SNP pairs (*P* < 0.001), *Dis* Distance

### MTAs for morphometric seed traits

**Fig. 7:**
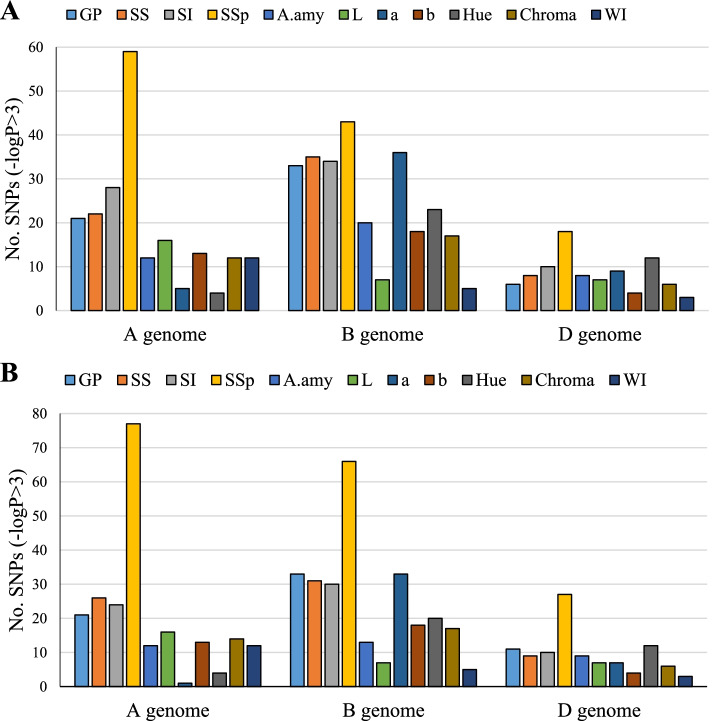
GWAS results for pre‑harvest sprouting traits in Iranian landraces and cultivars. A = GLM, B = MLM

**Fig. 8:**
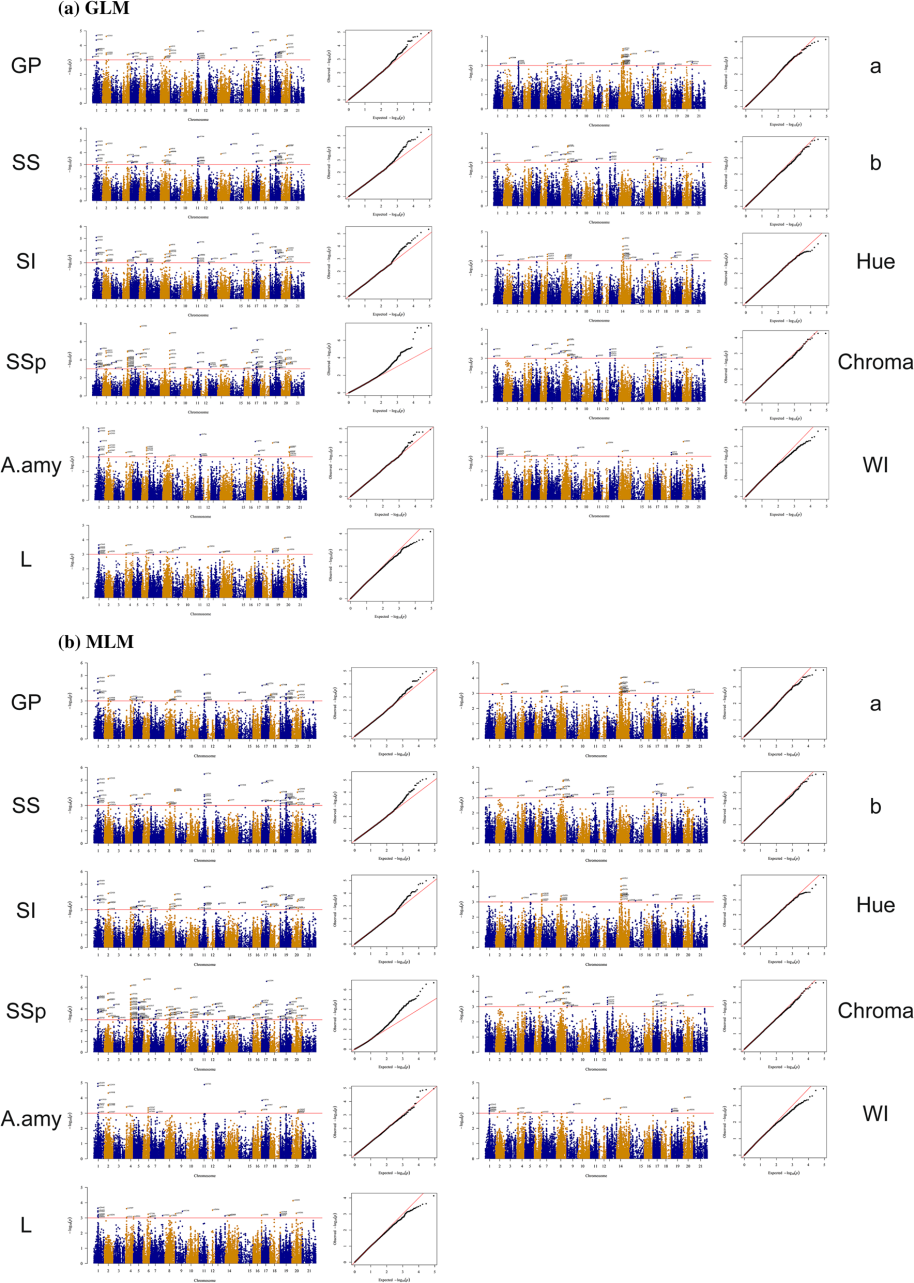
Manhattan and QQ-plots of highly associated haplotypes for GLM (a) and MLM (b) in Iranian wheat landraces and cultivars. X axis represents chromosomes: 1) 1A, 2) 1B, 3) 1D, 4) 2A, 5) 2B, 6) 2D, 7) 3A, 8) 3B, 9) 3D, 10) 4A, 11) 4B, 12) 4D, 13) 5A, 14) 5B, 15) 5D, 16) 6A, 17) 6B, 18) 6D, 19) 7A, 20) 7B, 21)7D. Abbreviations: GP, Germination Percentage; SS, Sprouting Score; SI, Sprouting Index; SSp, Sprouting spike; A.amy, Alpha amylase; WI, Whiteness Index

In total, 566 and 598 significant marker pairs (MTAs) were identified by using GLM and MLM approaches, respectively, for PHS-related traits (–log10 *P* > 3). Of the total number of MTAs in the GLM method, 204, 271, and 97 MTAs were assigned to genomes A, B, and D, respectively. Of 598 MTAs in the MLM method, 220, 273, and 105 MTAs belonged to genomes A, B, and D, respectively. Genome B with 47.9% (GLM) and 45.7% (MLM) harbored the highest significant marker pairs and genome D with 16.1% (GLM) and 17.6% (MLM) possessed the lowest marker pairs, respectively. The number of significant markers for GP, SS, SI, SSp, A.amy, L, a, b, Hue, Chroma, and WI traits using the GLM method were 60, 65, 72, 120, 40, 30, 50, 35, 39, 35, and 20, as well as using the MLM method were 65, 66, 64, 170, 34, 30, 41, 35, 36, 37, and 20, respectively. The highest and lowest numbers of significant marker pairs using GLM and MLM methods were related to SSp (120 and 170 marker pairs) and WI (20 and 20 marker pairs), respectively. The most significant markers for PHS were on genome B, which has a greater effect on seed dormancy when compared to other genomes. However, the seed brightness (L and WI)-associated markers were located on genome A (Fig. 7). Manhattan diagrams for common areas associated with each seed trait are shown in Fig. 8.

### Gene ontology

The markers with the highest significance (P < 0.0001) and pleiotropy were studied in more detail. A total of 41 markers with high significance and pleitropic were identified, most of which were on 1A, 1B, 2A, 3B, 6D, and 7A. The marker pairs located on 1A were found to be able to control seed color, α-Amy activity, and germination percentage. Some of the significant MTAs were responsible for important molecular and biological processes, including protein kinase, G protein-coupled receptor signaling, signal transduction, intracellular transport, oxidoreductase activity, Fe ion binding, oxidation–reduction process, monooxygenase activity, protein binding, regulation of transcription, and double-stranded DNA binding (Table 3).

**Table 3:** Description of expected MTAs using imputed SNPs for seed traits of Iranian wheat accessions in well-watered environment

No	SNP	Sequence	Trait- Index	Chromosome	Position (bp)	Cellular component	Molecular process	Biological process
1	rs10741	TGCAGCAAAAGTCTGAGTTCCTCCTCTCTGAGGTGGGGCTGGAACCAGCATACGTTGCTCATCG	GP, SS, SI, SSp and A.amy	6B	5683	-	double-stranded DNA binding	regulation of transcription, DNA-templated
2	rs12754	TGCAGCAAGTGGCGTATAGGGTTGGGTTTACCTGGTCAGAGTGAAGGTCTGACCGAAACTTTTT	GP, SS, SI and SSp	6B	58,062	-	-	-
3	rs13478	TGCAGCACACACCGTCGGCATGCTACACGTGTCTTCAAGATGAGGATAACCCCGATCACATTCT	b and Chroma	3B	76,224	-	-	-
4	rs15410	TGCAGCACTACCCCCACACCCAAAGCAACTCCGTACTAGCGATGTTGCTTCCCTTTCTCACTAA	GP, SS, SI and A.amy	1A	66,115	-	-	-
5	rs19991	TGCAGCATGGTGACCGCCGAGACCAGCATGGATTTCAGCCAGGAGCTGTTGTCCCTCTTCTTCG	GP, SS, SI and A.amy	1B	47,847	-	-	-
6	rs19993	TGCAGCATGGTGACCGCCGAGACCAGCATGGATTTCAGCCAGGAGCTGTTGTCCCTCTTCTTCG	GP, SS, SI and A.amy	1B	47,847	-	-	-
7	rs22935	TGCAGCCCACCAGGGAACCGTCATCGTCGCCCCGATCGCCACCGTCGCCCCCGAGCTCCACCGA	GP, SS, SI and A.amy	1B	47,847	-	-	-
8	rs23642	TGCAGCCCCGCAGAGGGCACGGAACGCGCGAGCGCGCGCGCACTTCAGCGCAGGCAAACATGGT	SSp, L and WI	1A	44,512	-	-	-
9	rs27586	TGCAGCCTTCCTACAAGGCATCCACGTACCGTCGGCTGTGTCTTCAACCTGACGATTAATGAGA	b, L and Chroma	2D	58,883	-	-	-
10	rs27947	TGCAGCGAAGCATCACAACACTGCAATGGAGCGTCGCCG	GP, SS, SI and A.amy	6D	119,937	-	-	-
11	rs27948	TGCAGCGAAGCATCACAACACTGCAATGGAGCGTCGCCG	GP, SS, SI and A.amy	6D	119,937	-	-	-
12	rs27950	TGCAGCGAAGCATCACGACACTGCAATGGAGCGTCGCCCG	GP, SS, SI and A.amy	6D	119,937	-	-	-
13	rs27951	TGCAGCGAAGCATCACGACACTGCAATGGAGCGTCGCCCG	GP, SS, SI and A.amy	6D	119,937	-	protein binding	-
14	rs3368	TGCAGACACTATGTTTGATTCGCCAGTGGATGCACAACGGACAGGCACCGAGATCGGAAGAGCG	b and Chroma	3B	77,361	-	-	-
15	rs34002	TGCAGCGTGTGGAGATCAAGCGAGAAGCACACCATATACGGCCTGGACACAGTGTACGAATCCC	GP, SS, SI and A.amy	7B	72,800	-	-	-
16	rs35658	TGCAGCTCAACCAAACACAGCCTAAAGCTCATTCTCGCCTAACTACGAGGACAAAATGTTGGCA	L and WI	5B	45,594	-	-	-
17	rs36765	TGCAGCTCCGCTTCGCTCCACCAGGTACGCCTCCCACCTCCACCACCCTCTGGTCGGGAAGTGG	GP, SS, SI and A.amy	7B	72,800	-	-	-
18	rs40099	TGCAGCTGGTTCACTGTAGACCTGCGACTCACGGCAGGAGAGGCGAATCCGAGATCGGAAGAGC	L and WI	7B	12,528	-	-	-
19	rs42907	TGCAGGAATCCCGCTTACTCCATGGATCTCTATTGATGGTGATCAACGGTTTGCTTGGCTGATG	b, L, WI and Chroma	2A	11,390	-	-	-
20	rs43563	TGCAGGACGAGATAAATCGAGTCACCGAAGGCAAACCGACCATCGAGGAAGACGACCTCAGCAG	a and Hue	5B	51,278	-	monooxygenase activity; iron ion binding; oxidoreductase activity	oxidation–reduction process
21	rs44886	TGCAGGAGGTGTGCGACAGCATAACACCGATGCCTAAAGGAAGGTTAAGGACGACCACAACCAC	GP, SS, SI and SSp	5D	7959	-	-	-
22	rs45340	TGCAGGATCTGTACAAGTGGGCTACTCGATGTAATTTTAGCCGAGATCGGAAGAGCGGGATCAC	GP, SS, SI and SSp	3B	113,948	-	-	-
23	rs51766	TGCAGGGTGAAATTAAAGCACTGCTAGCTGCTAGTACGAAACAAGATGCATGTTCAGCGTTAGT	GP, SS, SI, SSp and A.amy	4B	61,749	-	-	-
24	rs53795	TGCAGGTGATCGTGGAGGAGAGCAACACCAACTGCGCCTACTAACCCACCGACGAACCATTAGC	b, L and WI	3B	121,341	-	-	-
25	rs54459	TGCAGGTGGTCGAAGCAGCAGAAGCAGTAGGCGTCGTCGGTGGGGGCAGCAACAGCAGTAGGCG	GP, SS, SI, SSp, A.amy, L and WI	1A	44,512	-	-	-
26	rs54460	TGCAGGTGGTCGAAGCAGCAGAAGCAGTAGGCGTCGTCGGTGGGGGCAGCAACAGCAGTAGGCG	GP, SS, SI, SSp, A.amy, L and WI	1A	44,512	-	-	-
27	rs54593	TGCAGGTGTCGGCGCCCGATGTCATACCGAGGGTTCCTCAACCCTCGCCTGCTATGGAACATCA	GP, SS, SI and SSp	3B	113,379	-	-	-
28	rs6018	TGCAGAGCCGATCCTGCAAAACAAACCCAGCTCTAACACCCTGTGATTTCCCGAGATCGGAAGA	GP, SS, SI and SSp	3B	113,379	-	-	-
29	rs62109	TGCAGTGTCTCCACGCGACCCACCCCGATGCAGGCCGCGTGAAGGCCGCCGTACTGGGACGCCA	GP, SS, SI, SSp, A.amy, L and WI	7B	63,702	-	Intracellular transport	
30	rs63948	TGCAGTTGATGATAGCTAAACCCACGGAACCCTACGTGGATAACCAGCGGCCGCGCTGTACCTT	L, WI, Hue and Chroma	6D	119,937	-	-	-
31	rs8926	TGCAGATGAAACGCCTGCACATGTAACAAATAAACAGACTATTACATGCTCTATCTCTATACGC	GP, SS, SI, SSp, A.amy, L and WI	4A	44,512	-	-	-
32	rs20094	TGCAGCATGTGCCCCGCGGCACGAACAACGAAGCCGACGATATCGCCAAGAGGGTGTCCAGGCG	L and WI	4D	54,756	-	-	-
33	rs21099	TGCAGCCACATCTGCCATTCATTCCGTTCTTGGTGCTGCTTGGGCCATACCTGTTACTCCTTTC	GP, SS, SI and SSp	7A	59,400	Integral component of membrane	-	-
34	rs33980	TGCAGCGTGTCGCATTGTGGACACTACCAGGGAATTTTTCTTATACACATTTTCGGGTGTTACA	GP, SS, SI and SSp	2D	12,505	-	-	-
35	rs41352	TGCAGCTTGCCGCACGAAGAGACCATTGGAGCACCGCAGAGCGAGAGGCGCGGCGCGACGCACA	GP, SS, SI and A.amy	1A	44,512	-	-	-
36	rs45884	TGCAGGCAAGGGATCCCCTCGCAAGATTCAAGAAGCTAGGTGGGCGGCGGCGGATCTTTACCTG	L and WI	2A	92,517	-	Integral component of membrane	Signal transduction; protein kinase C-activating G protein-coupled receptor signaling pathway
37	rs52807	TGCAGGTCAGCAAATGCACGATGGCCGCCGCCACCTGGAGTGCTCTTCTTCAGAGCTTCTCCTC	GP, SS and SI	2B	33,023	-	-	-
38	rs53611	TGCAGGTCTTCGCCCTCGGCCTGAACAAGCGGCTCGCGGACGACGCCGAGATCGGAAGAGCGGG	b and Chroma	2B	15,931	-	-	-
39	rs55558	TGCAGGTTTTGCCTAAGAAAAACTCAGAATTCACTGGAAAAAAATCAGATTGCTGTAAACTGCA	GP, SS and SI	4B	61,749	-	-	-
40	rs57478	TGCAGTATGGCCACATTTGGCAATAGATTTGTTATAAACTTGACAATGGCTAAGAAGCCTCCGT	GP, SS, SI and SSp	2A	59,228	-	-	-
41	rs63525	TGCAGTTCGTAAGCAGAGCGGCAATATACGATATACCACTAGTATACTGTGTCACCACTGGGGT	GP, SS, SI and A.amy	7B	72,800	-	-	-

**Fig. 9:**
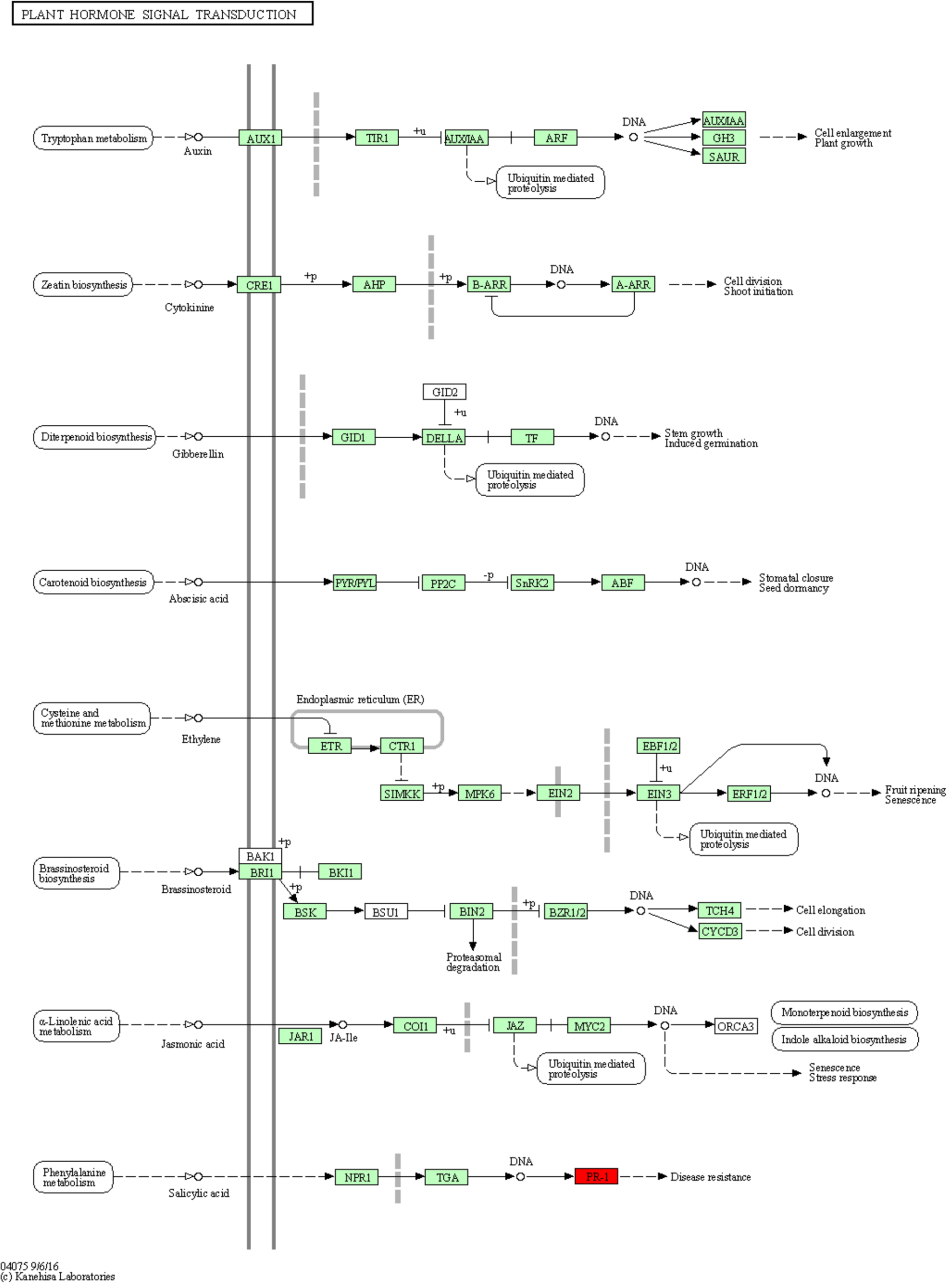
The KEGG pathway of hormone signal transduction (The pathway map without coloring is the original version that is manually drawn by in-house software called KegSketch. The other pathway maps with coloring are all computationally generated as summarized below). • Reference pathway: this is the original version; white boxes are hyperlinked to KO, ENZYME, and REACTION entries in metabolic pathways; they are hyperlinked to KO entries in non-metabolic pathways. • Reference pathway (KO): blue boxes are hyperlinked to KO entries that are selected from the original version. • Reference pathway (EC): blue boxes are hyperlinked to ENZYME entries that are selected from the original version. • Reference pathway (Reaction): blue boxes are hyperlinked to REACTION entries that are selected from the original version. • Organism-specific pathway: green boxes are hyperlinked to GENES entries by converting K numbers (KO identifiers) to gene identifiers in the reference pathway, indicating the presence of genes in the genome and also the completeness of the pathway

Based on the rice reference genome, the following pathways were discovered: hormone signal transduction (Fig. 9), metabolic pathways (Supplementary Fig. 1), MAPK signaling pathway (Supplementary Fig. 2), purine metabolism (Supplementary Fig. 3), spliceosome (Supplementary Fig. 4), and glycolysis/gluconeogenesis (Supplementary Fig. 5) ([37–39], www.kegg.jp/kegg/kegg1.html).

### Genomic prediction

**Fig. 10:**
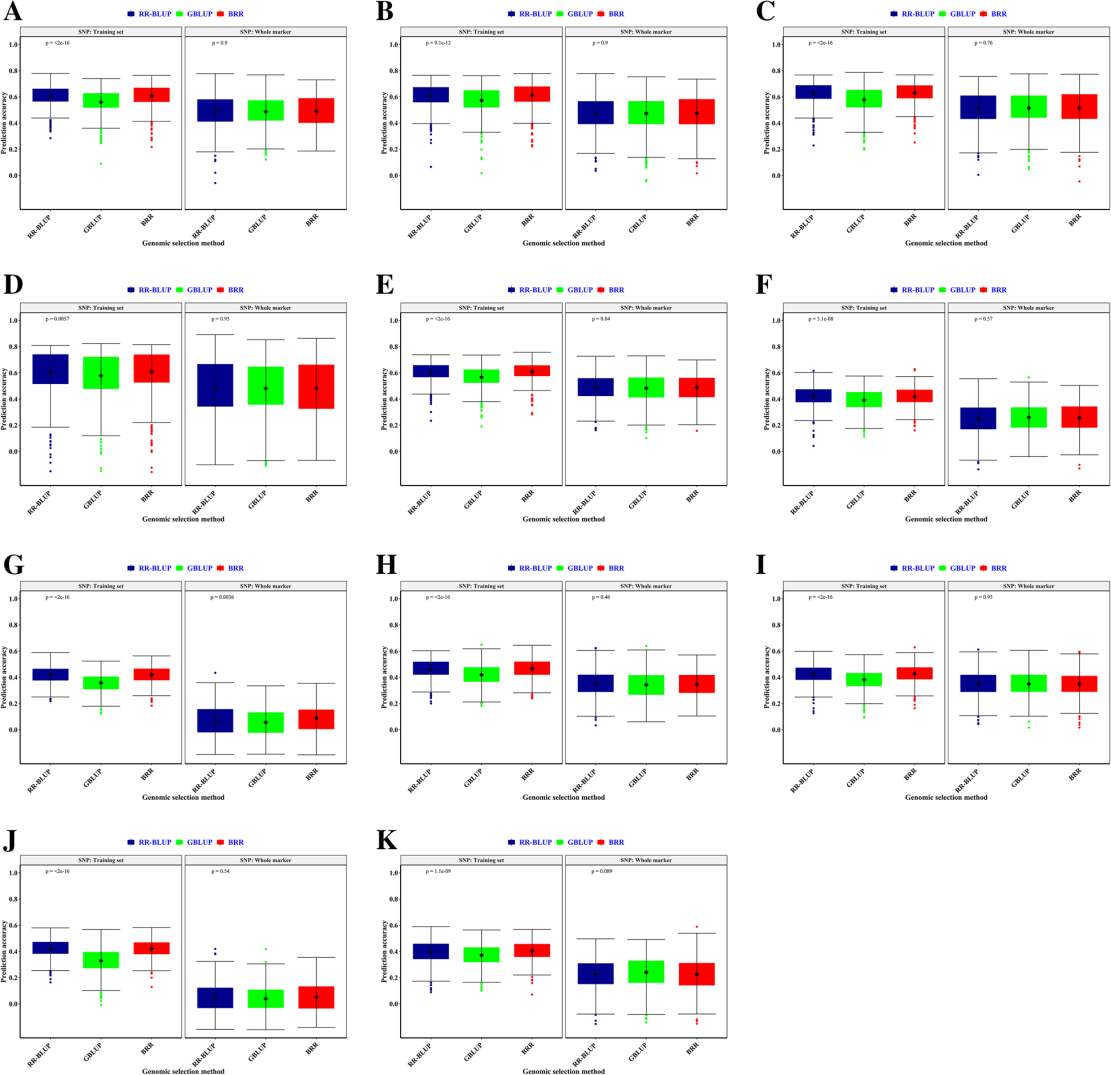
The effect of genomic selection (GS) method on genomic prediction (GP) accuracy for 11 pre-harvest sprouting traits for Iranian landraces and cultivars in the well-watered environment. A-K) The prediction accuracy for RR-BLUP, GBLUP, and BRR-based genomic selection (GS) is demonstrated with blue, green and red colors, respectively. The boxplots show the first, second (median), and third quartile. The middle points indicate a mean of GP accuracies for the trait of interest. **A** = Germination Percentage, **B** = Sprouting Index, **C** = Sprouting Score, **D** = Sprouting Spike, **E** = Alpha amylase, **F** = L, **G** = a, **H** = b, **I** = Chroma, **J** = Hue, **K** = Whiteness Index

BRR, RR-BLUP, and GBLUP models using imputed SNPs exhibited the highest prediction accuracy for phenotypes 6, 3, and 2. The highest prediction accuracy by the GBLUP was achieved for SSp, Hue, and WI; by the RR-BLUP method for SS, SI, A.amy, a, L, and b; as well as by the BRR for GP and L traits (Fig. 10). BRR, RR-BLUP, and GBLUP models using significant SNPs indicated the highest prediction accuracy for phenotypes 2, 7, and 2. The highest prediction accuracy by the GBLUP was achieved for L and WI; by the RR-BLUP method for GP, SS, SI, SSp, Hue, a, and b; as well as by the BRR for A.amy trait. Overall, the RR-BLUP showed higher prediction accuracy and the BRR had a slight difference in accuracy with the RR-BLUP.

## Discussion

PHS tolerance in wheat is a complicated quantitative trait influenced by genetic background and environment [4]. Thus, reliable phenotyping and genotyping for monitoring PHS tolerance can enhance the accuracy of QTL mapping. In this study, a total of 298 Iranian wheat accessions including 208 landraces and 90 cultivars were assembled as a natural population for mapping QTLs related to α-Amy enzyme activity, seed color, PHS using GWAS.

A high level of variation was uncovered in the studied traits for Iranian wheat accessions, suggesting the potential of the GWAS technique for exploring QTLs, as reported by Rahimi et al. [40]. The α-Amy enzyme activity was lower in native populations than that of cultivars. Moreover, the seeds of landraces were exposed to longer dormancy when compared to cultivars. From correlation analysis, the below facts were concluded based on the associations among α-Amy activity, grain color, and pr-harvest sprouting, i) the lower the α-Amy activity, the higher the resistance of accessions to PHS; ii) the darker the seed, the more dormant it is; and iii) the more dormant the seed, the more PHS resistant it is. Similar results were reported by Zhou et al. [3], Zhou et al. [4], and Albrecht et al. [16].

The possibility for false associations can be raised in mapping studies if population structure is not suitably accounted for [41]. Two kinds of kinships lead to a high rate of false positives in GWAS: cryptic relatedness and ancestry difference. Cryptic relatedness appears when some plant accessions are closely related; however, these shared ancestries are undisclosed to breeders [42]. Large populations inevitably consist of accessions having common ancestry from various populations. Ancestry difference also refers to various ancestries among accessions in research [33]. To evaluate the population structure in Iranian wheat accessions, PCA analysis and clustering were performed. Of results, the panel of accessions was stratified into three groups. The selection effects in breeding programs are considered as the reasons for such a genetic separation [43]. Rahimi et al. [40] observed the same grouping on these Iranian wheat accessions. Cultivars made up one group, while landraces made up the other two groups, regardless of their geographic origins. This mixture can be originated from grain exchanges between farmers in different local markets throughout the country [44].

Of the results, the detected SNPs could cover the wheat genome well. The SNPs were higher in genome B and lower in genome D. Therefore, it seems there is a direct correlation between chromosome size and SNP density [45], because of the smaller size of chromosomes B compared to A ones. The higher frequency of SNPs in genome B resulted from the evolutionary processes. This inference was also stated by Alipour et al. [46] and Mourad et al*.* [47].

Genomes D, A, and B have the highest LD, respectively. The strongest LD was recorded between marker pairs on chromosome 4A. The fact that cultivars exhibited higher LD in contrast to landraces, particularly in genome D, is presumably a consequence of selection throughout the time of breeding efforts for PHS-related traits [16]. The differences in LD occurred between genomes and accessions, in addition to the evolutionary processes, indicate the impact of breeding schedules. Similarly, Liu et al. [48] observed that the distance of LD decay in the native populations is less than cultivated varieties in wheat Pakistan/China collections.

Of the results, 1A, 2A, 4A, 1B, 2B, 6B, 4B, 3B, 5B, 7B, 6D, 5D, 4D, and 2D harbor genomic regions controlling PHS-related traits. Genome B possessed the highest number of MTAs, suggesting the potential of this genome in wheat adaptability to PHS. The most significant markers for PHS were on genome B, which has a greater effect on seed dormancy when compared to other genomes. However, the seed brightness-associated markers were located on genome A. These observations are in agreement with previous studies. For instance, Zhu et al. [3] mapped three key loci for PHS tolerance on chromosomes 6BL, 3BS, and 1AL, as well as validated one dCAPS and two CAPS markers for implementation in wheat genomics-based selection.

Genomic regions controlling PHS were detected in most wheat chromosomes in this study. To date, seven PHS QTLs were detected, including *Qphs.ahau-7A.2*, *Qphs.ahau- 6A*, *Qphs.ahau-5D*, *Qphs.ahau-5B.4*, *Qphs.ahau-3B*, *Qphs. ahau-2B.3* and *Qphs.ahau-2A.1* [3]. Our observations showed that the darker the seed, the more dormant it will be and thus the more resistant it will be to PHS. Of justifying the cause, some associations were observed between grain color and PHS tolerance. Zhu et al. [3], for instance, discovered the positive correlations between PHS tolerance and seed color and suggested that this association occurs because the red-colored populations harbor more tolerant *Qphs.ahau-1A* and *Qphs.ahau-3B* alleles. Therefore, the authors stated that wheat seed color may be modulated collectively via *Tamyb10-1* and other QTLs. In this work, MTAs related to grain color were found on 7B, 2A, etc. In this regard, the *Psy1* gene coding phytoene synthase 1, responsible for yellow pigment, is co-segregated with seed brightness on 7B [49]. A major QTL controlling both a* (redness) and L* (brightness) was also reported on 2A [44]. Therefore, it seems that QTLs located on 7B and 2A are involved in wheat seed brightness, and thereby PHS tolerance. In this work, MTAs related to seed dormancy were found in some chromosomes, such as 4A. Similarly, Torada et al. [21] mapped *TaMKK3-A* as a candidate gene for the wheat seed dormancy, namely *Phs1*, on chromosomes 4A. They suggested that a single amino acid substitution in the kinase domain of this protein is related to the length of seed dormancy. From our findings, α-Amy-related genomic regions were found on 6B, 6D, 7B, etc. This is in line with previous studies. Lazarus et al. [50] demonstrated that α-Amy-related genomic regions are multigene families located on the chromosomes 7A, 7B, 7D (α-*Amy2*) and 6A, 6B, 6D (α-*Amy1*).

The flanking sequences of imputed SNPs were searched and aligned versus the RefSeq v1.0 ([51], https://urgi.versailles.inra.fr/blast_iwgsc/). Interestingly, output indicated that most marker pairs are in the protein-coding regions, which control the transcription process. DNA-binding, transcription factor activity, and transmembrane transport are other examples that are likely responsible for PHS tolerance. These findings are similar to the earlier researches [31]. Based on the rice reference genome, the following pathways were discovered: metabolic pathways, hormone signal transduction, MAPK signaling pathway, purine metabolism, spliceosome, and glycolysis/gluconeogenesis. Liu et al. [52] observed that the slowed glycolysis leads to down-regulate glycerate-3-phosphate and inhibits seed germination (i.e., PHS). Torada et al. [21] uncovered a *MKK3* by a map-based approach as a candidate gene for the locus Phs1 on 4A in wheat. Liu et al. [53] revealed that water status changes transcript levels of key genes involved in auxin, JA, and ethylene biosynthesis and their metabolic pathways, suggesting roles in regulating seed dormancy and germination. Nonogaki et al. [54] showed that seed germination and dormancy, the two main factors around PHS, are controled by endogenous hormone balance, especially between GA and ABA, reflecting their vital roles in PHS. Wang et al. [38] indicated that MAPK signaling and hormone signal transduction are associated with PHS. Zhang et al. [55] also highlighted that transcripts of spliceosome-related genes are abundant in the early stage of seed germination, suggesting the role of spliceosome in PHS process.

The highest prediction accuracy by GBLUP was achieved for SSp, Hue, and WI; by RR-BLUP method for SS, SI, A.amy, a, L, and b; as well as by BRR for GP and L traits. Shabannejad et al. [56] revealed BRR and RR-BLUP are superior to other GP models, which are utilized in well-irrigated and rain-fed environments, respectively. Overall, obtaining the highest GP accuracy is depend on the genomic selection method, level of LD, genetic architecture, and genetic variation [57]. In this study, RRBLUP model indicated genetic effects better than GBLUP and BRR, offering a favorable tool for wheat genomic selection. It was reported that high genetic variation would be achieved by the GBLUP if markers were closely associated with the trait of interest and/or plant populations were advanced. RR-BLUP can work well for genetic architecture consisting of numerous loci with small impacts. BRR is similar to RR-BLUP however its shrinkage depends on the size of the studied population [58].

## Conclusion

In the current study, GWAS for PHS in Iranian bread wheat accessions revealed the lowest LD decay distance and the highest number of marker pairs on genome B due to evolutionary events. The loci controlling the traits of interest were mapped on 1A, 2A, 4A, 1B, 2B, 7B, 3B, 5B, 6B, 4B, 6D, 2D, 5D, and 4D. Gene ontology exhibited that the pleitropic MPs located on 1A can control seed color, α-Amy activity, and PHS. The verified markers in the current work can provide an opportunity to clone the underlying QTLs/genes, fine mapping, and genome-assisted selection.

## Material and methods

### Plant material and field trial

To monitor PHS resistance, 208 native landraces and 90 cultivars were cultured in an alpha-lattice with two repeats in three crop seasons (2017–18, 2018–19, and 2019–20) under well-irrigated conditions (Table 4). The sizes of plots were adjusted as 1 m^2^. After physiological maturing, a total of 50 spikes were chosen from each plot and stored at -20 °C. After about a month, the spikes were taken out of the refrigerator and kept at 25 °C for 48 h. From each repeat, 10 healthy spikes were selected and soaked in distilled water for 3 h. Spikes immersed under 100% humidity were placed inside the controlled chambers, in which the steam and mist systems are utilized to spray and to maintain the moisture of the spikes, with a 16 h light/ 8 h dark photoperiod at 25 °C [3]. The authors declare that all study complies with relevant institutional, national, and international guidelines and legislation for plant ethics in the methods section. Samples are provided from the Gene Bank of Agronomy and Plant Breeding Group and these samples are available at USDA and CIMMYT with USDA PI number and CIMMYT number (Supplementary Table 1), respectively. The authors declare that all that permissions or licenses were obtained to collect the wheat plant.

**Table 4:** Climatic data in the studied environments

Year	Month	Max Temperature °C	Min Temperature °C	Average Temperature °C	Total rainfall, mm	Average relative humidity	Sunny hours	Evaporation, mm
2017–2018	November	13.519	4.967	8.929	29.22	64.018	4.810	2.069
	December	9.172	-0.047	4.315	27.59	62.066	6.520	0.270
	January	9.255	-0.416	4.374	4.06	55.780	5.625	0.000
	February	10.356	-0.482	4.721	15.34	55.074	5.874	0.000
	March	15.623	3.985	9.844	38.66	50.191	7.228	0.000
	April	22.903	9.511	16.419	40.11	39.557	9.343	5.892
	May	29.258	14.192	21.833	11.94	35.941	9.233	9.207
	June	34.974	18.595	26.991	0.12	28.390	10.898	12.698
2018–2019	November	14.561	4.104	10.900	0.93	45.810	6.893	3.068
	December	9.242	-0.119	4.671	41.11	60.134	5.065	0.000
	January	8.406	-0.613	3.668	15.04	57.750	6.652	0.000
	February	7.871	-2.254	2.536	27.99	61.429	6.868	0.000
	March	14.216	4.623	9.271	38.44	56.847	5.942	0.179
	April	21.093	9.563	15.110	46.65	49.954	6.587	4.497
	May	29.229	14.261	21.935	22.01	38.722	10.435	7.377
	June	34.159	17.597	26.083	0.00	32.304	12.763	11.676
2019–2020	November	17.080	6.383	11.520	0.63	43.479	6.960	3.189
	December	12.303	1.652	6.671	4.71	50.419	7.226	0.000
	January	9.077	-0.055	4.052	19.84	54.476	6.526	0.000
	February	10.739	2.039	6.464	31.73	64.755	5.829	0.000
	March	20.558	8.377	14.652	14.11	38.952	7.303	0.000
	April	19.983	7.793	13.633	45.81	51.413	7.563	6.714
	May	25.513	12.061	18.432	57.07	54.907	8.287	6.161
	June	33.807	17.347	25.583	7.23	37.492	11.100	11.143

### Pre harvest sprouting and α-Amy activity

After 7 days, PHS resistance was measured (Supplementary Table 2) based on the sprouting score as follows: the wheat spikes were given a score of one to nine, including one (germinated), two (less than 5%), three (5 to 15%), four (16 to 25%), five (26 to 45%), six (46 to 65%), seven (66 to 85%), eight (86 to 95%), and nine (more than 95%). The sprouting Index (SI, Eq. 1) was given a score of zero to five for each spike, in which zero was considered as the non-germinated spikes and five as 100% germinated spikes. The germination percentage (GP) and sprouted spikes (SS) were estimated from Eq. (2) and (3), respectively [59, 60].

1 $$SI(\mathrm{\%})=\left(\sum \left(0-5\right)n*5\right)*100$$

Where n represents the number of clusters,

2 $$GP\left(\mathrm{\%}\right)=(\frac{{n}_{i}}{N})*100$$

Where ni and N are the numbers of germinated and total seeds, respectively,

3 $$SSp(\mathrm{\%})=(\frac{{m}_{i}}{M})*100$$

Where mi is the number of sprouted spikes and M is the total number of spikes.

To estimate α-Amy activity, the spikes of all accessions were taken out of the refrigerator, threshing was conducted by hand to avoid damaging the seed coat or embryos. Therefore, seeds were imbibed in a petri dish for a duration of 24 h at 25 °C and then prepared for enzyme extraction [61]. 0.5 ml of the seed extract (60 mM phosphate buffer (pH 8.6) and 0.5 ml of starch solution were incubated at 37 °C for 30 min. The reaction was ceased by adding 1 ml of hydrochloric acid (0.1 N), and then 1 ml of the iodine reagent was added to the solution. The color absorption was recorded using a plate reader at 620 nm [16].

### Evaluation of seed color with digital images

The digital images of wheat grains in the current work were provided by a camera (Canon SX540 HS) equipped with 800 dpi resolution. The captured images were analyzed and processed via Python 3.7 [62]. For calibration, the regression between L, a, and b indices calculated with the Japanese CR_400 colorimeter and a photo box of 17 standard colors printed on 8 cm squares were used. Chroma saturation or index was calculated by Eq. (4), Hue Angle by Eq. (5), and Whiteness Index by Eq. (6).

4 $$Chroma=\sqrt{{a}^{2}+{b}^{2}}$$

5 $$Hue=\mathrm{arctan}(\frac{b}{a})$$

6 $$WI=100-\sqrt{{(100-L)}^{2}+{a}^{2}+{b}^{2}}$$

Where, L, a, and b are color indices.

### GBS and imputation

The GBS libraries were established and sequenced for the Iranian wheat genotypes following the procedure as explained by Alipour et al. [46]. SNPs were discovered via internal alignments after trimming reads to 64 bp and categorizing them into tags. SNP calling was carried out using the UNEAK GBS pipeline, where SNPs with low allele frequency < 1% and reads with a low-quality score < 15 were removed to keep away from false-positive outputs. The imputation was accomplished according to available allele frequencies in BEAGLE version 3.3.2 [63]. The distance of LD decay was determined by the ggplot2 package in RStudio [64]. The W7984 reference genome was used because it fulfills the highest accuracy of imputation among various wheat reference genomes [65].

### Population structure and kinship matrix

Population structure in the Iranian wheat accessions was assayed via STRUCTURE version 2.3.4. An admixture model was exploited along with a simulation phase consisting of 10,000 steps for K = 1–10. In this study, ΔK was exerted to estimate the most likely number of subpopulations [66]. To measure LD among markers, the expected and observed allele frequencies were introduced into TASSEL. To determine the relationships among the Iranian wheat accessions, a neighbor-joining tree was constructed according to a pairwise distance matrix by TASSEL version 5 [67].

### Genome-wide association study

The general linear model (GLM) and mixed linear model (MLM) approaches were accomplished to obtain the marker effect estimations. The GLM was performed with population structure (Q matrix) integrated as covariate to correct for the effects of population substructure. The MLM was employed with accounting for both population structure and family structure matrix (Kinship) to control both Type I and Type II errors. The association mapping was carried out using GLM and MLM functions of TASSEL5 [65, 68]. To correct for multiple testing, a false discovery rate (FDR) method described was used to declare significant marker-trait associations with relevant grain phenotype descriptor. A Manhattan plot was obtained using the CMplot package to explore associations between genotypes and phenotypes.

### Annotation of genes

Sequences harboring associated SNP markers were exploited for the gene annotation by aligning to the IWGSC-RefSeq V1.0 (IWGSC) using Gramene (http://www.gramene.org/), an integrated database for comparative genomics in plant species. The overlapping genes with the highest blast score were picked out for further analysis. The ensemble-gramene database was utilized to extract the molecular functions and biological processes of genes in the gene ontology. Moreover, the significant SNPs were utilized in the enrichment analysis of gene ontology via KOBAS version 2.0 for testing in the KEGG (https://www.genome.jp/kegg/).

### Genomic prediction strategies

GP was calculated by various approaches, including BRR [69], GBLUP [70], and RR-BLUP [71] based on whole 43,525 marker set and GWAS on the training set. All of the analyses were performed by iPat Tool [72]. The GP accuracy was determined as Pearson’s correlations (r) between GEBVs and BLUPs over the validation and training sets [73].

### Statistical analysis

The descriptive statistics and correlation analysis was conducted by R 4.1 using the ggplot2, dplyr, ggpubr and psych packages to reveal the distribution of wheat traits. To classify wheat genotypes, heatmap analysis was carried out in RStudio.

## Supplementary Information

**Additional file 1: Supplementary Table 1.** Overview on the landraces and cultivars of Iranian wheat studied. **Supplementary Table 2.** Phenotypic (germination, grain color, alpha-amylase) data measured in landraces and cultivars of Iranian wheat studied. **Supplementary Fig 1. **The KEGG pathway of metabolic pathways. **Supplementary Fig 2. **The KEGG pathway of MAPK signaling. **Supplementary Fig 3. **The KEGG pathway of purine metabolism. **Supplementary Fig 4. **The KEGG pathway of spliceosome. **Supplementary Fig 5. **The KEGG pathway of glycolysis/gluconeogenesis.

## Data Availability

The datasets generated and analyzed during the current study are available in the Figshare repository **[**https://doi.org/10.6084/m9.figshare.18774476.v1**].**
