# Supplementary material for: Genome-wide association mapping and genomic prediction for pre‑harvest sprouting resistance, low α-amylase and seed color in Iranian bread wheat
Source: BMC Plant Biol. 2022 Jun 17;22:300. doi: 10.1186/s12870-022-03628-3 (PMC9204952; doi:10.1186/s12870-022-03628-3)
Supplement: Supplementary file 1 — Additional file 1: Supplementary Table 1. Overview on the landraces and cultivars of Iranian wheat studied. Supplementary Table 2. Phenotypic (germination, grain color, alpha-amylase) data measured in landraces and cultivars of Iranian wheat studied. Supplementary Fig 1. The KEGG pathway of metabolic pathways. Supplementary Fig 2. The KEGG pathway of MAPK signaling. Supplementary Fig 3. The KEGG pathway of purine metabolism. Supplementary Fig 4. The KEGG pathway of spliceosome. Supplementary Fig 5. The KEGG pathway of glycolysis/gluconeogenesis. [file 12870_2022_3628_MOESM1_ESM.docx]

**Supplementary Table 1.** Overview on the landraces and cultivars of Iranian wheat studied

| Genetic background: Landraces | | | | | | |
| --- | --- | --- | --- | --- | --- | --- |
| No. | USDA_PI_NO | Region of origin (Province) |  | No. | USDA_PI_NO | Region of origin (Province) |
| 1 | Gilan | 625281 |  | 105 | Tehran | 621669 |
| 2 | Mazandaran | 625362 |  | 106 | Gazvin | 621704 |
| 3 | Khorasan | 625433 |  | 107 | Gazvin | 621706 |
| 4 | Khorasan | 625661 |  | 108 | Gazvin | 621712 |
| 5 | Khorasan | 625810 |  | 109 | Gazvin | 621716 |
| 6 | Kerman | 626156 |  | 110 | Azarbayjan-Gharbi | 620903 |
| 7 | Kerman | 626158 |  | 111 | Hamadan | 621420 |
| 8 | Kerman | 626215 |  | 112 | Hamadan | 621421 |
| 9 | Sistan-Balouchestan | 626223 |  | 113 | Bakhtaran | 621492 |
| 10 | Sistan-Balouchestan | 626226 |  | 114 | Hamadan | 621565 |
| 11 | Sistan-Balouchestan | 626234 |  | 115 | Mazandaran | 622084 |
| 12 | Markazi | 625080 |  | 116 | Gilan | 622098 |
| 13 | Markazi | 625081 |  | 117 | Gilan | 622099 |
| 14 | Markazi | 625123 |  | 118 | Gilan | 622105 |
| 15 | Markazi | 625127 |  | 119 | Mazandaran | 622247 |
| 16 | Markazi | 625139 |  | 120 | Mazandaran | 622264 |
| 17 | Mazandaran | 625263 |  | 121 | Mazandaran | 622272 |
| 18 | Sistan-Balouchestan | 626260 |  | 122 | Khorasan | 622311 |
| 19 | Sistan-Balouchestan | 626261 |  | 123 | Gazvin | 621717 |
| 20 | Esfahan | 626358 |  | 124 | Gazvin | 621735 |
| 21 | Esfahan | 626360 |  | 125 | Gazvin | 621736 |
| 22 | Esfahan | 626565 |  | 126 | Markazi | 621869 |
| 23 | Esfahan | 626566 |  | 127 | Markazi | 621908 |
| 24 | Esfahan | 626573 |  | 128 | Zanjan | 622063 |
| 25 | Ilam | 626699 |  | 129 | Yazd | 623109 |
| 26 | Hamadan | 626706 |  | 130 | Fars | 623123 |
| 27 | Khorasan | 626736 |  | 131 | Fars | 623125 |
| 28 | Yazd | 626747 |  | 132 | Fars | 623127 |
| 29 | Yazd | 626764 |  | 133 | Esfahan | 623008 |
| 30 | Khorasan | 626776 |  | 134 | Esfahan | 623069 |
| 31 | Esfahan | 626814 |  | 135 | Bakhtaran | 623090 |
| 32 | Esfahan | 626825 |  | 136 | Khorasan | 623091 |
| 33 | Yazd | 626846 |  | 137 | Khorasan | 622379 |
| 34 | Markazi | 626855 |  | 138 | Esfahan | 622894 |
| 35 | Fars | 626872 |  | 139 | Azarbayjan-Gharbi | 623266 |
| 36 | Kerman | 626908 |  | 140 | Bakhtaran | 623274 |
| 37 | Gilan | 626923 |  | 141 | Hamadan | 623291 |
| 38 | Gilan | 626924 |  | 142 | Yazd | 623318 |
| 39 | Hormozgan | 626932 |  | 143 | Fars | 623338 |
| 40 | Azarbayjan-Shargi | 626881 |  | 144 | Bakhtaran | 623344 |
| 41 | Fars | 626883 |  | 145 | Kordestan | 623345 |
| 42 | Azarbayjan-Shargi | 626895 |  | 146 | Kerman | 623377 |
| 43 | Azarbayjan-Shargi | 626904 |  | 147 | Kerman | 623379 |
| 44 | Zanjan | 627072 |  | 148 | Azarbayjan-Gharbi | 623136 |
| 45 | Khouzestan | 627099 |  | 149 | Fars | 623139 |
| 46 | Zanjan | 627102 |  | 150 | Azarbayjan-Gharbi | 623161 |
| 47 | Mazandaran | 627103 |  | 151 | Azarbayjan-Gharbi | 623162 |
| 48 | Khorasan | 627189 |  | 152 | Gilan | 623169 |
| 49 | Zanjan | 627055 |  | 153 | Khorasan | 623176 |
| 50 | Gilan | 627057 |  | 154 | Azarbayjan-Gharbi | 623510 |
| 51 | Markazi | 627061 |  | 155 | Bakhtaran | 623905 |
| 52 | Kerman | 627066 |  | 156 | Bakhtaran | 623908 |
| 53 | Hormozgan | 626933 |  | 157 | Bakhtaran | 623909 |
| 54 | Kerman | 626943 |  | 158 | Bakhtaran | 623953 |
| 55 | Azarbayjan-Gharbi | 626958 |  | 159 | Hamadan | 623980 |
| 56 | Esfahan | 626978 |  | 160 | Hamadan | 624215 |
| 57 | Khouzestan | 627036 |  | 161 | Ilam | 624240 |
| 58 | Khouzestan | 627038 |  | 162 | Ilam | 624251 |
| 59 | Azarbayjan-Gharbi | 627043 |  | 163 | Ilam | 623475 |
| 60 | Gilan | 627054 |  | 164 | Ilam | 623503 |
| 61 | Khorasan | 627236 |  | 165 | Bakhtaran | 623506 |
| 62 | Yazd | 627299 |  | 166 | Bakhtaran | 623507 |
| 63 | Hormozgan | 627356 |  | 167 | Bakhtaran | 623508 |
| 64 | Markazi | 627359 |  | 168 | Kerman | 623382 |
| 65 | Kerman | 627360 |  | 169 | Sistan-Balouchestan | 623417 |
| 66 | Bakhtaran | 627385 |  | 170 | Azarbayjan-Shargi | 623421 |
| 67 | Zanjan | 627399 |  | 171 | Azarbayjan-Shargi | 623428 |
| 68 | Azarbayjan-Shargi | 627410 |  | 172 | Ilam | 623473 |
| 69 | Bakhtaran | 627414 |  | 173 | Hamadan | 624596 |
| 70 | Bakhtaran | 627416 |  | 174 | Bakhtaran | 624804 |
| 71 | Bakhtaran | 627417 |  | 175 | Bakhtaran | 624805 |
| 72 | Hamadan | 627423 |  | 176 | Ilam | 624818 |
| 73 | Khorasan | 627460 |  | 177 | Ilam | 624837 |
| 74 | Yazd | 627484 |  | 178 | Ilam | 624838 |
| 75 | Azarbayjan-Shargi | 627787 |  | 179 | Ilam | 624846 |
| 76 | Kerman | 627842 |  | 180 | Ilam | 624849 |
| 77 | Sistan-Balouchestan | 627845 |  | 181 | Ilam | 624861 |
| 78 | Sistan-Balouchestan | 627849 |  | 182 | Kordestan | 624315 |
| 79 | Sistan-Balouchestan | 627852 |  | 183 | Bakhtaran | 624378 |
| 80 | Sistan-Balouchestan | 627853 |  | 184 | Bakhtaran | 624381 |
| 81 | Mazandaran | 627856 |  | 185 | Hamadan | 624576 |
| 82 | Zanjan | 627873 |  | 186 | Hamadan | 624580 |
| 83 | Esfahan | 627688 |  | 187 | Hamadan | 624582 |
| 84 | Yazd | 627723 |  | 188 | Hamadan | 624585 |
| 85 | Azarbayjan-Shargi | 627760 |  | 189 | Tehran | 624944 |
| 86 | Azarbayjan-Shargi | 627551 |  | 190 | Tehran | 624946 |
| 87 | Kordestan | 627587 |  | 191 | Tehran | 624947 |
| 88 | Esfahan | 627616 |  | 192 | Tehran | 624956 |
| 89 | Azarbayjan-Shargi | 627881 |  | 193 | Tehran | 624963 |
| 90 | Azarbayjan-Shargi | 627883 |  | 194 | Gazvin | 624980 |
| 91 | Mazandaran | 627905 |  | 195 | Gazvin | 624983 |
| 92 | Markazi | 627908 |  | 196 | Gazvin | 624985 |
| 93 | Markazi | 627948 |  | 197 | Gazvin | 624990 |
| 94 | Hamadan | 627963 |  | 198 | Markazi | 625047 |
| 95 | Zanjan | 627987 |  | 199 | Ilam | 624863 |
| 96 | Bakhtaran | 627990 |  | 200 | Ilam | 624864 |
| 97 | Bakhtaran | 628012 |  | 201 | Kordestan | 624894 |
| 98 | Mazandaran | 628084 |  | 202 | Kordestan | 624900 |
| 99 | Markazi | 628088 |  | 203 | Kordestan | 624901 |
| 100 | Esfahan | 628114 |  | 204 | Hamadan | 624910 |
| 101 | Ilam | 628189 |  | 205 | Hamadan | 624911 |
| 102 | Kordestan | 621619 |  | 206 | Hamadan | 624925 |
| 103 | Tehran | 621650 |  | 207 | Tehran | 624939 |
| 104 | Tehran | 621668 |  | 208 | Tehran | 624941 |

| Genetic background: Cultivars | | | |
| --- | --- | --- | --- |
| No. | Variety Name | Introduced year | Growth Habit |
| 209 | 4820 | 1951 | Spring |
| 210 | ADL | 1976 | Spring |
| 211 | AFLAK | 2010 | Spring |
| 212 | AKBARI | 2006 | Spring |
| 213 | ALBORZ | 1978 | Spring |
| 214 | ARTA | 2006 | Spring |
| 215 | AZADI | 1979 | Facultative |
| 216 | AZAR | 1957 | Winter |
| 217 | BAYAT | 1976 | Spring |
| 218 | BISTON | 1980 | Spring |
| 219 | CHAMRAN | 1997 | Spring |
| 220 | CHAMRAN-2 | 2013 | Spring |
| 221 | DARAB 1 | 1980 | Spring |
| 222 | DARAB 2 | 1995 | Spring |
| 223 | DASTJERDI | 1960 | Spring |
| 224 | DAYHIM | 1968 | Spring |
| 225 | DN-11 | --- | --- |
| 226 | FALAT | 1990 | Spring |
| 227 | FONG | --- | --- |
| 228 | FONTANA | --- | --- |
| 229 | GAHAR | 1996 | Spring |
| 230 | GHODS | 1988 | Spring |
| 231 | GOLESTAN | 1986 | Spring |
| 232 | HOMA | 2009 | Winter |
| 233 | KARAJ 1 | 1974 | Facultative |
| 234 | KARAJ 2 | 1974 | Winter |
| 235 | KARAJ 3 | 1974 | Winter |
| 236 | KARIM | 2011 | Spring |
| 237 | Gascogne | 1994 | --- |
| 238 | KAVEH | 1980 | Spring |
| 239 | KHAZAR 1 | 1974 | Spring |
| 240 | KOOHDASHT | 2002 | Spring |
| 241 | MAHDAVI | 1995 | Spring |
| 242 | MAROON | 1991 | Spring |
| 243 | MIHAN | 2010 | Winter |
| 244 | MOGHAN 1 | 1974 | Spring |
| 245 | MOGHAN 2 | 1974 | Spring |
| 246 | MOGHAN 3 | 2006 | Spring |
| 247 | MORVARID | 2009 | Spring |
| 248 | MV-17 | 1993 | Winter |
| 249 | NAVID 1990 | 1990 | Facultative |
| 250 | NAZ | 1978 | Spring |
| 251 | NEISHABOUR | 2006 | Spring |
| 252 | NICKNEJAD | 1995 | Spring |
| 253 | OFOGH | 2012 | Spring |
| 254 | OHADI | 2010 | Winter |
| 255 | PANJAMO 62 | 1968 | Spring |
| 256 | PISHGAM | 2008 | Facultative |
| 257 | QABOOS | 2014 | Spring |
| 258 | RAYHANI | 1942 | Spring |
| 259 | RIJAW | 2011 | Facultative |
| 260 | SIOSSON | 1994 | Spring |
| 261 | SHAHI | 1967 | Winter |
| 262 | SHAHPASSAND | 1942 | Winter |
| 263 | SIRVAN | 2012 | Spring |
| 264 | SISTAN | 2006 | Spring |
| 265 | TAK-AB | 2013 | Spring |
| 266 | TOUS | 2002 | Facultative |
| 267 | TOBARI 66 | 1969 | Spring |
| 268 | UROUM | 2009 | Winter |
| 269 | VEE/NAC | 1997 | Spring |
| 270 | ZARE | 2010 | Facultative |
| 271 | ZARRIN | 1995 | Spring |
| 272 | SHANGHAI #7 | --- | Spring |
| 273 | INIA 66 | 1969 | Spring |
| 274 | ARVAND 1 | 1974 | Spring |
| 275 | ROSHAN | 1960 | Spring |
| 276 | RASHID | 1968 | Facultative |
| 277 | SABALAN | 1980 | Spring |
| 278 | DARYA | 2006 | Spring |
| 279 | ATRAK | 1995 | Spring |
| 280 | BAHAR | 2007 | Spring |
| 281 | SEPAHAN | 2006 | Spring |
| 282 | BAM | 2006 | Spring |
| 283 | SHIRAZ | 2002 | Spring |
| 284 | PISHTAZ | 2002 | Spring |
| 285 | HAMOON | 2002 | Spring |
| 286 | DEZ | 2002 | Spring |
| 287 | SHIROODI | 1997 | Spring |
| 288 | MARVDASHT | 1999 | Spring |
| 289 | ZAGROS | 1996 | Spring |
| 290 | TAJAN | 1995 | Spring |
| 291 | ALVAND | 1995 | Facultative |
| 292 | KAVIR | 1997 | Spring |
| 293 | PARSI | 2009 | Spring |
| 294 | SIVAND | 2009 | Spring |
| 295 | BEZOSTAYA | 1969 | Winter |
| 296 | AKOVA | 1958 | Winter |
| 297 | SHAHRYAR | 2002 | Winter |
| 298 | AZAR 2 | 1997 | Winter |

**Supplementary Table 2.** Phenotypic (germination, grain color, alpha-amylase) data measured in landraces and cultivars of Iranian wheat studied

| **Year** | **Rep** | **Block** | **Genotype-code** | **SSp** | **GP** | **SS** | **SI** | **A.amy** | **L** | **a** | **b** | **Chroma** | **Hue** | **WI** |
| --- | --- | --- | --- | --- | --- | --- | --- | --- | --- | --- | --- | --- | --- | --- |
| 2018-2019 | 1 | 1 | 627036 | 100 | 69.08 | 7.00 | 84 | 13.5 | 62.64 | 3.48 | 24.02 | 24.27 | 1.43 | 55.45 |
| 2018-2019 | 1 | 1 | 626156 | 100 | 32.98 | 4.80 | 52 | 7.93 | 55.02 | 3.61 | 20.99 | 21.30 | 1.40 | 50.23 |
| 2018-2019 | 1 | 1 | ROSHAN | 100 | 92.12 | 8.20 | 96 | 11.05 | 55.34 | 4.44 | 21.18 | 21.64 | 1.36 | 50.37 |
| 2018-2019 | 1 | 1 | 627852 | 100 | 79.33 | 7.20 | 76 | 13.46 | 62.78 | 3.71 | 23.21 | 23.50 | 1.41 | 55.98 |
| 2018-2019 | 1 | 1 | 628114 | 100 | 75.81 | 7.20 | 96 | 12.6 | 62.24 | 3.36 | 21.94 | 22.20 | 1.42 | 56.20 |
| 2018-2019 | 1 | 1 | 624985 | 100 | 52.21 | 5.80 | 60 | 9.39 | 53.56 | 5.41 | 22.38 | 23.02 | 1.33 | 48.17 |
| 2018-2019 | 1 | 1 | 624837 | 100 | 51.75 | 5.80 | 48 | 12.65 | 69.36 | 3.38 | 24.14 | 24.38 | 1.43 | 60.85 |
| 2018-2019 | 1 | 1 | TOUS | 100 | 25.37 | 4.20 | 52 | 6.9 | 52.97 | 4.22 | 20.64 | 21.07 | 1.37 | 48.47 |
| 2018-2019 | 1 | 1 | 623473 | 100 | 48.68 | 5.40 | 64 | 8.56 | 57.1 | 4.12 | 20.9 | 21.30 | 1.38 | 52.10 |
| 2018-2019 | 1 | 2 | MOGHAN2 | 100 | 60.34 | 6.20 | 76 | 12.89 | 63.97 | 4.02 | 23.64 | 23.98 | 1.40 | 56.72 |
| 2018-2019 | 1 | 2 | 627359 | 100 | 76.3 | 7.00 | 84 | 10.81 | 57.66 | 3.96 | 20.79 | 21.16 | 1.38 | 52.67 |
| 2018-2019 | 1 | 2 | 627038 | 100 | 91.87 | 8.00 | 100 | 12.13 | 58.63 | 5.19 | 21.48 | 22.10 | 1.33 | 53.10 |
| 2018-2019 | 1 | 2 | 621704 | 100 | 85.45 | 7.40 | 100 | 5.28 | 46.88 | 1.46 | 16.71 | 16.77 | 1.48 | 44.29 |
| 2018-2019 | 1 | 2 | 626566 | 100 | 31.95 | 4.60 | 40 | 6.83 | 51.32 | 4.35 | 20.21 | 20.67 | 1.36 | 47.11 |
| 2018-2019 | 1 | 2 | SHAHPASSAND | 100 | 83.96 | 7.80 | 100 | 15.42 | 67.83 | 3.84 | 23.29 | 23.60 | 1.41 | 60.10 |
| 2018-2019 | 1 | 2 | 623508 | 100 | 78.3 | 7.20 | 88 | 11.9 | 63.39 | 4.27 | 24.1 | 24.48 | 1.40 | 55.96 |
| 2018-2019 | 1 | 2 | 621735 | 100 | 100 | 9.00 | 100 | 9.06 | 51.72 | 1.95 | 19.71 | 19.81 | 1.47 | 47.82 |
| 2018-2019 | 1 | 2 | 623338 | 100 | 100 | 9.00 | 100 | 14.07 | 59.42 | 3.06 | 24.16 | 24.35 | 1.44 | 52.67 |
| 2018-2019 | 1 | 2 | 621668 | 100 | 99.02 | 8.80 | 100 | 8.36 | 51.93 | 3.18 | 17.36 | 17.65 | 1.39 | 48.79 |
| 2018-2019 | 1 | 3 | 621421 | 100 | 67.38 | 6.60 | 84 | 7.95 | 56.28 | 1.46 | 16.52 | 16.58 | 1.48 | 53.24 |
| 2018-2019 | 1 | 3 | 624240 | 100 | 66.67 | 6.60 | 76 | 10.85 | 57.87 | 3.97 | 21 | 21.37 | 1.38 | 52.76 |
| 2018-2019 | 1 | 3 | 623344 | 100 | 64.21 | 6.60 | 88 | 12.72 | 62.59 | 3.74 | 23.86 | 24.15 | 1.42 | 55.47 |
| 2018-2019 | 1 | 3 | 624941 | 0 | 0 | 1.00 | 20 | 0.16 | 50.44 | 4.07 | 17.1 | 17.58 | 1.34 | 47.42 |
| 2018-2019 | 1 | 3 | 621716 | 100 | 87.63 | 7.80 | 96 | 14.02 | 64.06 | 6.9 | 25.11 | 26.04 | 1.30 | 55.62 |
| 2018-2019 | 1 | 3 | 627236 | 100 | 98.3 | 8.80 | 100 | 14.21 | 67.85 | 4.35 | 22.16 | 22.58 | 1.38 | 60.71 |
| 2018-2019 | 1 | 3 | 624911 | 100 | 87.01 | 8.00 | 92 | 12.73 | 66.65 | 2.43 | 21.3 | 21.44 | 1.46 | 60.35 |
| 2018-2019 | 1 | 4 | OHADI | 100 | 82.54 | 7.60 | 96 | 14.22 | 71.67 | 4.72 | 22.74 | 23.22 | 1.37 | 63.37 |
| 2018-2019 | 1 | 4 | 624990 | 100 | 30.17 | 4.60 | 52 | 9.85 | 65.23 | 4.05 | 23.28 | 23.63 | 1.40 | 57.96 |
| 2018-2019 | 1 | 4 | 627484 | 100 | 100 | 9.00 | 100 | 10.58 | 61.51 | 6.2 | 21.24 | 22.13 | 1.29 | 55.60 |
| 2018-2019 | 1 | 4 | 627688 | 100 | 100 | 9.00 | 100 | 7.66 | 50.55 | 2.46 | 15.71 | 15.90 | 1.42 | 48.06 |
| 2018-2019 | 1 | 4 | 627416 | 100 | 100 | 9.00 | 100 | 14.82 | 72.24 | 3.72 | 20.93 | 21.26 | 1.39 | 65.04 |
| 2018-2019 | 1 | 4 | 625661 | 100 | 94.76 | 8.20 | 100 | 8.08 | 49.82 | 1.6 | 16.99 | 17.07 | 1.48 | 47.00 |
| 2018-2019 | 1 | 4 | 625081 | 100 | 91.75 | 8.20 | 96 | 10.71 | 59.57 | 4.33 | 22.68 | 23.09 | 1.38 | 53.44 |
| 2018-2019 | 1 | 4 | 627061 | 100 | 94.95 | 8.40 | 100 | 12.64 | 61.18 | 3.65 | 21.6 | 21.91 | 1.40 | 55.43 |
| 2018-2019 | 1 | 4 | NEISHABOUR | 100 | 66.67 | 6.60 | 72 | 13.69 | 67.71 | 4.32 | 22.81 | 23.22 | 1.38 | 60.23 |
| 2018-2019 | 1 | 4 | 624315 | 100 | 74.15 | 7.00 | 72 | 14.3 | 69.3 | 3.9 | 23.98 | 24.30 | 1.41 | 60.85 |
| 2018-2019 | 1 | 5 | 627103 | 100 | 8.79 | 3.00 | 36 | 0.65 | 49.29 | 1.98 | 16.33 | 16.45 | 1.45 | 46.69 |
| 2018-2019 | 1 | 5 | 623475 | 100 | 66.17 | 6.60 | 80 | 9.28 | 57.72 | 3.38 | 19.36 | 19.65 | 1.40 | 53.38 |
| 2018-2019 | 1 | 5 | 624596 | 100 | 63.11 | 6.00 | 84 | 9.69 | 58.77 | 4.27 | 21.76 | 22.17 | 1.38 | 53.19 |
| 2018-2019 | 1 | 5 | AKBARI | 100 | 70.12 | 6.80 | 76 | 8.6 | 59.12 | 3.66 | 20.1 | 20.43 | 1.39 | 54.30 |
| 2018-2019 | 1 | 5 | 623506 | 100 | 94.69 | 8.40 | 100 | 11.96 | 69.68 | 5.66 | 21.72 | 22.45 | 1.32 | 62.28 |
| 2018-2019 | 1 | 5 | 623510 | 100 | 89.91 | 8.40 | 96 | 8.79 | 51.58 | 4.8 | 19.93 | 20.50 | 1.33 | 47.42 |
| 2018-2019 | 1 | 5 | 627845 | 100 | 97.01 | 8.80 | 96 | 10.88 | 57.93 | 3.94 | 19.74 | 20.13 | 1.37 | 53.36 |
| 2018-2019 | 1 | 6 | 627856 | 80 | 4.03 | 2.20 | 28 | 0.24 | 50.27 | 1.95 | 18.16 | 18.26 | 1.46 | 47.02 |
| 2018-2019 | 1 | 6 | MAHDAVI | 100 | 97.39 | 8.80 | 100 | 15.47 | 66.98 | 3.86 | 24.22 | 24.53 | 1.41 | 58.87 |
| 2018-2019 | 1 | 6 | 627360 | 100 | 97.84 | 8.80 | 100 | 14.92 | 66.77 | 3.34 | 23.13 | 23.37 | 1.43 | 59.38 |
| 2018-2019 | 1 | 6 | 627883 | 100 | 90.32 | 8.20 | 92 | 13.45 | 69.25 | 3.94 | 22.62 | 22.96 | 1.40 | 61.62 |
| 2018-2019 | 1 | 6 | SHIROODI | 100 | 96.87 | 8.60 | 96 | 15.07 | 67.75 | 1.17 | 22.31 | 22.34 | 1.52 | 60.77 |
| 2018-2019 | 1 | 6 | 623379 | 100 | 93.1 | 8.40 | 92 | 10.45 | 56.13 | 5.24 | 23.78 | 24.35 | 1.35 | 49.83 |
| 2018-2019 | 1 | 6 | 624910 | 100 | 83.1 | 7.20 | 92 | 13.84 | 64.64 | 5.16 | 24.42 | 24.96 | 1.36 | 56.72 |
| 2018-2019 | 1 | 6 | MOGHAN3 | 100 | 95.74 | 8.40 | 100 | 15.35 | 63.6 | 4.07 | 25.35 | 25.67 | 1.41 | 55.46 |
| 2018-2019 | 1 | 6 | DASTJERDI | 100 | 100 | 9.00 | 96 | 4.25 | 68.49 | 3.45 | 22.57 | 22.83 | 1.42 | 61.09 |
| 2018-2019 | 1 | 7 | TAKAB | 80 | 15.1 | 3.00 | 32 | 0.98 | 50.75 | 4.02 | 17.23 | 17.69 | 1.34 | 47.67 |
| 2018-2019 | 1 | 7 | 623908 | 100 | 100 | 9.00 | 84 | 11.25 | 61.48 | 3.28 | 19.12 | 19.40 | 1.40 | 56.87 |
| 2018-2019 | 1 | 7 | RASHID | 100 | 97.9 | 8.80 | 96 | 14.77 | 70.59 | 3.72 | 21.56 | 21.88 | 1.40 | 63.34 |
| 2018-2019 | 1 | 7 | 624900 | 100 | 77.9 | 7.00 | 88 | 13.89 | 67.79 | 5.57 | 24.57 | 25.19 | 1.35 | 59.11 |
| 2018-2019 | 1 | 7 | 627054 | 100 | 83.44 | 7.40 | 96 | 11.76 | 55.21 | 6.04 | 23.4 | 24.17 | 1.32 | 49.11 |
| 2018-2019 | 1 | 7 | 626223 | 100 | 98.67 | 8.80 | 100 | 14 | 64.03 | 3.63 | 23.38 | 23.66 | 1.42 | 56.95 |
| 2018-2019 | 1 | 7 | 627189 | 100 | 98.48 | 8.80 | 100 | 14.24 | 67.55 | 2.88 | 21.68 | 21.87 | 1.44 | 60.87 |
| 2018-2019 | 1 | 7 | 627423 | 100 | 100 | 9.00 | 80 | 15.46 | 64.72 | 5.91 | 24.64 | 25.34 | 1.34 | 56.56 |
| 2018-2019 | 1 | 7 | ZARE | 100 | 100 | 9.00 | 100 | 12.32 | 61.5 | 4.22 | 21.03 | 21.45 | 1.37 | 55.93 |
| 2018-2019 | 1 | 8 | 627410 | 100 | 100 | 9.00 | 100 | 8.51 | 53.72 | 4.58 | 18.28 | 18.85 | 1.33 | 50.03 |
| 2018-2019 | 1 | 8 | DN11 | 100 | 100 | 9.00 | 100 | 13.98 | 66.92 | 3.28 | 23.46 | 23.69 | 1.43 | 59.31 |
| 2018-2019 | 1 | 8 | 625080 | 100 | 98.78 | 8.80 | 96 | 12.99 | 61.11 | 4.59 | 22.49 | 22.95 | 1.37 | 54.84 |
| 2018-2019 | 1 | 8 | HOMA | 100 | 90.85 | 8.00 | 80 | 13.66 | 70.59 | 3.96 | 23.13 | 23.47 | 1.40 | 62.38 |
| 2018-2019 | 1 | 8 | 627842 | 100 | 100 | 9.00 | 100 | 12.19 | 62.22 | 3.89 | 20.96 | 21.32 | 1.39 | 56.62 |
| 2018-2019 | 1 | 8 | 627043 | 100 | 70.83 | 7.00 | 88 | 7.14 | 59.55 | 2.01 | 18.58 | 18.69 | 1.46 | 55.44 |
| 2018-2019 | 1 | 8 | 624956 | 100 | 93.62 | 8.20 | 92 | 14.4 | 66.72 | 5.46 | 23.11 | 23.75 | 1.34 | 59.12 |
| 2018-2019 | 1 | 8 | 626261 | 100 | 96.21 | 8.60 | 100 | 12.62 | 56.86 | 3.84 | 22.72 | 23.04 | 1.40 | 51.09 |
| 2018-2019 | 1 | 8 | 628084 | 100 | 94.22 | 8.40 | 100 | 7.99 | 49.1 | 3.15 | 16.99 | 17.28 | 1.39 | 46.25 |
| 2018-2019 | 1 | 8 | VEE/NAC | 100 | 99.11 | 8.80 | 100 | 11.46 | 53.61 | 4.86 | 22.31 | 22.83 | 1.36 | 48.30 |
| 2018-2019 | 1 | 9 | 625047 | 100 | 79.54 | 7.40 | 92 | 13.82 | 66.16 | 3.88 | 22.62 | 22.95 | 1.40 | 59.11 |
| 2018-2019 | 1 | 9 | 626158 | 100 | 71.1 | 6.80 | 84 | 8.51 | 51.84 | 1.84 | 18.36 | 18.45 | 1.47 | 48.43 |
| 2018-2019 | 1 | 9 | 624925 | 100 | 75 | 7.00 | 88 | 14.48 | 66.93 | 2.57 | 24.58 | 24.71 | 1.47 | 58.72 |
| 2018-2019 | 1 | 9 | 621619 | 100 | 80.26 | 7.60 | 88 | 11.08 | 62.32 | 2.17 | 20.87 | 20.98 | 1.47 | 56.87 |
| 2018-2019 | 1 | 9 | 627299 | 100 | 95.6 | 8.60 | 100 | 15.34 | 65.9 | 2.73 | 24.74 | 24.89 | 1.46 | 57.78 |
| 2018-2019 | 1 | 9 | 623291 | 100 | 89.83 | 8.00 | 100 | 8.36 | 49.81 | 3.6 | 19.3 | 19.63 | 1.39 | 46.11 |
| 2018-2019 | 1 | 9 | 627849 | 100 | 93.6 | 8.40 | 100 | 12.48 | 59.79 | 2.11 | 22.6 | 22.70 | 1.48 | 53.83 |
| 2018-2019 | 1 | 9 | 626978 | 100 | 100 | 9.00 | 100 | 13.69 | 60.69 | 3.21 | 23.41 | 23.63 | 1.43 | 54.13 |
| 2018-2019 | 1 | 9 | AKOVA | 100 | 92.56 | 8.40 | 92 | 10.81 | 55.08 | 5.2 | 21.07 | 21.70 | 1.33 | 50.11 |
| 2018-2019 | 1 | 9 | 627072 | 100 | 77.13 | 7.00 | 84 | 12.83 | 63.22 | 3.27 | 22.09 | 22.33 | 1.42 | 56.97 |
| 2018-2019 | 1 | 10 | 626908 | 100 | 93.07 | 8.20 | 100 | 11.66 | 55.97 | 5.15 | 21.71 | 22.31 | 1.34 | 50.64 |
| 2018-2019 | 1 | 10 | DARAB1 | 100 | 77.91 | 7.00 | 88 | 7.5 | 53.45 | 3.07 | 22.21 | 22.42 | 1.43 | 48.33 |
| 2018-2019 | 1 | 10 | 623421 | 100 | 74.6 | 7.00 | 84 | 10.02 | 58.18 | 3.66 | 19.79 | 20.13 | 1.39 | 53.59 |
| 2018-2019 | 1 | 10 | 623090 | 100 | 93.26 | 8.20 | 100 | 13.58 | 65.44 | 2.02 | 21.96 | 22.05 | 1.48 | 59.00 |
| 2018-2019 | 1 | 10 | 626234 | 100 | 87.26 | 8.00 | 96 | 8.61 | 51.11 | 4.43 | 17.86 | 18.40 | 1.33 | 47.76 |
| 2018-2019 | 1 | 10 | 627853 | 100 | 96.27 | 8.40 | 100 | 12.46 | 58.6 | 3.14 | 21.84 | 22.06 | 1.43 | 53.09 |
| 2018-2019 | 1 | 10 | GHABOUS | 100 | 82.95 | 7.40 | 96 | 10.16 | 53.95 | 2 | 20.5 | 20.60 | 1.47 | 49.55 |
| 2018-2019 | 1 | 10 | 624805 | 40 | 4.17 | 1.80 | 20 | 0.45 | 50.33 | 3.68 | 19.72 | 20.06 | 1.39 | 46.43 |
| 2018-2019 | 1 | 10 | 621565 | 100 | 69.79 | 6.60 | 80 | 10.72 | 58.23 | 3.01 | 24.32 | 24.51 | 1.45 | 51.57 |
| 2018-2019 | 1 | 10 | SABALAN | 100 | 95.31 | 8.40 | 100 | 13.87 | 64.43 | 2.28 | 23.37 | 23.48 | 1.47 | 57.38 |
| 2018-2019 | 1 | 11 | 626706 | 100 | 83.44 | 7.20 | 96 | 13.53 | 66.66 | 4.01 | 23.7 | 24.04 | 1.40 | 58.90 |
| 2018-2019 | 1 | 11 | 622063 | 100 | 91.14 | 8.20 | 96 | 8.39 | 55.99 | 2 | 18.77 | 18.88 | 1.46 | 52.11 |
| 2018-2019 | 1 | 11 | 626747 | 100 | 93.47 | 8.20 | 100 | 15.32 | 67.65 | 2.51 | 24.31 | 24.44 | 1.47 | 59.46 |
| 2018-2019 | 1 | 11 | 626573 | 100 | 92.54 | 8.20 | 100 | 15.84 | 67.21 | 4 | 25.08 | 25.40 | 1.41 | 58.52 |
| 2018-2019 | 1 | 11 | 622247 | 0 | 0 | 1.00 | 20 | 0.22 | 50.03 | 4.03 | 19.8 | 20.21 | 1.37 | 46.10 |
| 2018-2019 | 1 | 11 | 627990 | 100 | 84.06 | 7.80 | 96 | 14.06 | 67.57 | 5.35 | 23.04 | 23.65 | 1.34 | 59.86 |
| 2018-2019 | 1 | 11 | MAROON | 100 | 96.03 | 8.80 | 100 | 11.07 | 56.35 | 4.2 | 20.42 | 20.85 | 1.37 | 51.63 |
| 2018-2019 | 1 | 11 | 626226 | 100 | 95.51 | 8.60 | 100 | 14.67 | 66.67 | 4.13 | 22.52 | 22.90 | 1.39 | 59.56 |
| 2018-2019 | 1 | 11 | 626924 | 100 | 81.58 | 7.40 | 96 | 10.99 | 63.82 | 4.77 | 24.57 | 25.03 | 1.38 | 56.01 |
| 2018-2019 | 1 | 11 | 622084 | 0 | 0 | 1.00 | 20 | 0.34 | 47.34 | 1.87 | 16.64 | 16.74 | 1.46 | 44.74 |
| 2018-2019 | 1 | 12 | 624849 | 100 | 90.73 | 8.00 | 100 | 10.36 | 58.85 | 3.63 | 20.43 | 20.75 | 1.39 | 53.91 |
| 2018-2019 | 1 | 12 | 624576 | 100 | 86.72 | 7.80 | 96 | 9.57 | 54.5 | 2.88 | 22.16 | 22.35 | 1.44 | 49.31 |
| 2018-2019 | 1 | 12 | 626904 | 100 | 97.53 | 8.60 | 84 | 13.77 | 64.94 | 3.21 | 23.98 | 24.19 | 1.44 | 57.40 |
| 2018-2019 | 1 | 12 | 626895 | 100 | 97.4 | 8.60 | 100 | 14.92 | 65.86 | 4.57 | 25.09 | 25.50 | 1.39 | 57.39 |
| 2018-2019 | 1 | 12 | 627057 | 100 | 86.11 | 7.40 | 100 | 12.53 | 65.28 | 3.29 | 26.76 | 26.96 | 1.45 | 56.04 |
| 2018-2019 | 1 | 12 | SIRVAN | 100 | 99.25 | 9.00 | 100 | 2.92 | 60.71 | 2.5 | 23.7 | 23.83 | 1.47 | 54.05 |
| 2018-2019 | 1 | 12 | ALBORZ | 100 | 94.32 | 8.40 | 100 | 13.48 | 61.59 | 1.5 | 22.67 | 22.72 | 1.50 | 55.37 |
| 2018-2019 | 1 | 12 | 627987 | 100 | 93.59 | 8.20 | 100 | 12.25 | 58.12 | 1.41 | 21.63 | 21.68 | 1.51 | 52.84 |
| 2018-2019 | 1 | 12 | 624582 | 100 | 92.55 | 8.00 | 100 | 15.44 | 69.32 | 1.94 | 22.83 | 22.91 | 1.49 | 61.71 |
| 2018-2019 | 1 | 13 | 623909 | 100 | 100 | 9.00 | 100 | 11.48 | 57.47 | 5.04 | 20.18 | 20.80 | 1.33 | 52.66 |
| 2018-2019 | 1 | 13 | MARVDASHT | 100 | 100 | 9.00 | 100 | 11.7 | 56.1 | 4.94 | 21.07 | 21.64 | 1.34 | 51.06 |
| 2018-2019 | 1 | 13 | GAHAR | 100 | 98.64 | 8.80 | 100 | 0.12 | 52.11 | 3.71 | 18.05 | 18.43 | 1.37 | 48.69 |
| 2018-2019 | 1 | 13 | 627616 | 100 | 98.48 | 8.80 | 100 | 11.05 | 60.15 | 2.57 | 18.48 | 18.66 | 1.43 | 56.00 |
| 2018-2019 | 1 | 13 | 627102 | 100 | 33.79 | 4.80 | 48 | 2.38 | 51.3 | 5.07 | 18.73 | 19.40 | 1.31 | 47.58 |
| 2018-2019 | 1 | 13 | NAZ | 100 | 94.32 | 8.40 | 96 | 12.38 | 61.33 | 3.76 | 20.48 | 20.82 | 1.39 | 56.08 |
| 2018-2019 | 1 | 13 | 623109 | 100 | 96.69 | 8.60 | 96 | 16 | 68.98 | 5.49 | 23.84 | 24.46 | 1.34 | 60.49 |
| 2018-2019 | 1 | 13 | 623091 | 100 | 84.98 | 7.60 | 76 | 8.12 | 56.6 | 2.76 | 21.11 | 21.29 | 1.44 | 51.66 |
| 2018-2019 | 1 | 13 | BISTON | 100 | 65.22 | 6.40 | 80 | 6.3 | 55.22 | 1.79 | 20.08 | 20.16 | 1.48 | 50.89 |
| 2018-2019 | 1 | 14 | 625433 | 100 | 75.56 | 7.00 | 80 | 11.13 | 62.04 | 3.15 | 21.75 | 21.98 | 1.43 | 56.14 |
| 2018-2019 | 1 | 14 | 623161 | 100 | 96.12 | 8.60 | 100 | 9.4 | 55.25 | 2.2 | 20.95 | 21.07 | 1.47 | 50.54 |
| 2018-2019 | 1 | 14 | AZAR2 | 100 | 98.5 | 9.00 | 92 | 12.76 | 60.17 | 1.85 | 23.17 | 23.24 | 1.49 | 53.88 |
| 2018-2019 | 1 | 14 | 626699 | 100 | 78 | 7.00 | 88 | 10.32 | 57.41 | 2.32 | 21.1 | 21.23 | 1.46 | 52.41 |
| 2018-2019 | 1 | 14 | 627873 | 100 | 94.66 | 8.20 | 96 | 10.19 | 56.2 | 1.33 | 19.59 | 19.64 | 1.50 | 52.00 |
| 2018-2019 | 1 | 14 | 622379 | 100 | 86.72 | 7.40 | 100 | 13.92 | 65.31 | 1.8 | 22.84 | 22.91 | 1.49 | 58.43 |
| 2018-2019 | 1 | 14 | 627587 | 100 | 80.56 | 7.40 | 92 | 12.03 | 60.03 | 3.17 | 22.76 | 22.98 | 1.43 | 53.90 |
| 2018-2019 | 1 | 14 | 623127 | 100 | 91.41 | 8.00 | 96 | 12.39 | 57.43 | 3.86 | 22.33 | 22.66 | 1.40 | 51.77 |
| 2018-2019 | 1 | 14 | 623507 | 100 | 97.63 | 8.60 | 100 | 11.69 | 61.15 | 2.78 | 19.23 | 19.43 | 1.43 | 56.56 |
| 2018-2019 | 1 | 15 | SHIRAZ | 100 | 91.16 | 8.20 | 96 | 14.76 | 62.48 | 4.65 | 24.25 | 24.69 | 1.38 | 55.08 |
| 2018-2019 | 1 | 15 | MIHAN | 100 | 94.21 | 8.40 | 92 | 15.32 | 68.52 | 3.04 | 22.83 | 23.03 | 1.44 | 60.99 |
| 2018-2019 | 1 | 15 | 621650 | 100 | 94.19 | 8.20 | 84 | 13.96 | 64.42 | 4.26 | 22.09 | 22.50 | 1.38 | 57.90 |
| 2018-2019 | 1 | 15 | 627066 | 100 | 93.3 | 8.20 | 96 | 11.16 | 59.44 | 3.27 | 19.1 | 19.38 | 1.40 | 55.05 |
| 2018-2019 | 1 | 15 | FONG | 100 | 90.29 | 7.80 | 76 | 13.99 | 62.12 | 2.26 | 23.45 | 23.56 | 1.47 | 55.39 |
| 2018-2019 | 1 | 15 | 627787 | 100 | 98.12 | 8.80 | 92 | 11.2 | 61.94 | 1.59 | 18.62 | 18.69 | 1.49 | 57.60 |
| 2018-2019 | 1 | 15 | 623139 | 100 | 94.42 | 8.40 | 100 | 14.3 | 60.5 | 3.05 | 24.59 | 24.78 | 1.45 | 53.37 |
| 2018-2019 | 1 | 15 | 621908 | 100 | 92.76 | 8.20 | 92 | 14.48 | 66.3 | 3.67 | 22.34 | 22.64 | 1.41 | 59.40 |
| 2018-2019 | 1 | 16 | HAMOON | 100 | 88.84 | 7.80 | 96 | 12.8 | 66.91 | 1.61 | 21.87 | 21.93 | 1.50 | 60.30 |
| 2018-2019 | 1 | 16 | ALVAND | 100 | 82.28 | 7.40 | 76 | 14.81 | 67.91 | 3.14 | 23.96 | 24.16 | 1.44 | 59.83 |
| 2018-2019 | 1 | 16 | 624944 | 100 | 78.02 | 7.00 | 88 | 12.86 | 66.67 | 4.74 | 21.76 | 22.27 | 1.36 | 59.91 |
| 2018-2019 | 1 | 16 | 624846 | 100 | 86.4 | 7.60 | 96 | 10.97 | 61.79 | 3.86 | 19.87 | 20.24 | 1.38 | 56.76 |
| 2018-2019 | 1 | 16 | PISHTAZ | 100 | 98.18 | 9.00 | 88 | 12.29 | 59.87 | 3 | 20.74 | 20.96 | 1.43 | 54.73 |
| 2018-2019 | 1 | 16 | 623428 | 100 | 88.56 | 7.60 | 84 | 14.06 | 65.8 | 3.45 | 22.21 | 22.48 | 1.42 | 59.08 |
| 2018-2019 | 1 | 16 | 622894 | 100 | 90.17 | 8.00 | 96 | 14.03 | 67.61 | 4.64 | 22.4 | 22.88 | 1.37 | 60.35 |
| 2018-2019 | 1 | 16 | ARTA | 100 | 98.24 | 9.00 | 92 | 14.49 | 64.29 | 3.52 | 22.93 | 23.20 | 1.42 | 57.42 |
| 2018-2019 | 1 | 16 | 623162 | 100 | 86.92 | 7.80 | 96 | 14.01 | 71.2 | 5.15 | 21.13 | 21.75 | 1.33 | 63.91 |
| 2018-2019 | 1 | 17 | 628189 | 100 | 72.46 | 6.80 | 80 | 8.76 | 54.81 | 4.16 | 23.63 | 23.99 | 1.40 | 48.84 |
| 2018-2019 | 1 | 17 | INIA | 100 | 99.65 | 9.00 | 100 | 15.64 | 66.36 | 2.08 | 23.69 | 23.78 | 1.48 | 58.80 |
| 2018-2019 | 1 | 17 | REYHANI | 100 | 71.81 | 6.60 | 84 | 9.76 | 57.39 | 3.63 | 24.21 | 24.48 | 1.42 | 50.86 |
| 2018-2019 | 1 | 17 | 626215 | 100 | 44.62 | 5.40 | 64 | 8.31 | 60.09 | 2.56 | 24.5 | 24.63 | 1.47 | 53.10 |
| 2018-2019 | 1 | 17 | KARIM | 100 | 98.01 | 8.80 | 100 | 10.23 | 53.97 | 3.39 | 19.61 | 19.90 | 1.40 | 49.85 |
| 2018-2019 | 1 | 17 | 627414 | 100 | 88.68 | 8.20 | 100 | 12.99 | 67.95 | 3.43 | 22.18 | 22.44 | 1.42 | 60.87 |
| 2018-2019 | 1 | 17 | 627399 | 100 | 91.26 | 7.80 | 84 | 13.65 | 70.42 | 1.58 | 22.49 | 22.55 | 1.50 | 62.81 |
| 2018-2019 | 1 | 17 | 623318 | 100 | 59.6 | 6.00 | 80 | 5.74 | 56.53 | 2.33 | 18.44 | 18.59 | 1.45 | 52.72 |
| 2018-2019 | 1 | 17 | 627963 | 100 | 91.27 | 8.00 | 100 | 13.49 | 69.77 | 5.43 | 22.88 | 23.52 | 1.34 | 61.70 |
| 2018-2019 | 1 | 18 | KARAJ1 | 100 | 88.42 | 8.00 | 88 | 14.59 | 72.17 | 3.77 | 22.47 | 22.78 | 1.40 | 64.03 |
| 2018-2019 | 1 | 18 | DEZ | 100 | 86.03 | 7.60 | 92 | 9.51 | 64.82 | 3.45 | 22.21 | 22.48 | 1.42 | 58.25 |
| 2018-2019 | 1 | 18 | 621706 | 100 | 92.1 | 8.20 | 100 | 16.09 | 68.34 | 4.2 | 24.49 | 24.85 | 1.40 | 59.75 |
| 2018-2019 | 1 | 18 | 626358 | 100 | 99.33 | 8.80 | 100 | 14.57 | 65.57 | 4.56 | 23.78 | 24.21 | 1.38 | 57.91 |
| 2018-2019 | 1 | 18 | 623980 | 100 | 88.67 | 7.80 | 96 | 12.55 | 62.96 | 3.97 | 22.31 | 22.66 | 1.39 | 56.58 |
| 2018-2019 | 1 | 18 | 626776 | 100 | 95.74 | 8.60 | 100 | 8.49 | 58.51 | 1.9 | 17.12 | 17.23 | 1.46 | 55.08 |
| 2018-2019 | 1 | 18 | 625127 | 100 | 89.87 | 7.80 | 100 | 12.03 | 58.95 | 1.5 | 21.9 | 21.95 | 1.50 | 53.45 |
| 2018-2019 | 1 | 18 | CHAMRAN2 | 100 | 45.52 | 5.60 | 52 | 14.1 | 63.23 | 4.34 | 23 | 23.41 | 1.38 | 56.41 |
| 2018-2019 | 1 | 18 | NICKNEJAD | 60 | 8.82 | 2.60 | 28 | 2.83 | 52.69 | 4.55 | 20.63 | 21.13 | 1.35 | 48.19 |
| 2018-2019 | 1 | 18 | 623503 | 100 | 73.36 | 7.20 | 72 | 8.85 | 61.85 | 3.71 | 20.73 | 21.06 | 1.39 | 56.42 |
| 2018-2019 | 1 | 19 | FRONTANA | 0 | 0 | 1.00 | 12 | 16.59 | 70.04 | 2.35 | 25.62 | 25.73 | 1.48 | 60.51 |
| 2018-2019 | 1 | 19 | 627881 | 100 | 83.67 | 7.60 | 100 | 9.12 | 61.16 | 3.57 | 21.81 | 22.10 | 1.41 | 55.31 |
| 2018-2019 | 1 | 19 | 623377 | 100 | 95.77 | 8.40 | 96 | 15.46 | 72.13 | 1.79 | 23.41 | 23.48 | 1.49 | 63.56 |
| 2018-2019 | 1 | 19 | MV17 | 100 | 92 | 8.00 | 100 | 10.26 | 56.07 | 2.66 | 20.94 | 21.11 | 1.44 | 51.26 |
| 2018-2019 | 1 | 19 | 627948 | 100 | 91.83 | 8.20 | 96 | 12.72 | 61.25 | 3.4 | 22.45 | 22.71 | 1.42 | 55.09 |
| 2018-2019 | 1 | 19 | BAHAR | 100 | 73.88 | 6.80 | 80 | 11.27 | 56.03 | 3.09 | 22.17 | 22.38 | 1.43 | 50.66 |
| 2018-2019 | 1 | 19 | 624818 | 100 | 90.33 | 7.80 | 100 | 5.95 | 48.78 | 2.48 | 17.41 | 17.59 | 1.43 | 45.85 |
| 2018-2019 | 1 | 19 | 623345 | 100 | 91.63 | 8.00 | 88 | 13.4 | 69.65 | 4.08 | 23.01 | 23.37 | 1.40 | 61.70 |
| 2018-2019 | 1 | 20 | 627908 | 100 | 97.93 | 8.80 | 100 | 11.07 | 59.08 | 4.73 | 21.29 | 21.81 | 1.35 | 53.63 |
| 2018-2019 | 1 | 20 | 623169 | 100 | 31.9 | 4.80 | 48 | 2.87 | 56.27 | 3.85 | 21.91 | 22.25 | 1.40 | 50.94 |
| 2018-2019 | 1 | 20 | ATRAK | 100 | 29.75 | 4.80 | 48 | 6.33 | 53.87 | 3.88 | 21.84 | 22.18 | 1.39 | 48.81 |
| 2018-2019 | 1 | 20 | 627356 | 100 | 83.33 | 7.80 | 92 | 11.77 | 62.35 | 2.82 | 22.08 | 22.26 | 1.44 | 56.26 |
| 2018-2019 | 1 | 20 | 626943 | 100 | 71.73 | 6.60 | 76 | 4.77 | 48.16 | 2.47 | 18.41 | 18.57 | 1.44 | 44.93 |
| 2018-2019 | 1 | 20 | 627417 | 100 | 96.45 | 8.60 | 96 | 8.97 | 57.6 | 2.83 | 18.98 | 19.19 | 1.42 | 53.46 |
| 2018-2019 | 1 | 20 | 628088 | 100 | 93.73 | 8.40 | 96 | 11.99 | 64.88 | 2.4 | 20.85 | 20.99 | 1.46 | 59.09 |
| 2018-2019 | 1 | 20 | BAYAT | 100 | 92.91 | 8.20 | 84 | 12.68 | 62.52 | 2.43 | 23.61 | 23.73 | 1.47 | 55.64 |
| 2018-2019 | 1 | 20 | FALAT | 100 | 96.73 | 8.60 | 88 | 12.57 | 56.57 | 4.31 | 22.77 | 23.17 | 1.38 | 50.77 |
| 2018-2019 | 1 | 20 | 626933 | 100 | 79.84 | 7.20 | 88 | 12.35 | 56.76 | 2.76 | 23.35 | 23.51 | 1.45 | 50.78 |
| 2018-2019 | 1 | 21 | 623125 | 100 | 63.22 | 6.20 | 80 | 10.2 | 60.2 | 4.51 | 22.64 | 23.08 | 1.37 | 53.99 |
| 2018-2019 | 1 | 21 | DARAB2 | 100 | 95.28 | 8.40 | 88 | 6.1 | 45.01 | 3.87 | 17.44 | 17.86 | 1.35 | 42.18 |
| 2018-2019 | 1 | 21 | 624980 | 100 | 67.65 | 6.60 | 68 | 10.32 | 59.86 | 4.91 | 23.03 | 23.55 | 1.36 | 53.46 |
| 2018-2019 | 1 | 21 | 625281 | 60 | 14.77 | 3.20 | 36 | 2.95 | 55.04 | 2.11 | 18.68 | 18.80 | 1.46 | 51.27 |
| 2018-2019 | 1 | 21 | TAJAN | 100 | 89.76 | 7.80 | 88 | 11.2 | 59.1 | 4.19 | 20.07 | 20.50 | 1.36 | 54.25 |
| 2018-2019 | 1 | 21 | 623382 | 100 | 93.82 | 8.20 | 100 | 11.82 | 66.46 | 3.49 | 24.34 | 24.59 | 1.43 | 58.41 |
| 2018-2019 | 1 | 21 | 624580 | 80 | 11.05 | 2.60 | 32 | 1.53 | 50.47 | 3.47 | 18.8 | 19.12 | 1.39 | 46.91 |
| 2018-2019 | 1 | 21 | KAVIR | 100 | 75.85 | 7.00 | 84 | 8.43 | 59.2 | 3.05 | 22.24 | 22.45 | 1.43 | 53.43 |
| 2018-2019 | 1 | 21 | 626883 | 100 | 90.12 | 7.80 | 84 | 14.31 | 70.12 | 1.09 | 23.27 | 23.30 | 1.52 | 62.11 |
| 2018-2019 | 1 | 21 | BAM | 100 | 79.81 | 7.40 | 88 | 11.74 | 66.38 | 2.32 | 23.49 | 23.60 | 1.47 | 58.92 |
| 2018-2019 | 1 | 22 | 623136 | 100 | 48.51 | 5.00 | 56 | 8.04 | 61.16 | 3.54 | 20.33 | 20.64 | 1.40 | 56.02 |
| 2018-2019 | 1 | 22 | KAVEH | 100 | 87.5 | 7.80 | 100 | 12.48 | 57.36 | 2.64 | 23.35 | 23.50 | 1.46 | 51.31 |
| 2018-2019 | 1 | 22 | 626923 | 100 | 30.04 | 5.60 | 48 | 6 | 66.74 | 2.33 | 22.55 | 22.67 | 1.47 | 59.75 |
| 2018-2019 | 1 | 22 | MOGHAN1 | 100 | 56.23 | 6.00 | 76 | 7.79 | 59.47 | 2.63 | 19.45 | 19.63 | 1.44 | 54.97 |
| 2018-2019 | 1 | 22 | 627099 | 100 | 81.61 | 7.60 | 92 | 8.15 | 53.7 | 1.38 | 17.54 | 17.59 | 1.49 | 50.47 |
| 2018-2019 | 1 | 22 | 626260 | 100 | 36.09 | 5.20 | 40 | 3.12 | 50.57 | 3.75 | 18.15 | 18.53 | 1.37 | 47.21 |
| 2018-2019 | 1 | 22 | DARYA | 100 | 39.06 | 5.00 | 40 | 10.75 | 54.77 | 4.11 | 21.46 | 21.85 | 1.38 | 49.77 |
| 2018-2019 | 1 | 22 | 623176 | 100 | 66.28 | 6.80 | 80 | 4.77 | 55.25 | 3.08 | 21.03 | 21.25 | 1.43 | 50.46 |
| 2018-2019 | 1 | 22 | 621492 | 100 | 28.38 | 4.40 | 44 | 3.32 | 61.96 | 4.19 | 22.93 | 23.31 | 1.39 | 55.39 |
| 2018-2019 | 1 | 22 | URUOM | 100 | 97.06 | 8.60 | 88 | 11.45 | 63.33 | 3.19 | 21.45 | 21.69 | 1.42 | 57.40 |
| 2018-2019 | 1 | 23 | 627723 | 100 | 65.75 | 6.20 | 76 | 8.92 | 59.12 | 4.44 | 20.61 | 21.08 | 1.36 | 54.00 |
| 2018-2019 | 1 | 23 | BEZOSTAYA | 100 | 19.77 | 4.00 | 44 | 1.38 | 55.67 | 5.48 | 20.29 | 21.02 | 1.31 | 50.94 |
| 2018-2019 | 1 | 23 | 626565 | 100 | 69.67 | 6.60 | 80 | 7.7 | 51.88 | 2.61 | 17.43 | 17.62 | 1.42 | 48.75 |
| 2018-2019 | 1 | 23 | 4820 | 100 | 79.41 | 7.60 | 84 | 10.11 | 66.09 | 2.54 | 22.35 | 22.49 | 1.46 | 59.31 |
| 2018-2019 | 1 | 23 | OFOG | 100 | 95.38 | 8.60 | 76 | 9.43 | 62.46 | 2.72 | 22.91 | 23.07 | 1.45 | 55.94 |
| 2018-2019 | 1 | 23 | 624946 | 80 | 31.11 | 4.00 | 44 | 2.34 | 61.85 | 2.01 | 21.76 | 21.85 | 1.48 | 56.03 |
| 2018-2019 | 1 | 23 | 624894 | 100 | 73.56 | 7.40 | 76 | 6.47 | 53.62 | 1.73 | 16.33 | 16.42 | 1.47 | 50.80 |
| 2018-2019 | 1 | 23 | AZAR1 | 100 | 77.13 | 7.20 | 84 | 4.33 | 49.48 | 2.99 | 18.26 | 18.50 | 1.41 | 46.20 |
| 2018-2019 | 1 | 23 | 626958 | 100 | 55.7 | 6.20 | 68 | 6.44 | 66.97 | 1.47 | 23.02 | 23.07 | 1.51 | 59.71 |
| 2018-2019 | 1 | 23 | 623417 | 100 | 97.56 | 8.80 | 96 | 7.72 | 62.34 | 2.32 | 18.02 | 18.17 | 1.44 | 58.19 |
| 2018-2019 | 1 | 24 | 624378 | 80 | 18.4 | 3.60 | 36 | 3.72 | 57.73 | 3.6 | 19.79 | 20.11 | 1.39 | 53.19 |
| 2018-2019 | 1 | 24 | SISTAN | 100 | 89.73 | 8.00 | 100 | 7.57 | 53.85 | 3.34 | 18.83 | 19.12 | 1.40 | 50.04 |
| 2018-2019 | 1 | 24 | 626872 | 100 | 92.8 | 8.00 | 92 | 6.53 | 49.57 | 3.03 | 15.58 | 15.87 | 1.38 | 47.13 |
| 2018-2019 | 1 | 24 | 624381 | 100 | 46.46 | 5.20 | 72 | 9.08 | 68.81 | 3.34 | 22.13 | 22.38 | 1.42 | 61.61 |
| 2018-2019 | 1 | 24 | 625810 | 60 | 16.84 | 3.00 | 36 | 3.92 | 62.03 | 3.63 | 21.95 | 22.25 | 1.41 | 55.99 |
| 2018-2019 | 1 | 24 | 622311 | 100 | 76.03 | 6.60 | 92 | 7.99 | 60.4 | 3.25 | 18.25 | 18.54 | 1.39 | 56.28 |
| 2018-2019 | 1 | 24 | 627385 | 80 | 92.52 | 8.20 | 84 | 12.27 | 65.26 | 3.65 | 22.41 | 22.71 | 1.41 | 58.50 |
| 2018-2019 | 1 | 24 | 623905 | 100 | 41.44 | 5.00 | 60 | 4.4 | 49.98 | 3.63 | 18.3 | 18.66 | 1.37 | 46.61 |
| 2018-2019 | 1 | 24 | 627460 | 100 | 88.37 | 7.80 | 92 | 12.67 | 63.93 | 4.03 | 21.76 | 22.13 | 1.39 | 57.68 |
| 2018-2019 | 1 | 25 | 627760 | 100 | 26.32 | 4.20 | 44 | 6 | 68.53 | 2.31 | 24.48 | 24.59 | 1.48 | 60.06 |
| 2018-2019 | 1 | 25 | 625362 | 100 | 73.81 | 6.60 | 60 | 8.03 | 57.2 | 3 | 24.18 | 24.37 | 1.45 | 50.75 |
| 2018-2019 | 1 | 25 | 623274 | 100 | 99.4 | 9.00 | 88 | 12.57 | 64.33 | 3.12 | 21.33 | 21.56 | 1.43 | 58.32 |
| 2018-2019 | 1 | 25 | 626736 | 100 | 91.41 | 8.20 | 96 | 7.78 | 57.17 | 2.54 | 22.01 | 22.16 | 1.46 | 51.78 |
| 2018-2019 | 1 | 25 | 624585 | 60 | 11.67 | 2.60 | 16 | 1.63 | 48.7 | 4.16 | 18.58 | 19.04 | 1.35 | 45.28 |
| 2018-2019 | 1 | 25 | KOOHDASHT | 80 | 91.83 | 8.20 | 92 | 12.78 | 64.08 | 1.87 | 25.54 | 25.61 | 1.50 | 55.89 |
| 2018-2019 | 1 | 25 | NAVID | 100 | 87.35 | 7.80 | 100 | 13.48 | 68.19 | 1.52 | 22.68 | 22.73 | 1.50 | 60.90 |
| 2018-2019 | 1 | 25 | KARAJ2 | 100 | 100 | 9.00 | 100 | 14.68 | 66.18 | 1.18 | 23.11 | 23.14 | 1.52 | 59.02 |
| 2018-2019 | 1 | 25 | CHAMRAN | 100 | 98.99 | 8.80 | 84 | 0.13 | 50.23 | 1.55 | 16.55 | 16.62 | 1.48 | 47.53 |
| 2018-2019 | 1 | 25 | 624864 | 100 | 82.39 | 7.40 | 80 | 5.76 | 62.62 | 3.74 | 20.49 | 20.83 | 1.39 | 57.21 |
| 2018-2019 | 1 | 26 | 625123 | 100 | 100 | 9.00 | 100 | 10.8 | 55.94 | 2.74 | 20.13 | 20.32 | 1.44 | 51.48 |
| 2018-2019 | 1 | 26 | CASGOGEN | 0 | 0 | 1.00 | 20 | 8.68 | 50.68 | 2.7 | 17.19 | 17.40 | 1.42 | 47.70 |
| 2018-2019 | 1 | 26 | 626764 | 100 | 48.33 | 5.80 | 80 | 4.76 | 52.79 | 3.09 | 17.34 | 17.61 | 1.39 | 49.61 |
| 2018-2019 | 1 | 26 | 624804 | 100 | 48.82 | 5.80 | 64 | 4.59 | 58.55 | 3.63 | 19.02 | 19.36 | 1.38 | 54.25 |
| 2018-2019 | 1 | 26 | 626846 | 100 | 95.07 | 8.60 | 92 | 7.23 | 49.66 | 3.58 | 16.15 | 16.54 | 1.35 | 47.01 |
| 2018-2019 | 1 | 26 | ZARRIN | 100 | 45.56 | 5.40 | 64 | 4.43 | 54.25 | 4.59 | 20.33 | 20.84 | 1.35 | 49.73 |
| 2018-2019 | 1 | 26 | 626814 | 100 | 99.32 | 9.00 | 92 | 9.25 | 57.13 | 2.45 | 21.66 | 21.80 | 1.46 | 51.91 |
| 2018-2019 | 1 | 26 | 626825 | 100 | 95.29 | 8.20 | 80 | 11.44 | 60.85 | 2.6 | 22.15 | 22.30 | 1.45 | 54.94 |
| 2018-2019 | 1 | 26 | DEYHEM | 100 | 83.53 | 8.00 | 72 | 8.69 | 51.99 | 3.56 | 17.13 | 17.50 | 1.37 | 48.90 |
| 2018-2019 | 1 | 26 | 624251 | 100 | 79.23 | 7.20 | 64 | 5.81 | 57.34 | 2.79 | 21.24 | 21.42 | 1.44 | 52.26 |
| 2018-2019 | 1 | 27 | 623953 | 0 | 0 | 1.00 | 20 | 0.21 | 66.9 | 2.1 | 25.67 | 25.76 | 1.49 | 58.06 |
| 2018-2019 | 1 | 27 | 626855 | 100 | 93.33 | 8.60 | 92 | 15.25 | 65.04 | 2.54 | 24.97 | 25.10 | 1.47 | 56.96 |
| 2018-2019 | 1 | 27 | 623008 | 100 | 95.58 | 8.60 | 96 | 11.09 | 57.06 | 2.85 | 21.56 | 21.75 | 1.44 | 51.87 |
| 2018-2019 | 1 | 27 | 621669 | 100 | 96.75 | 8.60 | 100 | 10.86 | 59.41 | 4.62 | 19.27 | 19.82 | 1.34 | 54.83 |
| 2018-2019 | 1 | 27 | SHARYAR | 100 | 95.51 | 8.60 | 100 | 9.72 | 67.59 | 4.81 | 23.62 | 24.10 | 1.37 | 59.61 |
| 2018-2019 | 1 | 27 | ADL | 100 | 94.26 | 8.40 | 100 | 10.52 | 60.29 | 2.1 | 18.06 | 18.18 | 1.46 | 56.33 |
| 2018-2019 | 1 | 27 | PANJAMO62 | 100 | 75 | 7.00 | 84 | 13.07 | 69.15 | 3.36 | 22.7 | 22.95 | 1.42 | 61.55 |
| 2018-2019 | 1 | 27 | 624939 | 100 | 93.52 | 8.40 | 96 | 10.88 | 64.63 | 3.97 | 19.88 | 20.27 | 1.37 | 59.23 |
| 2018-2019 | 1 | 27 | PISHGAM | 100 | 75.34 | 6.80 | 84 | 12.05 | 66 | 4.19 | 23.56 | 23.93 | 1.39 | 58.42 |
| 2018-2019 | 1 | 27 | 626360 | 100 | 78.22 | 7.20 | 76 | 10.58 | 58.56 | 4.36 | 21.46 | 21.90 | 1.37 | 53.13 |
| 2018-2019 | 1 | 28 | 624983 | 100 | 83.96 | 7.40 | 92 | 8.95 | 62.69 | 5.12 | 22.63 | 23.20 | 1.35 | 56.06 |
| 2018-2019 | 1 | 28 | SIVAND | 100 | 78.98 | 7.20 | 68 | 13.69 | 60.54 | 4.5 | 23.99 | 24.41 | 1.39 | 53.60 |
| 2018-2019 | 1 | 28 | 627551 | 60 | 5 | 2.20 | 8 | 0.53 | 45.07 | 4.63 | 18.45 | 19.02 | 1.32 | 41.87 |
| 2018-2019 | 1 | 28 | SHAHI | 100 | 81.2 | 7.60 | 64 | 11.2 | 58.75 | 4.54 | 21.99 | 22.45 | 1.37 | 53.03 |
| 2018-2019 | 1 | 28 | 621717 | 100 | 25 | 5.40 | 36 | 3.7 | 61.2 | 2.3 | 24.13 | 24.24 | 1.48 | 54.25 |
| 2018-2019 | 1 | 28 | ARVAND | 100 | 90.23 | 8.20 | 76 | 9.52 | 56.41 | 2.18 | 18.97 | 19.09 | 1.46 | 52.41 |
| 2018-2019 | 1 | 28 | MORVARID | 100 | 94.74 | 8.40 | 100 | 11.57 | 58.07 | 2.18 | 23.69 | 23.79 | 1.48 | 51.79 |
| 2018-2019 | 1 | 28 | 624863 | 80 | 17.35 | 3.60 | 44 | 2.32 | 63.18 | 2.32 | 22.08 | 22.20 | 1.47 | 57.00 |
| 2018-2019 | 1 | 28 | 626881 | 100 | 38.94 | 5.20 | 60 | 9.72 | 64.23 | 3.72 | 23.66 | 23.95 | 1.41 | 56.95 |
| 2018-2019 | 1 | 28 | TOUBARI | 100 | 83.47 | 7.40 | 100 | 7.67 | 59.1 | 1.93 | 20.19 | 20.28 | 1.48 | 54.35 |
| 2018-2019 | 1 | 29 | SHINGHAI | 60 | 15.72 | 2.80 | 16 | 10.78 | 57.09 | 4.98 | 20.14 | 20.75 | 1.33 | 52.34 |
| 2018-2019 | 1 | 29 | KARAJ3 | 100 | 67.72 | 6.20 | 60 | 8.14 | 60.59 | 4.76 | 20.42 | 20.97 | 1.34 | 55.36 |
| 2018-2019 | 1 | 29 | 624947 | 100 | 90.71 | 8.00 | 80 | 7.99 | 55.46 | 4.53 | 19.61 | 20.13 | 1.34 | 51.12 |
| 2018-2019 | 1 | 29 | GOLESTAN | 100 | 93.88 | 8.40 | 88 | 10.61 | 63.61 | 3.13 | 18.81 | 19.07 | 1.41 | 58.92 |
| 2018-2019 | 1 | 29 | 624963 | 100 | 37.2 | 5.20 | 44 | 4.95 | 59.17 | 3.85 | 19.87 | 20.24 | 1.38 | 54.43 |
| 2018-2019 | 1 | 29 | AZADI | 100 | 94.96 | 8.40 | 80 | 11.02 | 55.15 | 3.08 | 21.14 | 21.36 | 1.43 | 50.32 |
| 2018-2019 | 1 | 29 | 623123 | 100 | 73.08 | 7.00 | 64 | 13.19 | 61.41 | 4.32 | 24.13 | 24.51 | 1.39 | 54.28 |
| 2018-2019 | 1 | 29 | AFLAK | 100 | 98.96 | 8.80 | 80 | 16.4 | 68.2 | 1.88 | 24.79 | 24.86 | 1.50 | 59.64 |
| 2018-2019 | 1 | 29 | KHAZAR1 | 100 | 79.6 | 7.20 | 68 | 9.83 | 59.96 | 1.62 | 22.46 | 22.52 | 1.50 | 54.06 |
| 2018-2019 | 1 | 30 | 623069 | 100 | 61.47 | 6.40 | 56 | 8.15 | 67.94 | 2.66 | 23.29 | 23.44 | 1.46 | 60.28 |
| 2018-2019 | 1 | 30 | ZAGROS | 100 | 97.87 | 8.80 | 80 | 9.3 | 60.52 | 1.88 | 20.89 | 20.97 | 1.48 | 55.29 |
| 2018-2019 | 1 | 30 | 624901 | 100 | 32.35 | 4.80 | 28 | 4.97 | 55.58 | 2.3 | 18.75 | 18.89 | 1.45 | 51.73 |
| 2018-2019 | 1 | 30 | SISON | 40 | 1.92 | 1.40 | 4 | 10.41 | 66.21 | 2.74 | 22.67 | 22.83 | 1.45 | 59.22 |
| 2018-2019 | 1 | 30 | 624215 | 80 | 11.38 | 3.00 | 12 | 0.41 | 50.55 | 2.01 | 17.19 | 17.31 | 1.45 | 47.61 |
| 2018-2019 | 1 | 30 | GHODS | 100 | 99.07 | 8.80 | 80 | 10.74 | 57.4 | 3.49 | 20.4 | 20.70 | 1.40 | 52.64 |
| 2018-2019 | 1 | 30 | 624861 | 100 | 89.33 | 8.00 | 76 | 9.42 | 68.04 | 2.99 | 22.34 | 22.54 | 1.44 | 60.89 |
| 2018-2019 | 1 | 30 | RIJAW | 100 | 99.66 | 9.00 | 80 | 14.64 | 65.67 | 3.94 | 23.36 | 23.69 | 1.40 | 58.29 |
| 2018-2019 | 1 | 30 | SPAHAN | 100 | 98.85 | 8.80 | 80 | 9.29 | 56.95 | 2.65 | 20.62 | 20.79 | 1.44 | 52.19 |
| 2018-2019 | 1 | 31 | 627055 | 100 | 94.16 | 8.20 | 80 | 7.19 | 60.55 | 3.89 | 19.14 | 19.53 | 1.37 | 55.98 |
| 2018-2019 | 1 | 31 | 626932 | 100 | 90.79 | 8.20 | 72 | 9.38 | 70.28 | 3.98 | 23.49 | 23.82 | 1.40 | 61.91 |
| 2018-2019 | 1 | 31 | PARSI | 100 | 97.54 | 8.80 | 80 | 14.87 | 68.33 | 4.31 | 21.9 | 22.32 | 1.38 | 61.26 |
| 2018-2019 | 1 | 31 | 624838 | 100 | 82.93 | 7.60 | 72 | 7.86 | 64.65 | 4.61 | 23.15 | 23.60 | 1.37 | 57.49 |
| 2018-2019 | 1 | 31 | 621736 | 100 | 52.66 | 5.80 | 48 | 4.91 | 63.36 | 2.36 | 23.66 | 23.78 | 1.47 | 56.32 |
| 2018-2019 | 1 | 31 | 621869 | 100 | 64.76 | 6.60 | 68 | 5.96 | 67.19 | 2.16 | 21.97 | 22.08 | 1.47 | 60.45 |
| 2018-2019 | 1 | 31 | 628012 | 100 | 25 | 4.40 | 32 | 1.57 | 54.99 | 2.78 | 18.6 | 18.81 | 1.42 | 51.22 |
| 2018-2019 | 1 | 31 | 622099 | 0 | 0 | 1.00 | 0 | 0.12 | 51.43 | 1.52 | 18.09 | 18.15 | 1.49 | 48.15 |
| 2018-2019 | 1 | 31 | 622105 | 0 | 0 | 1.00 | 0 | 0.11 | 47.27 | 5.77 | 19.62 | 20.45 | 1.28 | 43.44 |
| 2018-2019 | 1 | 32 | 621420 | 100 | 29.89 | 4.80 | 32 | 4.94 | 57.87 | 2.84 | 21.36 | 21.55 | 1.44 | 52.68 |
| 2018-2019 | 1 | 32 | 623266 | 20 | 1.1 | 1.40 | 4 | 1.84 | 55.22 | 2.22 | 19.14 | 19.27 | 1.46 | 51.25 |
| 2018-2019 | 1 | 32 | 621712 | 100 | 94.77 | 8.40 | 80 | 7.11 | 50.73 | 4.78 | 18.11 | 18.73 | 1.31 | 47.29 |
| 2018-2019 | 1 | 32 | 620903 | 100 | 96.64 | 8.60 | 80 | 5.73 | 50.72 | 1.59 | 16.75 | 16.83 | 1.48 | 47.93 |
| 2018-2019 | 1 | 32 | 625139 | 100 | 61.79 | 6.60 | 56 | 3.04 | 53.09 | 2.37 | 15.76 | 15.94 | 1.42 | 50.46 |
| 2018-2019 | 1 | 32 | 622098 | 100 | 97.2 | 8.80 | 80 | 10.78 | 69.74 | 4.28 | 22.1 | 22.51 | 1.38 | 62.29 |
| 2018-2019 | 1 | 32 | 625263 | 40 | 5.54 | 2.00 | 8 | 0.36 | 56.4 | 1.47 | 18.68 | 18.74 | 1.49 | 52.54 |
| 2018-2019 | 1 | 32 | 622264 | 0 | 0 | 1.00 | 0 | 0.12 | 51.89 | 4.87 | 19.42 | 20.02 | 1.33 | 47.89 |
| 2018-2019 | 1 | 32 | 622272 | 0 | 0 | 1.00 | 0 | 0.11 | 51.58 | 2.44 | 18.36 | 18.52 | 1.44 | 48.16 |
| 2018-2019 | 1 | 32 | 627905 | 100 | 29.02 | 4.80 | 32 | 1.05 | 55.91 | 1.18 | 15.5 | 15.54 | 1.49 | 53.25 |
| 2018-2019 | 2 | 1 | 627036 | 100 | 97.04 | 8.60 | 100 | 13.24 | 59.97 | 3.41 | 23.28 | 23.53 | 1.43 | 53.57 |
| 2018-2019 | 2 | 1 | 626156 | 80 | 69 | 6.60 | 72 | 9.06 | 51.59 | 3.62 | 21.15 | 21.46 | 1.40 | 47.05 |
| 2018-2019 | 2 | 1 | ROSHAN | 100 | 100 | 9.00 | 100 | 11.54 | 55.71 | 4.48 | 21.1 | 21.57 | 1.36 | 50.74 |
| 2018-2019 | 2 | 1 | 627852 | 100 | 100 | 9.00 | 100 | 13.34 | 62.28 | 3.71 | 23.67 | 23.96 | 1.42 | 55.31 |
| 2018-2019 | 2 | 1 | 628114 | 100 | 100 | 9.00 | 100 | 10.24 | 61.1 | 3.41 | 21.8 | 22.07 | 1.42 | 55.28 |
| 2018-2019 | 2 | 1 | 624985 | 100 | 100 | 9.00 | 100 | 9.07 | 51.39 | 5.47 | 22.84 | 23.49 | 1.34 | 46.01 |
| 2018-2019 | 2 | 1 | 624837 | 100 | 76.21 | 6.80 | 80 | 13.33 | 67.93 | 3.36 | 23.59 | 23.83 | 1.43 | 60.05 |
| 2018-2019 | 2 | 1 | TOUS | 100 | 70.04 | 6.80 | 76 | 5.97 | 53.63 | 4.22 | 20.91 | 21.33 | 1.37 | 48.96 |
| 2018-2019 | 2 | 1 | 623473 | 100 | 80.85 | 7.00 | 96 | 8.19 | 58.12 | 4.17 | 21.76 | 22.16 | 1.38 | 52.62 |
| 2018-2019 | 2 | 2 | MOGHAN2 | 100 | 95.11 | 8.40 | 100 | 15.01 | 60.41 | 4 | 24.2 | 24.53 | 1.41 | 53.43 |
| 2018-2019 | 2 | 2 | 627359 | 100 | 100 | 9.00 | 100 | 10.59 | 58.45 | 3.92 | 20.15 | 20.53 | 1.38 | 53.66 |
| 2018-2019 | 2 | 2 | 627038 | 100 | 100 | 9.00 | 100 | 12.11 | 58.64 | 5.19 | 21.33 | 21.95 | 1.33 | 53.18 |
| 2018-2019 | 2 | 2 | 621704 | 100 | 86.83 | 8.00 | 88 | 5.26 | 46.95 | 1.43 | 17.37 | 17.43 | 1.49 | 44.16 |
| 2018-2019 | 2 | 2 | 626566 | 100 | 85.51 | 7.80 | 84 | 8.1 | 51.82 | 4.4 | 19.75 | 20.23 | 1.35 | 47.74 |
| 2018-2019 | 2 | 2 | SHAHPASSAND | 100 | 97.6 | 8.80 | 100 | 15.62 | 69.63 | 3.85 | 23.66 | 23.97 | 1.41 | 61.31 |
| 2018-2019 | 2 | 2 | 623508 | 100 | 91.67 | 8.00 | 96 | 12.31 | 60.86 | 4.34 | 24.36 | 24.74 | 1.39 | 53.69 |
| 2018-2019 | 2 | 2 | 621735 | 100 | 100 | 9.00 | 100 | 8.77 | 51.74 | 1.97 | 18.88 | 18.98 | 1.47 | 48.14 |
| 2018-2019 | 2 | 2 | 623338 | 100 | 100 | 9.00 | 100 | 14.05 | 59.46 | 3.07 | 24.26 | 24.45 | 1.44 | 52.66 |
| 2018-2019 | 2 | 2 | 621668 | 100 | 100 | 9.00 | 100 | 8.09 | 54.17 | 3.16 | 17.18 | 17.47 | 1.39 | 50.95 |
| 2018-2019 | 2 | 3 | 621421 | 100 | 100 | 9.00 | 100 | 7.11 | 54.65 | 1.5 | 15.66 | 15.73 | 1.48 | 52.00 |
| 2018-2019 | 2 | 3 | 624240 | 100 | 100 | 9.00 | 100 | 9.78 | 57 | 4 | 21.38 | 21.75 | 1.39 | 51.81 |
| 2018-2019 | 2 | 3 | 623344 | 100 | 100 | 9.00 | 100 | 11.8 | 60.93 | 3.71 | 23.6 | 23.89 | 1.41 | 54.20 |
| 2018-2019 | 2 | 3 | 624941 | 0 | 0 | 1.00 | 0 | 0.15 | 48.63 | 3.95 | 18.39 | 18.81 | 1.36 | 45.29 |
| 2018-2019 | 2 | 3 | 621716 | 100 | 100 | 9.00 | 100 | 14.89 | 63.69 | 6.87 | 24.93 | 25.86 | 1.30 | 55.42 |
| 2018-2019 | 2 | 3 | 627236 | 100 | 100 | 9.00 | 100 | 15.27 | 70.88 | 4.28 | 22.31 | 22.72 | 1.38 | 63.07 |
| 2018-2019 | 2 | 3 | 624911 | 100 | 100 | 9.00 | 100 | 12.23 | 66.11 | 2.42 | 20.47 | 20.61 | 1.45 | 60.33 |
| 2018-2019 | 2 | 4 | OHADI | 100 | 97.67 | 8.80 | 100 | 14.39 | 70.38 | 4.77 | 22.38 | 22.88 | 1.36 | 62.57 |
| 2018-2019 | 2 | 4 | 624990 | 100 | 58.97 | 6.20 | 68 | 11.5 | 65.15 | 3.84 | 24.16 | 24.46 | 1.41 | 57.42 |
| 2018-2019 | 2 | 4 | 627484 | 100 | 100 | 9.00 | 100 | 9.63 | 59.3 | 6.19 | 21.72 | 22.58 | 1.29 | 53.45 |
| 2018-2019 | 2 | 4 | 627688 | 100 | 100 | 9.00 | 100 | 6.89 | 51.16 | 2.48 | 16.02 | 16.21 | 1.42 | 48.54 |
| 2018-2019 | 2 | 4 | 627416 | 100 | 100 | 9.00 | 100 | 14.56 | 68.45 | 3.61 | 22.15 | 22.44 | 1.41 | 61.28 |
| 2018-2019 | 2 | 4 | 625661 | 100 | 100 | 9.00 | 100 | 7.8 | 48.86 | 1.6 | 17.38 | 17.45 | 1.48 | 45.96 |
| 2018-2019 | 2 | 4 | 625081 | 100 | 100 | 9.00 | 100 | 9.86 | 58.6 | 4.34 | 21.84 | 22.27 | 1.37 | 52.99 |
| 2018-2019 | 2 | 4 | 627061 | 100 | 100 | 9.00 | 100 | 13.46 | 60.69 | 3.68 | 21.61 | 21.92 | 1.40 | 54.99 |
| 2018-2019 | 2 | 4 | NEISHABOUR | 100 | 100 | 9.00 | 100 | 13.77 | 65.64 | 4.38 | 22.16 | 22.59 | 1.38 | 58.88 |
| 2018-2019 | 2 | 4 | 624315 | 100 | 86.43 | 7.80 | 92 | 13.79 | 70.62 | 3.89 | 24.36 | 24.67 | 1.41 | 61.64 |
| 2018-2019 | 2 | 5 | 627103 | 100 | 22.8 | 4.20 | 32 | 0.65 | 49.69 | 1.97 | 16.06 | 16.18 | 1.45 | 47.15 |
| 2018-2019 | 2 | 5 | 623475 | 100 | 88.81 | 8.20 | 92 | 8.75 | 54.99 | 3.38 | 20.03 | 20.31 | 1.40 | 50.62 |
| 2018-2019 | 2 | 5 | 624596 | 60 | 47.06 | 4.80 | 48 | 11.11 | 57.64 | 4.2 | 20.99 | 21.41 | 1.37 | 52.54 |
| 2018-2019 | 2 | 5 | AKBARI | 100 | 81.62 | 7.80 | 84 | 7.77 | 58.59 | 3.69 | 20.38 | 20.71 | 1.39 | 53.70 |
| 2018-2019 | 2 | 5 | 623506 | 100 | 96.24 | 8.60 | 100 | 11.29 | 70.78 | 5.6 | 22.26 | 22.95 | 1.32 | 62.84 |
| 2018-2019 | 2 | 5 | 623510 | 100 | 84.3 | 7.60 | 88 | 7.36 | 52.02 | 4.8 | 19.72 | 20.30 | 1.33 | 47.90 |
| 2018-2019 | 2 | 5 | 627845 | 100 | 100 | 9.00 | 100 | 10.61 | 58.35 | 3.98 | 19.86 | 20.25 | 1.37 | 53.69 |
| 2018-2019 | 2 | 6 | 627856 | 60 | 5.61 | 2.40 | 12 | 0.26 | 51.74 | 2 | 18.18 | 18.29 | 1.46 | 48.39 |
| 2018-2019 | 2 | 6 | MAHDAVI | 100 | 98.51 | 8.80 | 100 | 15.17 | 66.09 | 3.79 | 25.4 | 25.68 | 1.42 | 57.46 |
| 2018-2019 | 2 | 6 | 627360 | 100 | 97.74 | 8.80 | 100 | 15.97 | 68.54 | 3.24 | 23.83 | 24.05 | 1.44 | 60.40 |
| 2018-2019 | 2 | 6 | 627883 | 100 | 82.05 | 7.60 | 84 | 12.37 | 70.23 | 3.98 | 22.03 | 22.39 | 1.39 | 62.75 |
| 2018-2019 | 2 | 6 | SHIROODI | 100 | 99.02 | 8.80 | 100 | 16.27 | 66.79 | 1.24 | 21.76 | 21.80 | 1.51 | 60.28 |
| 2018-2019 | 2 | 6 | 623379 | 100 | 97.4 | 8.60 | 100 | 10.36 | 57.42 | 5.17 | 23.8 | 24.36 | 1.36 | 50.95 |
| 2018-2019 | 2 | 6 | 624910 | 100 | 94.55 | 8.00 | 88 | 12.34 | 64.67 | 5.02 | 24.17 | 24.69 | 1.37 | 56.90 |
| 2018-2019 | 2 | 6 | MOGHAN3 | 100 | 98.44 | 8.80 | 100 | 17.45 | 64.25 | 4.06 | 25.71 | 26.03 | 1.41 | 55.78 |
| 2018-2019 | 2 | 6 | DASTJERDI | 100 | 96.03 | 8.60 | 100 | 4.01 | 69.73 | 3.47 | 23.18 | 23.44 | 1.42 | 61.72 |
| 2018-2019 | 2 | 7 | TAKAB | 60 | 19.89 | 3.40 | 24 | 1.01 | 49.44 | 4.04 | 16.69 | 17.17 | 1.33 | 46.60 |
| 2018-2019 | 2 | 7 | 623908 | 100 | 96.12 | 8.60 | 96 | 11.49 | 61.86 | 3.19 | 19.95 | 20.20 | 1.41 | 56.84 |
| 2018-2019 | 2 | 7 | RASHID | 100 | 94.33 | 8.60 | 96 | 12.68 | 69.4 | 3.68 | 21.64 | 21.95 | 1.40 | 62.34 |
| 2018-2019 | 2 | 7 | 624900 | 100 | 71.19 | 6.80 | 80 | 15.23 | 67.93 | 5.6 | 24.85 | 25.47 | 1.35 | 59.04 |
| 2018-2019 | 2 | 7 | 627054 | 100 | 97.19 | 8.60 | 100 | 13.02 | 53.89 | 6.01 | 24.21 | 24.94 | 1.33 | 47.58 |
| 2018-2019 | 2 | 7 | 626223 | 100 | 82.2 | 7.40 | 88 | 13.08 | 63.56 | 3.61 | 21.75 | 22.05 | 1.41 | 57.41 |
| 2018-2019 | 2 | 7 | 627189 | 100 | 97.21 | 8.80 | 100 | 13.6 | 68.73 | 2.89 | 22.01 | 22.20 | 1.44 | 61.65 |
| 2018-2019 | 2 | 7 | 627423 | 100 | 98.15 | 8.80 | 100 | 18.46 | 62.67 | 5.88 | 23.91 | 24.62 | 1.33 | 55.28 |
| 2018-2019 | 2 | 7 | ZARE | 100 | 88.04 | 7.80 | 96 | 13 | 61.75 | 4.24 | 20.51 | 20.94 | 1.37 | 56.39 |
| 2018-2019 | 2 | 8 | 627410 | 100 | 93.26 | 8.60 | 96 | 8.44 | 54.95 | 4.6 | 19.17 | 19.71 | 1.34 | 50.83 |
| 2018-2019 | 2 | 8 | DN11 | 100 | 95.81 | 8.40 | 96 | 11.97 | 67.32 | 3.3 | 24.22 | 24.44 | 1.44 | 59.19 |
| 2018-2019 | 2 | 8 | 625080 | 100 | 96.97 | 8.60 | 96 | 14.54 | 59.37 | 4.58 | 23.26 | 23.71 | 1.38 | 52.96 |
| 2018-2019 | 2 | 8 | HOMA | 100 | 92.5 | 8.20 | 100 | 13.15 | 67.42 | 3.94 | 23.49 | 23.82 | 1.40 | 59.64 |
| 2018-2019 | 2 | 8 | 627842 | 100 | 90.41 | 8.40 | 96 | 11.36 | 60.66 | 3.92 | 21.34 | 21.70 | 1.39 | 55.07 |
| 2018-2019 | 2 | 8 | 627043 | 100 | 85.64 | 7.60 | 96 | 7.69 | 59.96 | 1.98 | 17.85 | 17.96 | 1.46 | 56.12 |
| 2018-2019 | 2 | 8 | 624956 | 100 | 99.17 | 8.80 | 100 | 14.94 | 67.52 | 5.49 | 23 | 23.65 | 1.34 | 59.82 |
| 2018-2019 | 2 | 8 | 626261 | 100 | 96.76 | 8.60 | 100 | 12.15 | 58.24 | 3.86 | 23.74 | 24.05 | 1.41 | 51.81 |
| 2018-2019 | 2 | 8 | 628084 | 100 | 96.23 | 8.60 | 100 | 8.6 | 52.08 | 3.19 | 15.97 | 16.29 | 1.37 | 49.39 |
| 2018-2019 | 2 | 8 | VEE/NAC | 100 | 93.67 | 8.40 | 96 | 9.14 | 52.94 | 4.9 | 20.36 | 20.94 | 1.33 | 48.49 |
| 2018-2019 | 2 | 9 | 625047 | 100 | 96.53 | 8.60 | 100 | 14.51 | 64.93 | 3.88 | 22.42 | 22.75 | 1.40 | 58.20 |
| 2018-2019 | 2 | 9 | 626158 | 100 | 93.39 | 8.20 | 96 | 6.32 | 53.32 | 1.9 | 17.27 | 17.37 | 1.46 | 50.19 |
| 2018-2019 | 2 | 9 | 624925 | 100 | 86.55 | 8.00 | 92 | 11.24 | 67.91 | 2.72 | 26.32 | 26.46 | 1.47 | 58.41 |
| 2018-2019 | 2 | 9 | 621619 | 100 | 87.36 | 7.80 | 96 | 11.7 | 61.26 | 2.14 | 21.62 | 21.73 | 1.47 | 55.58 |
| 2018-2019 | 2 | 9 | 627299 | 100 | 87.16 | 7.60 | 92 | 15.27 | 65.99 | 2.76 | 25.25 | 25.40 | 1.46 | 57.55 |
| 2018-2019 | 2 | 9 | 623291 | 100 | 68.61 | 6.60 | 80 | 7.6 | 49.59 | 3.6 | 19.39 | 19.72 | 1.39 | 45.87 |
| 2018-2019 | 2 | 9 | 627849 | 100 | 90.95 | 8.00 | 100 | 11.14 | 59.95 | 2.07 | 22.98 | 23.07 | 1.48 | 53.78 |
| 2018-2019 | 2 | 9 | 626978 | 100 | 96.33 | 8.80 | 100 | 13.46 | 58.48 | 3.25 | 24.18 | 24.40 | 1.44 | 51.84 |
| 2018-2019 | 2 | 9 | AKOVA | 100 | 89.03 | 8.40 | 96 | 10.43 | 54.36 | 5.19 | 20.74 | 21.38 | 1.33 | 49.60 |
| 2018-2019 | 2 | 9 | 627072 | 100 | 95.67 | 8.60 | 100 | 14.38 | 62.24 | 3.27 | 21.68 | 21.93 | 1.42 | 56.34 |
| 2018-2019 | 2 | 10 | 626908 | 100 | 96.53 | 8.60 | 100 | 8.45 | 59.28 | 5.23 | 20.94 | 21.58 | 1.33 | 53.91 |
| 2018-2019 | 2 | 10 | DARAB1 | 100 | 86.36 | 7.60 | 96 | 6.57 | 50.48 | 3.04 | 22.29 | 22.50 | 1.44 | 45.61 |
| 2018-2019 | 2 | 10 | 623421 | 100 | 93.06 | 8.20 | 100 | 10.34 | 56.6 | 3.66 | 20.44 | 20.77 | 1.39 | 51.89 |
| 2018-2019 | 2 | 10 | 623090 | 100 | 93.99 | 8.20 | 100 | 16.96 | 65.95 | 2.03 | 22.16 | 22.25 | 1.48 | 59.32 |
| 2018-2019 | 2 | 10 | 626234 | 100 | 95.56 | 8.60 | 100 | 9.62 | 51.39 | 4.44 | 18.15 | 18.69 | 1.33 | 47.92 |
| 2018-2019 | 2 | 10 | 627853 | 100 | 99.18 | 9.00 | 100 | 11.29 | 58.78 | 3.16 | 22.47 | 22.69 | 1.43 | 52.95 |
| 2018-2019 | 2 | 10 | GHABOUS | 100 | 89.02 | 7.60 | 100 | 10.65 | 52.81 | 1.99 | 20.8 | 20.89 | 1.48 | 48.39 |
| 2018-2019 | 2 | 10 | 624805 | 60 | 10.42 | 2.60 | 16 | 0.44 | 49.41 | 3.7 | 20.03 | 20.37 | 1.39 | 45.46 |
| 2018-2019 | 2 | 10 | 621565 | 100 | 86.78 | 7.40 | 100 | 10.17 | 56.16 | 2.99 | 25.07 | 25.25 | 1.45 | 49.41 |
| 2018-2019 | 2 | 10 | SABALAN | 100 | 91.74 | 8.00 | 100 | 11.62 | 66.53 | 2.25 | 23.84 | 23.95 | 1.48 | 58.85 |
| 2018-2019 | 2 | 11 | 626706 | 100 | 94.16 | 8.60 | 100 | 12.42 | 65.1 | 4.11 | 22.42 | 22.79 | 1.39 | 58.32 |
| 2018-2019 | 2 | 11 | 622063 | 100 | 46.7 | 5.60 | 52 | 7.91 | 56.81 | 1.93 | 19.13 | 19.23 | 1.47 | 52.72 |
| 2018-2019 | 2 | 11 | 626747 | 100 | 93.06 | 8.40 | 96 | 14.57 | 68.24 | 2.44 | 26.28 | 26.39 | 1.48 | 58.70 |
| 2018-2019 | 2 | 11 | 626573 | 100 | 92.56 | 8.40 | 100 | 16.86 | 64.62 | 3.98 | 25.09 | 25.40 | 1.41 | 56.44 |
| 2018-2019 | 2 | 11 | 622247 | 0 | 0 | 1.00 | 0 | 0.2 | 50.17 | 4.02 | 19.81 | 20.21 | 1.37 | 46.23 |
| 2018-2019 | 2 | 11 | 627990 | 100 | 92.38 | 8.20 | 100 | 13.29 | 66.02 | 5.36 | 23.09 | 23.70 | 1.34 | 58.57 |
| 2018-2019 | 2 | 11 | MAROON | 100 | 89.06 | 7.60 | 100 | 10.18 | 55.58 | 4.22 | 20.68 | 21.11 | 1.37 | 50.82 |
| 2018-2019 | 2 | 11 | 626226 | 100 | 97.36 | 8.60 | 100 | 15.6 | 67.79 | 4.08 | 22.59 | 22.96 | 1.39 | 60.45 |
| 2018-2019 | 2 | 11 | 626924 | 100 | 64.74 | 6.40 | 80 | 12.41 | 63.84 | 4.73 | 25.22 | 25.66 | 1.39 | 55.66 |
| 2018-2019 | 2 | 11 | 622084 | 0 | 0 | 1.00 | 0 | 0.3 | 47.08 | 1.76 | 14.65 | 14.76 | 1.45 | 45.06 |
| 2018-2019 | 2 | 12 | 624849 | 100 | 97.97 | 8.80 | 100 | 10.66 | 58 | 3.55 | 20.98 | 21.28 | 1.40 | 52.92 |
| 2018-2019 | 2 | 12 | 624576 | 100 | 52.49 | 5.60 | 64 | 10.01 | 54.64 | 2.89 | 22.11 | 22.30 | 1.44 | 49.46 |
| 2018-2019 | 2 | 12 | 626904 | 100 | 88.56 | 7.80 | 96 | 14.61 | 64.71 | 3.28 | 24.52 | 24.74 | 1.44 | 56.90 |
| 2018-2019 | 2 | 12 | 626895 | 100 | 92.24 | 8.20 | 100 | 16.23 | 65.5 | 4.64 | 25.29 | 25.71 | 1.39 | 56.97 |
| 2018-2019 | 2 | 12 | 627057 | 100 | 92.16 | 8.20 | 100 | 11.34 | 66.27 | 3.3 | 27.29 | 27.49 | 1.45 | 56.49 |
| 2018-2019 | 2 | 12 | SIRVAN | 100 | 99.69 | 9.00 | 100 | 2.36 | 61.5 | 2.53 | 23.69 | 23.82 | 1.46 | 54.72 |
| 2018-2019 | 2 | 12 | ALBORZ | 100 | 91.87 | 8.00 | 100 | 12.74 | 61.18 | 1.55 | 22.07 | 22.12 | 1.50 | 55.32 |
| 2018-2019 | 2 | 12 | 627987 | 100 | 95.95 | 8.40 | 100 | 11.84 | 57.31 | 1.52 | 22.09 | 22.14 | 1.50 | 51.91 |
| 2018-2019 | 2 | 12 | 624582 | 100 | 97.66 | 8.80 | 100 | 14.89 | 71.99 | 1.95 | 22.95 | 23.03 | 1.49 | 63.74 |
| 2018-2019 | 2 | 13 | 623909 | 100 | 95.78 | 8.40 | 100 | 10.97 | 57.75 | 5.03 | 20.27 | 20.88 | 1.33 | 52.87 |
| 2018-2019 | 2 | 13 | MARVDASHT | 100 | 95.69 | 8.60 | 100 | 10.68 | 54.91 | 4.97 | 21.12 | 21.70 | 1.34 | 49.96 |
| 2018-2019 | 2 | 13 | GAHAR | 100 | 100 | 9.00 | 100 | 0.1 | 52.52 | 3.68 | 17.73 | 18.11 | 1.37 | 49.18 |
| 2018-2019 | 2 | 13 | 627616 | 100 | 97.66 | 8.80 | 100 | 11.83 | 57.21 | 2.52 | 18.6 | 18.77 | 1.44 | 53.27 |
| 2018-2019 | 2 | 13 | 627102 | 100 | 38.91 | 5.40 | 48 | 2.04 | 50.53 | 5.1 | 18.1 | 18.80 | 1.30 | 47.08 |
| 2018-2019 | 2 | 13 | NAZ | 100 | 100 | 9.00 | 100 | 11.09 | 60.87 | 3.73 | 20.23 | 20.57 | 1.39 | 55.79 |
| 2018-2019 | 2 | 13 | 623109 | 100 | 97.14 | 8.60 | 100 | 14.28 | 67.58 | 5.51 | 24.01 | 24.63 | 1.35 | 59.28 |
| 2018-2019 | 2 | 13 | 623091 | 100 | 57.87 | 6.00 | 72 | 7.66 | 58.73 | 2.71 | 20.9 | 21.07 | 1.44 | 53.66 |
| 2018-2019 | 2 | 13 | BISTON | 100 | 24.1 | 4.00 | 36 | 6 | 54.46 | 1.8 | 19.85 | 19.93 | 1.48 | 50.29 |
| 2018-2019 | 2 | 14 | 625433 | 100 | 92.8 | 8.00 | 100 | 10.55 | 61.98 | 3.18 | 21.77 | 22.00 | 1.43 | 56.07 |
| 2018-2019 | 2 | 14 | 623161 | 100 | 53.04 | 5.80 | 64 | 9.2 | 54.74 | 2.16 | 21.41 | 21.52 | 1.47 | 49.88 |
| 2018-2019 | 2 | 14 | AZAR2 | 100 | 93.85 | 8.00 | 100 | 13.06 | 58.8 | 1.76 | 24.02 | 24.08 | 1.50 | 52.28 |
| 2018-2019 | 2 | 14 | 626699 | 100 | 87.8 | 7.80 | 96 | 7.63 | 56.89 | 2.34 | 20.9 | 21.03 | 1.46 | 52.03 |
| 2018-2019 | 2 | 14 | 627873 | 100 | 83.56 | 7.40 | 96 | 10.32 | 53.38 | 1.37 | 18.25 | 18.30 | 1.50 | 49.92 |
| 2018-2019 | 2 | 14 | 622379 | 100 | 95.37 | 8.40 | 100 | 13.04 | 65.55 | 1.79 | 23.63 | 23.70 | 1.50 | 58.19 |
| 2018-2019 | 2 | 14 | 627587 | 100 | 99.2 | 8.80 | 100 | 12.67 | 61.76 | 3.31 | 23.08 | 23.32 | 1.43 | 55.21 |
| 2018-2019 | 2 | 14 | 623127 | 100 | 98.55 | 9.00 | 100 | 14.21 | 54.53 | 3.84 | 23.09 | 23.41 | 1.41 | 48.86 |
| 2018-2019 | 2 | 14 | 623507 | 100 | 100 | 9.00 | 100 | 13.29 | 61.03 | 2.74 | 19.2 | 19.39 | 1.43 | 56.47 |
| 2018-2019 | 2 | 15 | SHIRAZ | 100 | 100 | 9.00 | 100 | 15.73 | 63.6 | 4.53 | 23.28 | 23.72 | 1.38 | 56.56 |
| 2018-2019 | 2 | 15 | MIHAN | 100 | 98.91 | 8.80 | 100 | 14.49 | 70.6 | 3.09 | 22.45 | 22.66 | 1.43 | 62.88 |
| 2018-2019 | 2 | 15 | 621650 | 100 | 100 | 9.00 | 100 | 10.83 | 64.1 | 4.18 | 21.96 | 22.35 | 1.38 | 57.71 |
| 2018-2019 | 2 | 15 | 627066 | 100 | 100 | 9.00 | 100 | 8.99 | 59.87 | 3.17 | 20.28 | 20.53 | 1.42 | 54.93 |
| 2018-2019 | 2 | 15 | FONG | 100 | 85.14 | 7.40 | 100 | 14.95 | 63.86 | 2.27 | 24.19 | 24.30 | 1.48 | 56.45 |
| 2018-2019 | 2 | 15 | 627787 | 100 | 90.26 | 7.80 | 96 | 12.86 | 61.19 | 1.57 | 18.89 | 18.96 | 1.49 | 56.81 |
| 2018-2019 | 2 | 15 | 623139 | 100 | 91.04 | 8.00 | 100 | 13.91 | 60.69 | 3.09 | 24.45 | 24.64 | 1.45 | 53.60 |
| 2018-2019 | 2 | 15 | 621908 | 100 | 98.32 | 8.80 | 100 | 12.28 | 67.2 | 3.6 | 23.45 | 23.72 | 1.42 | 59.52 |
| 2018-2019 | 2 | 16 | HAMOON | 100 | 61.71 | 6.40 | 72 | 15.59 | 69.67 | 1.6 | 22.55 | 22.61 | 1.50 | 62.17 |
| 2018-2019 | 2 | 16 | ALVAND | 100 | 92.02 | 8.00 | 100 | 15.29 | 69.8 | 3.14 | 23.89 | 24.10 | 1.44 | 61.37 |
| 2018-2019 | 2 | 16 | 624944 | 100 | 87.45 | 7.60 | 100 | 12.09 | 65.71 | 4.75 | 22.12 | 22.62 | 1.36 | 58.92 |
| 2018-2019 | 2 | 16 | 624846 | 100 | 80 | 7.00 | 88 | 12.34 | 60.64 | 3.95 | 20.09 | 20.47 | 1.38 | 55.63 |
| 2018-2019 | 2 | 16 | PISHTAZ | 100 | 97.84 | 8.60 | 100 | 12.41 | 61.77 | 2.91 | 21.29 | 21.49 | 1.43 | 56.14 |
| 2018-2019 | 2 | 16 | 623428 | 100 | 96.14 | 8.60 | 100 | 13.08 | 66.59 | 3.41 | 22.61 | 22.87 | 1.42 | 59.51 |
| 2018-2019 | 2 | 16 | 622894 | 100 | 100 | 9.00 | 100 | 15.37 | 67.48 | 4.68 | 22.14 | 22.63 | 1.36 | 60.38 |
| 2018-2019 | 2 | 16 | ARTA | 100 | 99.69 | 9.00 | 100 | 16.1 | 66.49 | 3.56 | 22.51 | 22.79 | 1.41 | 59.47 |
| 2018-2019 | 2 | 16 | 623162 | 100 | 99.56 | 9.00 | 100 | 15.83 | 71.54 | 5.18 | 21.16 | 21.78 | 1.33 | 64.16 |
| 2018-2019 | 2 | 17 | 628189 | 100 | 22.01 | 4.00 | 28 | 9.26 | 53.35 | 4.16 | 23.59 | 23.95 | 1.40 | 47.56 |
| 2018-2019 | 2 | 17 | INIA | 100 | 98.26 | 8.80 | 100 | 15.83 | 65.77 | 2.22 | 23.36 | 23.47 | 1.48 | 58.50 |
| 2018-2019 | 2 | 17 | REYHANI | 100 | 32.8 | 4.80 | 48 | 9.36 | 57.39 | 3.58 | 24.4 | 24.66 | 1.43 | 50.77 |
| 2018-2019 | 2 | 17 | 626215 | 100 | 32.79 | 4.80 | 40 | 8.76 | 59.39 | 2.67 | 24.76 | 24.90 | 1.46 | 52.36 |
| 2018-2019 | 2 | 17 | KARIM | 100 | 88.67 | 7.80 | 100 | 11 | 54.46 | 3.41 | 20.15 | 20.44 | 1.40 | 50.08 |
| 2018-2019 | 2 | 17 | 627414 | 100 | 89.82 | 7.80 | 100 | 12.47 | 67.79 | 3.36 | 20.76 | 21.03 | 1.41 | 61.53 |
| 2018-2019 | 2 | 17 | 627399 | 100 | 97.69 | 8.80 | 100 | 12.43 | 70.35 | 1.54 | 22.36 | 22.41 | 1.50 | 62.83 |
| 2018-2019 | 2 | 17 | 623318 | 100 | 47.69 | 5.40 | 52 | 4.89 | 56.28 | 2.35 | 18.26 | 18.41 | 1.44 | 52.56 |
| 2018-2019 | 2 | 17 | 627963 | 100 | 66.67 | 6.40 | 76 | 12.16 | 66.36 | 5.43 | 22.34 | 22.99 | 1.33 | 59.25 |
| 2018-2019 | 2 | 18 | KARAJ1 | 100 | 95.26 | 8.60 | 100 | 13.8 | 72.82 | 3.78 | 23.12 | 23.43 | 1.41 | 64.12 |
| 2018-2019 | 2 | 18 | DEZ | 100 | 83.89 | 7.40 | 92 | 8.39 | 66.12 | 3.36 | 21.47 | 21.73 | 1.42 | 59.75 |
| 2018-2019 | 2 | 18 | 621706 | 100 | 98.35 | 8.60 | 100 | 18.63 | 67.17 | 4.15 | 23.78 | 24.14 | 1.40 | 59.25 |
| 2018-2019 | 2 | 18 | 626358 | 100 | 96.93 | 8.60 | 100 | 16.4 | 68.24 | 4.55 | 23.08 | 23.52 | 1.38 | 60.48 |
| 2018-2019 | 2 | 18 | 623980 | 100 | 95.24 | 8.00 | 100 | 12.27 | 63.34 | 3.94 | 22.68 | 23.02 | 1.40 | 56.71 |
| 2018-2019 | 2 | 18 | 626776 | 100 | 90.69 | 8.00 | 100 | 7.62 | 57.13 | 1.91 | 17.44 | 17.54 | 1.46 | 53.68 |
| 2018-2019 | 2 | 18 | 625127 | 100 | 85.19 | 7.60 | 96 | 12.53 | 60.81 | 1.53 | 21.67 | 21.72 | 1.50 | 55.19 |
| 2018-2019 | 2 | 18 | CHAMRAN2 | 100 | 30.31 | 4.40 | 44 | 14.89 | 60.46 | 4.29 | 23.68 | 24.07 | 1.39 | 53.71 |
| 2018-2019 | 2 | 18 | NICKNEJAD | 80 | 15.21 | 3.20 | 20 | 3.4 | 52.52 | 4.53 | 20.59 | 21.08 | 1.35 | 48.05 |
| 2018-2019 | 2 | 18 | 623503 | 100 | 49.04 | 5.80 | 64 | 10.05 | 60.58 | 3.7 | 20.52 | 20.85 | 1.39 | 55.41 |
| 2018-2019 | 2 | 19 | FRONTANA | 0 | 0 | 1.00 | 0 | 18.37 | 68.78 | 2.35 | 25.7 | 25.81 | 1.48 | 59.49 |
| 2018-2019 | 2 | 19 | 627881 | 100 | 72.97 | 7.00 | 80 | 7.95 | 62.87 | 3.57 | 22.36 | 22.64 | 1.41 | 56.51 |
| 2018-2019 | 2 | 19 | 623377 | 100 | 70.43 | 6.80 | 80 | 15.73 | 72.75 | 1.75 | 23.06 | 23.13 | 1.50 | 64.26 |
| 2018-2019 | 2 | 19 | MV17 | 100 | 94.72 | 8.60 | 100 | 10.46 | 57.79 | 2.67 | 20.2 | 20.38 | 1.44 | 53.13 |
| 2018-2019 | 2 | 19 | 627948 | 100 | 89.57 | 7.80 | 96 | 12.98 | 60.59 | 3.4 | 22.54 | 22.79 | 1.42 | 54.47 |
| 2018-2019 | 2 | 19 | BAHAR | 100 | 88.95 | 7.60 | 100 | 10.91 | 55.58 | 3.14 | 22.8 | 23.02 | 1.43 | 49.97 |
| 2018-2019 | 2 | 19 | 624818 | 100 | 75.8 | 7.00 | 84 | 5.39 | 46.53 | 2.42 | 17.73 | 17.89 | 1.44 | 43.62 |
| 2018-2019 | 2 | 19 | 623345 | 100 | 90.98 | 8.00 | 96 | 14.75 | 69.85 | 4.12 | 22.79 | 23.16 | 1.39 | 61.98 |
| 2018-2019 | 2 | 20 | 627908 | 100 | 99.51 | 9.00 | 100 | 11.34 | 58.44 | 4.73 | 21.57 | 22.08 | 1.35 | 52.94 |
| 2018-2019 | 2 | 20 | 623169 | 100 | 48.32 | 5.60 | 60 | 3.07 | 57.09 | 3.83 | 21.8 | 22.13 | 1.40 | 51.72 |
| 2018-2019 | 2 | 20 | ATRAK | 80 | 13.36 | 3.00 | 16 | 5.39 | 54.49 | 3.88 | 22 | 22.34 | 1.40 | 49.30 |
| 2018-2019 | 2 | 20 | 627356 | 100 | 69.49 | 6.60 | 76 | 10.62 | 65.65 | 2.85 | 22.51 | 22.69 | 1.44 | 58.83 |
| 2018-2019 | 2 | 20 | 626943 | 100 | 65.8 | 6.40 | 80 | 5.19 | 51.63 | 2.43 | 17.44 | 17.61 | 1.43 | 48.52 |
| 2018-2019 | 2 | 20 | 627417 | 100 | 86.05 | 7.60 | 96 | 9.5 | 57.85 | 2.89 | 19.66 | 19.87 | 1.42 | 53.40 |
| 2018-2019 | 2 | 20 | 628088 | 100 | 70.23 | 6.80 | 76 | 13.24 | 65.82 | 2.25 | 21.65 | 21.77 | 1.47 | 59.48 |
| 2018-2019 | 2 | 20 | BAYAT | 100 | 78.95 | 7.00 | 84 | 13.27 | 62.24 | 2.41 | 22.82 | 22.95 | 1.47 | 55.81 |
| 2018-2019 | 2 | 20 | FALAT | 100 | 87.4 | 7.80 | 100 | 13.71 | 56.51 | 4.22 | 23.47 | 23.85 | 1.39 | 50.40 |
| 2018-2019 | 2 | 20 | 626933 | 100 | 92.83 | 8.20 | 100 | 12.55 | 59.04 | 2.73 | 22.49 | 22.66 | 1.45 | 53.19 |
| 2018-2019 | 2 | 21 | 623125 | 100 | 89.63 | 7.80 | 100 | 9.02 | 61.59 | 4.5 | 22.81 | 23.25 | 1.38 | 55.10 |
| 2018-2019 | 2 | 21 | DARAB2 | 100 | 85.59 | 7.80 | 96 | 6.21 | 46.33 | 3.74 | 17.9 | 18.29 | 1.36 | 43.30 |
| 2018-2019 | 2 | 21 | 624980 | 100 | 81.72 | 7.20 | 88 | 11.3 | 59.54 | 4.9 | 23.11 | 23.62 | 1.36 | 53.15 |
| 2018-2019 | 2 | 21 | 625281 | 100 | 20 | 4.00 | 28 | 2.91 | 56.02 | 2.08 | 17.99 | 18.11 | 1.46 | 52.44 |
| 2018-2019 | 2 | 21 | TAJAN | 100 | 96.09 | 8.20 | 100 | 11.87 | 59.68 | 4.21 | 20.24 | 20.67 | 1.37 | 54.69 |
| 2018-2019 | 2 | 21 | 623382 | 100 | 90.6 | 8.00 | 100 | 12.01 | 67.47 | 3.53 | 25.25 | 25.50 | 1.43 | 58.67 |
| 2018-2019 | 2 | 21 | 624580 | 100 | 21.99 | 4.00 | 36 | 1.67 | 49.97 | 3.4 | 19.23 | 19.53 | 1.40 | 46.29 |
| 2018-2019 | 2 | 21 | KAVIR | 100 | 55.96 | 5.60 | 56 | 6.92 | 60.69 | 3.11 | 22.14 | 22.36 | 1.43 | 54.78 |
| 2018-2019 | 2 | 21 | 626883 | 100 | 85 | 7.80 | 84 | 13.23 | 69.88 | 1.06 | 22.57 | 22.59 | 1.52 | 62.35 |
| 2018-2019 | 2 | 21 | BAM | 100 | 38.28 | 5.00 | 48 | 12.85 | 64.79 | 2.5 | 23.38 | 23.51 | 1.46 | 57.66 |
| 2018-2019 | 2 | 22 | 623136 | 100 | 48.81 | 5.60 | 56 | 8.46 | 61.3 | 3.55 | 19.98 | 20.29 | 1.39 | 56.30 |
| 2018-2019 | 2 | 22 | KAVEH | 100 | 85.92 | 8.00 | 92 | 11.75 | 57.78 | 2.65 | 23.6 | 23.75 | 1.46 | 51.56 |
| 2018-2019 | 2 | 22 | 626923 | 80 | 8.06 | 2.80 | 16 | 6.05 | 66.55 | 2.3 | 22.49 | 22.61 | 1.47 | 59.63 |
| 2018-2019 | 2 | 22 | MOGHAN1 | 100 | 30 | 4.80 | 36 | 6.57 | 58.52 | 2.57 | 19.13 | 19.30 | 1.44 | 54.25 |
| 2018-2019 | 2 | 22 | 627099 | 100 | 68.47 | 6.60 | 76 | 8.84 | 53.63 | 1.35 | 17.77 | 17.82 | 1.49 | 50.32 |
| 2018-2019 | 2 | 22 | 626260 | 100 | 15 | 3.80 | 24 | 3.26 | 51.22 | 3.72 | 18.1 | 18.48 | 1.37 | 47.84 |
| 2018-2019 | 2 | 22 | DARYA | 100 | 20.75 | 4.40 | 32 | 11.94 | 54.52 | 4.03 | 21.19 | 21.57 | 1.38 | 49.66 |
| 2018-2019 | 2 | 22 | 623176 | 100 | 10.09 | 3.20 | 20 | 4.79 | 56.23 | 3.16 | 20.21 | 20.46 | 1.42 | 51.69 |
| 2018-2019 | 2 | 22 | 621492 | 100 | 20.48 | 4.00 | 28 | 3.48 | 61.12 | 4.23 | 22.77 | 23.16 | 1.39 | 54.74 |
| 2018-2019 | 2 | 22 | URUOM | 100 | 92.69 | 8.20 | 100 | 13.43 | 64.38 | 3.2 | 21.09 | 21.33 | 1.42 | 58.48 |
| 2018-2019 | 2 | 23 | 627723 | 100 | 37.56 | 5.40 | 48 | 9.54 | 58.79 | 4.45 | 20.85 | 21.32 | 1.36 | 53.60 |
| 2018-2019 | 2 | 23 | BEZOSTAYA | 100 | 17.27 | 3.60 | 24 | 1.04 | 56.08 | 5.46 | 20.32 | 21.04 | 1.31 | 51.30 |
| 2018-2019 | 2 | 23 | 626565 | 100 | 80.32 | 7.20 | 84 | 7.25 | 51.1 | 2.59 | 18.18 | 18.36 | 1.43 | 47.77 |
| 2018-2019 | 2 | 23 | 4820 | 100 | 48.56 | 5.60 | 60 | 11.88 | 66.39 | 2.47 | 22.66 | 22.79 | 1.46 | 59.39 |
| 2018-2019 | 2 | 23 | OFOG | 100 | 22.48 | 3.80 | 32 | 10.02 | 63.58 | 2.78 | 23.1 | 23.27 | 1.45 | 56.78 |
| 2018-2019 | 2 | 23 | 624946 | 60 | 10.68 | 2.60 | 12 | 2.37 | 61.48 | 2.04 | 21.63 | 21.73 | 1.48 | 55.78 |
| 2018-2019 | 2 | 23 | 624894 | 100 | 91.59 | 8.20 | 96 | 6.36 | 52.87 | 1.73 | 16.62 | 16.71 | 1.47 | 50.00 |
| 2018-2019 | 2 | 23 | AZAR1 | 100 | 53.68 | 6.00 | 64 | 4.62 | 49.22 | 2.99 | 18.34 | 18.58 | 1.41 | 45.93 |
| 2018-2019 | 2 | 23 | 626958 | 100 | 28.72 | 4.60 | 36 | 6.84 | 65.12 | 1.58 | 22.16 | 22.22 | 1.50 | 58.65 |
| 2018-2019 | 2 | 23 | 623417 | 100 | 67.95 | 6.60 | 72 | 8.88 | 59.81 | 2.33 | 17.52 | 17.67 | 1.44 | 56.10 |
| 2018-2019 | 2 | 24 | 624378 | 100 | 28.46 | 4.40 | 40 | 3.84 | 56.35 | 3.61 | 19.77 | 20.10 | 1.39 | 51.95 |
| 2018-2019 | 2 | 24 | SISTAN | 100 | 37.02 | 5.00 | 44 | 7.3 | 53.21 | 3.31 | 18.37 | 18.67 | 1.39 | 49.62 |
| 2018-2019 | 2 | 24 | 626872 | 100 | 51.03 | 6.20 | 68 | 6.18 | 50.55 | 3.05 | 15.42 | 15.72 | 1.38 | 48.11 |
| 2018-2019 | 2 | 24 | 624381 | 100 | 35.44 | 5.00 | 40 | 9.85 | 70.33 | 3.36 | 21.98 | 22.24 | 1.42 | 62.92 |
| 2018-2019 | 2 | 24 | 625810 | 100 | 22.22 | 4.00 | 28 | 4.28 | 61.56 | 3.6 | 22.53 | 22.82 | 1.41 | 55.30 |
| 2018-2019 | 2 | 24 | 622311 | 100 | 80.8 | 7.40 | 88 | 7.41 | 61.11 | 3.22 | 18.04 | 18.33 | 1.39 | 57.01 |
| 2018-2019 | 2 | 24 | 627385 | 100 | 72 | 6.60 | 76 | 11.04 | 65.61 | 3.61 | 22.26 | 22.55 | 1.41 | 58.88 |
| 2018-2019 | 2 | 24 | 623905 | 100 | 20.64 | 4.00 | 36 | 4.06 | 49.77 | 3.66 | 18.69 | 19.04 | 1.38 | 46.28 |
| 2018-2019 | 2 | 24 | 627460 | 100 | 97.6 | 8.80 | 100 | 12.8 | 66.1 | 4.03 | 21.74 | 22.11 | 1.39 | 59.53 |
| 2018-2019 | 2 | 25 | 627760 | 100 | 37.07 | 4.80 | 44 | 6.61 | 69.66 | 2.35 | 25.02 | 25.13 | 1.48 | 60.60 |
| 2018-2019 | 2 | 25 | 625362 | 20 | 10.56 | 2.00 | 12 | 8.04 | 54.46 | 3.01 | 24.4 | 24.58 | 1.45 | 48.25 |
| 2018-2019 | 2 | 25 | 623274 | 100 | 91.41 | 8.40 | 92 | 11.04 | 65.29 | 3.09 | 21.1 | 21.33 | 1.43 | 59.26 |
| 2018-2019 | 2 | 25 | 626736 | 100 | 47.37 | 5.60 | 56 | 8.81 | 57.28 | 2.54 | 21.18 | 21.33 | 1.45 | 52.25 |
| 2018-2019 | 2 | 25 | 624585 | 60 | 13.74 | 2.60 | 20 | 1.46 | 48.92 | 4.13 | 19.26 | 19.70 | 1.36 | 45.25 |
| 2018-2019 | 2 | 25 | KOOHDASHT | 100 | 45.04 | 5.40 | 52 | 14.35 | 63.69 | 1.82 | 26 | 26.06 | 1.50 | 55.30 |
| 2018-2019 | 2 | 25 | NAVID | 100 | 61.26 | 6.20 | 72 | 13.07 | 68.32 | 1.49 | 22.94 | 22.99 | 1.51 | 60.86 |
| 2018-2019 | 2 | 25 | KARAJ2 | 100 | 100 | 9.00 | 100 | 15 | 65.25 | 1.2 | 22.83 | 22.86 | 1.52 | 58.40 |
| 2018-2019 | 2 | 25 | CHAMRAN | 100 | 92.98 | 8.20 | 100 | 0.13 | 52.06 | 1.51 | 16.25 | 16.32 | 1.48 | 49.36 |
| 2018-2019 | 2 | 25 | 624864 | 100 | 19.54 | 3.80 | 28 | 6.42 | 63.75 | 3.76 | 21.09 | 21.42 | 1.39 | 57.89 |
| 2018-2019 | 2 | 26 | 625123 | 100 | 100 | 9.00 | 100 | 8.97 | 57.51 | 2.61 | 20.68 | 20.84 | 1.45 | 52.67 |
| 2018-2019 | 2 | 26 | CASGOGEN | 0 | 0 | 1.00 | 0 | 7.67 | 50.99 | 2.69 | 18.02 | 18.22 | 1.42 | 47.71 |
| 2018-2019 | 2 | 26 | 626764 | 100 | 100 | 9.00 | 100 | 5.55 | 52.32 | 3.09 | 16.76 | 17.04 | 1.39 | 49.37 |
| 2018-2019 | 2 | 26 | 624804 | 100 | 33.65 | 4.80 | 44 | 4.43 | 55.92 | 3.63 | 19.7 | 20.03 | 1.39 | 51.58 |
| 2018-2019 | 2 | 26 | 626846 | 100 | 65.79 | 6.20 | 76 | 7.04 | 50.65 | 3.54 | 16.2 | 16.58 | 1.36 | 47.94 |
| 2018-2019 | 2 | 26 | ZARRIN | 100 | 29.79 | 4.20 | 40 | 4.08 | 49.72 | 4.65 | 21.09 | 21.60 | 1.35 | 45.28 |
| 2018-2019 | 2 | 26 | 626814 | 100 | 57.87 | 6.00 | 72 | 8.86 | 57.06 | 2.44 | 21.64 | 21.78 | 1.46 | 51.85 |
| 2018-2019 | 2 | 26 | 626825 | 100 | 97.87 | 8.60 | 96 | 10.68 | 60.53 | 2.57 | 22.07 | 22.22 | 1.45 | 54.71 |
| 2018-2019 | 2 | 26 | DEYHEM | 100 | 56.78 | 6.20 | 64 | 8.42 | 52.96 | 3.55 | 17.15 | 17.51 | 1.37 | 49.81 |
| 2018-2019 | 2 | 26 | 624251 | 100 | 20.18 | 3.80 | 24 | 5.84 | 56.54 | 2.76 | 20.64 | 20.82 | 1.44 | 51.81 |
| 2018-2019 | 2 | 27 | 623953 | 0 | 0 | 1.00 | 0 | 0.2 | 67.7 | 2.13 | 24.84 | 24.93 | 1.49 | 59.20 |
| 2018-2019 | 2 | 27 | 626855 | 100 | 89.44 | 8.20 | 96 | 15.41 | 65.14 | 2.52 | 25.06 | 25.19 | 1.47 | 56.99 |
| 2018-2019 | 2 | 27 | 623008 | 100 | 73.33 | 7.00 | 80 | 13 | 59.64 | 2.75 | 21.59 | 21.76 | 1.44 | 54.15 |
| 2018-2019 | 2 | 27 | 621669 | 100 | 89.01 | 7.80 | 96 | 10.94 | 62.27 | 4.66 | 20.48 | 21.00 | 1.35 | 56.82 |
| 2018-2019 | 2 | 27 | SHARYAR | 100 | 95.02 | 8.60 | 96 | 11.86 | 63.83 | 4.74 | 22.87 | 23.36 | 1.37 | 56.94 |
| 2018-2019 | 2 | 27 | ADL | 100 | 100 | 9.00 | 100 | 10.63 | 60.03 | 2.04 | 18.21 | 18.32 | 1.46 | 56.03 |
| 2018-2019 | 2 | 27 | PANJAMO62 | 100 | 80.63 | 7.40 | 88 | 14.07 | 69.26 | 3.23 | 23.69 | 23.91 | 1.44 | 61.06 |
| 2018-2019 | 2 | 27 | 624939 | 100 | 92 | 8.20 | 100 | 11.99 | 65.46 | 4.02 | 19.83 | 20.23 | 1.37 | 59.97 |
| 2018-2019 | 2 | 27 | PISHGAM | 100 | 70.59 | 6.40 | 80 | 12.01 | 69.82 | 4.14 | 24.03 | 24.38 | 1.40 | 61.20 |
| 2018-2019 | 2 | 27 | 626360 | 100 | 84.64 | 7.60 | 96 | 8.98 | 61.34 | 4.3 | 22.23 | 22.64 | 1.38 | 55.20 |
| 2018-2019 | 2 | 28 | 624983 | 100 | 67.32 | 7.00 | 76 | 7.6 | 60.44 | 5.05 | 22.42 | 22.98 | 1.35 | 54.25 |
| 2018-2019 | 2 | 28 | SIVAND | 100 | 88.84 | 8.00 | 96 | 13.47 | 59.96 | 4.5 | 23.76 | 24.18 | 1.38 | 53.22 |
| 2018-2019 | 2 | 28 | 627551 | 100 | 10.12 | 2.80 | 20 | 0.53 | 44.27 | 4.63 | 18.56 | 19.13 | 1.33 | 41.08 |
| 2018-2019 | 2 | 28 | SHAHI | 100 | 92.04 | 8.20 | 100 | 10.66 | 58.05 | 4.53 | 21.85 | 22.31 | 1.37 | 52.48 |
| 2018-2019 | 2 | 28 | 621717 | 100 | 27.91 | 4.60 | 40 | 3.65 | 62.21 | 2.29 | 24.17 | 24.28 | 1.48 | 55.08 |
| 2018-2019 | 2 | 28 | ARVAND | 100 | 89.22 | 8.20 | 96 | 8.56 | 57.54 | 2.21 | 18.98 | 19.11 | 1.45 | 53.44 |
| 2018-2019 | 2 | 28 | MORVARID | 100 | 96.23 | 8.60 | 100 | 10.24 | 60.16 | 2.21 | 24.27 | 24.37 | 1.48 | 53.30 |
| 2018-2019 | 2 | 28 | 624863 | 100 | 20.71 | 4.00 | 24 | 2.42 | 64.12 | 2.28 | 22.43 | 22.55 | 1.47 | 57.62 |
| 2018-2019 | 2 | 28 | 626881 | 100 | 72.97 | 7.00 | 80 | 9.21 | 65.01 | 3.58 | 23.32 | 23.59 | 1.42 | 57.80 |
| 2018-2019 | 2 | 28 | TOUBARI | 100 | 78.57 | 7.20 | 84 | 7.17 | 59.97 | 1.96 | 20.18 | 20.27 | 1.47 | 55.13 |
| 2018-2019 | 2 | 29 | SHINGHAI | 100 | 55.75 | 6.00 | 60 | 11.04 | 53.17 | 4.97 | 19.53 | 20.15 | 1.32 | 49.02 |
| 2018-2019 | 2 | 29 | KARAJ3 | 100 | 86.86 | 7.80 | 92 | 7.94 | 60.56 | 4.81 | 20.59 | 21.14 | 1.34 | 55.25 |
| 2018-2019 | 2 | 29 | 624947 | 100 | 63.6 | 6.20 | 68 | 8.13 | 55 | 4.55 | 19.63 | 20.15 | 1.34 | 50.69 |
| 2018-2019 | 2 | 29 | GOLESTAN | 100 | 88.36 | 7.60 | 100 | 9.4 | 62.98 | 3.12 | 19.19 | 19.44 | 1.41 | 58.19 |
| 2018-2019 | 2 | 29 | 624963 | 100 | 50.56 | 5.60 | 64 | 5.91 | 60.4 | 3.91 | 20.15 | 20.53 | 1.38 | 55.40 |
| 2018-2019 | 2 | 29 | AZADI | 100 | 88.43 | 8.00 | 100 | 11.28 | 55.63 | 3 | 20.2 | 20.42 | 1.42 | 51.16 |
| 2018-2019 | 2 | 29 | 623123 | 100 | 82.86 | 7.60 | 92 | 15.37 | 61.18 | 4.35 | 24.6 | 24.98 | 1.40 | 53.84 |
| 2018-2019 | 2 | 29 | AFLAK | 100 | 89.02 | 7.80 | 100 | 16.3 | 64.76 | 1.91 | 24.61 | 24.68 | 1.49 | 56.97 |
| 2018-2019 | 2 | 29 | KHAZAR1 | 100 | 44.57 | 5.60 | 52 | 9.68 | 61.75 | 1.58 | 22.28 | 22.34 | 1.50 | 55.71 |
| 2018-2019 | 2 | 30 | 623069 | 80 | 35.91 | 4.60 | 40 | 10.12 | 68.61 | 2.6 | 23.08 | 23.23 | 1.46 | 60.95 |
| 2018-2019 | 2 | 30 | ZAGROS | 100 | 58.26 | 6.20 | 68 | 9.41 | 58.91 | 1.92 | 20.89 | 20.98 | 1.48 | 53.86 |
| 2018-2019 | 2 | 30 | 624901 | 100 | 34.18 | 5.00 | 40 | 4.55 | 54.76 | 2.37 | 18.59 | 18.74 | 1.44 | 51.03 |
| 2018-2019 | 2 | 30 | SISON | 60 | 9.3 | 2.40 | 16 | 10.1 | 67.55 | 2.73 | 23.41 | 23.57 | 1.45 | 59.89 |
| 2018-2019 | 2 | 30 | 624215 | 60 | 7.29 | 2.20 | 12 | 0.39 | 50.02 | 1.91 | 17.52 | 17.62 | 1.46 | 47.00 |
| 2018-2019 | 2 | 30 | GHODS | 100 | 67.82 | 6.60 | 76 | 10.73 | 57.38 | 3.48 | 20.12 | 20.42 | 1.40 | 52.74 |
| 2018-2019 | 2 | 30 | 624861 | 100 | 68.42 | 6.80 | 76 | 9.63 | 68.01 | 2.89 | 21.88 | 22.07 | 1.44 | 61.14 |
| 2018-2019 | 2 | 30 | RIJAW | 100 | 84.68 | 7.40 | 92 | 14.72 | 65.47 | 3.9 | 23.14 | 23.47 | 1.40 | 58.25 |
| 2018-2019 | 2 | 30 | SPAHAN | 100 | 79.46 | 7.40 | 88 | 9.34 | 57.74 | 2.68 | 21.2 | 21.37 | 1.45 | 52.64 |
| 2018-2019 | 2 | 31 | 627055 | 100 | 74.06 | 7.00 | 80 | 5.85 | 59.7 | 3.86 | 19.48 | 19.86 | 1.38 | 55.07 |
| 2018-2019 | 2 | 31 | 626932 | 100 | 64.96 | 6.60 | 68 | 8.71 | 71.15 | 4.01 | 23.26 | 23.60 | 1.40 | 62.72 |
| 2018-2019 | 2 | 31 | PARSI | 100 | 100 | 9.00 | 100 | 14.51 | 65.85 | 4.28 | 20.86 | 21.29 | 1.37 | 59.75 |
| 2018-2019 | 2 | 31 | 624838 | 100 | 79.65 | 7.00 | 88 | 6.04 | 65.39 | 4.64 | 22.4 | 22.88 | 1.37 | 58.51 |
| 2018-2019 | 2 | 31 | 621736 | 80 | 16.92 | 3.40 | 20 | 4.15 | 64.38 | 2.34 | 23.74 | 23.86 | 1.47 | 57.13 |
| 2018-2019 | 2 | 31 | 621869 | 100 | 58.2 | 5.80 | 64 | 6.03 | 68.16 | 2.13 | 21.56 | 21.66 | 1.47 | 61.49 |
| 2018-2019 | 2 | 31 | 628012 | 80 | 13.39 | 3.00 | 20 | 1.43 | 55.05 | 2.76 | 18.28 | 18.49 | 1.42 | 51.40 |
| 2018-2019 | 2 | 31 | 622099 | 0 | 0 | 1.00 | 0 | 0.11 | 52.55 | 1.5 | 17.46 | 17.52 | 1.49 | 49.42 |
| 2018-2019 | 2 | 31 | 622105 | 0 | 0 | 1.00 | 0 | 0.13 | 47.65 | 5.78 | 19.72 | 20.55 | 1.29 | 43.76 |
| 2018-2019 | 2 | 32 | 621420 | 100 | 62.05 | 6.20 | 68 | 4.65 | 56.63 | 2.87 | 22.54 | 22.72 | 1.44 | 51.04 |
| 2018-2019 | 2 | 32 | 623266 | 60 | 21.86 | 3.00 | 20 | 1.7 | 52.98 | 2.28 | 19.12 | 19.26 | 1.45 | 49.19 |
| 2018-2019 | 2 | 32 | 621712 | 100 | 60.18 | 6.20 | 72 | 6.46 | 49.7 | 4.74 | 17.52 | 18.15 | 1.31 | 46.53 |
| 2018-2019 | 2 | 32 | 620903 | 100 | 100 | 9.00 | 100 | 5.28 | 51.12 | 1.58 | 16.63 | 16.70 | 1.48 | 48.34 |
| 2018-2019 | 2 | 32 | 625139 | 100 | 43.05 | 5.20 | 56 | 2.69 | 53.34 | 2.45 | 15.59 | 15.78 | 1.41 | 50.74 |
| 2018-2019 | 2 | 32 | 622098 | 100 | 82.8 | 7.60 | 88 | 11.61 | 65.85 | 4.26 | 22.3 | 22.70 | 1.38 | 58.99 |
| 2018-2019 | 2 | 32 | 625263 | 0 | 0 | 1.00 | 0 | 0.31 | 55.68 | 1.45 | 18.47 | 18.53 | 1.49 | 51.96 |
| 2018-2019 | 2 | 32 | 622264 | 0 | 0 | 1.00 | 0 | 0.11 | 49.96 | 4.82 | 19.34 | 19.93 | 1.33 | 46.14 |
| 2018-2019 | 2 | 32 | 622272 | 0 | 0 | 1.00 | 0 | 0.11 | 53.01 | 2.46 | 18.15 | 18.32 | 1.44 | 49.57 |
| 2018-2019 | 2 | 32 | 627905 | 80 | 17.67 | 3.40 | 24 | 0.9 | 54.96 | 1.15 | 15.64 | 15.68 | 1.50 | 52.31 |
| 2019-2020 | 1 | 1 | 627036 | 100 | 99.31 | 9.00 | 100 | 15.24 | 64.89 | 3.55 | 23.62 | 23.89 | 1.42 | 57.54 |
| 2019-2020 | 1 | 1 | 626156 | 100 | 82.42 | 7.67 | 93 | 8.04 | 55.62 | 3.67 | 19.95 | 20.28 | 1.39 | 51.20 |
| 2019-2020 | 1 | 1 | ROSHAN | 100 | 90.48 | 8.00 | 100 | 9.51 | 54.27 | 4.39 | 22.19 | 22.62 | 1.38 | 48.98 |
| 2019-2020 | 1 | 1 | 627852 | 100 | 90.21 | 7.67 | 100 | 16.65 | 62.75 | 3.73 | 23.45 | 23.74 | 1.41 | 55.83 |
| 2019-2020 | 1 | 1 | 628114 | 100 | 95.59 | 8.33 | 100 | 13.61 | 64.06 | 3.36 | 23.21 | 23.45 | 1.43 | 57.09 |
| 2019-2020 | 1 | 1 | 624985 | 100 | 89.44 | 7.67 | 100 | 10.5 | 55.14 | 5.38 | 21.44 | 22.10 | 1.32 | 49.99 |
| 2019-2020 | 1 | 1 | 624837 | 100 | 89.31 | 8.00 | 100 | 13.19 | 69.11 | 3.37 | 23.65 | 23.89 | 1.43 | 60.95 |
| 2019-2020 | 1 | 1 | TOUS | 100 | 92.7 | 8.33 | 100 | 7 | 54.66 | 4.2 | 21.44 | 21.85 | 1.38 | 49.67 |
| 2019-2020 | 1 | 1 | 623473 | 100 | 90.1 | 7.67 | 100 | 8 | 55.61 | 4.13 | 21.18 | 21.58 | 1.38 | 50.64 |
| 2019-2020 | 1 | 2 | MOGHAN2 | 100 | 93.51 | 8.67 | 100 | 12.58 | 61.62 | 4.07 | 23.94 | 24.28 | 1.40 | 54.58 |
| 2019-2020 | 1 | 2 | 627359 | 100 | 91.89 | 8.00 | 100 | 10.05 | 54.97 | 3.95 | 20.41 | 20.79 | 1.38 | 50.40 |
| 2019-2020 | 1 | 2 | 627038 | 100 | 95.95 | 8.67 | 100 | 13.3 | 62.8 | 5.18 | 21.94 | 22.54 | 1.34 | 56.50 |
| 2019-2020 | 1 | 2 | 621704 | 100 | 50 | 5.67 | 53 | 4.62 | 45.66 | 1.5 | 17.07 | 17.14 | 1.48 | 43.02 |
| 2019-2020 | 1 | 2 | 626566 | 100 | 57.02 | 6.00 | 67 | 8.24 | 52.89 | 4.33 | 19.7 | 20.17 | 1.35 | 48.75 |
| 2019-2020 | 1 | 2 | SHAHPASSAND | 100 | 81.63 | 7.33 | 93 | 18 | 68.64 | 3.88 | 23.76 | 24.07 | 1.41 | 60.46 |
| 2019-2020 | 1 | 2 | 623508 | 100 | 65.77 | 6.33 | 80 | 12.33 | 61.28 | 4.13 | 23.53 | 23.89 | 1.40 | 54.50 |
| 2019-2020 | 1 | 2 | 621735 | 100 | 81.4 | 7.00 | 93 | 9.55 | 51.26 | 1.97 | 19.41 | 19.51 | 1.47 | 47.50 |
| 2019-2020 | 1 | 2 | 623338 | 100 | 92.54 | 8.33 | 100 | 12.81 | 59.19 | 3.07 | 24.23 | 24.42 | 1.44 | 52.44 |
| 2019-2020 | 1 | 2 | 621668 | 100 | 89.87 | 8.00 | 100 | 8.18 | 50.77 | 3.32 | 17.52 | 17.83 | 1.38 | 47.64 |
| 2019-2020 | 1 | 3 | 621421 | 100 | 90.38 | 8.00 | 100 | 9.14 | 56.23 | 1.51 | 16.8 | 16.87 | 1.48 | 53.09 |
| 2019-2020 | 1 | 3 | 624240 | 100 | 95.12 | 8.33 | 100 | 10.33 | 55.8 | 4.03 | 20.89 | 21.28 | 1.38 | 50.95 |
| 2019-2020 | 1 | 3 | 623344 | 100 | 90.32 | 7.67 | 100 | 13.64 | 64.42 | 3.75 | 23.19 | 23.49 | 1.41 | 57.36 |
| 2019-2020 | 1 | 3 | 624941 | 0 | 0 | 1.00 | 0 | 0.16 | 55.8 | 3.98 | 17.31 | 17.76 | 1.34 | 52.36 |
| 2019-2020 | 1 | 3 | 621716 | 100 | 77.42 | 6.67 | 93 | 14.01 | 63.91 | 6.91 | 24.85 | 25.79 | 1.30 | 55.64 |
| 2019-2020 | 1 | 3 | 627236 | 100 | 87.66 | 7.67 | 100 | 13.17 | 66.24 | 4.26 | 22.88 | 23.27 | 1.39 | 59.00 |
| 2019-2020 | 1 | 3 | 624911 | 100 | 77.42 | 7.00 | 87 | 12.84 | 64.82 | 2.36 | 20.88 | 21.01 | 1.46 | 59.02 |
| 2019-2020 | 1 | 4 | OHADI | 100 | 74.34 | 6.67 | 87 | 11.26 | 71.36 | 4.67 | 21.72 | 22.22 | 1.36 | 63.75 |
| 2019-2020 | 1 | 4 | 624990 | 100 | 84.44 | 7.33 | 93 | 8.88 | 64.41 | 4 | 23.22 | 23.56 | 1.40 | 57.32 |
| 2019-2020 | 1 | 4 | 627484 | 100 | 45.45 | 5.67 | 53 | 10.78 | 61.58 | 6.09 | 21.18 | 22.04 | 1.29 | 55.71 |
| 2019-2020 | 1 | 4 | 627688 | 100 | 88.24 | 7.67 | 93 | 6.93 | 48.2 | 2.49 | 16.88 | 17.06 | 1.42 | 45.46 |
| 2019-2020 | 1 | 4 | 627416 | 100 | 93.29 | 8.33 | 100 | 13.92 | 74.53 | 3.81 | 22.38 | 22.70 | 1.40 | 65.88 |
| 2019-2020 | 1 | 4 | 625661 | 100 | 92.65 | 8.00 | 100 | 9.15 | 49.77 | 1.57 | 16.48 | 16.55 | 1.48 | 47.11 |
| 2019-2020 | 1 | 4 | 625081 | 100 | 54.11 | 6.00 | 67 | 9.75 | 59.81 | 4.32 | 22.5 | 22.91 | 1.38 | 53.74 |
| 2019-2020 | 1 | 4 | 627061 | 100 | 93.33 | 8.33 | 100 | 12 | 62.54 | 3.58 | 21.7 | 21.99 | 1.41 | 56.56 |
| 2019-2020 | 1 | 4 | NEISHABOUR | 100 | 94.79 | 8.67 | 100 | 10.13 | 69.02 | 4.29 | 23.64 | 24.03 | 1.39 | 60.80 |
| 2019-2020 | 1 | 4 | 624315 | 100 | 90.57 | 8.00 | 100 | 14.25 | 68.14 | 3.87 | 24.01 | 24.32 | 1.41 | 59.92 |
| 2019-2020 | 1 | 5 | 627103 | 20 | 0.52 | 1.33 | 7 | 0.7 | 49.48 | 1.97 | 16.57 | 16.69 | 1.45 | 46.80 |
| 2019-2020 | 1 | 5 | 623475 | 100 | 80.71 | 7.00 | 93 | 8.87 | 58.05 | 3.34 | 19.2 | 19.49 | 1.40 | 53.74 |
| 2019-2020 | 1 | 5 | 624596 | 100 | 88.57 | 7.67 | 93 | 8.9 | 56.94 | 4.27 | 22.04 | 22.45 | 1.38 | 51.44 |
| 2019-2020 | 1 | 5 | AKBARI | 100 | 61.22 | 6.00 | 67 | 7.65 | 59.93 | 3.71 | 20.4 | 20.73 | 1.39 | 54.88 |
| 2019-2020 | 1 | 5 | 623506 | 100 | 58.93 | 6.33 | 67 | 12.67 | 69.49 | 5.62 | 21.65 | 22.37 | 1.32 | 62.17 |
| 2019-2020 | 1 | 5 | 623510 | 100 | 75.28 | 7.00 | 80 | 9.9 | 51.92 | 4.79 | 19.93 | 20.50 | 1.33 | 47.73 |
| 2019-2020 | 1 | 5 | 627845 | 100 | 93.09 | 8.00 | 100 | 10.56 | 59.01 | 3.93 | 19.48 | 19.87 | 1.37 | 54.45 |
| 2019-2020 | 1 | 6 | 627856 | 20 | 0.99 | 1.33 | 7 | 0.23 | 50.15 | 1.93 | 18.16 | 18.26 | 1.46 | 46.91 |
| 2019-2020 | 1 | 6 | MAHDAVI | 100 | 89.71 | 8.00 | 100 | 13.65 | 67.69 | 3.8 | 24.04 | 24.34 | 1.41 | 59.55 |
| 2019-2020 | 1 | 6 | 627360 | 100 | 96.09 | 8.67 | 100 | 18.54 | 67.06 | 3.32 | 23.92 | 24.15 | 1.43 | 59.16 |
| 2019-2020 | 1 | 6 | 627883 | 100 | 87.5 | 8.00 | 93 | 12.51 | 69.33 | 3.97 | 23.04 | 23.38 | 1.40 | 61.44 |
| 2019-2020 | 1 | 6 | SHIROODI | 100 | 95.31 | 8.67 | 100 | 14.79 | 67.51 | 1.1 | 21.81 | 21.84 | 1.52 | 60.85 |
| 2019-2020 | 1 | 6 | 623379 | 100 | 67.19 | 6.67 | 73 | 9.41 | 56.33 | 5.17 | 24.22 | 24.77 | 1.36 | 49.80 |
| 2019-2020 | 1 | 6 | 624910 | 100 | 87.78 | 7.67 | 100 | 16.3 | 63.73 | 5.19 | 23.14 | 23.71 | 1.35 | 56.67 |
| 2019-2020 | 1 | 6 | MOGHAN3 | 100 | 95.45 | 8.33 | 100 | 17.29 | 61.69 | 4.1 | 25.63 | 25.96 | 1.41 | 53.73 |
| 2019-2020 | 1 | 6 | DASTJERDI | 100 | 95.48 | 8.33 | 100 | 3.99 | 66.61 | 3.51 | 21.62 | 21.90 | 1.41 | 60.07 |
| 2019-2020 | 1 | 7 | TAKAB | 60 | 4.5 | 2.00 | 13 | 0.9 | 50.11 | 4.02 | 17.43 | 17.89 | 1.34 | 47.00 |
| 2019-2020 | 1 | 7 | 623908 | 100 | 94.12 | 8.33 | 100 | 11.54 | 63.06 | 3.28 | 18.96 | 19.24 | 1.40 | 58.35 |
| 2019-2020 | 1 | 7 | RASHID | 100 | 96.55 | 8.33 | 100 | 14.68 | 70.62 | 3.71 | 21.58 | 21.90 | 1.40 | 63.36 |
| 2019-2020 | 1 | 7 | 624900 | 100 | 92.54 | 8.33 | 100 | 12.94 | 67.43 | 5.56 | 24.21 | 24.84 | 1.35 | 59.04 |
| 2019-2020 | 1 | 7 | 627054 | 100 | 93.66 | 8.00 | 100 | 11.43 | 55.14 | 5.96 | 23.76 | 24.50 | 1.33 | 48.89 |
| 2019-2020 | 1 | 7 | 626223 | 100 | 97.3 | 8.67 | 100 | 14.66 | 65.75 | 3.7 | 22.11 | 22.42 | 1.40 | 59.07 |
| 2019-2020 | 1 | 7 | 627189 | 100 | 96.39 | 8.67 | 100 | 12.88 | 68.43 | 2.88 | 22.07 | 22.26 | 1.44 | 61.37 |
| 2019-2020 | 1 | 7 | 627423 | 100 | 95.35 | 8.33 | 100 | 15.6 | 64.27 | 5.87 | 26.1 | 26.75 | 1.35 | 55.36 |
| 2019-2020 | 1 | 7 | ZARE | 100 | 98.18 | 9.00 | 100 | 11.06 | 62.89 | 4.14 | 20.72 | 21.13 | 1.37 | 57.30 |
| 2019-2020 | 1 | 8 | 627410 | 100 | 57.03 | 5.67 | 67 | 8.45 | 53.65 | 4.58 | 18.35 | 18.91 | 1.33 | 49.94 |
| 2019-2020 | 1 | 8 | DN11 | 100 | 97.95 | 9.00 | 100 | 11.99 | 66.53 | 3.27 | 24.13 | 24.35 | 1.44 | 58.61 |
| 2019-2020 | 1 | 8 | 625080 | 100 | 94.86 | 8.67 | 100 | 14.38 | 61.46 | 4.66 | 22.57 | 23.05 | 1.37 | 55.10 |
| 2019-2020 | 1 | 8 | HOMA | 100 | 78.15 | 6.67 | 93 | 13.56 | 71.82 | 3.93 | 23.54 | 23.87 | 1.41 | 63.07 |
| 2019-2020 | 1 | 8 | 627842 | 100 | 93.07 | 8.00 | 100 | 12.52 | 60.9 | 3.88 | 20.61 | 20.97 | 1.38 | 55.63 |
| 2019-2020 | 1 | 8 | 627043 | 100 | 39.88 | 5.00 | 47 | 7.01 | 58.47 | 1.95 | 18.03 | 18.14 | 1.46 | 54.68 |
| 2019-2020 | 1 | 8 | 624956 | 100 | 88.46 | 7.67 | 100 | 13.28 | 66.91 | 5.44 | 24.65 | 25.24 | 1.35 | 58.38 |
| 2019-2020 | 1 | 8 | 626261 | 100 | 99.31 | 9.00 | 100 | 13.66 | 56.29 | 3.73 | 22.86 | 23.16 | 1.41 | 50.53 |
| 2019-2020 | 1 | 8 | 628084 | 100 | 96.11 | 8.67 | 100 | 7.56 | 51.47 | 3.11 | 16.98 | 17.26 | 1.39 | 48.49 |
| 2019-2020 | 1 | 8 | VEE/NAC | 100 | 98.69 | 9.00 | 100 | 10.18 | 53.25 | 4.89 | 22.05 | 22.59 | 1.35 | 48.08 |
| 2019-2020 | 1 | 9 | 625047 | 100 | 96.32 | 8.67 | 100 | 13.6 | 64.76 | 3.96 | 22.75 | 23.09 | 1.40 | 57.87 |
| 2019-2020 | 1 | 9 | 626158 | 100 | 96.89 | 8.67 | 100 | 7.79 | 54.52 | 1.79 | 18.78 | 18.87 | 1.48 | 50.76 |
| 2019-2020 | 1 | 9 | 624925 | 100 | 97.39 | 9.00 | 100 | 18.13 | 65.33 | 2.53 | 24.51 | 24.64 | 1.47 | 57.47 |
| 2019-2020 | 1 | 9 | 621619 | 100 | 88.99 | 8.00 | 100 | 10.76 | 60.33 | 2.12 | 19.86 | 19.97 | 1.46 | 55.59 |
| 2019-2020 | 1 | 9 | 627299 | 100 | 97.08 | 9.00 | 100 | 14.31 | 65.8 | 2.84 | 25.82 | 25.98 | 1.46 | 57.05 |
| 2019-2020 | 1 | 9 | 623291 | 100 | 97.22 | 8.67 | 100 | 8.14 | 48.65 | 3.58 | 19.08 | 19.41 | 1.39 | 45.10 |
| 2019-2020 | 1 | 9 | 627849 | 100 | 86.22 | 7.33 | 100 | 13.87 | 59.89 | 2.13 | 22.84 | 22.94 | 1.48 | 53.79 |
| 2019-2020 | 1 | 9 | 626978 | 100 | 94.21 | 8.33 | 100 | 14.02 | 62.64 | 3.23 | 23.23 | 23.45 | 1.43 | 55.89 |
| 2019-2020 | 1 | 9 | AKOVA | 100 | 93.65 | 8.33 | 100 | 10.55 | 52.47 | 5.26 | 20.98 | 21.63 | 1.33 | 47.78 |
| 2019-2020 | 1 | 9 | 627072 | 100 | 99.36 | 9.00 | 100 | 13.78 | 63.32 | 3.25 | 21.99 | 22.23 | 1.42 | 57.11 |
| 2019-2020 | 1 | 10 | 626908 | 100 | 97.58 | 9.00 | 100 | 12.88 | 56.49 | 5.16 | 21.84 | 22.44 | 1.34 | 51.04 |
| 2019-2020 | 1 | 10 | DARAB1 | 100 | 87.17 | 8.00 | 93 | 7.2 | 53.1 | 3.02 | 22.95 | 23.15 | 1.44 | 47.70 |
| 2019-2020 | 1 | 10 | 623421 | 100 | 95.4 | 8.33 | 100 | 9.83 | 58.83 | 3.71 | 19.84 | 20.18 | 1.39 | 54.15 |
| 2019-2020 | 1 | 10 | 623090 | 100 | 94.19 | 8.67 | 100 | 11.04 | 67.37 | 2 | 22.14 | 22.23 | 1.48 | 60.52 |
| 2019-2020 | 1 | 10 | 626234 | 100 | 96.9 | 8.67 | 100 | 8.29 | 50.4 | 4.41 | 18.09 | 18.62 | 1.33 | 47.02 |
| 2019-2020 | 1 | 10 | 627853 | 100 | 100 | 9.00 | 100 | 9.93 | 56.21 | 3.14 | 21.69 | 21.92 | 1.43 | 51.03 |
| 2019-2020 | 1 | 10 | GHABOUS | 100 | 96.86 | 9.00 | 100 | 11.32 | 55.45 | 2.03 | 20.7 | 20.80 | 1.47 | 50.83 |
| 2019-2020 | 1 | 10 | 624805 | 20 | 3.45 | 1.67 | 7 | 0.47 | 50.01 | 3.58 | 19.51 | 19.84 | 1.39 | 46.22 |
| 2019-2020 | 1 | 10 | 621565 | 100 | 78.86 | 7.00 | 87 | 10.63 | 56.54 | 3.01 | 23.85 | 24.04 | 1.45 | 50.33 |
| 2019-2020 | 1 | 10 | SABALAN | 100 | 84.35 | 7.33 | 93 | 14.84 | 64.76 | 2.2 | 23.97 | 24.07 | 1.48 | 57.32 |
| 2019-2020 | 1 | 11 | 626706 | 100 | 92.8 | 8.33 | 100 | 12.73 | 68.43 | 3.95 | 22.71 | 23.05 | 1.40 | 60.91 |
| 2019-2020 | 1 | 11 | 622063 | 100 | 87.5 | 7.67 | 100 | 7.11 | 57.78 | 2.04 | 18.54 | 18.65 | 1.46 | 53.84 |
| 2019-2020 | 1 | 11 | 626747 | 100 | 93.88 | 8.33 | 100 | 12.68 | 66.31 | 2.45 | 24.22 | 24.34 | 1.47 | 58.44 |
| 2019-2020 | 1 | 11 | 626573 | 100 | 99.39 | 9.00 | 100 | 15.36 | 66.66 | 3.92 | 25.65 | 25.95 | 1.42 | 57.75 |
| 2019-2020 | 1 | 11 | 622247 | 0 | 0 | 1.00 | 0 | 0.2 | 50.87 | 4.07 | 18.77 | 19.21 | 1.36 | 47.25 |
| 2019-2020 | 1 | 11 | 627990 | 100 | 88.54 | 7.67 | 100 | 15.43 | 66.69 | 5.4 | 23.09 | 23.71 | 1.34 | 59.11 |
| 2019-2020 | 1 | 11 | MAROON | 100 | 98.94 | 9.00 | 100 | 11.78 | 54.1 | 4.23 | 20.21 | 20.65 | 1.36 | 49.67 |
| 2019-2020 | 1 | 11 | 626226 | 100 | 99.31 | 9.00 | 100 | 13.97 | 65.25 | 4.14 | 22.26 | 22.64 | 1.39 | 58.52 |
| 2019-2020 | 1 | 11 | 626924 | 100 | 80.13 | 7.33 | 87 | 9.19 | 63.72 | 4.77 | 24.11 | 24.58 | 1.38 | 56.18 |
| 2019-2020 | 1 | 11 | 622084 | 0 | 0 | 1.00 | 0 | 0.36 | 48.97 | 1.88 | 16.66 | 16.77 | 1.46 | 46.29 |
| 2019-2020 | 1 | 12 | 624849 | 100 | 70.56 | 7.00 | 80 | 9.38 | 57.81 | 3.68 | 19.96 | 20.30 | 1.39 | 53.18 |
| 2019-2020 | 1 | 12 | 624576 | 100 | 79.63 | 7.00 | 87 | 10.08 | 54.22 | 2.85 | 22.51 | 22.69 | 1.44 | 48.91 |
| 2019-2020 | 1 | 12 | 626904 | 100 | 73.85 | 6.67 | 80 | 14.98 | 64.9 | 3.18 | 23.89 | 24.10 | 1.44 | 57.42 |
| 2019-2020 | 1 | 12 | 626895 | 100 | 100 | 9.00 | 100 | 18.86 | 65.41 | 4.56 | 25.05 | 25.46 | 1.39 | 57.05 |
| 2019-2020 | 1 | 12 | 627057 | 100 | 84.76 | 7.33 | 93 | 10.01 | 66.17 | 3.24 | 27.9 | 28.09 | 1.46 | 56.03 |
| 2019-2020 | 1 | 12 | SIRVAN | 100 | 100 | 9.00 | 100 | 2.63 | 62.44 | 2.43 | 23.72 | 23.84 | 1.47 | 55.51 |
| 2019-2020 | 1 | 12 | ALBORZ | 100 | 100 | 9.00 | 100 | 13.94 | 61.54 | 1.46 | 22.52 | 22.57 | 1.51 | 55.41 |
| 2019-2020 | 1 | 12 | 627987 | 100 | 100 | 9.00 | 100 | 12.14 | 53.92 | 1.46 | 22.17 | 22.22 | 1.51 | 48.84 |
| 2019-2020 | 1 | 12 | 624582 | 100 | 100 | 9.00 | 100 | 14.29 | 67.72 | 1.92 | 22.47 | 22.55 | 1.49 | 60.62 |
| 2019-2020 | 1 | 13 | 623909 | 100 | 100 | 9.00 | 100 | 12.92 | 58.5 | 5.09 | 19.78 | 20.42 | 1.32 | 53.75 |
| 2019-2020 | 1 | 13 | MARVDASHT | 100 | 100 | 9.00 | 100 | 12.1 | 53.87 | 4.9 | 21.47 | 22.02 | 1.35 | 48.88 |
| 2019-2020 | 1 | 13 | GAHAR | 100 | 100 | 9.00 | 100 | 0.14 | 54.06 | 3.73 | 18.68 | 19.05 | 1.37 | 50.27 |
| 2019-2020 | 1 | 13 | 627616 | 100 | 100 | 9.00 | 100 | 11.86 | 60.8 | 2.52 | 18.73 | 18.90 | 1.44 | 56.48 |
| 2019-2020 | 1 | 13 | 627102 | 100 | 20.1 | 4.33 | 27 | 2.37 | 51.94 | 5.02 | 19.12 | 19.77 | 1.31 | 48.03 |
| 2019-2020 | 1 | 13 | NAZ | 100 | 100 | 9.00 | 100 | 11.37 | 61.89 | 3.79 | 19.92 | 20.28 | 1.38 | 56.83 |
| 2019-2020 | 1 | 13 | 623109 | 100 | 100 | 9.00 | 100 | 15.08 | 71.43 | 5.44 | 23.56 | 24.18 | 1.34 | 62.57 |
| 2019-2020 | 1 | 13 | 623091 | 100 | 36.08 | 4.67 | 47 | 8.12 | 57.73 | 2.69 | 21.26 | 21.43 | 1.44 | 52.61 |
| 2019-2020 | 1 | 13 | BISTON | 100 | 56.74 | 6.00 | 60 | 7.49 | 55.31 | 1.81 | 20.37 | 20.45 | 1.48 | 50.85 |
| 2019-2020 | 1 | 14 | 625433 | 100 | 88.7 | 8.00 | 93 | 10.34 | 61.35 | 3.16 | 21.2 | 21.43 | 1.42 | 55.80 |
| 2019-2020 | 1 | 14 | 623161 | 100 | 94.16 | 8.33 | 100 | 8.98 | 55.98 | 2.2 | 20.46 | 20.58 | 1.46 | 51.41 |
| 2019-2020 | 1 | 14 | AZAR2 | 100 | 82.19 | 7.33 | 93 | 11.17 | 59.86 | 1.94 | 23.77 | 23.85 | 1.49 | 53.31 |
| 2019-2020 | 1 | 14 | 626699 | 100 | 85.44 | 8.00 | 93 | 11.65 | 58.42 | 2.32 | 21.02 | 21.15 | 1.46 | 53.35 |
| 2019-2020 | 1 | 14 | 627873 | 100 | 91.93 | 8.33 | 100 | 8.92 | 58.28 | 1.36 | 17.96 | 18.01 | 1.50 | 54.56 |
| 2019-2020 | 1 | 14 | 622379 | 100 | 91.08 | 8.33 | 93 | 12.03 | 63.46 | 1.88 | 22.87 | 22.95 | 1.49 | 56.85 |
| 2019-2020 | 1 | 14 | 627587 | 100 | 72.22 | 7.00 | 80 | 11.82 | 59.35 | 3.22 | 23.52 | 23.74 | 1.43 | 52.93 |
| 2019-2020 | 1 | 14 | 623127 | 100 | 96.58 | 8.67 | 100 | 11.2 | 58.6 | 3.89 | 21.99 | 22.33 | 1.40 | 52.96 |
| 2019-2020 | 1 | 14 | 623507 | 100 | 100 | 9.00 | 100 | 12.88 | 62.05 | 2.74 | 19.65 | 19.84 | 1.43 | 57.18 |
| 2019-2020 | 1 | 15 | SHIRAZ | 100 | 94.29 | 8.33 | 100 | 13.54 | 64.35 | 4.63 | 25.21 | 25.63 | 1.39 | 56.09 |
| 2019-2020 | 1 | 15 | MIHAN | 100 | 98.58 | 8.67 | 100 | 16.12 | 70.63 | 3.07 | 22.61 | 22.82 | 1.44 | 62.81 |
| 2019-2020 | 1 | 15 | 621650 | 100 | 100 | 9.00 | 100 | 13.88 | 64.93 | 4.24 | 22.5 | 22.90 | 1.38 | 58.12 |
| 2019-2020 | 1 | 15 | 627066 | 100 | 100 | 9.00 | 100 | 11.96 | 61.01 | 3.26 | 19.36 | 19.63 | 1.40 | 56.35 |
| 2019-2020 | 1 | 15 | FONG | 100 | 100 | 9.00 | 100 | 12.86 | 65.43 | 2.28 | 23.82 | 23.93 | 1.48 | 57.96 |
| 2019-2020 | 1 | 15 | 627787 | 100 | 100 | 9.00 | 100 | 12.25 | 63.85 | 1.63 | 18.33 | 18.40 | 1.48 | 59.44 |
| 2019-2020 | 1 | 15 | 623139 | 100 | 99.11 | 9.00 | 100 | 16.15 | 60.67 | 3.05 | 24.8 | 24.99 | 1.45 | 53.40 |
| 2019-2020 | 1 | 15 | 621908 | 100 | 100 | 9.00 | 100 | 13.61 | 64.59 | 3.68 | 22.03 | 22.34 | 1.41 | 58.13 |
| 2019-2020 | 1 | 16 | HAMOON | 100 | 99.37 | 8.67 | 100 | 14.18 | 68.13 | 1.59 | 22.16 | 22.22 | 1.50 | 61.15 |
| 2019-2020 | 1 | 16 | ALVAND | 100 | 92.17 | 8.33 | 93 | 15.46 | 68.5 | 3.13 | 24.17 | 24.37 | 1.44 | 60.17 |
| 2019-2020 | 1 | 16 | 624944 | 100 | 90.85 | 8.00 | 93 | 14.08 | 66.34 | 4.67 | 22.11 | 22.60 | 1.36 | 59.46 |
| 2019-2020 | 1 | 16 | 624846 | 100 | 92 | 8.33 | 100 | 11.43 | 62.37 | 3.87 | 19.19 | 19.58 | 1.37 | 57.58 |
| 2019-2020 | 1 | 16 | PISHTAZ | 100 | 100 | 9.00 | 100 | 10.94 | 62.06 | 3.08 | 19.66 | 19.90 | 1.42 | 57.16 |
| 2019-2020 | 1 | 16 | 623428 | 100 | 100 | 9.00 | 100 | 10.66 | 64.69 | 3.44 | 22.01 | 22.28 | 1.42 | 58.25 |
| 2019-2020 | 1 | 16 | 622894 | 100 | 80.49 | 7.00 | 93 | 13.47 | 67.74 | 4.61 | 22.39 | 22.86 | 1.37 | 60.46 |
| 2019-2020 | 1 | 16 | ARTA | 100 | 95.18 | 8.33 | 100 | 14.29 | 65.58 | 3.52 | 22.93 | 23.20 | 1.42 | 58.49 |
| 2019-2020 | 1 | 16 | 623162 | 100 | 100 | 9.00 | 100 | 12.71 | 71.34 | 5.14 | 21.2 | 21.81 | 1.33 | 63.98 |
| 2019-2020 | 1 | 17 | 628189 | 100 | 80.43 | 7.33 | 87 | 9.01 | 57.27 | 4.12 | 23.65 | 24.01 | 1.40 | 50.99 |
| 2019-2020 | 1 | 17 | INIA | 100 | 100 | 9.00 | 100 | 16.55 | 64.71 | 1.94 | 23.05 | 23.13 | 1.49 | 57.80 |
| 2019-2020 | 1 | 17 | REYHANI | 100 | 76.36 | 6.67 | 93 | 11.26 | 57.11 | 3.64 | 23.89 | 24.17 | 1.42 | 50.77 |
| 2019-2020 | 1 | 17 | 626215 | 100 | 82.98 | 7.33 | 93 | 9 | 61.17 | 2.52 | 24.22 | 24.35 | 1.47 | 54.17 |
| 2019-2020 | 1 | 17 | KARIM | 100 | 100 | 9.00 | 100 | 11.82 | 54.65 | 3.43 | 19.43 | 19.73 | 1.40 | 50.54 |
| 2019-2020 | 1 | 17 | 627414 | 100 | 88.78 | 8.00 | 100 | 11.64 | 67.75 | 3.45 | 20.8 | 21.08 | 1.41 | 61.47 |
| 2019-2020 | 1 | 17 | 627399 | 100 | 66.67 | 6.67 | 80 | 12.8 | 69.31 | 1.62 | 22.6 | 22.66 | 1.50 | 61.85 |
| 2019-2020 | 1 | 17 | 623318 | 100 | 43.92 | 5.33 | 53 | 6.21 | 56.21 | 2.37 | 17.66 | 17.82 | 1.44 | 52.72 |
| 2019-2020 | 1 | 17 | 627963 | 100 | 95.59 | 8.33 | 100 | 15.19 | 71.82 | 5.48 | 23.03 | 23.67 | 1.34 | 63.20 |
| 2019-2020 | 1 | 18 | KARAJ1 | 100 | 88.24 | 8.00 | 100 | 14.1 | 72.24 | 3.76 | 22.43 | 22.74 | 1.40 | 64.11 |
| 2019-2020 | 1 | 18 | DEZ | 100 | 89.21 | 8.33 | 93 | 11.59 | 64.19 | 3.51 | 22.75 | 23.02 | 1.42 | 57.43 |
| 2019-2020 | 1 | 18 | 621706 | 100 | 95.12 | 8.33 | 100 | 19.85 | 70 | 4.3 | 24.45 | 24.83 | 1.40 | 61.06 |
| 2019-2020 | 1 | 18 | 626358 | 100 | 97.44 | 8.67 | 100 | 15.04 | 65.48 | 4.6 | 24.02 | 24.46 | 1.38 | 57.69 |
| 2019-2020 | 1 | 18 | 623980 | 100 | 92.25 | 8.33 | 100 | 14.29 | 62.9 | 4.03 | 21.73 | 22.10 | 1.39 | 56.82 |
| 2019-2020 | 1 | 18 | 626776 | 100 | 82.87 | 7.33 | 93 | 8.22 | 54.14 | 1.99 | 16.89 | 17.01 | 1.45 | 51.09 |
| 2019-2020 | 1 | 18 | 625127 | 100 | 100 | 9.00 | 100 | 11.04 | 59.14 | 1.51 | 21.9 | 21.95 | 1.50 | 53.62 |
| 2019-2020 | 1 | 18 | CHAMRAN2 | 100 | 87.24 | 8.00 | 93 | 13.63 | 60.85 | 4.47 | 22.11 | 22.56 | 1.37 | 54.82 |
| 2019-2020 | 1 | 18 | NICKNEJAD | 100 | 31.76 | 4.67 | 40 | 3.23 | 52.1 | 4.55 | 20.82 | 21.31 | 1.36 | 47.57 |
| 2019-2020 | 1 | 18 | 623503 | 100 | 72.51 | 6.67 | 87 | 8.95 | 63.89 | 3.76 | 20.68 | 21.02 | 1.39 | 58.22 |
| 2019-2020 | 1 | 19 | FRONTANA | 0 | 0 | 1.00 | 0 | 14.57 | 72.56 | 2.35 | 26.4 | 26.50 | 1.48 | 61.85 |
| 2019-2020 | 1 | 19 | 627881 | 100 | 29.81 | 4.67 | 40 | 9.46 | 63.38 | 3.6 | 21.53 | 21.83 | 1.41 | 57.37 |
| 2019-2020 | 1 | 19 | 623377 | 100 | 100 | 9.00 | 100 | 16.96 | 72.47 | 1.77 | 23.68 | 23.75 | 1.50 | 63.64 |
| 2019-2020 | 1 | 19 | MV17 | 100 | 76.74 | 7.67 | 93 | 11.66 | 57.93 | 2.52 | 19.96 | 20.12 | 1.45 | 53.37 |
| 2019-2020 | 1 | 19 | 627948 | 100 | 100 | 9.00 | 100 | 14.14 | 60.66 | 3.36 | 22.86 | 23.11 | 1.42 | 54.38 |
| 2019-2020 | 1 | 19 | BAHAR | 100 | 100 | 9.00 | 100 | 11.59 | 55.31 | 3.1 | 22.03 | 22.25 | 1.43 | 50.08 |
| 2019-2020 | 1 | 19 | 624818 | 100 | 55.75 | 6.00 | 67 | 4.81 | 49.42 | 2.55 | 18.2 | 18.38 | 1.43 | 46.18 |
| 2019-2020 | 1 | 19 | 623345 | 100 | 72.46 | 7.00 | 80 | 13.95 | 70.84 | 4.07 | 22.72 | 23.08 | 1.39 | 62.81 |
| 2019-2020 | 1 | 20 | 627908 | 100 | 66.95 | 6.67 | 73 | 9.18 | 60.97 | 4.75 | 21.81 | 22.32 | 1.36 | 55.04 |
| 2019-2020 | 1 | 20 | 623169 | 0 | 0 | 1.00 | 0 | 2.6 | 56.8 | 3.81 | 22.02 | 22.35 | 1.40 | 51.36 |
| 2019-2020 | 1 | 20 | ATRAK | 100 | 89.11 | 7.67 | 100 | 6.85 | 54.3 | 3.89 | 21.82 | 22.16 | 1.39 | 49.21 |
| 2019-2020 | 1 | 20 | 627356 | 100 | 96.32 | 8.67 | 100 | 12.06 | 68.34 | 2.87 | 21.81 | 22.00 | 1.44 | 61.45 |
| 2019-2020 | 1 | 20 | 626943 | 100 | 36.36 | 4.67 | 47 | 4.44 | 48.59 | 2.42 | 17.88 | 18.04 | 1.44 | 45.52 |
| 2019-2020 | 1 | 20 | 627417 | 100 | 100 | 9.00 | 100 | 8.52 | 56.94 | 2.94 | 19.21 | 19.43 | 1.42 | 52.76 |
| 2019-2020 | 1 | 20 | 628088 | 100 | 97.58 | 8.67 | 100 | 13.81 | 65.06 | 2.41 | 21.37 | 21.51 | 1.46 | 58.97 |
| 2019-2020 | 1 | 20 | BAYAT | 100 | 100 | 9.00 | 100 | 13.24 | 62.72 | 2.45 | 23.21 | 23.34 | 1.47 | 56.02 |
| 2019-2020 | 1 | 20 | FALAT | 100 | 100 | 9.00 | 100 | 11.39 | 56.99 | 4.35 | 23.18 | 23.58 | 1.39 | 50.95 |
| 2019-2020 | 1 | 20 | 626933 | 100 | 100 | 9.00 | 100 | 11.14 | 60 | 2.78 | 23.33 | 23.50 | 1.45 | 53.61 |
| 2019-2020 | 1 | 21 | 623125 | 100 | 46.59 | 5.00 | 53 | 11.23 | 60.73 | 4.56 | 22.99 | 23.44 | 1.37 | 54.27 |
| 2019-2020 | 1 | 21 | DARAB2 | 100 | 100 | 9.00 | 100 | 5.44 | 46.24 | 3.86 | 18.17 | 18.58 | 1.36 | 43.12 |
| 2019-2020 | 1 | 21 | 624980 | 100 | 88.1 | 8.00 | 93 | 11.06 | 59.87 | 4.93 | 23.34 | 23.85 | 1.36 | 53.32 |
| 2019-2020 | 1 | 21 | 625281 | 100 | 50 | 6.00 | 60 | 2.42 | 53.65 | 2.06 | 18.02 | 18.14 | 1.46 | 50.23 |
| 2019-2020 | 1 | 21 | TAJAN | 100 | 98.53 | 8.67 | 100 | 11.7 | 59.02 | 4.18 | 20.65 | 21.07 | 1.37 | 53.92 |
| 2019-2020 | 1 | 21 | 623382 | 100 | 77.27 | 7.00 | 80 | 12.18 | 64.91 | 3.46 | 23.28 | 23.54 | 1.42 | 57.75 |
| 2019-2020 | 1 | 21 | 624580 | 100 | 19.39 | 3.67 | 33 | 1.48 | 50.04 | 3.43 | 18.36 | 18.68 | 1.39 | 46.66 |
| 2019-2020 | 1 | 21 | KAVIR | 100 | 67.31 | 6.67 | 73 | 7.12 | 57.72 | 3.01 | 22.04 | 22.24 | 1.44 | 52.23 |
| 2019-2020 | 1 | 21 | 626883 | 100 | 100 | 9.00 | 100 | 14.68 | 66.67 | 1.03 | 24.02 | 24.04 | 1.53 | 58.90 |
| 2019-2020 | 1 | 21 | BAM | 100 | 93.33 | 8.33 | 93 | 12.47 | 66.62 | 2.39 | 23.71 | 23.83 | 1.47 | 58.99 |
| 2019-2020 | 1 | 22 | 623136 | 100 | 88.6 | 7.67 | 93 | 7.41 | 60.14 | 3.53 | 20.53 | 20.83 | 1.40 | 55.02 |
| 2019-2020 | 1 | 22 | KAVEH | 100 | 99.13 | 9.00 | 100 | 15.79 | 58.77 | 2.63 | 23.75 | 23.90 | 1.46 | 52.35 |
| 2019-2020 | 1 | 22 | 626923 | 100 | 78.2 | 7.00 | 93 | 6.11 | 69.53 | 2.36 | 22.66 | 22.78 | 1.47 | 61.95 |
| 2019-2020 | 1 | 22 | MOGHAN1 | 100 | 96 | 8.33 | 100 | 8.6 | 61.08 | 2.58 | 19.62 | 19.79 | 1.44 | 56.34 |
| 2019-2020 | 1 | 22 | 627099 | 100 | 96.55 | 8.67 | 100 | 7.79 | 53.42 | 1.43 | 16.83 | 16.89 | 1.49 | 50.45 |
| 2019-2020 | 1 | 22 | 626260 | 100 | 52.33 | 5.67 | 60 | 2.92 | 51.32 | 3.81 | 17.89 | 18.29 | 1.36 | 48.00 |
| 2019-2020 | 1 | 22 | DARYA | 100 | 18.75 | 4.00 | 27 | 10.92 | 56.64 | 4.04 | 21.47 | 21.85 | 1.38 | 51.45 |
| 2019-2020 | 1 | 22 | 623176 | 100 | 49.23 | 5.67 | 60 | 4.73 | 56.23 | 3.08 | 21.17 | 21.39 | 1.43 | 51.28 |
| 2019-2020 | 1 | 22 | 621492 | 100 | 23.98 | 4.33 | 33 | 4.33 | 60.04 | 4.2 | 23.47 | 23.84 | 1.39 | 53.47 |
| 2019-2020 | 1 | 22 | URUOM | 100 | 87.92 | 7.67 | 100 | 10.36 | 62.42 | 3.09 | 21.28 | 21.50 | 1.43 | 56.70 |
| 2019-2020 | 1 | 23 | 627723 | 100 | 89.43 | 7.67 | 100 | 9.21 | 59.32 | 4.41 | 21.28 | 21.73 | 1.37 | 53.88 |
| 2019-2020 | 1 | 23 | BEZOSTAYA | 80 | 2.7 | 1.67 | 13 | 1.68 | 57.27 | 5.45 | 20.41 | 21.13 | 1.31 | 52.33 |
| 2019-2020 | 1 | 23 | 626565 | 100 | 100 | 9.00 | 100 | 8.96 | 51.76 | 2.54 | 18.2 | 18.38 | 1.43 | 48.38 |
| 2019-2020 | 1 | 23 | 4820 | 100 | 97.01 | 8.67 | 100 | 8.89 | 65.54 | 2.6 | 22.17 | 22.32 | 1.45 | 58.94 |
| 2019-2020 | 1 | 23 | OFOG | 100 | 91.38 | 8.00 | 100 | 9.54 | 61.98 | 2.76 | 22.74 | 22.91 | 1.45 | 55.61 |
| 2019-2020 | 1 | 23 | 624946 | 20 | 10.32 | 2.33 | 13 | 2.07 | 62.57 | 1.98 | 22.08 | 22.17 | 1.48 | 56.50 |
| 2019-2020 | 1 | 23 | 624894 | 100 | 81.08 | 7.33 | 87 | 7.29 | 53.21 | 1.71 | 16.16 | 16.25 | 1.47 | 50.47 |
| 2019-2020 | 1 | 23 | AZAR1 | 100 | 33.9 | 5.00 | 40 | 3.95 | 48.38 | 2.97 | 18.71 | 18.94 | 1.41 | 45.01 |
| 2019-2020 | 1 | 23 | 626958 | 100 | 40.22 | 5.00 | 53 | 5.4 | 67.49 | 1.44 | 23.41 | 23.45 | 1.51 | 59.91 |
| 2019-2020 | 1 | 23 | 623417 | 100 | 48.12 | 5.67 | 53 | 8.98 | 64.1 | 2.32 | 18 | 18.15 | 1.44 | 59.77 |
| 2019-2020 | 1 | 24 | 624378 | 100 | 41.94 | 5.33 | 47 | 3.61 | 56.88 | 3.59 | 19.67 | 19.99 | 1.39 | 52.47 |
| 2019-2020 | 1 | 24 | SISTAN | 100 | 66.67 | 6.33 | 80 | 6.71 | 52.54 | 3.37 | 18.76 | 19.06 | 1.39 | 48.86 |
| 2019-2020 | 1 | 24 | 626872 | 100 | 94.57 | 8.33 | 100 | 7.18 | 49.89 | 3.05 | 15.19 | 15.49 | 1.37 | 47.55 |
| 2019-2020 | 1 | 24 | 624381 | 100 | 84.42 | 7.33 | 93 | 8.12 | 68.16 | 3.31 | 21.91 | 22.16 | 1.42 | 61.21 |
| 2019-2020 | 1 | 24 | 625810 | 100 | 43.18 | 5.33 | 53 | 3.83 | 61.77 | 3.65 | 21.46 | 21.77 | 1.40 | 56.01 |
| 2019-2020 | 1 | 24 | 622311 | 100 | 79.01 | 7.33 | 87 | 8.22 | 57.52 | 3.27 | 17.51 | 17.81 | 1.39 | 53.94 |
| 2019-2020 | 1 | 24 | 627385 | 100 | 86.87 | 7.67 | 100 | 11.78 | 65.9 | 3.63 | 22.45 | 22.74 | 1.41 | 59.01 |
| 2019-2020 | 1 | 24 | 623905 | 100 | 72.22 | 6.67 | 80 | 4.68 | 49.38 | 3.63 | 18.29 | 18.65 | 1.37 | 46.05 |
| 2019-2020 | 1 | 24 | 627460 | 100 | 78.38 | 7.00 | 87 | 10.81 | 64.61 | 4.06 | 21.74 | 22.12 | 1.39 | 58.27 |
| 2019-2020 | 1 | 25 | 627760 | 100 | 73.57 | 6.67 | 80 | 5.67 | 67.34 | 2.31 | 25.63 | 25.73 | 1.48 | 58.42 |
| 2019-2020 | 1 | 25 | 625362 | 100 | 77.14 | 7.00 | 87 | 7.2 | 56.03 | 3 | 24.21 | 24.40 | 1.45 | 49.72 |
| 2019-2020 | 1 | 25 | 623274 | 100 | 100 | 9.00 | 100 | 12.65 | 62.42 | 2.99 | 20.17 | 20.39 | 1.42 | 57.24 |
| 2019-2020 | 1 | 25 | 626736 | 100 | 68.46 | 6.67 | 80 | 7.8 | 56.26 | 2.47 | 21.61 | 21.75 | 1.46 | 51.15 |
| 2019-2020 | 1 | 25 | 624585 | 100 | 27.63 | 4.33 | 40 | 1.62 | 49.52 | 4.2 | 19 | 19.46 | 1.35 | 45.90 |
| 2019-2020 | 1 | 25 | KOOHDASHT | 100 | 89.91 | 8.00 | 100 | 12.29 | 63.43 | 1.84 | 25.63 | 25.70 | 1.50 | 55.30 |
| 2019-2020 | 1 | 25 | NAVID | 100 | 100 | 9.00 | 100 | 12.91 | 67.61 | 1.55 | 22.74 | 22.79 | 1.50 | 60.39 |
| 2019-2020 | 1 | 25 | KARAJ2 | 100 | 84.09 | 8.00 | 87 | 12.93 | 69.51 | 1.21 | 23.28 | 23.31 | 1.52 | 61.62 |
| 2019-2020 | 1 | 25 | CHAMRAN | 100 | 100 | 9.00 | 100 | 0.12 | 49.72 | 1.52 | 15.36 | 15.44 | 1.47 | 47.40 |
| 2019-2020 | 1 | 25 | 624864 | 100 | 48.05 | 5.67 | 53 | 6.7 | 61.3 | 3.75 | 20.49 | 20.83 | 1.39 | 56.05 |
| 2019-2020 | 1 | 26 | 625123 | 100 | 90.62 | 8.33 | 93 | 11.13 | 52.68 | 2.75 | 19.88 | 20.07 | 1.43 | 48.60 |
| 2019-2020 | 1 | 26 | CASGOGEN | 20 | 2.34 | 1.67 | 7 | 7.52 | 49.66 | 2.71 | 16.9 | 17.12 | 1.41 | 46.83 |
| 2019-2020 | 1 | 26 | 626764 | 100 | 24.02 | 4.33 | 33 | 5.09 | 52.35 | 3.12 | 17.6 | 17.87 | 1.40 | 49.11 |
| 2019-2020 | 1 | 26 | 624804 | 100 | 39.58 | 5.00 | 47 | 4.97 | 59.14 | 3.75 | 17.81 | 18.20 | 1.36 | 55.27 |
| 2019-2020 | 1 | 26 | 626846 | 100 | 100 | 9.00 | 100 | 8.05 | 49.34 | 3.59 | 17.04 | 17.41 | 1.36 | 46.43 |
| 2019-2020 | 1 | 26 | ZARRIN | 100 | 32.69 | 5.00 | 40 | 5.04 | 56.16 | 4.59 | 19.2 | 19.74 | 1.34 | 51.92 |
| 2019-2020 | 1 | 26 | 626814 | 100 | 86.17 | 7.67 | 87 | 7.6 | 57.62 | 2.46 | 21.84 | 21.98 | 1.46 | 52.26 |
| 2019-2020 | 1 | 26 | 626825 | 100 | 80.41 | 7.67 | 87 | 10.47 | 61.35 | 2.59 | 21.7 | 21.85 | 1.45 | 55.60 |
| 2019-2020 | 1 | 26 | DEYHEM | 100 | 54.65 | 6.00 | 60 | 7.51 | 53.45 | 3.52 | 17.1 | 17.46 | 1.37 | 50.28 |
| 2019-2020 | 1 | 26 | 624251 | 100 | 49.32 | 5.67 | 60 | 5.59 | 57.54 | 2.77 | 21.25 | 21.43 | 1.44 | 52.44 |
| 2019-2020 | 1 | 27 | 623953 | 20 | 2.34 | 1.67 | 7 | 0.24 | 67.46 | 2.08 | 26.14 | 26.22 | 1.49 | 58.21 |
| 2019-2020 | 1 | 27 | 626855 | 100 | 92.94 | 8.33 | 100 | 15.15 | 64.43 | 2.52 | 24.79 | 24.92 | 1.47 | 56.57 |
| 2019-2020 | 1 | 27 | 623008 | 100 | 91.49 | 8.33 | 100 | 11.25 | 57.04 | 2.91 | 21.96 | 22.15 | 1.44 | 51.67 |
| 2019-2020 | 1 | 27 | 621669 | 100 | 95 | 8.33 | 100 | 10.15 | 59.21 | 4.52 | 19.83 | 20.34 | 1.35 | 54.42 |
| 2019-2020 | 1 | 27 | SHARYAR | 100 | 100 | 9.00 | 100 | 8.5 | 69.49 | 4.68 | 24.93 | 25.37 | 1.39 | 60.32 |
| 2019-2020 | 1 | 27 | ADL | 100 | 92.31 | 8.00 | 100 | 10.45 | 61.79 | 2 | 17.12 | 17.24 | 1.45 | 58.08 |
| 2019-2020 | 1 | 27 | PANJAMO62 | 100 | 77.43 | 7.33 | 87 | 13.11 | 70.44 | 3.36 | 22.65 | 22.90 | 1.42 | 62.61 |
| 2019-2020 | 1 | 27 | 624939 | 100 | 62 | 6.33 | 73 | 10.21 | 67.64 | 3.98 | 19.57 | 19.97 | 1.37 | 61.97 |
| 2019-2020 | 1 | 27 | PISHGAM | 100 | 67.94 | 6.67 | 80 | 11.6 | 64.53 | 4.2 | 24.05 | 24.41 | 1.40 | 56.94 |
| 2019-2020 | 1 | 27 | 626360 | 100 | 73.79 | 7.00 | 80 | 9.9 | 57.61 | 4.35 | 21.03 | 21.48 | 1.37 | 52.48 |
| 2019-2020 | 1 | 28 | 624983 | 100 | 49.12 | 6.00 | 60 | 9.32 | 64.85 | 5.14 | 22.47 | 23.05 | 1.35 | 57.97 |
| 2019-2020 | 1 | 28 | SIVAND | 100 | 46.79 | 5.33 | 60 | 12.99 | 60.73 | 4.5 | 24.25 | 24.66 | 1.39 | 53.63 |
| 2019-2020 | 1 | 28 | 627551 | 100 | 5.8 | 2.67 | 20 | 0.52 | 45.28 | 4.56 | 17.72 | 18.30 | 1.32 | 42.30 |
| 2019-2020 | 1 | 28 | SHAHI | 100 | 61.59 | 6.67 | 73 | 10.93 | 60.37 | 4.57 | 21.63 | 22.11 | 1.36 | 54.62 |
| 2019-2020 | 1 | 28 | 621717 | 100 | 19.09 | 3.67 | 33 | 4.15 | 59.69 | 2.4 | 24.25 | 24.37 | 1.47 | 52.90 |
| 2019-2020 | 1 | 28 | ARVAND | 100 | 76.4 | 6.67 | 93 | 8.91 | 56.09 | 2.21 | 18.7 | 18.83 | 1.45 | 52.22 |
| 2019-2020 | 1 | 28 | MORVARID | 100 | 45.39 | 5.33 | 53 | 11.41 | 58.01 | 2.18 | 23.65 | 23.75 | 1.48 | 51.76 |
| 2019-2020 | 1 | 28 | 624863 | 100 | 18.52 | 3.67 | 27 | 2.2 | 63.35 | 2.34 | 22.03 | 22.15 | 1.46 | 57.17 |
| 2019-2020 | 1 | 28 | 626881 | 100 | 56.08 | 6.00 | 67 | 10.56 | 63.76 | 3.77 | 24.07 | 24.36 | 1.42 | 56.33 |
| 2019-2020 | 1 | 28 | TOUBARI | 100 | 43.09 | 5.33 | 53 | 7.03 | 59.79 | 1.96 | 20.51 | 20.60 | 1.48 | 54.82 |
| 2019-2020 | 1 | 29 | SHINGHAI | 100 | 48 | 5.67 | 53 | 10.81 | 60.18 | 5.05 | 20.37 | 20.99 | 1.33 | 54.99 |
| 2019-2020 | 1 | 29 | KARAJ3 | 100 | 53.92 | 6.00 | 60 | 7.91 | 61.2 | 4.75 | 20.52 | 21.06 | 1.34 | 55.85 |
| 2019-2020 | 1 | 29 | 624947 | 100 | 77.21 | 7.33 | 87 | 9.07 | 53.75 | 4.5 | 19.03 | 19.55 | 1.34 | 49.79 |
| 2019-2020 | 1 | 29 | GOLESTAN | 100 | 87.5 | 7.67 | 93 | 13.34 | 65.51 | 3.15 | 18.85 | 19.11 | 1.41 | 60.57 |
| 2019-2020 | 1 | 29 | 624963 | 100 | 35.94 | 5.00 | 47 | 5.23 | 59.1 | 3.9 | 19.96 | 20.34 | 1.38 | 54.32 |
| 2019-2020 | 1 | 29 | AZADI | 100 | 94.44 | 8.33 | 100 | 11.46 | 56.76 | 3.02 | 21.48 | 21.69 | 1.43 | 51.62 |
| 2019-2020 | 1 | 29 | 623123 | 100 | 100 | 9.00 | 100 | 11.52 | 62.09 | 4.35 | 22.45 | 22.87 | 1.38 | 55.73 |
| 2019-2020 | 1 | 29 | AFLAK | 100 | 100 | 9.00 | 100 | 16.93 | 66.51 | 1.92 | 24.22 | 24.30 | 1.49 | 58.63 |
| 2019-2020 | 1 | 29 | KHAZAR1 | 100 | 81.01 | 7.00 | 93 | 9.98 | 62.56 | 1.69 | 23.46 | 23.52 | 1.50 | 55.78 |
| 2019-2020 | 1 | 30 | 623069 | 100 | 37.67 | 5.33 | 47 | 9.8 | 64.28 | 2.66 | 23.25 | 23.40 | 1.46 | 57.30 |
| 2019-2020 | 1 | 30 | ZAGROS | 100 | 49.22 | 6.00 | 60 | 9.82 | 58.82 | 1.85 | 22.06 | 22.14 | 1.49 | 53.25 |
| 2019-2020 | 1 | 30 | 624901 | 100 | 46.71 | 5.67 | 53 | 3.32 | 55.8 | 2.32 | 18.63 | 18.77 | 1.45 | 51.98 |
| 2019-2020 | 1 | 30 | SISON | 100 | 50 | 5.67 | 53 | 9.99 | 66.03 | 2.71 | 22.81 | 22.97 | 1.45 | 58.99 |
| 2019-2020 | 1 | 30 | 624215 | 0 | 0 | 1.00 | 0 | 0.41 | 52.91 | 2.05 | 18.09 | 18.21 | 1.46 | 49.51 |
| 2019-2020 | 1 | 30 | GHODS | 100 | 100 | 9.00 | 100 | 10.59 | 57.86 | 3.53 | 20.06 | 20.37 | 1.40 | 53.20 |
| 2019-2020 | 1 | 30 | 624861 | 100 | 47.8 | 5.33 | 60 | 10.37 | 68.56 | 3.09 | 21.68 | 21.90 | 1.43 | 61.68 |
| 2019-2020 | 1 | 30 | RIJAW | 100 | 98.25 | 8.67 | 100 | 16.1 | 67.23 | 3.93 | 23.48 | 23.81 | 1.40 | 59.50 |
| 2019-2020 | 1 | 30 | SPAHAN | 100 | 98.67 | 8.67 | 100 | 10.32 | 55.91 | 2.63 | 21.41 | 21.57 | 1.45 | 50.92 |
| 2019-2020 | 1 | 31 | 627055 | 100 | 42.68 | 5.33 | 53 | 7.4 | 63.66 | 3.93 | 17.93 | 18.36 | 1.36 | 59.29 |
| 2019-2020 | 1 | 31 | 626932 | 100 | 33.33 | 5.00 | 40 | 10.6 | 70.56 | 3.95 | 23.62 | 23.95 | 1.41 | 62.05 |
| 2019-2020 | 1 | 31 | PARSI | 100 | 100 | 9.00 | 100 | 13.46 | 69.49 | 4.28 | 21.63 | 22.05 | 1.38 | 62.36 |
| 2019-2020 | 1 | 31 | 624838 | 100 | 25 | 4.33 | 33 | 8.3 | 65.21 | 4.59 | 21.57 | 22.05 | 1.36 | 58.81 |
| 2019-2020 | 1 | 31 | 621736 | 100 | 36.67 | 5.00 | 47 | 4.88 | 61.76 | 2.36 | 23.16 | 23.28 | 1.47 | 55.23 |
| 2019-2020 | 1 | 31 | 621869 | 80 | 24.47 | 3.67 | 27 | 6.17 | 68.41 | 2.16 | 21.8 | 21.91 | 1.47 | 61.56 |
| 2019-2020 | 1 | 31 | 628012 | 100 | 9.82 | 3.00 | 20 | 1.43 | 57.88 | 2.9 | 18.36 | 18.59 | 1.41 | 53.96 |
| 2019-2020 | 1 | 31 | 622099 | 0 | 0 | 1.00 | 0 | 0.14 | 50.85 | 1.54 | 18.57 | 18.63 | 1.49 | 47.44 |
| 2019-2020 | 1 | 31 | 622105 | 0 | 0 | 1.00 | 0 | 0.11 | 47.63 | 5.81 | 19.81 | 20.64 | 1.29 | 43.71 |
| 2019-2020 | 1 | 32 | 621420 | 100 | 27.89 | 4.67 | 33 | 5.95 | 54.96 | 2.87 | 20.42 | 20.62 | 1.43 | 50.46 |
| 2019-2020 | 1 | 32 | 623266 | 60 | 17.68 | 3.00 | 20 | 2.09 | 58.38 | 2.17 | 20.52 | 20.63 | 1.47 | 53.55 |
| 2019-2020 | 1 | 32 | 621712 | 100 | 76.67 | 7.00 | 87 | 7.28 | 54.04 | 4.76 | 17.09 | 17.74 | 1.30 | 50.73 |
| 2019-2020 | 1 | 32 | 620903 | 100 | 30 | 4.33 | 40 | 5.96 | 47.25 | 1.59 | 18.48 | 18.55 | 1.48 | 44.08 |
| 2019-2020 | 1 | 32 | 625139 | 20 | 2.21 | 1.67 | 7 | 3.05 | 56.29 | 2.31 | 14.36 | 14.54 | 1.41 | 53.93 |
| 2019-2020 | 1 | 32 | 622098 | 80 | 31.37 | 3.67 | 40 | 10.54 | 69.79 | 4.26 | 21.48 | 21.90 | 1.38 | 62.69 |
| 2019-2020 | 1 | 32 | 625263 | 0 | 0 | 1.00 | 0 | 0.39 | 55.86 | 1.49 | 17.97 | 18.03 | 1.49 | 52.32 |
| 2019-2020 | 1 | 32 | 622264 | 0 | 0 | 1.00 | 0 | 0.12 | 50.53 | 4.82 | 19.02 | 19.62 | 1.32 | 46.78 |
| 2019-2020 | 1 | 32 | 622272 | 0 | 0 | 1.00 | 0 | 0.11 | 51.09 | 2.6 | 18.78 | 18.96 | 1.43 | 47.54 |
| 2019-2020 | 1 | 32 | 627905 | 0 | 0 | 1.00 | 0 | 1.1 | 55.79 | 1.2 | 15.18 | 15.23 | 1.49 | 53.24 |
| 2019-2020 | 2 | 1 | 627036 | 100 | 93.41 | 8.33 | 100 | 13.19 | 62.97 | 3.51 | 24.33 | 24.58 | 1.43 | 55.55 |
| 2019-2020 | 2 | 1 | 626156 | 100 | 90.3 | 8.00 | 100 | 7.13 | 52.48 | 3.63 | 20.72 | 21.04 | 1.40 | 48.03 |
| 2019-2020 | 2 | 1 | ROSHAN | 100 | 94.41 | 8.33 | 100 | 11.43 | 55.34 | 4.49 | 20.04 | 20.54 | 1.35 | 50.84 |
| 2019-2020 | 2 | 1 | 627852 | 100 | 100 | 9.00 | 100 | 13.1 | 62.35 | 3.75 | 23.02 | 23.32 | 1.41 | 55.71 |
| 2019-2020 | 2 | 1 | 628114 | 100 | 97.48 | 8.67 | 100 | 11.14 | 62.41 | 3.46 | 22.81 | 23.07 | 1.42 | 55.89 |
| 2019-2020 | 2 | 1 | 624985 | 100 | 71.76 | 7.00 | 80 | 9.14 | 52.55 | 5.42 | 22.51 | 23.15 | 1.33 | 47.20 |
| 2019-2020 | 2 | 1 | 624837 | 100 | 80.99 | 7.33 | 87 | 11.74 | 69.19 | 3.36 | 22.26 | 22.51 | 1.42 | 61.84 |
| 2019-2020 | 2 | 1 | TOUS | 100 | 64.42 | 6.33 | 73 | 7.29 | 52.25 | 4.22 | 21.02 | 21.44 | 1.37 | 47.66 |
| 2019-2020 | 2 | 1 | 623473 | 100 | 67.37 | 6.67 | 73 | 8.77 | 55.95 | 4.08 | 20.83 | 21.23 | 1.38 | 51.10 |
| 2019-2020 | 2 | 2 | MOGHAN2 | 100 | 92 | 8.33 | 100 | 12.3 | 65.17 | 3.98 | 24.31 | 24.63 | 1.41 | 57.34 |
| 2019-2020 | 2 | 2 | 627359 | 100 | 92.47 | 8.00 | 100 | 11.09 | 57.21 | 3.99 | 21.12 | 21.49 | 1.38 | 52.12 |
| 2019-2020 | 2 | 2 | 627038 | 100 | 97.53 | 9.00 | 100 | 12.57 | 60.1 | 5.23 | 23.26 | 23.84 | 1.35 | 53.52 |
| 2019-2020 | 2 | 2 | 621704 | 100 | 47.57 | 5.67 | 53 | 5.12 | 47.93 | 1.51 | 16.73 | 16.80 | 1.48 | 45.29 |
| 2019-2020 | 2 | 2 | 626566 | 100 | 82.89 | 7.33 | 87 | 6.8 | 52.64 | 4.32 | 20.69 | 21.14 | 1.36 | 48.14 |
| 2019-2020 | 2 | 2 | SHAHPASSAND | 100 | 84.09 | 7.67 | 87 | 17.16 | 71.98 | 3.85 | 23.13 | 23.45 | 1.41 | 63.46 |
| 2019-2020 | 2 | 2 | 623508 | 100 | 75.94 | 7.00 | 80 | 11.78 | 65.25 | 4.17 | 24.43 | 24.78 | 1.40 | 57.32 |
| 2019-2020 | 2 | 2 | 621735 | 100 | 74.19 | 6.67 | 87 | 9.12 | 54.2 | 2.04 | 19.43 | 19.54 | 1.47 | 50.21 |
| 2019-2020 | 2 | 2 | 623338 | 100 | 99.42 | 8.67 | 100 | 15.91 | 60.49 | 3 | 23.89 | 24.08 | 1.45 | 53.73 |
| 2019-2020 | 2 | 2 | 621668 | 100 | 82.24 | 7.33 | 87 | 8.46 | 49.79 | 3.1 | 15.74 | 16.04 | 1.38 | 47.29 |
| 2019-2020 | 2 | 3 | 621421 | 100 | 84.43 | 7.33 | 93 | 7.03 | 55.58 | 1.47 | 16.26 | 16.33 | 1.48 | 52.67 |
| 2019-2020 | 2 | 3 | 624240 | 100 | 95.27 | 8.33 | 100 | 7.3 | 56.21 | 3.93 | 20.65 | 21.02 | 1.38 | 51.43 |
| 2019-2020 | 2 | 3 | 623344 | 100 | 86.11 | 7.67 | 100 | 12.06 | 62.99 | 3.76 | 24.04 | 24.33 | 1.42 | 55.71 |
| 2019-2020 | 2 | 3 | 624941 | 0 | 0 | 1.00 | 0 | 0.15 | 54.12 | 4 | 16.67 | 17.14 | 1.34 | 51.02 |
| 2019-2020 | 2 | 3 | 621716 | 100 | 83.55 | 7.00 | 100 | 13.65 | 63.53 | 6.92 | 25.4 | 26.33 | 1.30 | 55.02 |
| 2019-2020 | 2 | 3 | 627236 | 100 | 92.23 | 8.33 | 100 | 10.09 | 64.57 | 4.29 | 21.07 | 21.50 | 1.37 | 58.56 |
| 2019-2020 | 2 | 3 | 624911 | 100 | 94.16 | 8.33 | 100 | 12.14 | 66.82 | 2.28 | 20.24 | 20.37 | 1.46 | 61.07 |
| 2019-2020 | 2 | 4 | OHADI | 100 | 94.81 | 8.00 | 100 | 15.62 | 73.25 | 4.68 | 22.48 | 22.96 | 1.37 | 64.75 |
| 2019-2020 | 2 | 4 | 624990 | 100 | 85.89 | 7.33 | 93 | 9.71 | 65.37 | 4.19 | 22.8 | 23.18 | 1.39 | 58.33 |
| 2019-2020 | 2 | 4 | 627484 | 100 | 78.38 | 7.00 | 87 | 9.91 | 61.15 | 6.26 | 21.38 | 22.28 | 1.29 | 55.22 |
| 2019-2020 | 2 | 4 | 627688 | 100 | 97.52 | 9.00 | 100 | 6.83 | 52.94 | 2.54 | 15.79 | 15.99 | 1.41 | 50.30 |
| 2019-2020 | 2 | 4 | 627416 | 100 | 98.77 | 9.00 | 100 | 15.94 | 73.45 | 3.69 | 19.82 | 20.16 | 1.39 | 66.66 |
| 2019-2020 | 2 | 4 | 625661 | 100 | 94.59 | 8.67 | 100 | 8.28 | 49.89 | 1.61 | 17.34 | 17.41 | 1.48 | 46.95 |
| 2019-2020 | 2 | 4 | 625081 | 100 | 71.2 | 6.67 | 80 | 10.7 | 57.73 | 4.36 | 22.96 | 23.37 | 1.38 | 51.70 |
| 2019-2020 | 2 | 4 | 627061 | 100 | 94.23 | 8.33 | 100 | 12.04 | 61.85 | 3.68 | 21.95 | 22.26 | 1.40 | 55.83 |
| 2019-2020 | 2 | 4 | NEISHABOUR | 100 | 93.04 | 8.00 | 100 | 15.83 | 67.13 | 4.28 | 22.05 | 22.46 | 1.38 | 60.19 |
| 2019-2020 | 2 | 4 | 624315 | 100 | 93.01 | 8.00 | 100 | 14.23 | 66.9 | 3.92 | 24.91 | 25.22 | 1.41 | 58.39 |
| 2019-2020 | 2 | 5 | 627103 | 0 | 0 | 1.00 | 0 | 0.65 | 49.73 | 1.97 | 16.36 | 16.48 | 1.45 | 47.10 |
| 2019-2020 | 2 | 5 | 623475 | 100 | 96.3 | 8.67 | 100 | 9.86 | 57.96 | 3.29 | 20.06 | 20.33 | 1.41 | 53.30 |
| 2019-2020 | 2 | 5 | 624596 | 100 | 89.54 | 8.00 | 100 | 10.4 | 59.11 | 4.23 | 21.86 | 22.27 | 1.38 | 53.44 |
| 2019-2020 | 2 | 5 | AKBARI | 100 | 76.47 | 7.00 | 87 | 8.83 | 56.98 | 3.6 | 20.1 | 20.42 | 1.39 | 52.38 |
| 2019-2020 | 2 | 5 | 623506 | 100 | 67.12 | 6.33 | 80 | 12.09 | 71.23 | 5.63 | 23.33 | 24.00 | 1.33 | 62.53 |
| 2019-2020 | 2 | 5 | 623510 | 100 | 92.51 | 8.00 | 100 | 8.19 | 52.56 | 4.79 | 19.23 | 19.82 | 1.33 | 48.59 |
| 2019-2020 | 2 | 5 | 627845 | 100 | 90.57 | 8.00 | 100 | 10.41 | 59.68 | 3.89 | 19.21 | 19.60 | 1.37 | 55.17 |
| 2019-2020 | 2 | 6 | 627856 | 0 | 0 | 1.00 | 0 | 0.26 | 47.82 | 1.92 | 18.72 | 18.82 | 1.47 | 44.53 |
| 2019-2020 | 2 | 6 | MAHDAVI | 100 | 96.07 | 8.67 | 100 | 14.68 | 66.37 | 3.93 | 23.5 | 23.83 | 1.41 | 58.79 |
| 2019-2020 | 2 | 6 | 627360 | 100 | 94.77 | 8.33 | 100 | 14.16 | 68.38 | 3.45 | 23.1 | 23.36 | 1.42 | 60.69 |
| 2019-2020 | 2 | 6 | 627883 | 100 | 83.64 | 7.33 | 100 | 14.71 | 69.07 | 3.9 | 22.65 | 22.98 | 1.40 | 61.47 |
| 2019-2020 | 2 | 6 | SHIROODI | 100 | 98.67 | 9.00 | 100 | 14.72 | 71.15 | 1.11 | 23.08 | 23.11 | 1.52 | 63.04 |
| 2019-2020 | 2 | 6 | 623379 | 100 | 53.85 | 6.00 | 60 | 8.47 | 52.96 | 5.27 | 24.05 | 24.62 | 1.36 | 46.91 |
| 2019-2020 | 2 | 6 | 624910 | 100 | 85.26 | 7.33 | 100 | 11.89 | 66.1 | 5.16 | 24.77 | 25.30 | 1.37 | 57.70 |
| 2019-2020 | 2 | 6 | MOGHAN3 | 100 | 91.26 | 8.00 | 93 | 14.58 | 65.56 | 4.09 | 27.02 | 27.33 | 1.42 | 56.03 |
| 2019-2020 | 2 | 6 | DASTJERDI | 100 | 98.6 | 9.00 | 100 | 3.7 | 70.13 | 3.44 | 22.31 | 22.57 | 1.42 | 62.56 |
| 2019-2020 | 2 | 7 | TAKAB | 40 | 5.23 | 2.00 | 7 | 1.17 | 50.88 | 4.01 | 16.93 | 17.40 | 1.34 | 47.89 |
| 2019-2020 | 2 | 7 | 623908 | 100 | 92.72 | 8.00 | 100 | 12.89 | 61.66 | 3.29 | 17.82 | 18.12 | 1.39 | 57.59 |
| 2019-2020 | 2 | 7 | RASHID | 100 | 99.4 | 9.00 | 100 | 15.47 | 71.69 | 3.7 | 21.18 | 21.50 | 1.40 | 64.45 |
| 2019-2020 | 2 | 7 | 624900 | 100 | 91.75 | 8.33 | 100 | 14 | 67.93 | 5.58 | 24.62 | 25.24 | 1.35 | 59.19 |
| 2019-2020 | 2 | 7 | 627054 | 100 | 89.93 | 8.00 | 100 | 13.42 | 57 | 6.07 | 23.64 | 24.41 | 1.32 | 50.56 |
| 2019-2020 | 2 | 7 | 626223 | 100 | 95.87 | 8.67 | 100 | 15.38 | 64.08 | 3.61 | 22.95 | 23.23 | 1.41 | 57.22 |
| 2019-2020 | 2 | 7 | 627189 | 100 | 96.99 | 8.33 | 100 | 12.14 | 67.51 | 2.86 | 21.55 | 21.74 | 1.44 | 60.91 |
| 2019-2020 | 2 | 7 | 627423 | 100 | 94.23 | 8.33 | 100 | 15.81 | 62.82 | 5.91 | 25.5 | 26.18 | 1.34 | 54.53 |
| 2019-2020 | 2 | 7 | ZARE | 100 | 94.61 | 8.33 | 100 | 11.53 | 61.92 | 4.22 | 20.81 | 21.23 | 1.37 | 56.40 |
| 2019-2020 | 2 | 8 | 627410 | 100 | 96.12 | 8.67 | 100 | 7.92 | 54.21 | 4.59 | 18.44 | 19.00 | 1.33 | 50.42 |
| 2019-2020 | 2 | 8 | DN11 | 100 | 96.49 | 8.33 | 100 | 14.07 | 68.61 | 3.27 | 24.65 | 24.87 | 1.44 | 59.95 |
| 2019-2020 | 2 | 8 | 625080 | 100 | 87.5 | 7.67 | 100 | 9.46 | 62.25 | 4.55 | 22.2 | 22.66 | 1.37 | 55.97 |
| 2019-2020 | 2 | 8 | HOMA | 100 | 71.43 | 7.00 | 80 | 12.76 | 70.84 | 3.91 | 22.73 | 23.06 | 1.40 | 62.82 |
| 2019-2020 | 2 | 8 | 627842 | 100 | 92.26 | 8.33 | 100 | 13.83 | 59.93 | 3.91 | 20.1 | 20.48 | 1.38 | 55.00 |
| 2019-2020 | 2 | 8 | 627043 | 100 | 61.72 | 6.33 | 73 | 8.53 | 57.78 | 1.96 | 19.02 | 19.12 | 1.47 | 53.65 |
| 2019-2020 | 2 | 8 | 624956 | 100 | 92.26 | 8.33 | 93 | 14.76 | 63.87 | 5.54 | 22.93 | 23.59 | 1.33 | 56.85 |
| 2019-2020 | 2 | 8 | 626261 | 100 | 98.33 | 9.00 | 100 | 9.36 | 54.52 | 3.75 | 22.69 | 23.00 | 1.41 | 49.04 |
| 2019-2020 | 2 | 8 | 628084 | 100 | 96.62 | 8.33 | 100 | 8.42 | 50.02 | 3.23 | 16.11 | 16.43 | 1.37 | 47.39 |
| 2019-2020 | 2 | 8 | VEE/NAC | 100 | 91.89 | 8.33 | 100 | 10.61 | 54.66 | 4.78 | 21.57 | 22.09 | 1.35 | 49.56 |
| 2019-2020 | 2 | 9 | 625047 | 100 | 97.66 | 9.00 | 100 | 15.54 | 67.58 | 3.78 | 22.8 | 23.11 | 1.41 | 60.19 |
| 2019-2020 | 2 | 9 | 626158 | 100 | 96.18 | 8.67 | 100 | 8.52 | 52.63 | 1.96 | 18.45 | 18.55 | 1.46 | 49.13 |
| 2019-2020 | 2 | 9 | 624925 | 100 | 93.1 | 8.33 | 100 | 11.9 | 64.33 | 2.53 | 23.82 | 23.95 | 1.46 | 57.03 |
| 2019-2020 | 2 | 9 | 621619 | 100 | 84.09 | 7.33 | 93 | 12.93 | 63.49 | 2.19 | 19.76 | 19.88 | 1.46 | 58.43 |
| 2019-2020 | 2 | 9 | 627299 | 100 | 96.48 | 8.67 | 100 | 14.63 | 66.69 | 2.72 | 25.02 | 25.17 | 1.46 | 58.25 |
| 2019-2020 | 2 | 9 | 623291 | 100 | 89.92 | 8.00 | 100 | 7.52 | 48.89 | 3.54 | 20.06 | 20.37 | 1.40 | 44.98 |
| 2019-2020 | 2 | 9 | 627849 | 100 | 98.73 | 9.00 | 100 | 11.68 | 59.67 | 2.08 | 22.26 | 22.36 | 1.48 | 53.89 |
| 2019-2020 | 2 | 9 | 626978 | 100 | 95.52 | 8.33 | 100 | 13.31 | 62.73 | 3.21 | 23.87 | 24.08 | 1.44 | 55.63 |
| 2019-2020 | 2 | 9 | AKOVA | 100 | 97.53 | 8.67 | 100 | 10.02 | 56.07 | 5.25 | 20.35 | 21.02 | 1.32 | 51.30 |
| 2019-2020 | 2 | 9 | 627072 | 100 | 95.17 | 8.67 | 100 | 13.74 | 61.81 | 3.25 | 22.4 | 22.63 | 1.43 | 55.61 |
| 2019-2020 | 2 | 10 | 626908 | 100 | 96.67 | 8.67 | 100 | 13.38 | 57.27 | 5.14 | 22.22 | 22.81 | 1.34 | 51.56 |
| 2019-2020 | 2 | 10 | DARAB1 | 100 | 59.64 | 6.33 | 67 | 7.76 | 51.27 | 3.07 | 22.07 | 22.28 | 1.43 | 46.42 |
| 2019-2020 | 2 | 10 | 623421 | 100 | 85.12 | 7.67 | 100 | 10.55 | 56.78 | 3.66 | 19.47 | 19.81 | 1.38 | 52.46 |
| 2019-2020 | 2 | 10 | 623090 | 100 | 96.48 | 8.67 | 100 | 13.76 | 67.08 | 1.9 | 21.88 | 21.96 | 1.48 | 60.43 |
| 2019-2020 | 2 | 10 | 626234 | 100 | 97.35 | 8.67 | 100 | 9.08 | 50.73 | 4.47 | 17.4 | 17.96 | 1.32 | 47.56 |
| 2019-2020 | 2 | 10 | 627853 | 100 | 94.12 | 8.33 | 100 | 13.66 | 60.59 | 3.15 | 22.02 | 22.24 | 1.43 | 54.75 |
| 2019-2020 | 2 | 10 | GHABOUS | 100 | 98.43 | 9.00 | 100 | 10.84 | 53.69 | 1.99 | 19.54 | 19.64 | 1.47 | 49.70 |
| 2019-2020 | 2 | 10 | 624805 | 0 | 0.89 | 1.33 | 7 | 0.42 | 47.74 | 3.61 | 19.87 | 20.20 | 1.39 | 43.97 |
| 2019-2020 | 2 | 10 | 621565 | 100 | 67.28 | 6.67 | 73 | 11.46 | 60.11 | 2.94 | 23.73 | 23.91 | 1.45 | 53.49 |
| 2019-2020 | 2 | 10 | SABALAN | 100 | 96.99 | 8.67 | 100 | 13.49 | 65.93 | 2.26 | 23.12 | 23.23 | 1.47 | 58.76 |
| 2019-2020 | 2 | 11 | 626706 | 100 | 72 | 7.67 | 87 | 11.91 | 67.94 | 3.95 | 22.61 | 22.95 | 1.40 | 60.57 |
| 2019-2020 | 2 | 11 | 622063 | 100 | 93.27 | 8.33 | 100 | 8.2 | 57.43 | 2.01 | 18.84 | 18.95 | 1.46 | 53.40 |
| 2019-2020 | 2 | 11 | 626747 | 100 | 92.14 | 8.00 | 100 | 14.86 | 66.02 | 2.55 | 23.98 | 24.12 | 1.46 | 58.33 |
| 2019-2020 | 2 | 11 | 626573 | 100 | 91.25 | 8.00 | 100 | 13.03 | 65.8 | 4.13 | 25.65 | 25.98 | 1.41 | 57.05 |
| 2019-2020 | 2 | 11 | 622247 | 0 | 0 | 1.00 | 0 | 0.2 | 48.87 | 4 | 19.51 | 19.92 | 1.37 | 45.13 |
| 2019-2020 | 2 | 11 | 627990 | 100 | 96.17 | 8.67 | 100 | 12.69 | 70.66 | 5.39 | 23.35 | 23.96 | 1.34 | 62.12 |
| 2019-2020 | 2 | 11 | MAROON | 100 | 98.65 | 9.00 | 100 | 12.79 | 55.71 | 4.26 | 20.1 | 20.55 | 1.36 | 51.18 |
| 2019-2020 | 2 | 11 | 626226 | 100 | 98.63 | 8.67 | 100 | 15.22 | 66.54 | 4.16 | 22.13 | 22.52 | 1.38 | 59.67 |
| 2019-2020 | 2 | 11 | 626924 | 100 | 51.87 | 5.67 | 60 | 11.37 | 62.07 | 4.76 | 24.01 | 24.48 | 1.38 | 54.86 |
| 2019-2020 | 2 | 11 | 622084 | 0 | 0 | 1.00 | 0 | 0.32 | 48.3 | 1.84 | 16.45 | 16.55 | 1.46 | 45.71 |
| 2019-2020 | 2 | 12 | 624849 | 100 | 85.42 | 7.67 | 100 | 11.98 | 57.85 | 3.54 | 19.7 | 20.02 | 1.39 | 53.34 |
| 2019-2020 | 2 | 12 | 624576 | 100 | 98.28 | 9.00 | 100 | 8.82 | 56.71 | 2.88 | 21.75 | 21.94 | 1.44 | 51.47 |
| 2019-2020 | 2 | 12 | 626904 | 100 | 93.8 | 8.33 | 100 | 13.26 | 65.61 | 3.29 | 23.55 | 23.78 | 1.43 | 58.19 |
| 2019-2020 | 2 | 12 | 626895 | 100 | 71.54 | 6.67 | 80 | 14.18 | 66.1 | 4.63 | 25.36 | 25.78 | 1.39 | 57.41 |
| 2019-2020 | 2 | 12 | 627057 | 100 | 22.86 | 4.00 | 40 | 11.25 | 67.25 | 3.28 | 25.99 | 26.20 | 1.45 | 58.06 |
| 2019-2020 | 2 | 12 | SIRVAN | 100 | 100 | 9.00 | 100 | 2.69 | 62.76 | 2.51 | 23.77 | 23.90 | 1.47 | 55.75 |
| 2019-2020 | 2 | 12 | ALBORZ | 100 | 100 | 9.00 | 100 | 12.19 | 61.29 | 1.5 | 22.65 | 22.70 | 1.50 | 55.13 |
| 2019-2020 | 2 | 12 | 627987 | 100 | 100 | 9.00 | 100 | 11.32 | 59.74 | 1.4 | 21.18 | 21.23 | 1.50 | 54.49 |
| 2019-2020 | 2 | 12 | 624582 | 100 | 100 | 9.00 | 100 | 15.04 | 70.2 | 1.76 | 21.58 | 21.65 | 1.49 | 63.16 |
| 2019-2020 | 2 | 13 | 623909 | 100 | 100 | 9.00 | 100 | 11.69 | 55.96 | 5.09 | 20.31 | 20.94 | 1.33 | 51.24 |
| 2019-2020 | 2 | 13 | MARVDASHT | 100 | 100 | 9.00 | 100 | 13.14 | 57.02 | 4.86 | 21.4 | 21.94 | 1.35 | 51.74 |
| 2019-2020 | 2 | 13 | GAHAR | 100 | 100 | 9.00 | 100 | 0.11 | 52.09 | 3.72 | 17.86 | 18.24 | 1.37 | 48.73 |
| 2019-2020 | 2 | 13 | 627616 | 100 | 100 | 9.00 | 100 | 12.34 | 59.56 | 2.58 | 17.58 | 17.77 | 1.43 | 55.83 |
| 2019-2020 | 2 | 13 | 627102 | 60 | 6.41 | 2.00 | 7 | 2.48 | 52.38 | 5.06 | 18.1 | 18.79 | 1.30 | 48.81 |
| 2019-2020 | 2 | 13 | NAZ | 100 | 100 | 9.00 | 100 | 12.09 | 64.17 | 3.79 | 20.75 | 21.09 | 1.39 | 58.42 |
| 2019-2020 | 2 | 13 | 623109 | 100 | 95.33 | 8.33 | 100 | 16.59 | 67.16 | 5.44 | 24.28 | 24.88 | 1.35 | 58.80 |
| 2019-2020 | 2 | 13 | 623091 | 100 | 88.77 | 7.67 | 100 | 8.45 | 56.09 | 2.78 | 20.92 | 21.10 | 1.44 | 51.28 |
| 2019-2020 | 2 | 13 | BISTON | 100 | 81.25 | 7.67 | 80 | 6.63 | 55.14 | 1.87 | 20.5 | 20.59 | 1.48 | 50.64 |
| 2019-2020 | 2 | 14 | 625433 | 100 | 72.85 | 6.67 | 80 | 12.12 | 63.35 | 3.15 | 21.77 | 22.00 | 1.43 | 57.26 |
| 2019-2020 | 2 | 14 | 623161 | 100 | 81.55 | 7.33 | 93 | 9.5 | 55.92 | 2.18 | 21.56 | 21.67 | 1.47 | 50.88 |
| 2019-2020 | 2 | 14 | AZAR2 | 100 | 91.6 | 8.33 | 93 | 12.52 | 57.89 | 1.87 | 23.91 | 23.98 | 1.49 | 51.54 |
| 2019-2020 | 2 | 14 | 626699 | 100 | 89.81 | 7.67 | 93 | 10.49 | 57.47 | 2.29 | 20.55 | 20.68 | 1.46 | 52.71 |
| 2019-2020 | 2 | 14 | 627873 | 100 | 100 | 9.00 | 100 | 10.64 | 54.96 | 1.25 | 20.08 | 20.12 | 1.51 | 50.67 |
| 2019-2020 | 2 | 14 | 622379 | 100 | 100 | 9.00 | 100 | 13.92 | 65.14 | 1.8 | 22.68 | 22.75 | 1.49 | 58.37 |
| 2019-2020 | 2 | 14 | 627587 | 100 | 100 | 9.00 | 100 | 14.09 | 58.39 | 3.16 | 22.51 | 22.73 | 1.43 | 52.59 |
| 2019-2020 | 2 | 14 | 623127 | 100 | 100 | 9.00 | 100 | 13.16 | 58.29 | 3.82 | 21.9 | 22.23 | 1.40 | 52.74 |
| 2019-2020 | 2 | 14 | 623507 | 100 | 100 | 9.00 | 100 | 11.8 | 60.56 | 2.74 | 18.82 | 19.02 | 1.43 | 56.21 |
| 2019-2020 | 2 | 15 | SHIRAZ | 100 | 89.56 | 7.67 | 100 | 14.57 | 62.05 | 4.64 | 24.54 | 24.97 | 1.38 | 54.57 |
| 2019-2020 | 2 | 15 | MIHAN | 100 | 100 | 9.00 | 100 | 14.83 | 67.28 | 3.02 | 21.81 | 22.02 | 1.43 | 60.56 |
| 2019-2020 | 2 | 15 | 621650 | 100 | 98.08 | 8.67 | 100 | 14.91 | 66.11 | 4.31 | 21.03 | 21.47 | 1.37 | 59.88 |
| 2019-2020 | 2 | 15 | 627066 | 100 | 100 | 9.00 | 100 | 11.3 | 60.66 | 3.22 | 18.43 | 18.71 | 1.40 | 56.44 |
| 2019-2020 | 2 | 15 | FONG | 100 | 94.38 | 8.33 | 100 | 14.61 | 60.69 | 2.26 | 23.48 | 23.59 | 1.47 | 54.16 |
| 2019-2020 | 2 | 15 | 627787 | 100 | 100 | 9.00 | 100 | 11.2 | 61.66 | 1.6 | 18.56 | 18.63 | 1.48 | 57.37 |
| 2019-2020 | 2 | 15 | 623139 | 100 | 100 | 9.00 | 100 | 14.57 | 61.16 | 3.08 | 24.68 | 24.87 | 1.45 | 53.88 |
| 2019-2020 | 2 | 15 | 621908 | 100 | 100 | 9.00 | 100 | 15.38 | 64.58 | 3.61 | 21.72 | 22.02 | 1.41 | 58.29 |
| 2019-2020 | 2 | 16 | HAMOON | 100 | 100 | 9.00 | 100 | 11.2 | 68.35 | 1.59 | 21.94 | 22.00 | 1.50 | 61.46 |
| 2019-2020 | 2 | 16 | ALVAND | 100 | 100 | 9.00 | 100 | 14.83 | 66.39 | 3.17 | 24.29 | 24.50 | 1.44 | 58.41 |
| 2019-2020 | 2 | 16 | 624944 | 100 | 100 | 9.00 | 100 | 13.56 | 66.08 | 4.76 | 22.1 | 22.61 | 1.36 | 59.24 |
| 2019-2020 | 2 | 16 | 624846 | 100 | 98.72 | 9.00 | 100 | 10.92 | 60.92 | 3.82 | 20.02 | 20.38 | 1.38 | 55.92 |
| 2019-2020 | 2 | 16 | PISHTAZ | 100 | 100 | 9.00 | 100 | 9.71 | 64.31 | 3.12 | 20.15 | 20.39 | 1.42 | 58.90 |
| 2019-2020 | 2 | 16 | 623428 | 100 | 100 | 9.00 | 100 | 12.39 | 65.11 | 3.43 | 22.83 | 23.09 | 1.42 | 58.16 |
| 2019-2020 | 2 | 16 | 622894 | 100 | 100 | 9.00 | 100 | 11.76 | 66.47 | 4.61 | 22.21 | 22.68 | 1.37 | 59.52 |
| 2019-2020 | 2 | 16 | ARTA | 100 | 100 | 9.00 | 100 | 13.72 | 65.44 | 3.54 | 22.87 | 23.14 | 1.42 | 58.41 |
| 2019-2020 | 2 | 16 | 623162 | 100 | 85.9 | 7.67 | 87 | 16.38 | 70.83 | 5.13 | 21.06 | 21.68 | 1.33 | 63.66 |
| 2019-2020 | 2 | 17 | 628189 | 100 | 95.72 | 8.67 | 100 | 8.87 | 54.44 | 4.21 | 23.43 | 23.81 | 1.39 | 48.60 |
| 2019-2020 | 2 | 17 | INIA | 100 | 100 | 9.00 | 100 | 15.37 | 66.66 | 2.14 | 23.18 | 23.28 | 1.48 | 59.34 |
| 2019-2020 | 2 | 17 | REYHANI | 100 | 100 | 9.00 | 100 | 9.86 | 57.75 | 3.66 | 24.96 | 25.23 | 1.43 | 50.79 |
| 2019-2020 | 2 | 17 | 626215 | 100 | 65.56 | 6.33 | 73 | 7.14 | 58.1 | 2.63 | 25.59 | 25.72 | 1.47 | 50.83 |
| 2019-2020 | 2 | 17 | KARIM | 100 | 100 | 9.00 | 100 | 10.65 | 54.67 | 3.46 | 19.44 | 19.75 | 1.39 | 50.56 |
| 2019-2020 | 2 | 17 | 627414 | 100 | 77.27 | 7.00 | 87 | 13.71 | 67.78 | 3.48 | 21.93 | 22.20 | 1.41 | 60.87 |
| 2019-2020 | 2 | 17 | 627399 | 100 | 89.05 | 7.67 | 100 | 14.27 | 70.58 | 1.57 | 22.51 | 22.56 | 1.50 | 62.92 |
| 2019-2020 | 2 | 17 | 623318 | 100 | 68.91 | 6.67 | 80 | 5.61 | 56.93 | 2.39 | 17.66 | 17.82 | 1.44 | 53.39 |
| 2019-2020 | 2 | 17 | 627963 | 100 | 84.43 | 8.00 | 93 | 13.73 | 69.67 | 5.47 | 22.62 | 23.27 | 1.33 | 61.77 |
| 2019-2020 | 2 | 18 | KARAJ1 | 100 | 87.96 | 7.67 | 93 | 11.93 | 71.49 | 3.75 | 22.17 | 22.48 | 1.40 | 63.69 |
| 2019-2020 | 2 | 18 | DEZ | 100 | 97.02 | 8.33 | 100 | 10.53 | 66.17 | 3.44 | 22.38 | 22.64 | 1.42 | 59.29 |
| 2019-2020 | 2 | 18 | 621706 | 100 | 98.31 | 8.67 | 100 | 14.9 | 68.98 | 4.18 | 24.47 | 24.82 | 1.40 | 60.27 |
| 2019-2020 | 2 | 18 | 626358 | 100 | 80.15 | 7.67 | 87 | 12.87 | 65.93 | 4.62 | 24.53 | 24.96 | 1.38 | 57.76 |
| 2019-2020 | 2 | 18 | 623980 | 100 | 81.15 | 7.67 | 93 | 10.97 | 65.27 | 3.95 | 22.81 | 23.15 | 1.40 | 58.26 |
| 2019-2020 | 2 | 18 | 626776 | 100 | 69.83 | 6.67 | 73 | 7.9 | 57.74 | 1.87 | 17.49 | 17.59 | 1.46 | 54.23 |
| 2019-2020 | 2 | 18 | 625127 | 100 | 97.74 | 8.67 | 100 | 13.2 | 58.74 | 1.51 | 21.93 | 21.98 | 1.50 | 53.25 |
| 2019-2020 | 2 | 18 | CHAMRAN2 | 100 | 89.77 | 8.00 | 100 | 13.84 | 64.56 | 4.29 | 23.55 | 23.94 | 1.39 | 57.23 |
| 2019-2020 | 2 | 18 | NICKNEJAD | 100 | 48.28 | 5.33 | 60 | 2.91 | 51.23 | 4.56 | 20.54 | 21.04 | 1.35 | 46.89 |
| 2019-2020 | 2 | 18 | 623503 | 100 | 81.12 | 7.33 | 87 | 8.45 | 61.02 | 3.79 | 20.64 | 20.99 | 1.39 | 55.73 |
| 2019-2020 | 2 | 19 | FRONTANA | 0 | 0 | 1.00 | 0 | 17.69 | 68.55 | 2.41 | 25.09 | 25.21 | 1.48 | 59.70 |
| 2019-2020 | 2 | 19 | 627881 | 100 | 87.1 | 8.00 | 93 | 8.34 | 60.51 | 3.52 | 22.2 | 22.48 | 1.41 | 54.56 |
| 2019-2020 | 2 | 19 | 623377 | 100 | 100 | 9.00 | 100 | 15.46 | 73.19 | 1.77 | 23.71 | 23.78 | 1.50 | 64.17 |
| 2019-2020 | 2 | 19 | MV17 | 100 | 83.66 | 7.67 | 93 | 9.99 | 54.19 | 2.66 | 20.42 | 20.59 | 1.44 | 49.77 |
| 2019-2020 | 2 | 19 | 627948 | 100 | 86.99 | 8.00 | 93 | 13.61 | 61.38 | 3.43 | 22.18 | 22.44 | 1.42 | 55.33 |
| 2019-2020 | 2 | 19 | BAHAR | 100 | 100 | 9.00 | 100 | 12.69 | 53.77 | 3.11 | 21.58 | 21.80 | 1.43 | 48.89 |
| 2019-2020 | 2 | 19 | 624818 | 100 | 58.06 | 6.00 | 60 | 4.97 | 47.46 | 2.53 | 17.76 | 17.94 | 1.43 | 44.48 |
| 2019-2020 | 2 | 19 | 623345 | 100 | 79.41 | 7.33 | 87 | 11.58 | 68.11 | 4.09 | 22.81 | 23.17 | 1.39 | 60.58 |
| 2019-2020 | 2 | 20 | 627908 | 100 | 87.5 | 8.00 | 93 | 9.35 | 60.21 | 4.73 | 20.67 | 21.20 | 1.35 | 54.91 |
| 2019-2020 | 2 | 20 | 623169 | 100 | 12.96 | 3.33 | 20 | 2.77 | 56.93 | 3.84 | 21.63 | 21.97 | 1.40 | 51.65 |
| 2019-2020 | 2 | 20 | ATRAK | 100 | 82.94 | 7.33 | 87 | 6.31 | 52.95 | 3.88 | 21.33 | 21.68 | 1.39 | 48.20 |
| 2019-2020 | 2 | 20 | 627356 | 100 | 93.03 | 8.00 | 100 | 10.19 | 60.85 | 2.77 | 22.14 | 22.31 | 1.45 | 54.94 |
| 2019-2020 | 2 | 20 | 626943 | 100 | 40.83 | 5.33 | 53 | 4.92 | 44.79 | 2.45 | 18.99 | 19.15 | 1.44 | 41.56 |
| 2019-2020 | 2 | 20 | 627417 | 100 | 45.57 | 5.33 | 60 | 7.16 | 58.05 | 2.81 | 17.9 | 18.12 | 1.42 | 54.30 |
| 2019-2020 | 2 | 20 | 628088 | 100 | 93.09 | 8.33 | 93 | 11.28 | 65.15 | 2.44 | 20.71 | 20.85 | 1.45 | 59.39 |
| 2019-2020 | 2 | 20 | BAYAT | 100 | 71.77 | 6.67 | 73 | 13.45 | 64.14 | 2.39 | 23.91 | 24.03 | 1.47 | 56.83 |
| 2019-2020 | 2 | 20 | FALAT | 100 | 100 | 9.00 | 100 | 13.32 | 58.02 | 4.33 | 22.21 | 22.63 | 1.38 | 52.31 |
| 2019-2020 | 2 | 20 | 626933 | 100 | 100 | 9.00 | 100 | 9.64 | 56.81 | 2.86 | 23.46 | 23.63 | 1.45 | 50.77 |
| 2019-2020 | 2 | 21 | 623125 | 100 | 100 | 9.00 | 100 | 11 | 60.27 | 4.49 | 22.88 | 23.32 | 1.38 | 53.93 |
| 2019-2020 | 2 | 21 | DARAB2 | 100 | 85.05 | 7.67 | 93 | 7.77 | 41.51 | 3.85 | 17.7 | 18.11 | 1.36 | 38.77 |
| 2019-2020 | 2 | 21 | 624980 | 100 | 61.83 | 6.33 | 73 | 10.39 | 60.35 | 4.9 | 21.95 | 22.49 | 1.35 | 54.42 |
| 2019-2020 | 2 | 21 | 625281 | 100 | 28.81 | 4.33 | 33 | 2.84 | 54.45 | 2.06 | 18.63 | 18.74 | 1.46 | 50.74 |
| 2019-2020 | 2 | 21 | TAJAN | 100 | 93.33 | 8.33 | 100 | 12.21 | 56.6 | 4.14 | 20.07 | 20.49 | 1.37 | 52.01 |
| 2019-2020 | 2 | 21 | 623382 | 100 | 30.61 | 4.67 | 40 | 11.06 | 64.69 | 3.46 | 25.26 | 25.50 | 1.43 | 56.45 |
| 2019-2020 | 2 | 21 | 624580 | 80 | 12.14 | 2.67 | 20 | 1.32 | 48.4 | 3.57 | 17.77 | 18.13 | 1.37 | 45.31 |
| 2019-2020 | 2 | 21 | KAVIR | 100 | 56.86 | 5.67 | 67 | 8.39 | 63.47 | 3.04 | 22.77 | 22.97 | 1.44 | 56.85 |
| 2019-2020 | 2 | 21 | 626883 | 100 | 75.66 | 7.00 | 80 | 15.42 | 72.71 | 1.08 | 23.14 | 23.17 | 1.52 | 64.20 |
| 2019-2020 | 2 | 21 | BAM | 100 | 89.84 | 8.00 | 93 | 11.88 | 64.46 | 2.33 | 24.35 | 24.46 | 1.48 | 56.86 |
| 2019-2020 | 2 | 22 | 623136 | 100 | 72.55 | 6.67 | 80 | 8.69 | 60.07 | 3.55 | 20.34 | 20.65 | 1.40 | 55.05 |
| 2019-2020 | 2 | 22 | KAVEH | 100 | 100 | 9.00 | 100 | 12.54 | 55.86 | 2.64 | 23.38 | 23.53 | 1.46 | 49.98 |
| 2019-2020 | 2 | 22 | 626923 | 100 | 42.11 | 5.33 | 53 | 6.66 | 67.45 | 2.34 | 22.78 | 22.90 | 1.47 | 60.20 |
| 2019-2020 | 2 | 22 | MOGHAN1 | 100 | 87.27 | 8.00 | 93 | 6.67 | 58.34 | 2.62 | 19.02 | 19.20 | 1.43 | 54.13 |
| 2019-2020 | 2 | 22 | 627099 | 100 | 100 | 9.00 | 100 | 9.14 | 53.55 | 1.41 | 17.54 | 17.60 | 1.49 | 50.33 |
| 2019-2020 | 2 | 22 | 626260 | 100 | 32.56 | 5.00 | 47 | 3.4 | 50.16 | 3.76 | 17.97 | 18.36 | 1.36 | 46.89 |
| 2019-2020 | 2 | 22 | DARYA | 100 | 31.51 | 5.00 | 40 | 10.42 | 54.19 | 4.08 | 20.96 | 21.35 | 1.38 | 49.46 |
| 2019-2020 | 2 | 22 | 623176 | 100 | 38.46 | 5.33 | 47 | 4.52 | 56.79 | 3.07 | 21.66 | 21.88 | 1.43 | 51.57 |
| 2019-2020 | 2 | 22 | 621492 | 100 | 20.71 | 4.00 | 27 | 3.36 | 61.93 | 4.16 | 22.63 | 23.01 | 1.39 | 55.52 |
| 2019-2020 | 2 | 22 | URUOM | 100 | 59.52 | 6.00 | 67 | 11.06 | 61.32 | 3.14 | 22.57 | 22.79 | 1.43 | 55.11 |
| 2019-2020 | 2 | 23 | 627723 | 100 | 100 | 9.00 | 100 | 10.44 | 60.57 | 4.42 | 20.26 | 20.74 | 1.36 | 55.45 |
| 2019-2020 | 2 | 23 | BEZOSTAYA | 60 | 8.97 | 2.67 | 13 | 1.4 | 55.86 | 5.45 | 20.25 | 20.97 | 1.31 | 51.13 |
| 2019-2020 | 2 | 23 | 626565 | 100 | 90.52 | 8.00 | 93 | 8.57 | 54.11 | 2.63 | 17.52 | 17.72 | 1.42 | 50.81 |
| 2019-2020 | 2 | 23 | 4820 | 100 | 48.8 | 5.67 | 60 | 10.03 | 65.45 | 2.51 | 22.52 | 22.66 | 1.46 | 58.68 |
| 2019-2020 | 2 | 23 | OFOG | 100 | 54.21 | 6.00 | 60 | 11.25 | 62.7 | 2.71 | 22.87 | 23.03 | 1.45 | 56.16 |
| 2019-2020 | 2 | 23 | 624946 | 100 | 17.46 | 3.67 | 27 | 2.02 | 62.42 | 1.97 | 22.66 | 22.75 | 1.48 | 56.07 |
| 2019-2020 | 2 | 23 | 624894 | 100 | 48.67 | 5.67 | 60 | 5.71 | 52.67 | 1.7 | 16.21 | 16.30 | 1.47 | 49.94 |
| 2019-2020 | 2 | 23 | AZAR1 | 100 | 27.11 | 4.67 | 33 | 4.24 | 48.34 | 2.92 | 18.29 | 18.52 | 1.41 | 45.12 |
| 2019-2020 | 2 | 23 | 626958 | 100 | 42.31 | 5.33 | 53 | 6.61 | 67.69 | 1.49 | 23.55 | 23.60 | 1.51 | 59.99 |
| 2019-2020 | 2 | 23 | 623417 | 100 | 61.43 | 6.33 | 67 | 8.67 | 62.49 | 2.25 | 17.21 | 17.36 | 1.44 | 58.67 |
| 2019-2020 | 2 | 24 | 624378 | 100 | 41.24 | 5.33 | 47 | 3.31 | 56.73 | 3.65 | 19.79 | 20.12 | 1.39 | 52.28 |
| 2019-2020 | 2 | 24 | SISTAN | 100 | 84 | 7.33 | 93 | 8.1 | 53.95 | 3.3 | 17.5 | 17.81 | 1.38 | 50.63 |
| 2019-2020 | 2 | 24 | 626872 | 100 | 100 | 9.00 | 100 | 5.97 | 49.12 | 2.97 | 15.19 | 15.48 | 1.38 | 46.82 |
| 2019-2020 | 2 | 24 | 624381 | 100 | 72.12 | 7.00 | 80 | 6.68 | 68.38 | 3.33 | 22.27 | 22.52 | 1.42 | 61.18 |
| 2019-2020 | 2 | 24 | 625810 | 100 | 33.05 | 4.67 | 47 | 4.35 | 61.19 | 3.59 | 21.62 | 21.92 | 1.41 | 55.43 |
| 2019-2020 | 2 | 24 | 622311 | 100 | 54.27 | 5.67 | 60 | 8.87 | 59.82 | 3.2 | 18.01 | 18.29 | 1.39 | 55.85 |
| 2019-2020 | 2 | 24 | 627385 | 100 | 98 | 8.67 | 100 | 11.71 | 66.79 | 3.64 | 22.6 | 22.89 | 1.41 | 59.66 |
| 2019-2020 | 2 | 24 | 623905 | 100 | 58.12 | 6.33 | 67 | 4.93 | 50.42 | 3.7 | 18.03 | 18.41 | 1.37 | 47.11 |
| 2019-2020 | 2 | 24 | 627460 | 100 | 100 | 9.00 | 100 | 12.97 | 65.13 | 4.07 | 20.79 | 21.18 | 1.38 | 59.20 |
| 2019-2020 | 2 | 25 | 627760 | 80 | 6.08 | 2.33 | 13 | 5.91 | 65.94 | 2.25 | 24.71 | 24.81 | 1.48 | 57.86 |
| 2019-2020 | 2 | 25 | 625362 | 100 | 70.59 | 6.33 | 73 | 9.57 | 57.75 | 3.01 | 24.47 | 24.65 | 1.45 | 51.08 |
| 2019-2020 | 2 | 25 | 623274 | 100 | 75.68 | 7.00 | 87 | 11.77 | 59.39 | 3.13 | 21.65 | 21.88 | 1.43 | 53.87 |
| 2019-2020 | 2 | 25 | 626736 | 100 | 40 | 5.00 | 47 | 7.64 | 57.92 | 2.48 | 21.05 | 21.20 | 1.45 | 52.88 |
| 2019-2020 | 2 | 25 | 624585 | 100 | 18.79 | 4.00 | 20 | 1.54 | 51.16 | 4.13 | 18.4 | 18.86 | 1.35 | 47.65 |
| 2019-2020 | 2 | 25 | KOOHDASHT | 100 | 100 | 9.00 | 100 | 11.98 | 65.86 | 1.88 | 25.5 | 25.57 | 1.50 | 57.35 |
| 2019-2020 | 2 | 25 | NAVID | 100 | 100 | 9.00 | 100 | 12.79 | 67.08 | 1.5 | 22.93 | 22.98 | 1.51 | 59.85 |
| 2019-2020 | 2 | 25 | KARAJ2 | 100 | 100 | 9.00 | 100 | 14.38 | 66.88 | 1.18 | 23.26 | 23.29 | 1.52 | 59.51 |
| 2019-2020 | 2 | 25 | CHAMRAN | 100 | 95.68 | 8.67 | 100 | 0.12 | 53.34 | 1.57 | 16.1 | 16.18 | 1.47 | 50.62 |
| 2019-2020 | 2 | 25 | 624864 | 100 | 29.58 | 4.33 | 33 | 6.18 | 61.99 | 3.74 | 20.15 | 20.49 | 1.39 | 56.82 |
| 2019-2020 | 2 | 26 | 625123 | 100 | 92.95 | 8.33 | 93 | 11.84 | 58.17 | 2.66 | 20.04 | 20.22 | 1.44 | 53.54 |
| 2019-2020 | 2 | 26 | CASGOGEN | 20 | 4.1 | 1.67 | 7 | 9.11 | 51.54 | 2.65 | 17.62 | 17.82 | 1.42 | 48.37 |
| 2019-2020 | 2 | 26 | 626764 | 100 | 35.71 | 5.00 | 47 | 4.04 | 53.58 | 3.1 | 17.5 | 17.77 | 1.40 | 50.29 |
| 2019-2020 | 2 | 26 | 624804 | 100 | 42.86 | 5.33 | 47 | 5.25 | 59.41 | 3.6 | 18.55 | 18.90 | 1.38 | 55.23 |
| 2019-2020 | 2 | 26 | 626846 | 100 | 100 | 9.00 | 100 | 6.49 | 49.05 | 3.58 | 16.46 | 16.84 | 1.36 | 46.34 |
| 2019-2020 | 2 | 26 | ZARRIN | 100 | 52.63 | 6.00 | 60 | 3.98 | 56.18 | 4.61 | 19.75 | 20.28 | 1.34 | 51.71 |
| 2019-2020 | 2 | 26 | 626814 | 100 | 55.77 | 6.00 | 67 | 8.8 | 57.71 | 2.42 | 21.73 | 21.86 | 1.46 | 52.39 |
| 2019-2020 | 2 | 26 | 626825 | 100 | 65.97 | 6.67 | 73 | 12.16 | 60.03 | 2.64 | 22.59 | 22.74 | 1.45 | 54.01 |
| 2019-2020 | 2 | 26 | DEYHEM | 100 | 69.12 | 6.67 | 80 | 8.48 | 49.93 | 3.58 | 17.2 | 17.57 | 1.37 | 46.94 |
| 2019-2020 | 2 | 26 | 624251 | 100 | 42.03 | 5.00 | 53 | 5.66 | 57.3 | 2.84 | 21.11 | 21.30 | 1.44 | 52.28 |
| 2019-2020 | 2 | 27 | 623953 | 20 | 2.59 | 1.67 | 7 | 0.23 | 67.16 | 2.12 | 26.1 | 26.19 | 1.49 | 58.00 |
| 2019-2020 | 2 | 27 | 626855 | 100 | 100 | 9.00 | 100 | 14.89 | 64.82 | 2.51 | 24.94 | 25.07 | 1.47 | 56.80 |
| 2019-2020 | 2 | 27 | 623008 | 100 | 100 | 9.00 | 100 | 12.96 | 57.12 | 2.86 | 21.84 | 22.03 | 1.44 | 51.79 |
| 2019-2020 | 2 | 27 | 621669 | 100 | 98.8 | 9.00 | 100 | 12.36 | 58.49 | 4.55 | 18.9 | 19.44 | 1.33 | 54.16 |
| 2019-2020 | 2 | 27 | SHARYAR | 100 | 100 | 9.00 | 100 | 9.01 | 63.94 | 4.74 | 23.94 | 24.40 | 1.38 | 56.46 |
| 2019-2020 | 2 | 27 | ADL | 100 | 100 | 9.00 | 100 | 9.26 | 62.87 | 2.15 | 16.57 | 16.71 | 1.44 | 59.28 |
| 2019-2020 | 2 | 27 | PANJAMO62 | 100 | 100 | 9.00 | 100 | 13.23 | 73.02 | 3.36 | 22.54 | 22.79 | 1.42 | 64.68 |
| 2019-2020 | 2 | 27 | 624939 | 100 | 91.23 | 8.33 | 93 | 10.3 | 64.08 | 4.03 | 19.64 | 20.05 | 1.37 | 58.86 |
| 2019-2020 | 2 | 27 | PISHGAM | 100 | 95.93 | 8.67 | 100 | 12.44 | 67.76 | 4.21 | 23.27 | 23.65 | 1.39 | 60.02 |
| 2019-2020 | 2 | 27 | 626360 | 100 | 100 | 9.00 | 100 | 11.46 | 60.51 | 4.39 | 21.26 | 21.71 | 1.37 | 54.94 |
| 2019-2020 | 2 | 28 | 624983 | 100 | 51.82 | 5.67 | 60 | 7.8 | 64.3 | 5.04 | 23.58 | 24.11 | 1.36 | 56.92 |
| 2019-2020 | 2 | 28 | SIVAND | 100 | 83.77 | 7.33 | 93 | 12.95 | 60.98 | 4.49 | 23.72 | 24.14 | 1.38 | 54.12 |
| 2019-2020 | 2 | 28 | 627551 | 80 | 4.67 | 2.33 | 13 | 0.56 | 45.5 | 4.76 | 18.6 | 19.20 | 1.32 | 42.22 |
| 2019-2020 | 2 | 28 | SHAHI | 100 | 81.48 | 7.00 | 100 | 11.36 | 55.61 | 4.51 | 22.7 | 23.14 | 1.37 | 49.94 |
| 2019-2020 | 2 | 28 | 621717 | 100 | 26.37 | 4.33 | 33 | 2.99 | 60.69 | 2.34 | 23.72 | 23.84 | 1.47 | 54.03 |
| 2019-2020 | 2 | 28 | ARVAND | 100 | 100 | 9.00 | 100 | 10.75 | 55.99 | 2.17 | 18.97 | 19.09 | 1.46 | 52.03 |
| 2019-2020 | 2 | 28 | MORVARID | 100 | 100 | 9.00 | 100 | 9.46 | 57.87 | 2.15 | 22.74 | 22.84 | 1.48 | 52.08 |
| 2019-2020 | 2 | 28 | 624863 | 100 | 9.84 | 3.00 | 20 | 2.04 | 63.01 | 2.32 | 22.22 | 22.34 | 1.47 | 56.79 |
| 2019-2020 | 2 | 28 | 626881 | 100 | 87.82 | 7.67 | 93 | 10.87 | 64.54 | 3.78 | 24.19 | 24.48 | 1.42 | 56.91 |
| 2019-2020 | 2 | 28 | TOUBARI | 100 | 52.03 | 6.00 | 60 | 7.54 | 59.14 | 1.98 | 20.2 | 20.30 | 1.47 | 54.38 |
| 2019-2020 | 2 | 29 | SHINGHAI | 100 | 100 | 9.00 | 100 | 10.94 | 55.63 | 5.14 | 19.68 | 20.34 | 1.32 | 51.19 |
| 2019-2020 | 2 | 29 | KARAJ3 | 100 | 56.46 | 6.00 | 60 | 8.92 | 60.79 | 4.77 | 20.68 | 21.22 | 1.34 | 55.41 |
| 2019-2020 | 2 | 29 | 624947 | 100 | 62.21 | 6.33 | 67 | 7.17 | 56.17 | 4.48 | 19.32 | 19.83 | 1.34 | 51.89 |
| 2019-2020 | 2 | 29 | GOLESTAN | 100 | 84.72 | 7.67 | 93 | 11.49 | 64.6 | 3.11 | 18.64 | 18.90 | 1.41 | 59.87 |
| 2019-2020 | 2 | 29 | 624963 | 100 | 44.5 | 5.33 | 53 | 5.45 | 55.78 | 4 | 21.2 | 21.57 | 1.38 | 50.80 |
| 2019-2020 | 2 | 29 | AZADI | 100 | 100 | 9.00 | 100 | 11.41 | 56.23 | 3.14 | 20.38 | 20.62 | 1.42 | 51.62 |
| 2019-2020 | 2 | 29 | 623123 | 100 | 100 | 9.00 | 100 | 14.16 | 59.92 | 4.22 | 25.67 | 26.01 | 1.41 | 52.22 |
| 2019-2020 | 2 | 29 | AFLAK | 100 | 100 | 9.00 | 100 | 17.64 | 68.1 | 1.85 | 24.98 | 25.05 | 1.50 | 59.44 |
| 2019-2020 | 2 | 29 | KHAZAR1 | 100 | 86.71 | 7.67 | 93 | 11.6 | 59.62 | 1.58 | 21.92 | 21.98 | 1.50 | 54.03 |
| 2019-2020 | 2 | 30 | 623069 | 100 | 70.97 | 6.67 | 80 | 8.82 | 68.13 | 2.64 | 23.2 | 23.35 | 1.46 | 60.49 |
| 2019-2020 | 2 | 30 | ZAGROS | 100 | 88.82 | 8.00 | 93 | 8.12 | 61.97 | 1.83 | 20.66 | 20.74 | 1.48 | 56.68 |
| 2019-2020 | 2 | 30 | 624901 | 100 | 77.54 | 7.33 | 87 | 5.27 | 55.38 | 2.33 | 18.59 | 18.74 | 1.45 | 51.61 |
| 2019-2020 | 2 | 30 | SISON | 100 | 20 | 3.67 | 27 | 10.9 | 66.71 | 2.73 | 22.93 | 23.09 | 1.45 | 59.49 |
| 2019-2020 | 2 | 30 | 624215 | 0 | 0 | 1.00 | 0 | 0.32 | 47.51 | 1.98 | 18.47 | 18.58 | 1.46 | 44.32 |
| 2019-2020 | 2 | 30 | GHODS | 100 | 100 | 9.00 | 100 | 11.18 | 57.7 | 3.48 | 20.47 | 20.76 | 1.40 | 52.88 |
| 2019-2020 | 2 | 30 | 624861 | 100 | 42.36 | 5.00 | 60 | 9.77 | 70.12 | 2.93 | 21.99 | 22.18 | 1.44 | 62.78 |
| 2019-2020 | 2 | 30 | RIJAW | 100 | 99.02 | 9.00 | 100 | 15.33 | 66.08 | 3.95 | 23.46 | 23.79 | 1.40 | 58.57 |
| 2019-2020 | 2 | 30 | SPAHAN | 100 | 100 | 9.00 | 100 | 9.65 | 58.01 | 2.61 | 21.27 | 21.43 | 1.45 | 52.86 |
| 2019-2020 | 2 | 31 | 627055 | 100 | 37.32 | 5.33 | 47 | 6.15 | 58.87 | 3.93 | 18.69 | 19.10 | 1.36 | 54.65 |
| 2019-2020 | 2 | 31 | 626932 | 100 | 38.19 | 5.00 | 53 | 10.05 | 71.09 | 4.03 | 23.27 | 23.62 | 1.40 | 62.67 |
| 2019-2020 | 2 | 31 | PARSI | 100 | 100 | 9.00 | 100 | 15.68 | 68.45 | 4.14 | 21.48 | 21.88 | 1.38 | 61.61 |
| 2019-2020 | 2 | 31 | 624838 | 100 | 22.54 | 4.33 | 33 | 7.21 | 62.75 | 4.65 | 22.68 | 23.15 | 1.37 | 56.14 |
| 2019-2020 | 2 | 31 | 621736 | 100 | 25.38 | 4.33 | 33 | 3.91 | 63.34 | 2.38 | 23.63 | 23.75 | 1.47 | 56.32 |
| 2019-2020 | 2 | 31 | 621869 | 80 | 14.21 | 3.00 | 20 | 6.94 | 66.47 | 2.17 | 22.12 | 22.23 | 1.47 | 59.77 |
| 2019-2020 | 2 | 31 | 628012 | 80 | 13.3 | 3.00 | 13 | 1.47 | 57.65 | 2.83 | 18.62 | 18.83 | 1.42 | 53.65 |
| 2019-2020 | 2 | 31 | 622099 | 0 | 0 | 1.00 | 0 | 0.14 | 52.52 | 1.49 | 18.75 | 18.81 | 1.49 | 48.93 |
| 2019-2020 | 2 | 31 | 622105 | 0 | 0 | 1.00 | 0 | 0.11 | 47.63 | 5.77 | 19.66 | 20.49 | 1.29 | 43.76 |
| 2019-2020 | 2 | 32 | 621420 | 100 | 39.47 | 5.33 | 47 | 4.1 | 58.04 | 2.78 | 20.53 | 20.72 | 1.44 | 53.20 |
| 2019-2020 | 2 | 32 | 623266 | 100 | 28.9 | 4.67 | 40 | 1.92 | 54.32 | 2.25 | 20.86 | 20.98 | 1.46 | 49.73 |
| 2019-2020 | 2 | 32 | 621712 | 100 | 77.98 | 7.00 | 73 | 7.53 | 51.6 | 4.75 | 18.11 | 18.72 | 1.31 | 48.10 |
| 2019-2020 | 2 | 32 | 620903 | 100 | 43.28 | 5.33 | 47 | 5.13 | 50.05 | 1.55 | 16.21 | 16.28 | 1.48 | 47.46 |
| 2019-2020 | 2 | 32 | 625139 | 100 | 38.24 | 5.00 | 53 | 3.18 | 56.03 | 2.34 | 14.66 | 14.85 | 1.41 | 53.59 |
| 2019-2020 | 2 | 32 | 622098 | 100 | 68.42 | 7.33 | 80 | 11.39 | 71.18 | 4.23 | 22.69 | 23.08 | 1.39 | 63.08 |
| 2019-2020 | 2 | 32 | 625263 | 20 | 4.22 | 1.67 | 7 | 0.4 | 56.14 | 1.47 | 18.46 | 18.52 | 1.49 | 52.39 |
| 2019-2020 | 2 | 32 | 622264 | 0 | 0 | 1.00 | 0 | 0.12 | 51.42 | 4.83 | 19.48 | 20.07 | 1.33 | 47.44 |
| 2019-2020 | 2 | 32 | 622272 | 0 | 0 | 1.00 | 0 | 0.12 | 51.91 | 2.35 | 18.5 | 18.65 | 1.44 | 48.42 |
| 2019-2020 | 2 | 32 | 627905 | 0 | 0 | 1.00 | 0 | 1.19 | 54.92 | 1.23 | 15.75 | 15.80 | 1.49 | 52.23 |
| 2020-2021 | 1 | 1 | 627036 | 100 | 81.25 | 7.67 | 92 | 13.34 | 62.17 | 3.51 | 24.48 | 24.73 | 1.43 | 54.80 |
| 2020-2021 | 1 | 1 | 626156 | 100 | 61.64 | 6.40 | 76 | 7.53 | 54.76 | 3.68 | 21.23 | 21.55 | 1.40 | 49.89 |
| 2020-2021 | 1 | 1 | ROSHAN | 100 | 93.26 | 8.27 | 98 | 11.31 | 53.12 | 4.51 | 21.32 | 21.79 | 1.36 | 48.30 |
| 2020-2021 | 1 | 1 | 627852 | 100 | 89.66 | 8.10 | 88 | 12.8 | 62.84 | 3.7 | 22.75 | 23.05 | 1.41 | 56.27 |
| 2020-2021 | 1 | 1 | 628114 | 100 | 86.65 | 7.93 | 98 | 13.28 | 62.61 | 3.31 | 22.57 | 22.81 | 1.43 | 56.20 |
| 2020-2021 | 1 | 1 | 624985 | 100 | 61.99 | 6.40 | 70 | 9.93 | 52.59 | 5.46 | 20.91 | 21.61 | 1.32 | 47.90 |
| 2020-2021 | 1 | 1 | 624837 | 100 | 66.37 | 6.57 | 67 | 12.2 | 69.71 | 3.39 | 23.61 | 23.85 | 1.43 | 61.45 |
| 2020-2021 | 1 | 1 | TOUS | 100 | 44.89 | 5.27 | 63 | 5.91 | 52.78 | 4.26 | 20.63 | 21.07 | 1.37 | 48.29 |
| 2020-2021 | 1 | 1 | 623473 | 100 | 58.02 | 6.03 | 69 | 10.28 | 56.58 | 4.09 | 21.19 | 21.58 | 1.38 | 51.51 |
| 2020-2021 | 1 | 2 | MOGHAN2 | 100 | 76.17 | 7.27 | 88 | 13.75 | 64.64 | 4 | 23.98 | 24.31 | 1.41 | 57.09 |
| 2020-2021 | 1 | 2 | 627359 | 100 | 84.38 | 7.50 | 92 | 9.72 | 57.24 | 3.98 | 21.05 | 21.42 | 1.38 | 52.17 |
| 2020-2021 | 1 | 2 | 627038 | 100 | 94.7 | 8.50 | 100 | 11.36 | 60.99 | 5.19 | 21.09 | 21.72 | 1.33 | 55.35 |
| 2020-2021 | 1 | 2 | 621704 | 100 | 66.51 | 6.53 | 77 | 5.53 | 46.15 | 1.46 | 17.5 | 17.56 | 1.49 | 43.36 |
| 2020-2021 | 1 | 2 | 626566 | 100 | 57.42 | 5.97 | 63 | 6.7 | 52.94 | 4.37 | 19.6 | 20.08 | 1.35 | 48.83 |
| 2020-2021 | 1 | 2 | SHAHPASSAND | 100 | 84.02 | 7.73 | 93 | 18.51 | 67.46 | 3.87 | 22.66 | 22.99 | 1.40 | 60.16 |
| 2020-2021 | 1 | 2 | 623508 | 100 | 77.12 | 7.10 | 84 | 10.27 | 63.69 | 4.34 | 23.73 | 24.12 | 1.39 | 56.41 |
| 2020-2021 | 1 | 2 | 621735 | 100 | 87.1 | 7.83 | 93 | 8.17 | 50.96 | 1.96 | 20.15 | 20.25 | 1.47 | 46.95 |
| 2020-2021 | 1 | 2 | 623338 | 100 | 99.71 | 8.83 | 100 | 15.29 | 58.64 | 3.09 | 24.24 | 24.44 | 1.44 | 51.96 |
| 2020-2021 | 1 | 2 | 621668 | 100 | 90.63 | 8.07 | 93 | 9.36 | 54.12 | 3.13 | 18.25 | 18.52 | 1.40 | 50.52 |
| 2020-2021 | 1 | 3 | 621421 | 100 | 75.91 | 6.97 | 89 | 7.63 | 55.5 | 1.44 | 16.31 | 16.37 | 1.48 | 52.58 |
| 2020-2021 | 1 | 3 | 624240 | 100 | 80.97 | 7.47 | 88 | 9.62 | 57.18 | 4.12 | 20.61 | 21.02 | 1.37 | 52.30 |
| 2020-2021 | 1 | 3 | 623344 | 100 | 75.16 | 7.13 | 94 | 12.48 | 62.33 | 3.7 | 24.23 | 24.51 | 1.42 | 55.06 |
| 2020-2021 | 1 | 3 | 624941 | 0 | 0 | 1.00 | 10 | 0.14 | 51.73 | 4.07 | 16.72 | 17.21 | 1.33 | 48.75 |
| 2020-2021 | 1 | 3 | 621716 | 100 | 85.59 | 7.40 | 98 | 15.29 | 65.14 | 6.9 | 25.24 | 26.17 | 1.30 | 56.41 |
| 2020-2021 | 1 | 3 | 627236 | 100 | 95.26 | 8.57 | 100 | 14.32 | 67.29 | 4.33 | 22.06 | 22.48 | 1.38 | 60.31 |
| 2020-2021 | 1 | 3 | 624911 | 100 | 90.58 | 8.17 | 96 | 12.93 | 65.89 | 2.41 | 21.24 | 21.38 | 1.46 | 59.75 |
| 2020-2021 | 1 | 4 | OHADI | 100 | 88.67 | 7.80 | 98 | 16.38 | 70.77 | 4.68 | 22.22 | 22.71 | 1.36 | 62.99 |
| 2020-2021 | 1 | 4 | 624990 | 100 | 58.03 | 5.97 | 73 | 9.06 | 63.58 | 4.01 | 24.44 | 24.77 | 1.41 | 55.96 |
| 2020-2021 | 1 | 4 | 627484 | 100 | 89.19 | 8.00 | 93 | 10.34 | 66.41 | 6.19 | 19.99 | 20.93 | 1.27 | 60.42 |
| 2020-2021 | 1 | 4 | 627688 | 100 | 98.76 | 9.00 | 100 | 7.38 | 54.42 | 2.36 | 17.07 | 17.23 | 1.43 | 51.27 |
| 2020-2021 | 1 | 4 | 627416 | 100 | 99.38 | 9.00 | 100 | 14.59 | 73.6 | 3.75 | 20.49 | 20.83 | 1.39 | 66.37 |
| 2020-2021 | 1 | 4 | 625661 | 100 | 94.68 | 8.43 | 100 | 8.69 | 50.9 | 1.61 | 16.87 | 16.95 | 1.48 | 48.06 |
| 2020-2021 | 1 | 4 | 625081 | 100 | 81.48 | 7.43 | 88 | 9.9 | 61.98 | 4.42 | 22.42 | 22.85 | 1.38 | 55.64 |
| 2020-2021 | 1 | 4 | 627061 | 100 | 94.59 | 8.37 | 100 | 13.47 | 63.79 | 3.63 | 20.74 | 21.06 | 1.40 | 58.11 |
| 2020-2021 | 1 | 4 | NEISHABOUR | 100 | 79.85 | 7.30 | 86 | 14.25 | 68.99 | 4.26 | 22.45 | 22.85 | 1.38 | 61.48 |
| 2020-2021 | 1 | 4 | 624315 | 100 | 83.58 | 7.50 | 86 | 11.79 | 67.53 | 3.99 | 25.01 | 25.33 | 1.41 | 58.82 |
| 2020-2021 | 1 | 5 | 627103 | 50 | 4.4 | 2.00 | 18 | 0.57 | 48.25 | 2.03 | 16.39 | 16.52 | 1.45 | 45.68 |
| 2020-2021 | 1 | 5 | 623475 | 100 | 81.23 | 7.63 | 90 | 9.41 | 57.6 | 3.4 | 20.48 | 20.76 | 1.41 | 52.79 |
| 2020-2021 | 1 | 5 | 624596 | 100 | 76.33 | 7.00 | 92 | 8.92 | 58.41 | 4.19 | 21.69 | 22.09 | 1.38 | 52.91 |
| 2020-2021 | 1 | 5 | AKBARI | 100 | 73.3 | 6.90 | 81 | 7.05 | 59.8 | 3.6 | 20.73 | 21.04 | 1.40 | 54.63 |
| 2020-2021 | 1 | 5 | 623506 | 100 | 80.91 | 7.37 | 90 | 13.21 | 68.86 | 5.54 | 22.5 | 23.17 | 1.33 | 61.18 |
| 2020-2021 | 1 | 5 | 623510 | 100 | 91.21 | 8.20 | 98 | 7.96 | 51.3 | 4.82 | 20.33 | 20.89 | 1.34 | 47.01 |
| 2020-2021 | 1 | 5 | 627845 | 100 | 93.79 | 8.40 | 98 | 10.99 | 59.03 | 3.89 | 20.31 | 20.68 | 1.38 | 54.11 |
| 2020-2021 | 1 | 6 | 627856 | 40 | 2.02 | 1.60 | 14 | 0.2 | 47.84 | 1.98 | 19.03 | 19.13 | 1.47 | 44.44 |
| 2020-2021 | 1 | 6 | MAHDAVI | 100 | 96.73 | 8.73 | 100 | 16.65 | 66.83 | 3.91 | 24.21 | 24.52 | 1.41 | 58.75 |
| 2020-2021 | 1 | 6 | 627360 | 100 | 96.31 | 8.57 | 100 | 16.22 | 65.59 | 3.24 | 22.44 | 22.67 | 1.43 | 58.79 |
| 2020-2021 | 1 | 6 | 627883 | 100 | 86.98 | 7.77 | 96 | 12.15 | 68.98 | 4.02 | 21.87 | 22.24 | 1.39 | 61.83 |
| 2020-2021 | 1 | 6 | SHIROODI | 100 | 97.77 | 8.80 | 98 | 15.77 | 66.46 | 1.13 | 21.56 | 21.59 | 1.52 | 60.11 |
| 2020-2021 | 1 | 6 | 623379 | 100 | 73.47 | 7.20 | 76 | 11.35 | 58.27 | 5.28 | 24.35 | 24.92 | 1.36 | 51.40 |
| 2020-2021 | 1 | 6 | 624910 | 100 | 84.18 | 7.27 | 96 | 15.29 | 65.24 | 5.22 | 24.45 | 25.00 | 1.36 | 57.18 |
| 2020-2021 | 1 | 6 | MOGHAN3 | 100 | 93.5 | 8.20 | 97 | 13.87 | 66.29 | 4.12 | 24.02 | 24.37 | 1.40 | 58.40 |
| 2020-2021 | 1 | 6 | DASTJERDI | 100 | 99.3 | 9.00 | 98 | 3.92 | 67.32 | 3.37 | 21.64 | 21.90 | 1.42 | 60.66 |
| 2020-2021 | 1 | 7 | TAKAB | 60 | 10.17 | 2.50 | 19 | 1.06 | 50.63 | 4.04 | 18.23 | 18.67 | 1.35 | 47.22 |
| 2020-2021 | 1 | 7 | 623908 | 100 | 96.36 | 8.50 | 92 | 12.94 | 60.98 | 3.35 | 19.52 | 19.81 | 1.40 | 56.24 |
| 2020-2021 | 1 | 7 | RASHID | 100 | 98.65 | 8.90 | 98 | 16.42 | 70.88 | 3.68 | 21.79 | 22.10 | 1.40 | 63.44 |
| 2020-2021 | 1 | 7 | 624900 | 100 | 84.83 | 7.67 | 94 | 13.28 | 67.67 | 5.58 | 24.42 | 25.05 | 1.35 | 59.10 |
| 2020-2021 | 1 | 7 | 627054 | 100 | 86.68 | 7.70 | 98 | 12.06 | 54.11 | 6.04 | 23.94 | 24.69 | 1.32 | 47.89 |
| 2020-2021 | 1 | 7 | 626223 | 100 | 97.27 | 8.73 | 100 | 13.36 | 63.15 | 3.64 | 22.97 | 23.26 | 1.41 | 56.42 |
| 2020-2021 | 1 | 7 | 627189 | 100 | 97.74 | 8.57 | 100 | 12.01 | 67.8 | 2.89 | 21.7 | 21.89 | 1.44 | 61.06 |
| 2020-2021 | 1 | 7 | 627423 | 100 | 97.12 | 8.67 | 90 | 13.21 | 65.39 | 5.96 | 24.42 | 25.14 | 1.33 | 57.22 |
| 2020-2021 | 1 | 7 | ZARE | 100 | 97.31 | 8.67 | 100 | 15.3 | 60.55 | 4.2 | 21.37 | 21.78 | 1.38 | 54.94 |
| 2020-2021 | 1 | 8 | 627410 | 100 | 98.06 | 8.83 | 100 | 9.11 | 54.12 | 4.57 | 18.73 | 19.28 | 1.33 | 50.23 |
| 2020-2021 | 1 | 8 | DN11 | 100 | 98.25 | 8.67 | 100 | 13.68 | 65.58 | 3.3 | 23.58 | 23.81 | 1.43 | 58.15 |
| 2020-2021 | 1 | 8 | 625080 | 100 | 93.14 | 8.23 | 98 | 13.51 | 62.05 | 4.67 | 21.85 | 22.34 | 1.36 | 55.96 |
| 2020-2021 | 1 | 8 | HOMA | 100 | 81.14 | 7.50 | 80 | 15.47 | 72.88 | 3.88 | 22.18 | 22.52 | 1.40 | 64.75 |
| 2020-2021 | 1 | 8 | 627842 | 100 | 96.13 | 8.67 | 100 | 12.56 | 62.79 | 3.99 | 20.84 | 21.22 | 1.38 | 57.17 |
| 2020-2021 | 1 | 8 | 627043 | 100 | 66.28 | 6.67 | 81 | 5.62 | 64.06 | 2.14 | 18.81 | 18.93 | 1.46 | 59.38 |
| 2020-2021 | 1 | 8 | 624956 | 100 | 92.94 | 8.27 | 93 | 13.98 | 68.27 | 5.51 | 22.83 | 23.49 | 1.33 | 60.52 |
| 2020-2021 | 1 | 8 | 626261 | 100 | 97.27 | 8.80 | 100 | 11.64 | 57.12 | 3.91 | 22.14 | 22.48 | 1.40 | 51.58 |
| 2020-2021 | 1 | 8 | 628084 | 100 | 95.42 | 8.37 | 100 | 8.19 | 51.06 | 3.04 | 16.81 | 17.08 | 1.39 | 48.16 |
| 2020-2021 | 1 | 8 | VEE/NAC | 100 | 95.5 | 8.57 | 100 | 11.18 | 55.02 | 4.85 | 23.27 | 23.77 | 1.37 | 49.13 |
| 2020-2021 | 1 | 9 | 625047 | 100 | 88.6 | 8.20 | 96 | 15.43 | 66.36 | 3.8 | 22.11 | 22.43 | 1.40 | 59.57 |
| 2020-2021 | 1 | 9 | 626158 | 100 | 83.64 | 7.73 | 92 | 7.33 | 53.75 | 1.8 | 17.03 | 17.12 | 1.47 | 50.68 |
| 2020-2021 | 1 | 9 | 624925 | 100 | 84.05 | 7.67 | 94 | 13.84 | 67.24 | 2.55 | 23.92 | 24.06 | 1.46 | 59.36 |
| 2020-2021 | 1 | 9 | 621619 | 100 | 82.18 | 7.47 | 91 | 10.36 | 62.85 | 2.2 | 20.36 | 20.48 | 1.46 | 57.58 |
| 2020-2021 | 1 | 9 | 627299 | 100 | 96.04 | 8.63 | 100 | 15.37 | 67.41 | 2.78 | 25.14 | 25.29 | 1.46 | 58.75 |
| 2020-2021 | 1 | 9 | 623291 | 100 | 89.87 | 8.00 | 100 | 7.14 | 48.1 | 3.64 | 18.41 | 18.77 | 1.38 | 44.81 |
| 2020-2021 | 1 | 9 | 627849 | 100 | 96.17 | 8.70 | 100 | 15.64 | 60.37 | 2.12 | 22.41 | 22.51 | 1.48 | 54.42 |
| 2020-2021 | 1 | 9 | 626978 | 100 | 97.76 | 8.67 | 100 | 14.93 | 60.01 | 3.23 | 22.79 | 23.02 | 1.43 | 53.86 |
| 2020-2021 | 1 | 9 | AKOVA | 100 | 95.04 | 8.53 | 96 | 12.47 | 55.3 | 5.3 | 21.41 | 22.06 | 1.33 | 50.15 |
| 2020-2021 | 1 | 9 | 627072 | 100 | 86.15 | 7.83 | 92 | 13.32 | 64.24 | 3.24 | 22.44 | 22.67 | 1.43 | 57.66 |
| 2020-2021 | 1 | 10 | 626908 | 100 | 94.87 | 8.43 | 100 | 12.02 | 55.12 | 5.17 | 22.56 | 23.14 | 1.35 | 49.50 |
| 2020-2021 | 1 | 10 | DARAB1 | 100 | 68.77 | 6.67 | 77 | 6.05 | 51.99 | 3.03 | 22.25 | 22.46 | 1.44 | 47.00 |
| 2020-2021 | 1 | 10 | 623421 | 100 | 79.86 | 7.33 | 92 | 11.02 | 56.71 | 3.61 | 19.95 | 20.27 | 1.39 | 52.20 |
| 2020-2021 | 1 | 10 | 623090 | 100 | 94.87 | 8.43 | 100 | 14.62 | 64.89 | 2 | 21.19 | 21.28 | 1.48 | 58.94 |
| 2020-2021 | 1 | 10 | 626234 | 100 | 92.31 | 8.33 | 98 | 9.34 | 50.57 | 4.45 | 17.02 | 17.59 | 1.32 | 47.53 |
| 2020-2021 | 1 | 10 | 627853 | 100 | 95.19 | 8.37 | 100 | 12.5 | 60.16 | 3.06 | 23.09 | 23.29 | 1.44 | 53.85 |
| 2020-2021 | 1 | 10 | GHABOUS | 100 | 90.69 | 8.20 | 98 | 8.91 | 53.26 | 1.99 | 20.45 | 20.55 | 1.47 | 48.94 |
| 2020-2021 | 1 | 10 | 624805 | 20 | 2.53 | 1.57 | 13 | 0.39 | 50.21 | 3.6 | 20.15 | 20.47 | 1.39 | 46.17 |
| 2020-2021 | 1 | 10 | 621565 | 100 | 68.54 | 6.63 | 77 | 12.37 | 57.25 | 2.97 | 24.3 | 24.48 | 1.45 | 50.74 |
| 2020-2021 | 1 | 10 | SABALAN | 100 | 96.15 | 8.53 | 100 | 14.07 | 67.15 | 2.31 | 23.51 | 23.62 | 1.47 | 59.54 |
| 2020-2021 | 1 | 11 | 626706 | 100 | 77.72 | 7.43 | 91 | 14.05 | 65.31 | 4.02 | 24.81 | 25.13 | 1.41 | 57.16 |
| 2020-2021 | 1 | 11 | 622063 | 100 | 92.2 | 8.27 | 98 | 8.14 | 56.86 | 1.98 | 19.19 | 19.29 | 1.47 | 52.74 |
| 2020-2021 | 1 | 11 | 626747 | 100 | 92.81 | 8.10 | 100 | 16 | 68.37 | 2.55 | 24.74 | 24.87 | 1.47 | 59.76 |
| 2020-2021 | 1 | 11 | 626573 | 100 | 91.9 | 8.10 | 100 | 15.66 | 69.34 | 4.06 | 24.85 | 25.18 | 1.41 | 60.33 |
| 2020-2021 | 1 | 11 | 622247 | 0 | 0 | 1.00 | 10 | 0.22 | 50.53 | 3.98 | 19.25 | 19.66 | 1.37 | 46.77 |
| 2020-2021 | 1 | 11 | 627990 | 100 | 90.12 | 8.23 | 98 | 13.85 | 66.66 | 5.29 | 22.62 | 23.23 | 1.34 | 59.36 |
| 2020-2021 | 1 | 11 | MAROON | 100 | 97.34 | 8.90 | 100 | 11.74 | 57.53 | 4.29 | 20.19 | 20.64 | 1.36 | 52.78 |
| 2020-2021 | 1 | 11 | 626226 | 100 | 97.07 | 8.63 | 100 | 15.87 | 67.37 | 4.1 | 22.79 | 23.16 | 1.39 | 59.99 |
| 2020-2021 | 1 | 11 | 626924 | 100 | 66.73 | 6.53 | 78 | 11.63 | 62.64 | 4.72 | 24.85 | 25.29 | 1.38 | 54.88 |
| 2020-2021 | 1 | 11 | 622084 | 0 | 0 | 1.00 | 10 | 0.31 | 48.73 | 1.84 | 17.53 | 17.63 | 1.47 | 45.78 |
| 2020-2021 | 1 | 12 | 624849 | 100 | 88.07 | 7.83 | 100 | 8.9 | 58.16 | 3.52 | 20.8 | 21.10 | 1.40 | 53.14 |
| 2020-2021 | 1 | 12 | 624576 | 100 | 92.5 | 8.40 | 98 | 8.46 | 53.5 | 2.84 | 22.06 | 22.24 | 1.44 | 48.45 |
| 2020-2021 | 1 | 12 | 626904 | 100 | 95.66 | 8.47 | 92 | 13.75 | 65.05 | 3.2 | 24.75 | 24.96 | 1.44 | 57.05 |
| 2020-2021 | 1 | 12 | 626895 | 100 | 84.47 | 7.63 | 90 | 17.96 | 65.58 | 4.6 | 24.83 | 25.25 | 1.39 | 57.31 |
| 2020-2021 | 1 | 12 | 627057 | 100 | 54.48 | 5.70 | 70 | 13.34 | 65.08 | 3.25 | 27.29 | 27.48 | 1.45 | 55.56 |
| 2020-2021 | 1 | 12 | SIRVAN | 100 | 99.62 | 9.00 | 100 | 3.15 | 60.44 | 2.47 | 24.65 | 24.77 | 1.47 | 53.32 |
| 2020-2021 | 1 | 12 | ALBORZ | 100 | 97.16 | 8.70 | 100 | 12.1 | 60.7 | 1.48 | 22.87 | 22.92 | 1.51 | 54.51 |
| 2020-2021 | 1 | 12 | 627987 | 100 | 96.79 | 8.60 | 100 | 10.37 | 61.22 | 1.44 | 20.19 | 20.24 | 1.50 | 56.26 |
| 2020-2021 | 1 | 12 | 624582 | 100 | 96.27 | 8.50 | 100 | 13.33 | 68.5 | 2.11 | 23.83 | 23.92 | 1.48 | 60.45 |
| 2020-2021 | 1 | 13 | 623909 | 100 | 100 | 9.00 | 100 | 12.68 | 56.59 | 5.05 | 20.56 | 21.17 | 1.33 | 51.70 |
| 2020-2021 | 1 | 13 | MARVDASHT | 100 | 100 | 9.00 | 100 | 12.84 | 60.37 | 4.98 | 20.9 | 21.49 | 1.34 | 54.92 |
| 2020-2021 | 1 | 13 | GAHAR | 100 | 99.32 | 8.90 | 100 | 0.1 | 52.3 | 3.67 | 17.84 | 18.21 | 1.37 | 48.94 |
| 2020-2021 | 1 | 13 | 627616 | 100 | 99.24 | 8.90 | 100 | 11.47 | 61.9 | 2.59 | 19.04 | 19.22 | 1.44 | 57.33 |
| 2020-2021 | 1 | 13 | 627102 | 80 | 20.1 | 3.40 | 27 | 2.87 | 51.12 | 5.05 | 19.04 | 19.70 | 1.31 | 47.30 |
| 2020-2021 | 1 | 13 | NAZ | 100 | 97.16 | 8.70 | 98 | 13.22 | 60.5 | 3.69 | 21.12 | 21.44 | 1.40 | 55.06 |
| 2020-2021 | 1 | 13 | 623109 | 100 | 96.01 | 8.47 | 98 | 15.68 | 71.1 | 5.45 | 23.97 | 24.58 | 1.35 | 62.06 |
| 2020-2021 | 1 | 13 | 623091 | 100 | 86.87 | 7.63 | 88 | 7.41 | 56.48 | 2.82 | 21.24 | 21.43 | 1.44 | 51.49 |
| 2020-2021 | 1 | 13 | BISTON | 100 | 73.23 | 7.03 | 80 | 6.01 | 56.33 | 1.77 | 20.19 | 20.27 | 1.48 | 51.86 |
| 2020-2021 | 1 | 14 | 625433 | 100 | 74.2 | 6.83 | 80 | 12.94 | 61.4 | 3.15 | 21.42 | 21.65 | 1.42 | 55.74 |
| 2020-2021 | 1 | 14 | 623161 | 100 | 88.84 | 7.97 | 97 | 9.68 | 54.17 | 2.23 | 20.65 | 20.77 | 1.46 | 49.68 |
| 2020-2021 | 1 | 14 | AZAR2 | 100 | 95.05 | 8.67 | 93 | 13.53 | 59.89 | 1.78 | 23.31 | 23.38 | 1.49 | 53.57 |
| 2020-2021 | 1 | 14 | 626699 | 100 | 83.91 | 7.33 | 91 | 12.43 | 60.14 | 2.29 | 21.17 | 21.29 | 1.46 | 54.81 |
| 2020-2021 | 1 | 14 | 627873 | 100 | 97.33 | 8.60 | 98 | 9.86 | 55.67 | 1.44 | 21.74 | 21.79 | 1.50 | 50.61 |
| 2020-2021 | 1 | 14 | 622379 | 100 | 93.36 | 8.20 | 100 | 15.06 | 66.02 | 1.82 | 23.11 | 23.18 | 1.49 | 58.87 |
| 2020-2021 | 1 | 14 | 627587 | 100 | 90.28 | 8.20 | 96 | 13.33 | 59.54 | 3.11 | 23.88 | 24.08 | 1.44 | 52.92 |
| 2020-2021 | 1 | 14 | 623127 | 100 | 95.71 | 8.50 | 98 | 11.68 | 59.05 | 3.93 | 21.17 | 21.53 | 1.39 | 53.73 |
| 2020-2021 | 1 | 14 | 623507 | 100 | 98.82 | 8.80 | 100 | 12.42 | 62.89 | 2.77 | 19.08 | 19.28 | 1.43 | 58.18 |
| 2020-2021 | 1 | 15 | SHIRAZ | 100 | 90.36 | 7.93 | 98 | 14.31 | 65.99 | 4.56 | 24.04 | 24.47 | 1.38 | 58.10 |
| 2020-2021 | 1 | 15 | MIHAN | 100 | 97.1 | 8.70 | 96 | 15.9 | 69.8 | 3.13 | 21.95 | 22.17 | 1.43 | 62.53 |
| 2020-2021 | 1 | 15 | 621650 | 100 | 96.13 | 8.43 | 92 | 14.87 | 62.55 | 4.28 | 21.86 | 22.28 | 1.38 | 56.43 |
| 2020-2021 | 1 | 15 | 627066 | 100 | 96.65 | 8.60 | 98 | 8.77 | 58.3 | 3.3 | 19.53 | 19.81 | 1.40 | 53.84 |
| 2020-2021 | 1 | 15 | FONG | 100 | 92.34 | 8.07 | 88 | 14.43 | 62.85 | 2.24 | 24.55 | 24.65 | 1.48 | 55.41 |
| 2020-2021 | 1 | 15 | 627787 | 100 | 99.06 | 8.90 | 96 | 9.83 | 61.91 | 1.62 | 18.95 | 19.02 | 1.49 | 57.43 |
| 2020-2021 | 1 | 15 | 623139 | 100 | 97.21 | 8.70 | 100 | 15.28 | 60.88 | 3.05 | 24.95 | 25.14 | 1.45 | 53.50 |
| 2020-2021 | 1 | 15 | 621908 | 100 | 96.38 | 8.60 | 96 | 14.37 | 66.72 | 3.68 | 23.97 | 24.25 | 1.42 | 58.82 |
| 2020-2021 | 1 | 16 | HAMOON | 100 | 94.42 | 8.40 | 98 | 12.44 | 65.85 | 1.6 | 21.73 | 21.79 | 1.50 | 59.49 |
| 2020-2021 | 1 | 16 | ALVAND | 100 | 91.14 | 8.20 | 88 | 14.28 | 66.85 | 3.12 | 23.49 | 23.70 | 1.44 | 59.25 |
| 2020-2021 | 1 | 16 | 624944 | 100 | 89.01 | 8.00 | 94 | 14.03 | 67.06 | 4.7 | 21.72 | 22.22 | 1.36 | 60.26 |
| 2020-2021 | 1 | 16 | 624846 | 100 | 92.56 | 8.30 | 98 | 12.73 | 63.41 | 3.74 | 20.33 | 20.67 | 1.39 | 57.97 |
| 2020-2021 | 1 | 16 | PISHTAZ | 100 | 99.09 | 9.00 | 94 | 12.49 | 57.12 | 3 | 20.8 | 21.02 | 1.43 | 52.25 |
| 2020-2021 | 1 | 16 | 623428 | 100 | 94.28 | 8.30 | 92 | 12.98 | 63.82 | 3.41 | 20.76 | 21.04 | 1.41 | 58.15 |
| 2020-2021 | 1 | 16 | 622894 | 100 | 95.09 | 8.50 | 98 | 13.68 | 69.18 | 4.64 | 22.98 | 23.44 | 1.37 | 61.28 |
| 2020-2021 | 1 | 16 | ARTA | 100 | 99.12 | 9.00 | 96 | 15.4 | 63.97 | 3.58 | 23.52 | 23.79 | 1.42 | 56.82 |
| 2020-2021 | 1 | 16 | 623162 | 100 | 86.41 | 7.73 | 91 | 12.29 | 72.91 | 5.18 | 21.07 | 21.70 | 1.33 | 65.29 |
| 2020-2021 | 1 | 17 | 628189 | 100 | 84.09 | 7.73 | 90 | 8.25 | 54.87 | 4.23 | 23.05 | 23.43 | 1.39 | 49.15 |
| 2020-2021 | 1 | 17 | INIA | 100 | 99.82 | 9.00 | 100 | 16.56 | 68.22 | 2.07 | 22.99 | 23.08 | 1.48 | 60.72 |
| 2020-2021 | 1 | 17 | REYHANI | 100 | 85.9 | 7.80 | 92 | 7.19 | 57.55 | 3.58 | 23.8 | 24.07 | 1.42 | 51.20 |
| 2020-2021 | 1 | 17 | 626215 | 100 | 55.09 | 5.87 | 69 | 7.78 | 60.4 | 2.58 | 23.76 | 23.90 | 1.46 | 53.75 |
| 2020-2021 | 1 | 17 | KARIM | 100 | 99 | 8.90 | 100 | 8.76 | 54.68 | 3.43 | 19.69 | 19.99 | 1.40 | 50.47 |
| 2020-2021 | 1 | 17 | 627414 | 100 | 82.98 | 7.60 | 93 | 12.21 | 66.22 | 3.53 | 21.59 | 21.88 | 1.41 | 59.75 |
| 2020-2021 | 1 | 17 | 627399 | 100 | 90.15 | 7.73 | 92 | 15.35 | 71.63 | 1.55 | 23.13 | 23.18 | 1.50 | 63.36 |
| 2020-2021 | 1 | 17 | 623318 | 100 | 64.25 | 6.33 | 80 | 5.65 | 55.29 | 2.34 | 18.24 | 18.39 | 1.44 | 51.66 |
| 2020-2021 | 1 | 17 | 627963 | 100 | 87.85 | 8.00 | 97 | 13.74 | 71.65 | 5.39 | 22.57 | 23.20 | 1.34 | 63.36 |
| 2020-2021 | 1 | 18 | KARAJ1 | 100 | 88.19 | 7.83 | 91 | 14.46 | 73.04 | 3.81 | 23.05 | 23.36 | 1.41 | 64.33 |
| 2020-2021 | 1 | 18 | DEZ | 100 | 91.53 | 7.97 | 96 | 11.37 | 67.15 | 3.51 | 21.85 | 22.13 | 1.41 | 60.39 |
| 2020-2021 | 1 | 18 | 621706 | 100 | 95.21 | 8.43 | 100 | 15.53 | 70.23 | 4.2 | 24.63 | 24.99 | 1.40 | 61.13 |
| 2020-2021 | 1 | 18 | 626358 | 100 | 89.74 | 8.23 | 93 | 15.5 | 63.16 | 4.63 | 22.79 | 23.26 | 1.37 | 56.43 |
| 2020-2021 | 1 | 18 | 623980 | 100 | 84.91 | 7.73 | 95 | 14.22 | 63.36 | 3.93 | 22.1 | 22.45 | 1.39 | 57.03 |
| 2020-2021 | 1 | 18 | 626776 | 100 | 82.79 | 7.63 | 87 | 10.06 | 59.74 | 1.87 | 17.45 | 17.55 | 1.46 | 56.08 |
| 2020-2021 | 1 | 18 | 625127 | 100 | 93.81 | 8.23 | 100 | 15.02 | 56.29 | 1.49 | 21.81 | 21.86 | 1.50 | 51.13 |
| 2020-2021 | 1 | 18 | CHAMRAN2 | 100 | 67.65 | 6.80 | 76 | 15.93 | 61.2 | 4.41 | 22.74 | 23.16 | 1.38 | 54.81 |
| 2020-2021 | 1 | 18 | NICKNEJAD | 80 | 28.55 | 3.97 | 44 | 2.83 | 52.78 | 4.56 | 20.59 | 21.09 | 1.35 | 48.28 |
| 2020-2021 | 1 | 18 | 623503 | 100 | 77.24 | 7.27 | 79 | 7.91 | 61.43 | 3.6 | 19.11 | 19.45 | 1.38 | 56.81 |
| 2020-2021 | 1 | 19 | FRONTANA | 0 | 0 | 1.00 | 6 | 16.36 | 72.31 | 2.4 | 24.78 | 24.90 | 1.47 | 62.76 |
| 2020-2021 | 1 | 19 | 627881 | 100 | 85.39 | 7.80 | 97 | 9.35 | 61.59 | 3.57 | 22.03 | 22.32 | 1.41 | 55.58 |
| 2020-2021 | 1 | 19 | 623377 | 100 | 97.88 | 8.70 | 98 | 14.81 | 74.82 | 1.85 | 23.49 | 23.56 | 1.49 | 65.51 |
| 2020-2021 | 1 | 19 | MV17 | 100 | 87.83 | 7.83 | 97 | 9.14 | 57.63 | 2.71 | 21.54 | 21.71 | 1.45 | 52.39 |
| 2020-2021 | 1 | 19 | 627948 | 100 | 89.41 | 8.10 | 95 | 11.81 | 60.62 | 3.45 | 22.46 | 22.72 | 1.42 | 54.53 |
| 2020-2021 | 1 | 19 | BAHAR | 100 | 86.94 | 7.90 | 90 | 12.6 | 58.09 | 3.16 | 24.23 | 24.44 | 1.44 | 51.49 |
| 2020-2021 | 1 | 19 | 624818 | 100 | 74.2 | 6.90 | 80 | 6.31 | 49.92 | 2.46 | 17.95 | 18.12 | 1.43 | 46.74 |
| 2020-2021 | 1 | 19 | 623345 | 100 | 85.52 | 7.67 | 87 | 13.88 | 68.51 | 4.07 | 23.38 | 23.73 | 1.40 | 60.57 |
| 2020-2021 | 1 | 20 | 627908 | 100 | 92.71 | 8.40 | 97 | 10.11 | 59.27 | 4.68 | 20.83 | 21.35 | 1.35 | 54.01 |
| 2020-2021 | 1 | 20 | 623169 | 100 | 22.43 | 4.07 | 34 | 2.9 | 56.08 | 3.85 | 22.42 | 22.75 | 1.40 | 50.54 |
| 2020-2021 | 1 | 20 | ATRAK | 100 | 56.35 | 6.07 | 67 | 6.1 | 53.66 | 3.87 | 22.55 | 22.88 | 1.40 | 48.32 |
| 2020-2021 | 1 | 20 | 627356 | 100 | 88.18 | 7.90 | 96 | 12.98 | 62.59 | 2.77 | 21.88 | 22.05 | 1.44 | 56.57 |
| 2020-2021 | 1 | 20 | 626943 | 100 | 56.28 | 5.97 | 65 | 4.95 | 52.36 | 2.49 | 18.99 | 19.15 | 1.44 | 48.65 |
| 2020-2021 | 1 | 20 | 627417 | 100 | 71.01 | 6.97 | 78 | 9.34 | 59.46 | 2.86 | 19.45 | 19.66 | 1.42 | 54.94 |
| 2020-2021 | 1 | 20 | 628088 | 100 | 93.41 | 8.37 | 95 | 11.69 | 65.08 | 2.48 | 21.4 | 21.54 | 1.46 | 58.97 |
| 2020-2021 | 1 | 20 | BAYAT | 100 | 82.34 | 7.43 | 79 | 12.49 | 61.97 | 2.44 | 23.65 | 23.78 | 1.47 | 55.15 |
| 2020-2021 | 1 | 20 | FALAT | 100 | 98.37 | 8.80 | 94 | 12.19 | 55.73 | 4.4 | 23.37 | 23.78 | 1.38 | 49.75 |
| 2020-2021 | 1 | 20 | 626933 | 100 | 89.92 | 8.10 | 94 | 12.92 | 59.88 | 2.75 | 22.75 | 22.92 | 1.45 | 53.80 |
| 2020-2021 | 1 | 21 | 623125 | 100 | 81.61 | 7.60 | 90 | 10.84 | 61.27 | 4.55 | 22.12 | 22.58 | 1.37 | 55.17 |
| 2020-2021 | 1 | 21 | DARAB2 | 100 | 90.17 | 8.03 | 91 | 7.57 | 44.42 | 3.82 | 16.47 | 16.91 | 1.34 | 41.91 |
| 2020-2021 | 1 | 21 | 624980 | 100 | 64.74 | 6.47 | 71 | 8.01 | 60.48 | 4.92 | 22.39 | 22.92 | 1.35 | 54.31 |
| 2020-2021 | 1 | 21 | 625281 | 80 | 21.79 | 3.77 | 35 | 3.04 | 56.14 | 2.1 | 18.7 | 18.82 | 1.46 | 52.27 |
| 2020-2021 | 1 | 21 | TAJAN | 100 | 91.55 | 8.07 | 94 | 11.06 | 59.07 | 4.21 | 19.56 | 20.01 | 1.36 | 54.44 |
| 2020-2021 | 1 | 21 | 623382 | 100 | 62.22 | 6.43 | 70 | 12.55 | 66.05 | 3.51 | 25.24 | 25.48 | 1.43 | 57.55 |
| 2020-2021 | 1 | 21 | 624580 | 80 | 11.59 | 2.63 | 26 | 1.37 | 50.09 | 3.42 | 18.91 | 19.22 | 1.39 | 46.52 |
| 2020-2021 | 1 | 21 | KAVIR | 100 | 66.36 | 6.33 | 75 | 8.25 | 60.61 | 3.06 | 23.23 | 23.43 | 1.44 | 54.17 |
| 2020-2021 | 1 | 21 | 626883 | 100 | 82.89 | 7.40 | 82 | 17.74 | 69.03 | 0.98 | 23.12 | 23.14 | 1.53 | 61.34 |
| 2020-2021 | 1 | 21 | BAM | 100 | 84.83 | 7.70 | 91 | 11.12 | 67.7 | 2.39 | 25.36 | 25.47 | 1.48 | 58.86 |
| 2020-2021 | 1 | 22 | 623136 | 100 | 60.53 | 5.83 | 68 | 8.69 | 60.98 | 3.55 | 20.59 | 20.89 | 1.40 | 55.74 |
| 2020-2021 | 1 | 22 | KAVEH | 100 | 93.75 | 8.40 | 100 | 12.75 | 57.78 | 2.64 | 24 | 24.14 | 1.46 | 51.36 |
| 2020-2021 | 1 | 22 | 626923 | 100 | 36.07 | 5.47 | 51 | 5.82 | 66.54 | 2.33 | 22.31 | 22.43 | 1.47 | 59.72 |
| 2020-2021 | 1 | 22 | MOGHAN1 | 100 | 71.75 | 7.00 | 85 | 8.06 | 57.38 | 2.69 | 19.3 | 19.49 | 1.43 | 53.14 |
| 2020-2021 | 1 | 22 | 627099 | 100 | 90.8 | 8.30 | 96 | 8.69 | 54.42 | 1.4 | 17.17 | 17.23 | 1.49 | 51.27 |
| 2020-2021 | 1 | 22 | 626260 | 100 | 34.33 | 5.10 | 43 | 3.17 | 50.47 | 3.68 | 18.17 | 18.54 | 1.37 | 47.11 |
| 2020-2021 | 1 | 22 | DARYA | 100 | 35.28 | 5.00 | 40 | 11.37 | 52.41 | 4.18 | 20.89 | 21.30 | 1.37 | 47.86 |
| 2020-2021 | 1 | 22 | 623176 | 100 | 52.37 | 6.07 | 63 | 4.19 | 52.44 | 2.98 | 21.4 | 21.61 | 1.43 | 47.76 |
| 2020-2021 | 1 | 22 | 621492 | 100 | 24.55 | 4.20 | 35 | 3.1 | 61.76 | 4.12 | 23.27 | 23.63 | 1.40 | 55.05 |
| 2020-2021 | 1 | 22 | URUOM | 100 | 78.29 | 7.30 | 77 | 10.39 | 65.63 | 3.12 | 21.57 | 21.79 | 1.43 | 59.30 |
| 2020-2021 | 1 | 23 | 627723 | 100 | 82.87 | 7.60 | 88 | 9.37 | 60.48 | 4.38 | 20.02 | 20.49 | 1.36 | 55.48 |
| 2020-2021 | 1 | 23 | BEZOSTAYA | 80 | 14.37 | 3.33 | 29 | 1.4 | 55.25 | 5.51 | 20.23 | 20.97 | 1.30 | 50.58 |
| 2020-2021 | 1 | 23 | 626565 | 100 | 80.09 | 7.30 | 87 | 7.17 | 52.79 | 2.58 | 16.75 | 16.95 | 1.42 | 49.84 |
| 2020-2021 | 1 | 23 | 4820 | 100 | 64.11 | 6.63 | 72 | 11.59 | 66.66 | 2.48 | 22.62 | 22.76 | 1.46 | 59.63 |
| 2020-2021 | 1 | 23 | OFOG | 100 | 74.8 | 7.30 | 68 | 9.41 | 62.11 | 2.75 | 22.88 | 23.04 | 1.45 | 55.65 |
| 2020-2021 | 1 | 23 | 624946 | 90 | 24.29 | 3.83 | 35 | 2.49 | 60.61 | 2 | 21.58 | 21.67 | 1.48 | 55.04 |
| 2020-2021 | 1 | 23 | 624894 | 100 | 61.11 | 6.53 | 68 | 4.94 | 53.04 | 1.73 | 16.13 | 16.22 | 1.46 | 50.32 |
| 2020-2021 | 1 | 23 | AZAR1 | 100 | 52.12 | 5.93 | 59 | 4.27 | 47.81 | 2.99 | 18.17 | 18.41 | 1.41 | 44.66 |
| 2020-2021 | 1 | 23 | 626958 | 100 | 49 | 5.77 | 61 | 7.68 | 66.95 | 1.43 | 22.69 | 22.74 | 1.51 | 59.89 |
| 2020-2021 | 1 | 23 | 623417 | 100 | 79.49 | 7.57 | 81 | 6.88 | 63.54 | 2.32 | 18.35 | 18.50 | 1.45 | 59.12 |
| 2020-2021 | 1 | 24 | 624378 | 90 | 29.82 | 4.47 | 41 | 3.53 | 57.69 | 3.64 | 19.3 | 19.64 | 1.38 | 53.35 |
| 2020-2021 | 1 | 24 | SISTAN | 100 | 86.87 | 7.67 | 97 | 6.08 | 52.54 | 3.36 | 18.78 | 19.08 | 1.39 | 48.85 |
| 2020-2021 | 1 | 24 | 626872 | 100 | 96.4 | 8.50 | 96 | 6.73 | 49.77 | 3 | 15.72 | 16.00 | 1.38 | 47.28 |
| 2020-2021 | 1 | 24 | 624381 | 100 | 59.29 | 6.10 | 76 | 9.38 | 68.97 | 3.35 | 22.57 | 22.82 | 1.42 | 61.48 |
| 2020-2021 | 1 | 24 | 625810 | 80 | 24.94 | 3.83 | 41 | 4.45 | 64.1 | 3.69 | 22.4 | 22.70 | 1.41 | 57.52 |
| 2020-2021 | 1 | 24 | 622311 | 100 | 65.15 | 6.13 | 76 | 8.65 | 61.04 | 3.32 | 18.03 | 18.33 | 1.39 | 56.94 |
| 2020-2021 | 1 | 24 | 627385 | 90 | 95.26 | 8.43 | 92 | 11.99 | 66 | 3.69 | 22.51 | 22.81 | 1.41 | 59.06 |
| 2020-2021 | 1 | 24 | 623905 | 100 | 49.78 | 5.67 | 63 | 4.67 | 51.4 | 3.61 | 18.65 | 19.00 | 1.38 | 47.82 |
| 2020-2021 | 1 | 24 | 627460 | 100 | 94.19 | 8.40 | 96 | 13.07 | 65.56 | 4.13 | 20.81 | 21.22 | 1.37 | 59.55 |
| 2020-2021 | 1 | 25 | 627760 | 90 | 16.2 | 3.27 | 29 | 6.76 | 71.11 | 2.29 | 23.19 | 23.30 | 1.47 | 62.88 |
| 2020-2021 | 1 | 25 | 625362 | 100 | 72.2 | 6.47 | 67 | 9.53 | 57.32 | 2.98 | 23.97 | 24.15 | 1.45 | 50.96 |
| 2020-2021 | 1 | 25 | 623274 | 100 | 87.54 | 8.00 | 87 | 13.63 | 63.48 | 3.14 | 21.56 | 21.79 | 1.43 | 57.47 |
| 2020-2021 | 1 | 25 | 626736 | 100 | 65.71 | 6.60 | 71 | 7.88 | 53.54 | 2.49 | 21.99 | 22.13 | 1.46 | 48.54 |
| 2020-2021 | 1 | 25 | 624585 | 80 | 15.23 | 3.30 | 18 | 1.48 | 47.2 | 4.14 | 18.38 | 18.84 | 1.35 | 43.94 |
| 2020-2021 | 1 | 25 | KOOHDASHT | 90 | 95.91 | 8.60 | 96 | 14.91 | 62.58 | 1.88 | 25.36 | 25.43 | 1.50 | 54.76 |
| 2020-2021 | 1 | 25 | NAVID | 100 | 93.68 | 8.40 | 100 | 10.02 | 67.25 | 1.49 | 23.06 | 23.11 | 1.51 | 59.92 |
| 2020-2021 | 1 | 25 | KARAJ2 | 100 | 100 | 9.00 | 100 | 15.23 | 67.77 | 1.21 | 22.11 | 22.14 | 1.52 | 60.90 |
| 2020-2021 | 1 | 25 | CHAMRAN | 100 | 97.33 | 8.73 | 92 | 0.14 | 51.35 | 1.47 | 15.56 | 15.63 | 1.48 | 48.90 |
| 2020-2021 | 1 | 25 | 624864 | 100 | 55.98 | 5.87 | 57 | 5.49 | 61.81 | 3.71 | 20.41 | 20.74 | 1.39 | 56.54 |
| 2020-2021 | 1 | 26 | 625123 | 100 | 96.47 | 8.67 | 97 | 12.15 | 53.58 | 2.73 | 21.14 | 21.32 | 1.44 | 48.92 |
| 2020-2021 | 1 | 26 | CASGOGEN | 10 | 2.05 | 1.33 | 13 | 8.89 | 52.18 | 2.75 | 16.54 | 16.77 | 1.41 | 49.33 |
| 2020-2021 | 1 | 26 | 626764 | 100 | 42.02 | 5.40 | 63 | 4.8 | 54.37 | 3.06 | 17.65 | 17.91 | 1.40 | 50.98 |
| 2020-2021 | 1 | 26 | 624804 | 100 | 45.84 | 5.57 | 55 | 4.72 | 58.02 | 3.65 | 19.02 | 19.37 | 1.38 | 53.77 |
| 2020-2021 | 1 | 26 | 626846 | 100 | 97.54 | 8.80 | 96 | 7.25 | 49.53 | 3.63 | 16.24 | 16.64 | 1.35 | 46.86 |
| 2020-2021 | 1 | 26 | ZARRIN | 100 | 49.1 | 5.70 | 62 | 3.82 | 56.34 | 4.63 | 20.71 | 21.22 | 1.35 | 51.46 |
| 2020-2021 | 1 | 26 | 626814 | 100 | 77.54 | 7.50 | 79 | 9.33 | 57.1 | 2.45 | 21.55 | 21.69 | 1.46 | 51.93 |
| 2020-2021 | 1 | 26 | 626825 | 100 | 80.63 | 7.43 | 77 | 10.34 | 61.69 | 2.62 | 22.23 | 22.38 | 1.45 | 55.63 |
| 2020-2021 | 1 | 26 | DEYHEM | 100 | 76.32 | 7.33 | 76 | 9.29 | 54.65 | 3.55 | 17.31 | 17.67 | 1.37 | 51.33 |
| 2020-2021 | 1 | 26 | 624251 | 100 | 60.63 | 6.10 | 59 | 5.78 | 57.39 | 2.85 | 20.68 | 20.88 | 1.43 | 52.55 |
| 2020-2021 | 1 | 27 | 623953 | 10 | 1.29 | 1.33 | 13 | 0.19 | 66.72 | 2.07 | 25.36 | 25.44 | 1.49 | 58.11 |
| 2020-2021 | 1 | 27 | 626855 | 100 | 96.67 | 8.80 | 96 | 15.91 | 64.86 | 2.53 | 24.77 | 24.90 | 1.47 | 56.93 |
| 2020-2021 | 1 | 27 | 623008 | 100 | 97.79 | 8.80 | 98 | 9.87 | 55.34 | 2.79 | 20.48 | 20.67 | 1.44 | 50.79 |
| 2020-2021 | 1 | 27 | 621669 | 100 | 97.78 | 8.80 | 100 | 9.35 | 60.55 | 4.64 | 19.5 | 20.04 | 1.34 | 55.75 |
| 2020-2021 | 1 | 27 | SHARYAR | 100 | 97.75 | 8.80 | 100 | 10.2 | 67.36 | 4.77 | 22.02 | 22.53 | 1.36 | 60.34 |
| 2020-2021 | 1 | 27 | ADL | 100 | 97.13 | 8.70 | 100 | 11.36 | 60.58 | 2.06 | 19.8 | 19.91 | 1.47 | 55.84 |
| 2020-2021 | 1 | 27 | PANJAMO62 | 100 | 87.5 | 8.00 | 92 | 11.61 | 72.31 | 3.49 | 23.18 | 23.44 | 1.42 | 63.72 |
| 2020-2021 | 1 | 27 | 624939 | 100 | 92.37 | 8.37 | 95 | 9.71 | 62.75 | 3.94 | 19.05 | 19.45 | 1.37 | 57.98 |
| 2020-2021 | 1 | 27 | PISHGAM | 100 | 85.64 | 7.73 | 92 | 13.6 | 67.11 | 4.25 | 23.9 | 24.27 | 1.39 | 59.12 |
| 2020-2021 | 1 | 27 | 626360 | 100 | 89.11 | 8.10 | 88 | 9.73 | 56.37 | 4.33 | 20.66 | 21.11 | 1.36 | 51.53 |
| 2020-2021 | 1 | 28 | 624983 | 100 | 67.89 | 6.53 | 76 | 8.99 | 62.49 | 5.12 | 22.27 | 22.85 | 1.34 | 56.08 |
| 2020-2021 | 1 | 28 | SIVAND | 100 | 81.37 | 7.27 | 81 | 14.23 | 60.61 | 4.49 | 24.17 | 24.58 | 1.39 | 53.57 |
| 2020-2021 | 1 | 28 | 627551 | 70 | 4.83 | 2.27 | 11 | 0.5 | 43.84 | 4.59 | 18.16 | 18.73 | 1.32 | 40.80 |
| 2020-2021 | 1 | 28 | SHAHI | 100 | 81.34 | 7.30 | 82 | 9.97 | 59.69 | 4.46 | 22.56 | 23.00 | 1.38 | 53.59 |
| 2020-2021 | 1 | 28 | 621717 | 100 | 25.69 | 4.87 | 35 | 3.47 | 60.38 | 2.27 | 24.27 | 24.38 | 1.48 | 53.48 |
| 2020-2021 | 1 | 28 | ARVAND | 100 | 95.12 | 8.60 | 88 | 11.78 | 56.64 | 2.15 | 18.7 | 18.82 | 1.46 | 52.73 |
| 2020-2021 | 1 | 28 | MORVARID | 100 | 97.37 | 8.70 | 100 | 9.99 | 56.03 | 2.14 | 23.31 | 23.41 | 1.48 | 50.19 |
| 2020-2021 | 1 | 28 | 624863 | 90 | 13.59 | 3.30 | 32 | 2.3 | 63.12 | 2.28 | 22.18 | 22.30 | 1.47 | 56.90 |
| 2020-2021 | 1 | 28 | 626881 | 100 | 63.38 | 6.43 | 77 | 12.55 | 60.52 | 3.59 | 23.07 | 23.35 | 1.42 | 54.13 |
| 2020-2021 | 1 | 28 | TOUBARI | 100 | 67.75 | 6.70 | 80 | 9.15 | 59.35 | 1.87 | 20.08 | 20.17 | 1.48 | 54.62 |
| 2020-2021 | 1 | 29 | SHINGHAI | 80 | 57.86 | 5.90 | 58 | 11.01 | 59.94 | 4.98 | 20.11 | 20.72 | 1.33 | 54.90 |
| 2020-2021 | 1 | 29 | KARAJ3 | 100 | 62.09 | 6.10 | 60 | 8.93 | 60.73 | 4.77 | 20.77 | 21.31 | 1.35 | 55.32 |
| 2020-2021 | 1 | 29 | 624947 | 100 | 76.46 | 7.17 | 73 | 8.31 | 53.68 | 4.52 | 19.67 | 20.18 | 1.34 | 49.47 |
| 2020-2021 | 1 | 29 | GOLESTAN | 100 | 89.3 | 8.03 | 91 | 10.89 | 64.41 | 3.14 | 18.77 | 19.03 | 1.41 | 59.64 |
| 2020-2021 | 1 | 29 | 624963 | 100 | 40.85 | 5.27 | 49 | 4.61 | 59.34 | 3.79 | 20.5 | 20.85 | 1.39 | 54.31 |
| 2020-2021 | 1 | 29 | AZADI | 100 | 97.48 | 8.70 | 90 | 12.07 | 54.42 | 3.07 | 20.12 | 20.35 | 1.42 | 50.08 |
| 2020-2021 | 1 | 29 | 623123 | 100 | 86.54 | 8.00 | 82 | 11.75 | 58.89 | 4.33 | 23.9 | 24.29 | 1.39 | 52.25 |
| 2020-2021 | 1 | 29 | AFLAK | 100 | 99.48 | 8.90 | 90 | 15.01 | 70.5 | 1.85 | 24.65 | 24.72 | 1.50 | 61.51 |
| 2020-2021 | 1 | 29 | KHAZAR1 | 100 | 83.15 | 7.43 | 81 | 9.82 | 61.44 | 1.48 | 21.9 | 21.95 | 1.50 | 55.63 |
| 2020-2021 | 1 | 30 | 623069 | 100 | 66.22 | 6.53 | 68 | 8.03 | 68.99 | 2.69 | 23.69 | 23.84 | 1.46 | 60.88 |
| 2020-2021 | 1 | 30 | ZAGROS | 100 | 93.34 | 8.40 | 87 | 8.91 | 56.87 | 1.88 | 21.46 | 21.54 | 1.48 | 51.79 |
| 2020-2021 | 1 | 30 | 624901 | 100 | 54.94 | 6.07 | 57 | 5.17 | 56.46 | 2.28 | 18.66 | 18.80 | 1.45 | 52.58 |
| 2020-2021 | 1 | 30 | SISON | 70 | 10.96 | 2.53 | 15 | 12.6 | 65.54 | 2.75 | 21.65 | 21.82 | 1.44 | 59.21 |
| 2020-2021 | 1 | 30 | 624215 | 40 | 5.69 | 2.00 | 6 | 0.4 | 53.14 | 2.01 | 17.39 | 17.51 | 1.46 | 49.98 |
| 2020-2021 | 1 | 30 | GHODS | 100 | 99.53 | 8.90 | 90 | 12.7 | 57.77 | 3.49 | 20.3 | 20.60 | 1.40 | 53.01 |
| 2020-2021 | 1 | 30 | 624861 | 100 | 65.85 | 6.50 | 68 | 9.17 | 66.66 | 2.9 | 22.48 | 22.67 | 1.44 | 59.68 |
| 2020-2021 | 1 | 30 | RIJAW | 100 | 99.34 | 9.00 | 90 | 14.25 | 66.5 | 3.99 | 23.16 | 23.50 | 1.40 | 59.08 |
| 2020-2021 | 1 | 30 | SPAHAN | 100 | 99.42 | 8.90 | 90 | 9.99 | 58.29 | 2.65 | 21.06 | 21.23 | 1.45 | 53.20 |
| 2020-2021 | 1 | 31 | 627055 | 100 | 65.74 | 6.77 | 63 | 6.63 | 61.13 | 3.88 | 18.56 | 18.96 | 1.36 | 56.75 |
| 2020-2021 | 1 | 31 | 626932 | 100 | 64.49 | 6.60 | 63 | 8.97 | 70.73 | 4 | 23 | 23.35 | 1.40 | 62.56 |
| 2020-2021 | 1 | 31 | PARSI | 100 | 98.77 | 8.90 | 90 | 13.5 | 65.45 | 4.23 | 22.28 | 22.68 | 1.38 | 58.67 |
| 2020-2021 | 1 | 31 | 624838 | 100 | 52.73 | 5.97 | 53 | 6.57 | 63.59 | 4.63 | 23.7 | 24.15 | 1.38 | 56.31 |
| 2020-2021 | 1 | 31 | 621736 | 100 | 39.02 | 5.07 | 41 | 4.98 | 63.69 | 2.39 | 23.3 | 23.42 | 1.47 | 56.79 |
| 2020-2021 | 1 | 31 | 621869 | 90 | 39.49 | 4.80 | 44 | 5.63 | 66.63 | 2.13 | 22.4 | 22.50 | 1.48 | 59.75 |
| 2020-2021 | 1 | 31 | 628012 | 90 | 19.15 | 3.70 | 23 | 1.62 | 57.11 | 2.8 | 18.34 | 18.55 | 1.42 | 53.27 |
| 2020-2021 | 1 | 31 | 622099 | 0 | 0 | 1.00 | 0 | 0.13 | 52.3 | 1.48 | 17.18 | 17.24 | 1.48 | 49.28 |
| 2020-2021 | 1 | 31 | 622105 | 0 | 0 | 1.00 | 0 | 0.09 | 47.85 | 5.78 | 19.42 | 20.26 | 1.28 | 44.05 |
| 2020-2021 | 1 | 32 | 621420 | 100 | 34.68 | 5.07 | 39 | 5.23 | 56.3 | 2.74 | 20.43 | 20.61 | 1.44 | 51.68 |
| 2020-2021 | 1 | 32 | 623266 | 60 | 15 | 3.03 | 22 | 1.66 | 54.35 | 2.25 | 18.69 | 18.82 | 1.45 | 50.62 |
| 2020-2021 | 1 | 32 | 621712 | 100 | 86.37 | 7.70 | 77 | 8.4 | 49.65 | 4.84 | 18.59 | 19.21 | 1.32 | 46.11 |
| 2020-2021 | 1 | 32 | 620903 | 100 | 69.96 | 6.97 | 63 | 4.46 | 48.18 | 1.62 | 16.56 | 16.64 | 1.47 | 45.57 |
| 2020-2021 | 1 | 32 | 625139 | 100 | 50.01 | 5.80 | 55 | 3.12 | 53.13 | 2.36 | 15.32 | 15.50 | 1.42 | 50.63 |
| 2020-2021 | 1 | 32 | 622098 | 100 | 82.81 | 8.07 | 80 | 10.77 | 69.2 | 4.29 | 21.12 | 21.55 | 1.37 | 62.41 |
| 2020-2021 | 1 | 32 | 625263 | 30 | 4.88 | 1.83 | 7 | 0.34 | 56.8 | 1.48 | 19.02 | 19.08 | 1.49 | 52.78 |
| 2020-2021 | 1 | 32 | 622264 | 0 | 0 | 1.00 | 0 | 0.12 | 50.04 | 4.82 | 19.05 | 19.65 | 1.32 | 46.31 |
| 2020-2021 | 1 | 32 | 622272 | 0 | 0 | 1.00 | 0 | 0.12 | 47.04 | 2.47 | 18.62 | 18.78 | 1.44 | 43.81 |
| 2020-2021 | 1 | 32 | 627905 | 50 | 14.51 | 2.90 | 16 | 1 | 55.51 | 1.15 | 15.14 | 15.18 | 1.49 | 52.99 |
| 2020-2021 | 2 | 1 | 627036 | 100 | 98.17 | 8.80 | 100 | 12.63 | 62.1 | 3.52 | 23.75 | 24.01 | 1.42 | 55.14 |
| 2020-2021 | 2 | 1 | 626156 | 90 | 75.71 | 7.13 | 83 | 7.83 | 52.68 | 3.6 | 21.48 | 21.78 | 1.40 | 47.91 |
| 2020-2021 | 2 | 1 | ROSHAN | 100 | 95.24 | 8.50 | 100 | 14.04 | 54.04 | 4.37 | 21.54 | 21.98 | 1.37 | 49.06 |
| 2020-2021 | 2 | 1 | 627852 | 100 | 95.1 | 8.33 | 100 | 13.91 | 61.47 | 3.67 | 23.79 | 24.07 | 1.42 | 54.57 |
| 2020-2021 | 2 | 1 | 628114 | 100 | 97.79 | 8.67 | 100 | 11.17 | 63.65 | 3.38 | 22.07 | 22.33 | 1.42 | 57.34 |
| 2020-2021 | 2 | 1 | 624985 | 100 | 94.72 | 8.33 | 100 | 9.12 | 56.81 | 5.45 | 21.52 | 22.20 | 1.32 | 51.44 |
| 2020-2021 | 2 | 1 | 624837 | 100 | 82.76 | 7.40 | 90 | 11.72 | 70.67 | 3.41 | 23.83 | 24.07 | 1.43 | 62.06 |
| 2020-2021 | 2 | 1 | TOUS | 100 | 81.37 | 7.57 | 88 | 7.74 | 52.59 | 4.19 | 21.02 | 21.43 | 1.37 | 47.97 |
| 2020-2021 | 2 | 1 | 623473 | 100 | 85.48 | 7.33 | 98 | 9.59 | 54.78 | 4.05 | 21.05 | 21.44 | 1.38 | 49.96 |
| 2020-2021 | 2 | 2 | MOGHAN2 | 100 | 94.31 | 8.53 | 100 | 14.13 | 64.64 | 4.04 | 22.34 | 22.70 | 1.39 | 57.98 |
| 2020-2021 | 2 | 2 | 627359 | 100 | 95.95 | 8.50 | 100 | 10.02 | 56.37 | 3.93 | 21.42 | 21.78 | 1.39 | 51.24 |
| 2020-2021 | 2 | 2 | 627038 | 100 | 97.97 | 8.83 | 100 | 12.4 | 57.26 | 5.14 | 20.82 | 21.45 | 1.33 | 52.18 |
| 2020-2021 | 2 | 2 | 621704 | 100 | 68.41 | 6.83 | 71 | 5.6 | 49.95 | 1.45 | 16.72 | 16.78 | 1.48 | 47.21 |
| 2020-2021 | 2 | 2 | 626566 | 100 | 71.26 | 6.90 | 75 | 7.02 | 51.63 | 4.25 | 20.32 | 20.76 | 1.36 | 47.36 |
| 2020-2021 | 2 | 2 | SHAHPASSAND | 100 | 89.62 | 8.07 | 97 | 15.64 | 66.22 | 3.86 | 23.13 | 23.45 | 1.41 | 58.88 |
| 2020-2021 | 2 | 2 | 623508 | 100 | 78.72 | 7.17 | 88 | 13.44 | 62.38 | 4.36 | 24.54 | 24.92 | 1.39 | 54.87 |
| 2020-2021 | 2 | 2 | 621735 | 100 | 90.7 | 8.00 | 97 | 10.05 | 50.89 | 1.89 | 19.97 | 20.06 | 1.48 | 46.95 |
| 2020-2021 | 2 | 2 | 623338 | 100 | 96.27 | 8.67 | 100 | 15.88 | 59.46 | 3.05 | 23.96 | 24.15 | 1.44 | 52.81 |
| 2020-2021 | 2 | 2 | 621668 | 100 | 94.94 | 8.50 | 100 | 8.73 | 52.15 | 3.17 | 17.01 | 17.30 | 1.39 | 49.12 |
| 2020-2021 | 2 | 3 | 621421 | 100 | 95.19 | 8.50 | 100 | 7.08 | 57.01 | 1.56 | 16.18 | 16.26 | 1.47 | 54.04 |
| 2020-2021 | 2 | 3 | 624240 | 100 | 97.56 | 8.67 | 100 | 10.51 | 57.38 | 3.95 | 21.01 | 21.38 | 1.38 | 52.32 |
| 2020-2021 | 2 | 3 | 623344 | 100 | 95.16 | 8.33 | 100 | 16.05 | 63.22 | 3.72 | 23.78 | 24.07 | 1.42 | 56.04 |
| 2020-2021 | 2 | 3 | 624941 | 0 | 0 | 1.00 | 0 | 0.13 | 51.17 | 4.04 | 17.69 | 18.15 | 1.35 | 47.91 |
| 2020-2021 | 2 | 3 | 621716 | 100 | 88.71 | 7.83 | 97 | 12.63 | 63.95 | 6.89 | 24.85 | 25.79 | 1.30 | 55.68 |
| 2020-2021 | 2 | 3 | 627236 | 100 | 93.83 | 8.33 | 100 | 14.93 | 67.91 | 4.33 | 22.91 | 23.32 | 1.38 | 60.33 |
| 2020-2021 | 2 | 3 | 624911 | 100 | 88.71 | 8.00 | 93 | 12.53 | 67.49 | 2.45 | 21.81 | 21.95 | 1.46 | 60.78 |
| 2020-2021 | 2 | 4 | OHADI | 100 | 86.01 | 7.73 | 93 | 14.6 | 70.31 | 4.7 | 22.85 | 23.33 | 1.37 | 62.24 |
| 2020-2021 | 2 | 4 | 624990 | 100 | 71.71 | 6.77 | 81 | 10.54 | 65.49 | 3.98 | 22.76 | 23.11 | 1.40 | 58.47 |
| 2020-2021 | 2 | 4 | 627484 | 100 | 72.73 | 7.33 | 77 | 10.74 | 59.63 | 6.24 | 21.67 | 22.55 | 1.29 | 53.76 |
| 2020-2021 | 2 | 4 | 627688 | 100 | 94.12 | 8.33 | 97 | 6.59 | 46.61 | 2.52 | 15.96 | 16.16 | 1.41 | 44.22 |
| 2020-2021 | 2 | 4 | 627416 | 100 | 96.65 | 8.67 | 100 | 15.11 | 70.09 | 3.68 | 21.64 | 21.95 | 1.40 | 62.90 |
| 2020-2021 | 2 | 4 | 625661 | 100 | 96.32 | 8.50 | 100 | 9.22 | 48.73 | 1.56 | 17.45 | 17.52 | 1.48 | 45.82 |
| 2020-2021 | 2 | 4 | 625081 | 100 | 77.05 | 7.50 | 83 | 12.52 | 57.53 | 4.34 | 22 | 22.42 | 1.38 | 51.97 |
| 2020-2021 | 2 | 4 | 627061 | 100 | 96.67 | 8.67 | 100 | 12.07 | 60.76 | 3.65 | 21.8 | 22.10 | 1.40 | 54.96 |
| 2020-2021 | 2 | 4 | NEISHABOUR | 100 | 97.4 | 8.83 | 100 | 12.32 | 68.94 | 4.36 | 22.99 | 23.40 | 1.38 | 61.11 |
| 2020-2021 | 2 | 4 | 624315 | 100 | 88.5 | 7.90 | 96 | 15.65 | 66.09 | 3.83 | 24.09 | 24.39 | 1.41 | 58.23 |
| 2020-2021 | 2 | 5 | 627103 | 60 | 11.66 | 2.77 | 19 | 0.61 | 49.27 | 1.95 | 16.22 | 16.34 | 1.45 | 46.70 |
| 2020-2021 | 2 | 5 | 623475 | 100 | 84.76 | 7.60 | 93 | 8.2 | 58.48 | 3.29 | 19.29 | 19.57 | 1.40 | 54.10 |
| 2020-2021 | 2 | 5 | 624596 | 80 | 67.82 | 6.23 | 71 | 11.61 | 57.81 | 4.19 | 21.85 | 22.25 | 1.38 | 52.30 |
| 2020-2021 | 2 | 5 | AKBARI | 100 | 71.42 | 6.90 | 75 | 8.38 | 59.47 | 3.7 | 19.98 | 20.32 | 1.39 | 54.66 |
| 2020-2021 | 2 | 5 | 623506 | 100 | 77.58 | 7.47 | 83 | 12.91 | 65.93 | 5.7 | 22.32 | 23.04 | 1.32 | 58.87 |
| 2020-2021 | 2 | 5 | 623510 | 100 | 79.79 | 7.30 | 84 | 8.72 | 51.07 | 4.84 | 19.52 | 20.11 | 1.33 | 47.10 |
| 2020-2021 | 2 | 5 | 627845 | 100 | 96.54 | 8.50 | 100 | 11.84 | 57.22 | 3.95 | 19.93 | 20.32 | 1.38 | 52.64 |
| 2020-2021 | 2 | 6 | 627856 | 40 | 3.3 | 1.87 | 9 | 0.26 | 49.85 | 1.9 | 18.97 | 19.06 | 1.47 | 46.35 |
| 2020-2021 | 2 | 6 | MAHDAVI | 100 | 94.11 | 8.40 | 100 | 17.28 | 68.89 | 3.93 | 23.26 | 23.59 | 1.40 | 60.96 |
| 2020-2021 | 2 | 6 | 627360 | 100 | 96.91 | 8.73 | 100 | 13.88 | 66.03 | 3.26 | 23.65 | 23.87 | 1.43 | 58.48 |
| 2020-2021 | 2 | 6 | 627883 | 100 | 84.78 | 7.80 | 89 | 12.36 | 67.09 | 3.9 | 22.27 | 22.61 | 1.40 | 60.07 |
| 2020-2021 | 2 | 6 | SHIROODI | 100 | 97.17 | 8.73 | 100 | 16.95 | 68.14 | 1.11 | 21.01 | 21.04 | 1.52 | 61.82 |
| 2020-2021 | 2 | 6 | 623379 | 100 | 82.3 | 7.63 | 87 | 8.77 | 57.12 | 5.1 | 23.57 | 24.12 | 1.36 | 50.80 |
| 2020-2021 | 2 | 6 | 624910 | 100 | 91.16 | 7.83 | 94 | 11.4 | 65.09 | 5.18 | 24.23 | 24.78 | 1.36 | 57.19 |
| 2020-2021 | 2 | 6 | MOGHAN3 | 100 | 96.95 | 8.57 | 100 | 13.48 | 64.52 | 4.03 | 25.08 | 25.40 | 1.41 | 56.36 |
| 2020-2021 | 2 | 6 | DASTJERDI | 100 | 95.75 | 8.47 | 100 | 4.64 | 71.04 | 3.43 | 23.57 | 23.82 | 1.43 | 62.50 |
| 2020-2021 | 2 | 7 | TAKAB | 60 | 12.2 | 2.70 | 19 | 0.82 | 50.46 | 4.01 | 16.73 | 17.20 | 1.34 | 47.56 |
| 2020-2021 | 2 | 7 | 623908 | 100 | 95.12 | 8.47 | 98 | 9.9 | 61.57 | 3.27 | 19.02 | 19.30 | 1.40 | 57.00 |
| 2020-2021 | 2 | 7 | RASHID | 100 | 95.44 | 8.47 | 98 | 14.76 | 69.9 | 3.73 | 22.21 | 22.52 | 1.40 | 62.41 |
| 2020-2021 | 2 | 7 | 624900 | 100 | 81.86 | 7.57 | 90 | 13.56 | 68.76 | 5.54 | 24.6 | 25.22 | 1.35 | 59.85 |
| 2020-2021 | 2 | 7 | 627054 | 100 | 95.43 | 8.30 | 100 | 8.96 | 52.91 | 6.14 | 22.86 | 23.67 | 1.31 | 47.30 |
| 2020-2021 | 2 | 7 | 626223 | 100 | 89.75 | 8.03 | 94 | 12.84 | 65.53 | 3.5 | 23.27 | 23.53 | 1.42 | 58.26 |
| 2020-2021 | 2 | 7 | 627189 | 100 | 96.8 | 8.73 | 100 | 15.93 | 68.65 | 2.88 | 22.09 | 22.28 | 1.44 | 61.54 |
| 2020-2021 | 2 | 7 | 627423 | 100 | 96.75 | 8.57 | 100 | 16.74 | 64.22 | 6.02 | 24.91 | 25.63 | 1.33 | 55.99 |
| 2020-2021 | 2 | 7 | ZARE | 100 | 93.11 | 8.40 | 98 | 12.03 | 60.91 | 4.23 | 21.67 | 22.08 | 1.38 | 55.11 |
| 2020-2021 | 2 | 8 | 627410 | 100 | 75.14 | 7.13 | 81 | 9.72 | 55.24 | 4.59 | 17.98 | 18.56 | 1.32 | 51.55 |
| 2020-2021 | 2 | 8 | DN11 | 100 | 96.88 | 8.70 | 98 | 14.58 | 69.89 | 3.33 | 23.6 | 23.83 | 1.43 | 61.60 |
| 2020-2021 | 2 | 8 | 625080 | 100 | 95.91 | 8.63 | 98 | 11.28 | 60.24 | 4.62 | 22.42 | 22.89 | 1.37 | 54.12 |
| 2020-2021 | 2 | 8 | HOMA | 100 | 85.33 | 7.43 | 97 | 12.75 | 70.41 | 3.94 | 22.82 | 23.16 | 1.40 | 62.43 |
| 2020-2021 | 2 | 8 | 627842 | 100 | 91.74 | 8.20 | 98 | 12.46 | 61.19 | 3.92 | 20.66 | 21.03 | 1.38 | 55.86 |
| 2020-2021 | 2 | 8 | 627043 | 100 | 62.76 | 6.30 | 71 | 6.75 | 58.62 | 2.04 | 17.82 | 17.94 | 1.46 | 54.90 |
| 2020-2021 | 2 | 8 | 624956 | 100 | 93.82 | 8.23 | 100 | 13.89 | 64.79 | 5.48 | 23.7 | 24.33 | 1.34 | 57.20 |
| 2020-2021 | 2 | 8 | 626261 | 100 | 98.04 | 8.80 | 100 | 13.7 | 57.07 | 3.85 | 22.4 | 22.73 | 1.40 | 51.42 |
| 2020-2021 | 2 | 8 | 628084 | 100 | 96.17 | 8.63 | 100 | 9.2 | 51.13 | 3.15 | 17.32 | 17.60 | 1.39 | 48.06 |
| 2020-2021 | 2 | 8 | VEE/NAC | 100 | 96.18 | 8.70 | 98 | 10.99 | 54.55 | 4.83 | 22.93 | 23.43 | 1.36 | 48.86 |
| 2020-2021 | 2 | 9 | 625047 | 100 | 96.42 | 8.63 | 100 | 13.65 | 66.97 | 3.81 | 23.14 | 23.45 | 1.41 | 59.49 |
| 2020-2021 | 2 | 9 | 626158 | 100 | 95.14 | 8.43 | 98 | 8.8 | 49.93 | 1.76 | 18.87 | 18.95 | 1.48 | 46.46 |
| 2020-2021 | 2 | 9 | 624925 | 100 | 91.97 | 8.50 | 96 | 14.19 | 64.62 | 2.58 | 24.29 | 24.43 | 1.46 | 57.01 |
| 2020-2021 | 2 | 9 | 621619 | 100 | 88.18 | 7.90 | 98 | 9.95 | 63.23 | 2.18 | 20.36 | 20.48 | 1.46 | 57.91 |
| 2020-2021 | 2 | 9 | 627299 | 100 | 92.12 | 8.30 | 96 | 15.98 | 65.57 | 2.84 | 24.84 | 25.00 | 1.46 | 57.45 |
| 2020-2021 | 2 | 9 | 623291 | 100 | 82.92 | 7.63 | 90 | 7.54 | 50.98 | 3.63 | 19.45 | 19.79 | 1.39 | 47.14 |
| 2020-2021 | 2 | 9 | 627849 | 100 | 88.59 | 7.67 | 100 | 11.43 | 60.43 | 2.16 | 22.33 | 22.43 | 1.47 | 54.51 |
| 2020-2021 | 2 | 9 | 626978 | 100 | 95.27 | 8.57 | 100 | 14.29 | 60.73 | 3.16 | 23.77 | 23.98 | 1.44 | 53.99 |
| 2020-2021 | 2 | 9 | AKOVA | 100 | 91.34 | 8.37 | 98 | 12.55 | 54.72 | 5.19 | 20.16 | 20.82 | 1.32 | 50.16 |
| 2020-2021 | 2 | 9 | 627072 | 100 | 97.52 | 8.80 | 100 | 12.74 | 63.53 | 3.28 | 22.23 | 22.47 | 1.42 | 57.16 |
| 2020-2021 | 2 | 10 | 626908 | 100 | 97.05 | 8.80 | 100 | 12.27 | 55.58 | 5.13 | 22.66 | 23.23 | 1.35 | 49.87 |
| 2020-2021 | 2 | 10 | DARAB1 | 100 | 86.76 | 7.80 | 95 | 7.12 | 50.31 | 3.16 | 22.32 | 22.54 | 1.43 | 45.44 |
| 2020-2021 | 2 | 10 | 623421 | 100 | 94.23 | 8.27 | 100 | 10.69 | 60.79 | 3.59 | 20.52 | 20.83 | 1.40 | 55.60 |
| 2020-2021 | 2 | 10 | 623090 | 100 | 94.09 | 8.43 | 100 | 14.03 | 64.35 | 2.02 | 23.21 | 23.30 | 1.48 | 57.41 |
| 2020-2021 | 2 | 10 | 626234 | 100 | 96.23 | 8.63 | 100 | 8.87 | 51.18 | 4.4 | 17.8 | 18.34 | 1.33 | 47.85 |
| 2020-2021 | 2 | 10 | 627853 | 100 | 99.59 | 9.00 | 100 | 12.44 | 60.64 | 3.14 | 21.32 | 21.55 | 1.42 | 55.13 |
| 2020-2021 | 2 | 10 | GHABOUS | 100 | 92.94 | 8.30 | 100 | 10.33 | 53.84 | 2.01 | 20.99 | 21.09 | 1.48 | 49.25 |
| 2020-2021 | 2 | 10 | 624805 | 40 | 6.93 | 2.13 | 11 | 0.5 | 52.31 | 3.62 | 19.94 | 20.27 | 1.39 | 48.18 |
| 2020-2021 | 2 | 10 | 621565 | 100 | 82.82 | 7.20 | 93 | 11.27 | 58.39 | 2.96 | 23.78 | 23.96 | 1.45 | 51.98 |
| 2020-2021 | 2 | 10 | SABALAN | 100 | 88.04 | 7.67 | 97 | 12.18 | 66.26 | 2.3 | 22.93 | 23.05 | 1.47 | 59.14 |
| 2020-2021 | 2 | 11 | 626706 | 100 | 93.48 | 8.47 | 100 | 13.59 | 66.9 | 4 | 23.15 | 23.49 | 1.40 | 59.41 |
| 2020-2021 | 2 | 11 | 622063 | 100 | 67.1 | 6.63 | 76 | 8.11 | 53.09 | 2 | 20.33 | 20.43 | 1.47 | 48.84 |
| 2020-2021 | 2 | 11 | 626747 | 100 | 93.47 | 8.37 | 98 | 18.76 | 66.7 | 2.55 | 24.53 | 24.66 | 1.47 | 58.56 |
| 2020-2021 | 2 | 11 | 626573 | 100 | 95.97 | 8.70 | 100 | 12.85 | 68.94 | 4 | 24.55 | 24.87 | 1.41 | 60.21 |
| 2020-2021 | 2 | 11 | 622247 | 0 | 0 | 1.00 | 0 | 0.23 | 49.85 | 3.98 | 19.4 | 19.80 | 1.37 | 46.08 |
| 2020-2021 | 2 | 11 | 627990 | 100 | 90.46 | 7.93 | 100 | 13.99 | 66.98 | 5.32 | 22.45 | 23.07 | 1.34 | 59.72 |
| 2020-2021 | 2 | 11 | MAROON | 100 | 94 | 8.30 | 100 | 11.76 | 55.23 | 4.15 | 19.51 | 19.95 | 1.36 | 50.99 |
| 2020-2021 | 2 | 11 | 626226 | 100 | 98.33 | 8.80 | 100 | 12.99 | 67.23 | 4.13 | 22.76 | 23.13 | 1.39 | 59.89 |
| 2020-2021 | 2 | 11 | 626924 | 100 | 72.44 | 6.87 | 83 | 12.14 | 64.66 | 4.75 | 25.03 | 25.48 | 1.38 | 56.43 |
| 2020-2021 | 2 | 11 | 622084 | 0 | 0 | 1.00 | 0 | 0.39 | 45.42 | 2 | 17.07 | 17.19 | 1.45 | 42.78 |
| 2020-2021 | 2 | 12 | 624849 | 100 | 84.26 | 7.90 | 90 | 10.79 | 59.88 | 3.63 | 19.75 | 20.08 | 1.39 | 55.14 |
| 2020-2021 | 2 | 12 | 624576 | 100 | 66.06 | 6.30 | 75 | 8.15 | 55.97 | 2.9 | 21.86 | 22.05 | 1.44 | 50.76 |
| 2020-2021 | 2 | 12 | 626904 | 100 | 81.2 | 7.23 | 88 | 15.91 | 64.45 | 3.17 | 23.8 | 24.01 | 1.44 | 57.10 |
| 2020-2021 | 2 | 12 | 626895 | 100 | 96.12 | 8.60 | 100 | 13.46 | 65.06 | 4.43 | 27 | 27.36 | 1.41 | 55.62 |
| 2020-2021 | 2 | 12 | 627057 | 100 | 88.46 | 7.77 | 97 | 13.01 | 65.57 | 3.27 | 27.88 | 28.07 | 1.45 | 55.58 |
| 2020-2021 | 2 | 12 | SIRVAN | 100 | 99.84 | 9.00 | 100 | 2.42 | 59.56 | 2.48 | 22.91 | 23.04 | 1.46 | 53.46 |
| 2020-2021 | 2 | 12 | ALBORZ | 100 | 95.93 | 8.50 | 100 | 15.6 | 60.33 | 1.54 | 22.45 | 22.50 | 1.50 | 54.39 |
| 2020-2021 | 2 | 12 | 627987 | 100 | 97.98 | 8.70 | 100 | 11.51 | 58.56 | 1.42 | 21.09 | 21.14 | 1.50 | 53.48 |
| 2020-2021 | 2 | 12 | 624582 | 100 | 98.83 | 8.90 | 100 | 14.83 | 67.48 | 1.94 | 22.44 | 22.52 | 1.48 | 60.44 |
| 2020-2021 | 2 | 13 | 623909 | 100 | 97.89 | 8.70 | 100 | 10.01 | 57.1 | 5.04 | 20.21 | 20.83 | 1.33 | 52.31 |
| 2020-2021 | 2 | 13 | MARVDASHT | 100 | 97.84 | 8.80 | 100 | 11.27 | 55.33 | 4.94 | 20.6 | 21.18 | 1.34 | 50.56 |
| 2020-2021 | 2 | 13 | GAHAR | 100 | 100 | 9.00 | 100 | 0.12 | 52.6 | 3.7 | 17.55 | 17.94 | 1.36 | 49.32 |
| 2020-2021 | 2 | 13 | 627616 | 100 | 98.83 | 8.90 | 100 | 11.93 | 59.15 | 2.59 | 18.67 | 18.85 | 1.43 | 55.01 |
| 2020-2021 | 2 | 13 | 627102 | 100 | 29.51 | 4.87 | 37 | 2.09 | 51.45 | 5.05 | 18.8 | 19.47 | 1.31 | 47.69 |
| 2020-2021 | 2 | 13 | NAZ | 100 | 100 | 9.00 | 100 | 13.92 | 61.8 | 3.76 | 20 | 20.35 | 1.38 | 56.72 |
| 2020-2021 | 2 | 13 | 623109 | 100 | 98.57 | 8.80 | 100 | 14.97 | 70.05 | 5.45 | 23.44 | 24.07 | 1.34 | 61.58 |
| 2020-2021 | 2 | 13 | 623091 | 100 | 46.98 | 5.33 | 59 | 9.31 | 58.2 | 2.76 | 21.49 | 21.67 | 1.44 | 52.92 |
| 2020-2021 | 2 | 13 | BISTON | 100 | 40.42 | 5.00 | 48 | 6.42 | 54.62 | 1.78 | 20.13 | 20.21 | 1.48 | 50.32 |
| 2020-2021 | 2 | 14 | 625433 | 100 | 90.75 | 8.00 | 97 | 12.41 | 61.47 | 3.1 | 21.86 | 22.08 | 1.43 | 55.59 |
| 2020-2021 | 2 | 14 | 623161 | 100 | 73.6 | 7.07 | 82 | 9.45 | 55.82 | 2.26 | 21.14 | 21.26 | 1.46 | 50.97 |
| 2020-2021 | 2 | 14 | AZAR2 | 100 | 88.02 | 7.67 | 97 | 11.33 | 59.69 | 1.87 | 22.1 | 22.18 | 1.49 | 53.99 |
| 2020-2021 | 2 | 14 | 626699 | 100 | 86.62 | 7.90 | 95 | 9.59 | 57 | 2.41 | 20.15 | 20.29 | 1.45 | 52.45 |
| 2020-2021 | 2 | 14 | 627873 | 100 | 87.74 | 7.87 | 98 | 9.75 | 55.33 | 1.34 | 19.7 | 19.75 | 1.50 | 51.16 |
| 2020-2021 | 2 | 14 | 622379 | 100 | 93.23 | 8.37 | 97 | 10.76 | 65.07 | 1.79 | 22.08 | 22.15 | 1.49 | 58.64 |
| 2020-2021 | 2 | 14 | 627587 | 100 | 85.71 | 7.90 | 90 | 11.22 | 60.74 | 3.17 | 23.42 | 23.63 | 1.44 | 54.18 |
| 2020-2021 | 2 | 14 | 623127 | 100 | 97.56 | 8.83 | 100 | 13.27 | 55.67 | 3.85 | 21.38 | 21.72 | 1.39 | 50.63 |
| 2020-2021 | 2 | 14 | 623507 | 100 | 100 | 9.00 | 100 | 10.98 | 61.37 | 2.88 | 19.76 | 19.97 | 1.43 | 56.51 |
| 2020-2021 | 2 | 15 | SHIRAZ | 100 | 97.14 | 8.67 | 100 | 15.48 | 62.64 | 4.67 | 23.69 | 24.15 | 1.38 | 55.52 |
| 2020-2021 | 2 | 15 | MIHAN | 100 | 98.74 | 8.73 | 100 | 11.72 | 67.03 | 3.02 | 22.81 | 23.01 | 1.44 | 59.80 |
| 2020-2021 | 2 | 15 | 621650 | 100 | 100 | 9.00 | 100 | 14.63 | 66.62 | 4.22 | 21.57 | 21.98 | 1.38 | 60.03 |
| 2020-2021 | 2 | 15 | 627066 | 100 | 100 | 9.00 | 100 | 10.94 | 58.42 | 3.26 | 19.77 | 20.04 | 1.41 | 53.84 |
| 2020-2021 | 2 | 15 | FONG | 100 | 92.57 | 8.20 | 100 | 14.87 | 62.99 | 2.37 | 23.11 | 23.23 | 1.47 | 56.30 |
| 2020-2021 | 2 | 15 | 627787 | 100 | 95.13 | 8.40 | 98 | 11.7 | 63.31 | 1.58 | 18.74 | 18.81 | 1.49 | 58.77 |
| 2020-2021 | 2 | 15 | 623139 | 100 | 95.08 | 8.50 | 100 | 13.16 | 59.99 | 3.03 | 24.68 | 24.87 | 1.45 | 52.89 |
| 2020-2021 | 2 | 15 | 621908 | 100 | 99.16 | 8.90 | 100 | 14.63 | 68.38 | 3.68 | 22.44 | 22.74 | 1.41 | 61.05 |
| 2020-2021 | 2 | 16 | HAMOON | 100 | 80.54 | 7.53 | 86 | 14.56 | 67.42 | 1.48 | 22.26 | 22.31 | 1.50 | 60.51 |
| 2020-2021 | 2 | 16 | ALVAND | 100 | 92.09 | 8.17 | 97 | 15.52 | 66.67 | 3.13 | 24.45 | 24.65 | 1.44 | 58.55 |
| 2020-2021 | 2 | 16 | 624944 | 100 | 89.15 | 7.80 | 97 | 13.05 | 68.05 | 4.74 | 22.32 | 22.82 | 1.36 | 60.74 |
| 2020-2021 | 2 | 16 | 624846 | 100 | 86 | 7.67 | 94 | 11.92 | 61.87 | 3.86 | 20.86 | 21.21 | 1.39 | 56.37 |
| 2020-2021 | 2 | 16 | PISHTAZ | 100 | 98.92 | 8.80 | 100 | 13.1 | 61.1 | 3.03 | 20.04 | 20.27 | 1.42 | 56.14 |
| 2020-2021 | 2 | 16 | 623428 | 100 | 98.07 | 8.80 | 100 | 13.83 | 64.66 | 3.54 | 23.51 | 23.78 | 1.42 | 57.41 |
| 2020-2021 | 2 | 16 | 622894 | 100 | 90.24 | 8.00 | 97 | 14.49 | 67.35 | 4.66 | 23.05 | 23.52 | 1.37 | 59.76 |
| 2020-2021 | 2 | 16 | ARTA | 100 | 97.44 | 8.67 | 100 | 14.28 | 65.82 | 3.51 | 22.31 | 22.58 | 1.41 | 59.03 |
| 2020-2021 | 2 | 16 | 623162 | 100 | 99.78 | 9.00 | 100 | 13.35 | 71.43 | 5.11 | 20.83 | 21.45 | 1.33 | 64.28 |
| 2020-2021 | 2 | 17 | 628189 | 100 | 51.22 | 5.67 | 57 | 9.4 | 52.57 | 4.19 | 23.83 | 24.20 | 1.40 | 46.76 |
| 2020-2021 | 2 | 17 | INIA | 100 | 99.13 | 8.90 | 100 | 13.3 | 66.56 | 2.08 | 25.18 | 25.27 | 1.49 | 58.09 |
| 2020-2021 | 2 | 17 | REYHANI | 100 | 54.58 | 5.73 | 71 | 9.51 | 58.26 | 3.61 | 24.13 | 24.40 | 1.42 | 51.65 |
| 2020-2021 | 2 | 17 | 626215 | 100 | 57.88 | 6.07 | 67 | 8.44 | 60.78 | 2.59 | 24.08 | 24.22 | 1.46 | 53.90 |
| 2020-2021 | 2 | 17 | KARIM | 100 | 94.33 | 8.40 | 100 | 11.18 | 53.28 | 3.42 | 20.34 | 20.63 | 1.40 | 48.93 |
| 2020-2021 | 2 | 17 | 627414 | 100 | 89.3 | 7.90 | 100 | 12.33 | 64.86 | 3.38 | 23.45 | 23.69 | 1.43 | 57.62 |
| 2020-2021 | 2 | 17 | 627399 | 100 | 82.18 | 7.73 | 90 | 12.94 | 70.01 | 1.58 | 22.01 | 22.07 | 1.50 | 62.77 |
| 2020-2021 | 2 | 17 | 623318 | 100 | 45.81 | 5.37 | 53 | 5.12 | 55.51 | 2.36 | 18.17 | 18.32 | 1.44 | 51.88 |
| 2020-2021 | 2 | 17 | 627963 | 100 | 81.13 | 7.37 | 88 | 14.14 | 71.64 | 5.32 | 22.82 | 23.43 | 1.34 | 63.21 |
| 2020-2021 | 2 | 18 | KARAJ1 | 100 | 91.75 | 8.30 | 100 | 12.84 | 72.51 | 3.8 | 22.54 | 22.86 | 1.40 | 64.25 |
| 2020-2021 | 2 | 18 | DEZ | 100 | 86.55 | 7.87 | 93 | 10.05 | 64.65 | 3.46 | 22.75 | 23.01 | 1.42 | 57.82 |
| 2020-2021 | 2 | 18 | 621706 | 100 | 96.74 | 8.47 | 100 | 15.6 | 69.96 | 4.17 | 25.14 | 25.48 | 1.41 | 60.61 |
| 2020-2021 | 2 | 18 | 626358 | 100 | 97.18 | 8.63 | 100 | 12.47 | 68.82 | 4.6 | 23.54 | 23.99 | 1.38 | 60.66 |
| 2020-2021 | 2 | 18 | 623980 | 100 | 93.75 | 8.17 | 100 | 11.7 | 63.45 | 3.94 | 21.58 | 21.94 | 1.39 | 57.37 |
| 2020-2021 | 2 | 18 | 626776 | 100 | 86.78 | 7.67 | 97 | 8.05 | 57.56 | 1.93 | 17.46 | 17.57 | 1.46 | 54.07 |
| 2020-2021 | 2 | 18 | 625127 | 100 | 92.59 | 8.30 | 98 | 12.67 | 60.31 | 1.51 | 21.07 | 21.12 | 1.50 | 55.04 |
| 2020-2021 | 2 | 18 | CHAMRAN2 | 100 | 58.78 | 6.20 | 69 | 14.64 | 62.85 | 4.27 | 22.63 | 23.03 | 1.38 | 56.29 |
| 2020-2021 | 2 | 18 | NICKNEJAD | 90 | 23.49 | 3.93 | 30 | 2.87 | 53.21 | 4.55 | 20.7 | 21.19 | 1.35 | 48.63 |
| 2020-2021 | 2 | 18 | 623503 | 100 | 60.78 | 6.23 | 75 | 8.99 | 63.99 | 3.73 | 20.2 | 20.54 | 1.39 | 58.54 |
| 2020-2021 | 2 | 19 | FRONTANA | 0 | 0 | 1.00 | 0 | 18.22 | 68.38 | 2.33 | 25 | 25.11 | 1.48 | 59.62 |
| 2020-2021 | 2 | 19 | 627881 | 100 | 51.39 | 5.83 | 60 | 9.47 | 61.83 | 3.57 | 22.91 | 23.19 | 1.42 | 55.34 |
| 2020-2021 | 2 | 19 | 623377 | 100 | 85.21 | 7.90 | 90 | 12.3 | 72.86 | 1.67 | 23.11 | 23.17 | 1.50 | 64.31 |
| 2020-2021 | 2 | 19 | MV17 | 100 | 85.73 | 8.13 | 97 | 11.37 | 55.43 | 2.67 | 20.09 | 20.27 | 1.44 | 51.04 |
| 2020-2021 | 2 | 19 | 627948 | 100 | 94.78 | 8.40 | 98 | 13.14 | 61.98 | 3.39 | 22.7 | 22.95 | 1.42 | 55.59 |
| 2020-2021 | 2 | 19 | BAHAR | 100 | 94.47 | 8.30 | 100 | 12.74 | 57.29 | 3.2 | 22.65 | 22.87 | 1.43 | 51.55 |
| 2020-2021 | 2 | 19 | 624818 | 100 | 65.77 | 6.50 | 75 | 5.41 | 43.93 | 2.46 | 17.26 | 17.43 | 1.43 | 41.28 |
| 2020-2021 | 2 | 19 | 623345 | 100 | 81.72 | 7.50 | 88 | 13.56 | 71.28 | 4.02 | 22.91 | 23.26 | 1.40 | 63.04 |
| 2020-2021 | 2 | 20 | 627908 | 100 | 83.23 | 7.83 | 87 | 11.81 | 60.38 | 4.8 | 20.5 | 21.05 | 1.34 | 55.13 |
| 2020-2021 | 2 | 20 | 623169 | 50 | 24.16 | 3.30 | 30 | 2.76 | 55.73 | 3.79 | 21.75 | 22.08 | 1.40 | 50.53 |
| 2020-2021 | 2 | 20 | ATRAK | 90 | 51.23 | 5.33 | 58 | 6.11 | 53.22 | 3.88 | 22.08 | 22.42 | 1.40 | 48.13 |
| 2020-2021 | 2 | 20 | 627356 | 100 | 82.9 | 7.63 | 88 | 10.94 | 63.61 | 2.72 | 21.77 | 21.94 | 1.45 | 57.51 |
| 2020-2021 | 2 | 20 | 626943 | 100 | 51.08 | 5.53 | 63 | 4.73 | 50.04 | 2.4 | 18.73 | 18.88 | 1.44 | 46.59 |
| 2020-2021 | 2 | 20 | 627417 | 100 | 93.02 | 8.30 | 98 | 7.56 | 57.3 | 2.77 | 18.09 | 18.30 | 1.42 | 53.54 |
| 2020-2021 | 2 | 20 | 628088 | 100 | 83.91 | 7.73 | 88 | 11.58 | 64.62 | 2.44 | 19.81 | 19.96 | 1.45 | 59.38 |
| 2020-2021 | 2 | 20 | BAYAT | 100 | 89.47 | 8.00 | 92 | 14.87 | 62.17 | 2.42 | 23.59 | 23.71 | 1.47 | 55.35 |
| 2020-2021 | 2 | 20 | FALAT | 100 | 93.7 | 8.40 | 100 | 12.25 | 55.84 | 4.31 | 22.45 | 22.86 | 1.38 | 50.27 |
| 2020-2021 | 2 | 20 | 626933 | 100 | 96.42 | 8.60 | 100 | 11.11 | 56.39 | 2.74 | 22.34 | 22.51 | 1.45 | 50.92 |
| 2020-2021 | 2 | 21 | 623125 | 100 | 68.11 | 6.40 | 77 | 10.63 | 62.59 | 4.53 | 22.93 | 23.37 | 1.38 | 55.89 |
| 2020-2021 | 2 | 21 | DARAB2 | 100 | 92.79 | 8.40 | 98 | 5.72 | 45.91 | 3.87 | 18.09 | 18.50 | 1.36 | 42.83 |
| 2020-2021 | 2 | 21 | 624980 | 100 | 84.91 | 7.60 | 91 | 10.09 | 60.11 | 4.93 | 23.74 | 24.25 | 1.37 | 53.32 |
| 2020-2021 | 2 | 21 | 625281 | 100 | 35 | 5.00 | 44 | 3.23 | 55.73 | 2.09 | 18.87 | 18.99 | 1.46 | 51.83 |
| 2020-2021 | 2 | 21 | TAJAN | 100 | 97.31 | 8.43 | 100 | 11.2 | 59.33 | 4.17 | 19.05 | 19.50 | 1.36 | 54.90 |
| 2020-2021 | 2 | 21 | 623382 | 100 | 83.94 | 7.50 | 90 | 10.14 | 66.52 | 3.48 | 24.43 | 24.68 | 1.43 | 58.41 |
| 2020-2021 | 2 | 21 | 624580 | 100 | 20.69 | 3.83 | 35 | 1.53 | 48 | 3.59 | 19.36 | 19.69 | 1.39 | 44.40 |
| 2020-2021 | 2 | 21 | KAVIR | 100 | 61.64 | 6.13 | 65 | 7.44 | 57.99 | 3.03 | 22.5 | 22.70 | 1.44 | 52.25 |
| 2020-2021 | 2 | 21 | 626883 | 100 | 92.5 | 8.40 | 92 | 14.28 | 70.71 | 1.13 | 23.19 | 23.22 | 1.52 | 62.62 |
| 2020-2021 | 2 | 21 | BAM | 100 | 65.81 | 6.67 | 71 | 12.51 | 67.79 | 2.34 | 22.77 | 22.89 | 1.47 | 60.49 |
| 2020-2021 | 2 | 22 | 623136 | 100 | 68.7 | 6.63 | 75 | 7.51 | 61.64 | 3.53 | 21.2 | 21.49 | 1.41 | 56.03 |
| 2020-2021 | 2 | 22 | KAVEH | 100 | 92.52 | 8.50 | 96 | 12.44 | 57.57 | 2.64 | 23.51 | 23.66 | 1.46 | 51.42 |
| 2020-2021 | 2 | 22 | 626923 | 90 | 43.13 | 4.90 | 55 | 5.83 | 66.58 | 2.33 | 22.83 | 22.95 | 1.47 | 59.46 |
| 2020-2021 | 2 | 22 | MOGHAN1 | 100 | 63 | 6.57 | 68 | 6.21 | 62.17 | 2.62 | 19.13 | 19.31 | 1.43 | 57.53 |
| 2020-2021 | 2 | 22 | 627099 | 100 | 82.51 | 7.63 | 88 | 9.56 | 56.12 | 1.39 | 17.76 | 17.81 | 1.49 | 52.64 |
| 2020-2021 | 2 | 22 | 626260 | 100 | 33.66 | 4.73 | 42 | 3.61 | 49.88 | 3.75 | 17.92 | 18.31 | 1.36 | 46.64 |
| 2020-2021 | 2 | 22 | DARYA | 100 | 19.75 | 4.20 | 29 | 10.33 | 55.14 | 4.03 | 21.43 | 21.81 | 1.38 | 50.12 |
| 2020-2021 | 2 | 22 | 623176 | 100 | 29.66 | 4.43 | 40 | 5.31 | 54.72 | 3.11 | 22.2 | 22.42 | 1.43 | 49.47 |
| 2020-2021 | 2 | 22 | 621492 | 100 | 22.23 | 4.17 | 31 | 3.23 | 62.23 | 4.21 | 23.58 | 23.95 | 1.39 | 55.28 |
| 2020-2021 | 2 | 22 | URUOM | 100 | 90.3 | 7.93 | 100 | 11.18 | 63.47 | 3.18 | 21.41 | 21.64 | 1.42 | 57.54 |
| 2020-2021 | 2 | 23 | 627723 | 100 | 63.5 | 6.53 | 74 | 8.36 | 59.36 | 4.51 | 20.89 | 21.37 | 1.36 | 54.08 |
| 2020-2021 | 2 | 23 | BEZOSTAYA | 90 | 9.99 | 2.63 | 19 | 1.35 | 56.38 | 5.48 | 19.39 | 20.15 | 1.30 | 51.95 |
| 2020-2021 | 2 | 23 | 626565 | 100 | 90.16 | 8.10 | 92 | 8.36 | 52.54 | 2.62 | 17.35 | 17.55 | 1.42 | 49.40 |
| 2020-2021 | 2 | 23 | 4820 | 100 | 72.79 | 7.13 | 80 | 10.76 | 66.37 | 2.58 | 22.31 | 22.46 | 1.46 | 59.56 |
| 2020-2021 | 2 | 23 | OFOG | 100 | 56.93 | 5.90 | 66 | 10.32 | 62.39 | 2.68 | 22.99 | 23.15 | 1.45 | 55.84 |
| 2020-2021 | 2 | 23 | 624946 | 40 | 10.5 | 2.47 | 13 | 2.41 | 61.46 | 2.09 | 21.85 | 21.95 | 1.48 | 55.65 |
| 2020-2021 | 2 | 23 | 624894 | 100 | 86.33 | 7.77 | 91 | 6.62 | 54.73 | 1.71 | 16.47 | 16.56 | 1.47 | 51.80 |
| 2020-2021 | 2 | 23 | AZAR1 | 100 | 43.79 | 5.50 | 52 | 4.37 | 47.76 | 3.09 | 18.25 | 18.51 | 1.40 | 44.58 |
| 2020-2021 | 2 | 23 | 626958 | 100 | 34.47 | 4.80 | 45 | 6.82 | 66.61 | 1.49 | 23.49 | 23.54 | 1.51 | 59.15 |
| 2020-2021 | 2 | 23 | 623417 | 100 | 58.04 | 6.13 | 63 | 7.3 | 61.97 | 2.33 | 18.05 | 18.20 | 1.44 | 57.84 |
| 2020-2021 | 2 | 24 | 624378 | 100 | 35.2 | 4.87 | 43 | 4.42 | 57.06 | 3.6 | 18.97 | 19.31 | 1.38 | 52.92 |
| 2020-2021 | 2 | 24 | SISTAN | 100 | 51.84 | 5.67 | 62 | 8.17 | 51.34 | 3.33 | 18.54 | 18.84 | 1.39 | 47.82 |
| 2020-2021 | 2 | 24 | 626872 | 100 | 72.8 | 7.27 | 84 | 6.11 | 49.67 | 3.07 | 15.57 | 15.87 | 1.38 | 47.23 |
| 2020-2021 | 2 | 24 | 624381 | 100 | 59.93 | 6.17 | 67 | 8.84 | 68.76 | 3.29 | 22.76 | 23.00 | 1.43 | 61.21 |
| 2020-2021 | 2 | 24 | 625810 | 100 | 32.7 | 4.67 | 41 | 3.98 | 65.35 | 3.51 | 21.77 | 22.05 | 1.41 | 58.93 |
| 2020-2021 | 2 | 24 | 622311 | 100 | 79.9 | 7.37 | 87 | 10.19 | 61.7 | 3.29 | 18.63 | 18.92 | 1.40 | 57.28 |
| 2020-2021 | 2 | 24 | 627385 | 100 | 79.43 | 7.13 | 88 | 13.59 | 65.3 | 3.63 | 22.6 | 22.89 | 1.41 | 58.43 |
| 2020-2021 | 2 | 24 | 623905 | 100 | 46.43 | 5.33 | 58 | 4.51 | 50.69 | 3.61 | 18.84 | 19.18 | 1.38 | 47.09 |
| 2020-2021 | 2 | 24 | 627460 | 100 | 87.99 | 7.90 | 93 | 16.15 | 61.79 | 4 | 21.35 | 21.72 | 1.39 | 56.05 |
| 2020-2021 | 2 | 25 | 627760 | 100 | 55.32 | 5.73 | 62 | 5.7 | 69.1 | 2.32 | 24.02 | 24.13 | 1.47 | 60.79 |
| 2020-2021 | 2 | 25 | 625362 | 60 | 43.85 | 4.50 | 49 | 7.54 | 57.5 | 2.97 | 24.15 | 24.33 | 1.45 | 51.03 |
| 2020-2021 | 2 | 25 | 623274 | 100 | 95.7 | 8.70 | 96 | 12.42 | 64.74 | 3.1 | 22.04 | 22.26 | 1.43 | 58.30 |
| 2020-2021 | 2 | 25 | 626736 | 100 | 57.91 | 6.13 | 68 | 7.44 | 56.11 | 2.55 | 23.15 | 23.29 | 1.46 | 50.31 |
| 2020-2021 | 2 | 25 | 624585 | 80 | 20.68 | 3.47 | 30 | 1.41 | 48.72 | 4.17 | 18.21 | 18.68 | 1.35 | 45.42 |
| 2020-2021 | 2 | 25 | KOOHDASHT | 100 | 67.47 | 6.70 | 76 | 12.19 | 62.1 | 1.82 | 25.17 | 25.24 | 1.50 | 54.47 |
| 2020-2021 | 2 | 25 | NAVID | 100 | 80.63 | 7.60 | 86 | 14.5 | 68.63 | 1.53 | 22.76 | 22.81 | 1.50 | 61.21 |
| 2020-2021 | 2 | 25 | KARAJ2 | 100 | 92.05 | 8.50 | 93 | 14.95 | 67.39 | 1.21 | 21.43 | 21.46 | 1.51 | 60.96 |
| 2020-2021 | 2 | 25 | CHAMRAN | 100 | 96.49 | 8.60 | 100 | 0.12 | 47.96 | 1.5 | 15.56 | 15.63 | 1.47 | 45.66 |
| 2020-2021 | 2 | 25 | 624864 | 100 | 33.8 | 4.73 | 41 | 5.51 | 61.44 | 3.76 | 20.42 | 20.76 | 1.39 | 56.21 |
| 2020-2021 | 2 | 26 | 625123 | 100 | 95.31 | 8.67 | 97 | 12.32 | 56.22 | 2.65 | 20.03 | 20.20 | 1.44 | 51.78 |
| 2020-2021 | 2 | 26 | CASGOGEN | 10 | 1.17 | 1.33 | 3 | 8.55 | 51.48 | 2.66 | 17.36 | 17.56 | 1.42 | 48.40 |
| 2020-2021 | 2 | 26 | 626764 | 100 | 62.01 | 6.67 | 67 | 4.49 | 54.54 | 3.12 | 16.89 | 17.18 | 1.39 | 51.40 |
| 2020-2021 | 2 | 26 | 624804 | 100 | 36.62 | 4.90 | 45 | 4.51 | 58.13 | 3.64 | 18.72 | 19.07 | 1.38 | 53.99 |
| 2020-2021 | 2 | 26 | 626846 | 100 | 82.89 | 7.60 | 88 | 6.69 | 50.73 | 3.58 | 16.4 | 16.79 | 1.36 | 47.95 |
| 2020-2021 | 2 | 26 | ZARRIN | 100 | 31.24 | 4.60 | 40 | 4.71 | 52.94 | 4.57 | 19.41 | 19.94 | 1.34 | 48.89 |
| 2020-2021 | 2 | 26 | 626814 | 100 | 72.02 | 6.83 | 79 | 7.63 | 56.78 | 2.43 | 21.97 | 22.10 | 1.46 | 51.46 |
| 2020-2021 | 2 | 26 | 626825 | 100 | 89.14 | 8.13 | 91 | 12.07 | 62.04 | 2.59 | 22.19 | 22.34 | 1.45 | 55.95 |
| 2020-2021 | 2 | 26 | DEYHEM | 100 | 55.72 | 6.10 | 62 | 8.65 | 52.59 | 3.59 | 17.48 | 17.84 | 1.37 | 49.34 |
| 2020-2021 | 2 | 26 | 624251 | 100 | 34.75 | 4.73 | 42 | 6.22 | 58.44 | 2.72 | 21.91 | 22.08 | 1.45 | 52.94 |
| 2020-2021 | 2 | 27 | 623953 | 10 | 1.17 | 1.33 | 3 | 0.22 | 67.74 | 2.1 | 25.51 | 25.60 | 1.49 | 58.82 |
| 2020-2021 | 2 | 27 | 626855 | 100 | 91.19 | 8.27 | 98 | 17.18 | 64.49 | 2.54 | 24.62 | 24.75 | 1.47 | 56.72 |
| 2020-2021 | 2 | 27 | 623008 | 100 | 82.41 | 7.67 | 90 | 12.14 | 56.01 | 2.77 | 22.04 | 22.21 | 1.45 | 50.72 |
| 2020-2021 | 2 | 27 | 621669 | 100 | 92 | 8.07 | 98 | 10.37 | 59.12 | 4.6 | 19.04 | 19.59 | 1.33 | 54.67 |
| 2020-2021 | 2 | 27 | SHARYAR | 100 | 97.51 | 8.80 | 98 | 7.8 | 69.25 | 4.84 | 24.2 | 24.68 | 1.37 | 60.57 |
| 2020-2021 | 2 | 27 | ADL | 100 | 96.15 | 8.50 | 100 | 9.65 | 61.86 | 2.14 | 18.57 | 18.69 | 1.46 | 57.53 |
| 2020-2021 | 2 | 27 | PANJAMO62 | 100 | 79.03 | 7.37 | 87 | 12.58 | 69.63 | 3.37 | 20.43 | 20.71 | 1.41 | 63.24 |
| 2020-2021 | 2 | 27 | 624939 | 100 | 77 | 7.27 | 87 | 10.63 | 66.78 | 4.05 | 19.67 | 20.08 | 1.37 | 61.18 |
| 2020-2021 | 2 | 27 | PISHGAM | 100 | 69.26 | 6.53 | 80 | 13.17 | 65.53 | 4.16 | 23.42 | 23.79 | 1.40 | 58.12 |
| 2020-2021 | 2 | 27 | 626360 | 100 | 79.22 | 7.30 | 88 | 9.54 | 57.19 | 4.4 | 21.78 | 22.22 | 1.37 | 51.77 |
| 2020-2021 | 2 | 28 | 624983 | 100 | 58.22 | 6.50 | 68 | 8.19 | 63.54 | 5.16 | 22.72 | 23.30 | 1.35 | 56.73 |
| 2020-2021 | 2 | 28 | SIVAND | 100 | 67.82 | 6.67 | 78 | 12.51 | 59.14 | 4.5 | 23.97 | 24.39 | 1.39 | 52.41 |
| 2020-2021 | 2 | 28 | 627551 | 100 | 7.96 | 2.73 | 20 | 0.51 | 45.34 | 4.65 | 19.1 | 19.66 | 1.33 | 41.91 |
| 2020-2021 | 2 | 28 | SHAHI | 100 | 76.81 | 7.43 | 87 | 11.3 | 58.45 | 4.53 | 22.33 | 22.78 | 1.37 | 52.61 |
| 2020-2021 | 2 | 28 | 621717 | 100 | 23.5 | 4.13 | 37 | 3.97 | 60.36 | 2.31 | 23.85 | 23.96 | 1.47 | 53.68 |
| 2020-2021 | 2 | 28 | ARVAND | 100 | 82.81 | 7.43 | 95 | 8.09 | 55.87 | 2.14 | 18.52 | 18.64 | 1.46 | 52.09 |
| 2020-2021 | 2 | 28 | MORVARID | 100 | 70.81 | 6.97 | 77 | 12.2 | 57.5 | 2.12 | 23.49 | 23.59 | 1.48 | 51.39 |
| 2020-2021 | 2 | 28 | 624863 | 100 | 19.61 | 3.83 | 25 | 2.28 | 63.14 | 2.33 | 22.4 | 22.52 | 1.47 | 56.80 |
| 2020-2021 | 2 | 28 | 626881 | 100 | 64.53 | 6.50 | 73 | 8.98 | 62.92 | 3.61 | 24.04 | 24.31 | 1.42 | 55.66 |
| 2020-2021 | 2 | 28 | TOUBARI | 100 | 60.83 | 6.27 | 69 | 8.32 | 59.1 | 1.99 | 20.24 | 20.34 | 1.47 | 54.32 |
| 2020-2021 | 2 | 29 | SHINGHAI | 100 | 51.87 | 5.83 | 57 | 9.51 | 56.17 | 4.97 | 20.38 | 20.98 | 1.33 | 51.41 |
| 2020-2021 | 2 | 29 | KARAJ3 | 100 | 70.39 | 6.90 | 76 | 7.75 | 60.81 | 4.79 | 20.58 | 21.13 | 1.34 | 55.48 |
| 2020-2021 | 2 | 29 | 624947 | 100 | 70.4 | 6.77 | 77 | 7.78 | 56.25 | 4.47 | 19.68 | 20.18 | 1.35 | 51.82 |
| 2020-2021 | 2 | 29 | GOLESTAN | 100 | 87.93 | 7.63 | 97 | 11.02 | 64.68 | 3.11 | 18.63 | 18.89 | 1.41 | 59.95 |
| 2020-2021 | 2 | 29 | 624963 | 100 | 43.25 | 5.30 | 55 | 4.93 | 58.72 | 3.89 | 21.03 | 21.39 | 1.39 | 53.51 |
| 2020-2021 | 2 | 29 | AZADI | 100 | 91.44 | 8.17 | 100 | 12.38 | 54.12 | 3.07 | 22.48 | 22.69 | 1.44 | 48.82 |
| 2020-2021 | 2 | 29 | 623123 | 100 | 91.43 | 8.30 | 96 | 13.44 | 63.96 | 4.42 | 22.88 | 23.30 | 1.38 | 57.08 |
| 2020-2021 | 2 | 29 | AFLAK | 100 | 94.51 | 8.40 | 100 | 14.21 | 68.32 | 1.8 | 25.09 | 25.15 | 1.50 | 59.55 |
| 2020-2021 | 2 | 29 | KHAZAR1 | 100 | 62.79 | 6.30 | 73 | 11.02 | 61.52 | 1.61 | 22.47 | 22.53 | 1.50 | 55.41 |
| 2020-2021 | 2 | 30 | 623069 | 90 | 36.79 | 4.97 | 43 | 9.2 | 65.5 | 2.6 | 24.74 | 24.88 | 1.47 | 57.47 |
| 2020-2021 | 2 | 30 | ZAGROS | 100 | 53.74 | 6.10 | 64 | 9.22 | 60.07 | 2.01 | 19.13 | 19.24 | 1.47 | 55.68 |
| 2020-2021 | 2 | 30 | 624901 | 100 | 40.45 | 5.33 | 47 | 5.78 | 56.71 | 2.29 | 19.09 | 19.23 | 1.45 | 52.63 |
| 2020-2021 | 2 | 30 | SISON | 80 | 29.65 | 4.03 | 35 | 11.78 | 66.79 | 2.74 | 22.5 | 22.67 | 1.45 | 59.79 |
| 2020-2021 | 2 | 30 | 624215 | 30 | 3.65 | 1.60 | 6 | 0.41 | 50.9 | 2.05 | 16.58 | 16.71 | 1.45 | 48.14 |
| 2020-2021 | 2 | 30 | GHODS | 100 | 83.91 | 7.80 | 88 | 11.21 | 57.59 | 3.48 | 20.27 | 20.57 | 1.40 | 52.87 |
| 2020-2021 | 2 | 30 | 624861 | 100 | 58.11 | 6.07 | 68 | 9.89 | 67.51 | 3.02 | 22.79 | 22.99 | 1.44 | 60.20 |
| 2020-2021 | 2 | 30 | RIJAW | 100 | 91.46 | 8.03 | 96 | 15.07 | 64.9 | 3.9 | 24.38 | 24.69 | 1.41 | 57.09 |
| 2020-2021 | 2 | 30 | SPAHAN | 100 | 89.07 | 8.03 | 94 | 9.99 | 58.87 | 2.62 | 21.22 | 21.38 | 1.45 | 53.64 |
| 2020-2021 | 2 | 31 | 627055 | 100 | 58.37 | 6.17 | 67 | 7.48 | 57.56 | 4.02 | 18.6 | 19.03 | 1.36 | 53.49 |
| 2020-2021 | 2 | 31 | 626932 | 100 | 49.15 | 5.80 | 54 | 9.41 | 70.28 | 4.01 | 23.53 | 23.87 | 1.40 | 61.88 |
| 2020-2021 | 2 | 31 | PARSI | 100 | 100 | 9.00 | 100 | 14.94 | 68.33 | 4.38 | 21.76 | 22.20 | 1.37 | 61.33 |
| 2020-2021 | 2 | 31 | 624838 | 100 | 52.32 | 5.67 | 61 | 7.14 | 63.62 | 4.67 | 23.07 | 23.54 | 1.37 | 56.67 |
| 2020-2021 | 2 | 31 | 621736 | 90 | 26.79 | 4.20 | 33 | 4.67 | 62.62 | 2.35 | 23.62 | 23.74 | 1.47 | 55.72 |
| 2020-2021 | 2 | 31 | 621869 | 90 | 41.33 | 4.73 | 45 | 5.86 | 66.61 | 2.16 | 21.67 | 21.78 | 1.47 | 60.14 |
| 2020-2021 | 2 | 31 | 628012 | 90 | 11.6 | 3.00 | 20 | 1.76 | 53.61 | 2.91 | 17.74 | 17.98 | 1.41 | 50.25 |
| 2020-2021 | 2 | 31 | 622099 | 0 | 0 | 1.00 | 0 | 0.15 | 47.43 | 1.51 | 18.23 | 18.29 | 1.49 | 44.34 |
| 2020-2021 | 2 | 31 | 622105 | 0 | 0 | 1.00 | 0 | 0.11 | 46.89 | 5.76 | 19.71 | 20.53 | 1.29 | 43.06 |
| 2020-2021 | 2 | 32 | 621420 | 100 | 44.97 | 5.43 | 51 | 5.11 | 58.96 | 2.91 | 21.98 | 22.17 | 1.44 | 53.35 |
| 2020-2021 | 2 | 32 | 623266 | 60 | 19.77 | 3.00 | 20 | 1.91 | 53.64 | 2.14 | 19.1 | 19.22 | 1.46 | 49.81 |
| 2020-2021 | 2 | 32 | 621712 | 100 | 68.42 | 6.60 | 79 | 7.9 | 52.76 | 4.81 | 17.98 | 18.61 | 1.31 | 49.23 |
| 2020-2021 | 2 | 32 | 620903 | 100 | 65 | 6.67 | 70 | 6.45 | 49.39 | 1.62 | 17.4 | 17.48 | 1.48 | 46.46 |
| 2020-2021 | 2 | 32 | 625139 | 60 | 22.63 | 3.43 | 31 | 3.21 | 53.47 | 2.41 | 15.76 | 15.94 | 1.42 | 50.81 |
| 2020-2021 | 2 | 32 | 622098 | 90 | 57.08 | 5.63 | 64 | 11.26 | 69.49 | 4.31 | 22.88 | 23.28 | 1.38 | 61.62 |
| 2020-2021 | 2 | 32 | 625263 | 0 | 0 | 1.00 | 0 | 0.34 | 56.33 | 1.48 | 18.99 | 19.05 | 1.49 | 52.36 |
| 2020-2021 | 2 | 32 | 622264 | 0 | 0 | 1.00 | 0 | 0.13 | 51.26 | 4.92 | 19.49 | 20.10 | 1.32 | 47.28 |
| 2020-2021 | 2 | 32 | 622272 | 0 | 0 | 1.00 | 0 | 0.12 | 52.24 | 2.36 | 19.24 | 19.38 | 1.45 | 48.46 |
| 2020-2021 | 2 | 32 | 627905 | 40 | 8.83 | 2.20 | 12 | 1.06 | 55.77 | 1.14 | 15.43 | 15.47 | 1.50 | 53.14 |


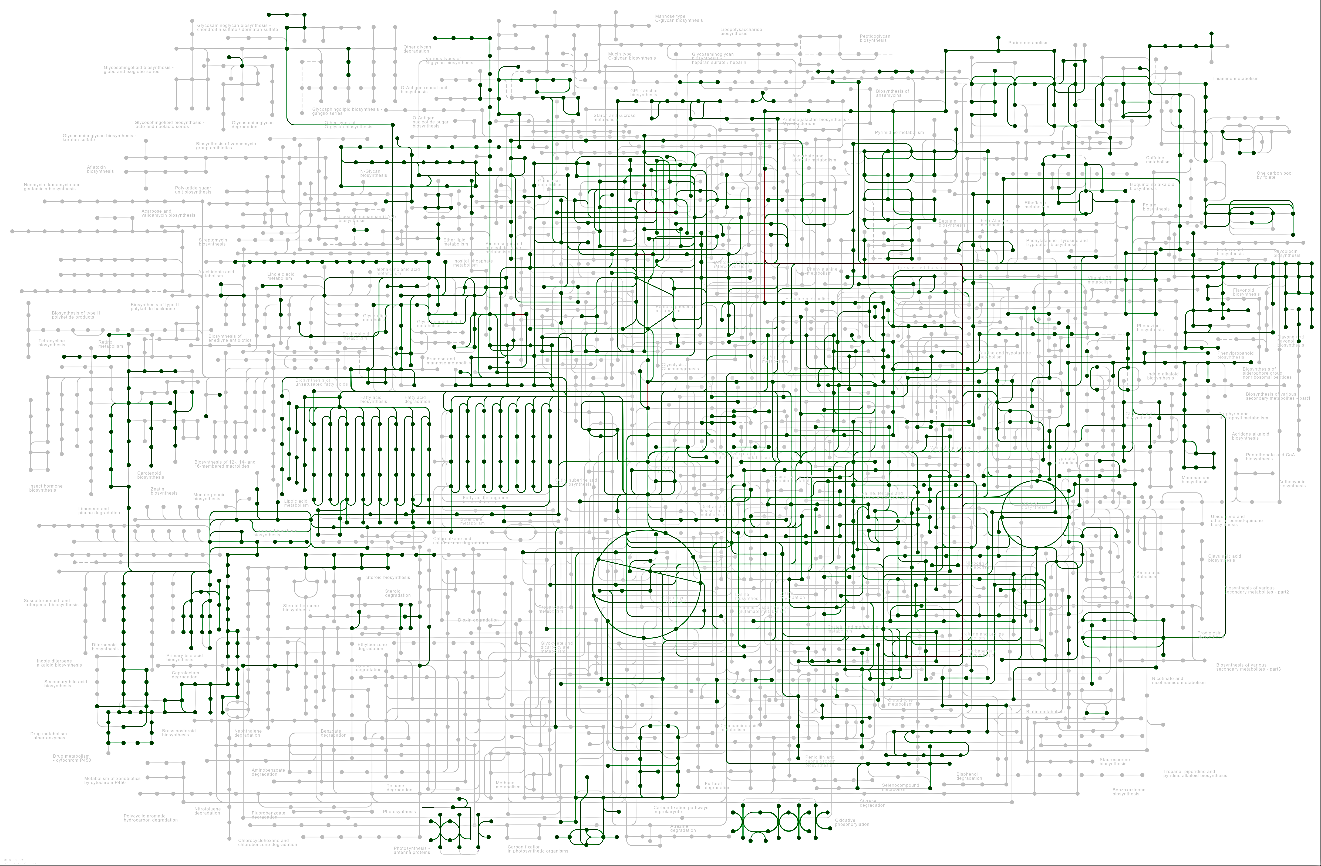


**Supplementary Fig 1.** The KEGG pathway of metabolic pathways.


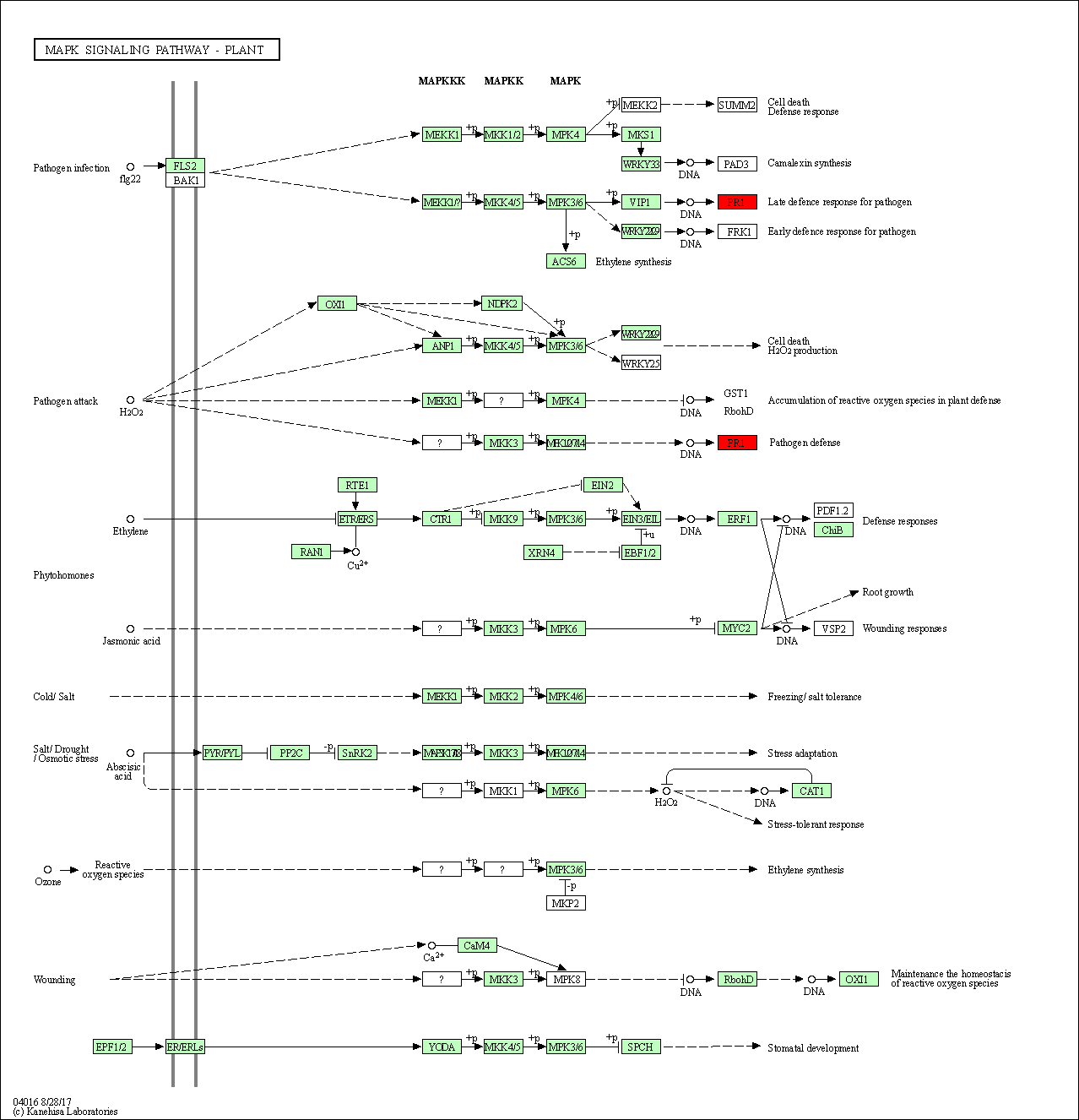


**Supplementary Fig 2.** The KEGG pathway of MAPK signaling.

- Reference pathway: this is the original version; white boxes are hyperlinked to KO, ENZYME, and REACTION entries in metabolic pathways; they are hyperlinked to KO entries in non-metabolic pathways.
- Reference pathway (KO): blue boxes are hyperlinked to KO entries that are selected from the original version.
- Reference pathway (EC): blue boxes are hyperlinked to ENZYME entries that are selected from the original version.
- Reference pathway (Reaction): blue boxes are hyperlinked to REACTION entries that are selected from the original version.
- Organism-specific pathway: green boxes are hyperlinked to GENES entries by converting K numbers (KO identifiers) to gene identifiers in the reference pathway, indicating the presence of genes in the genome and also the completeness of the pathway.


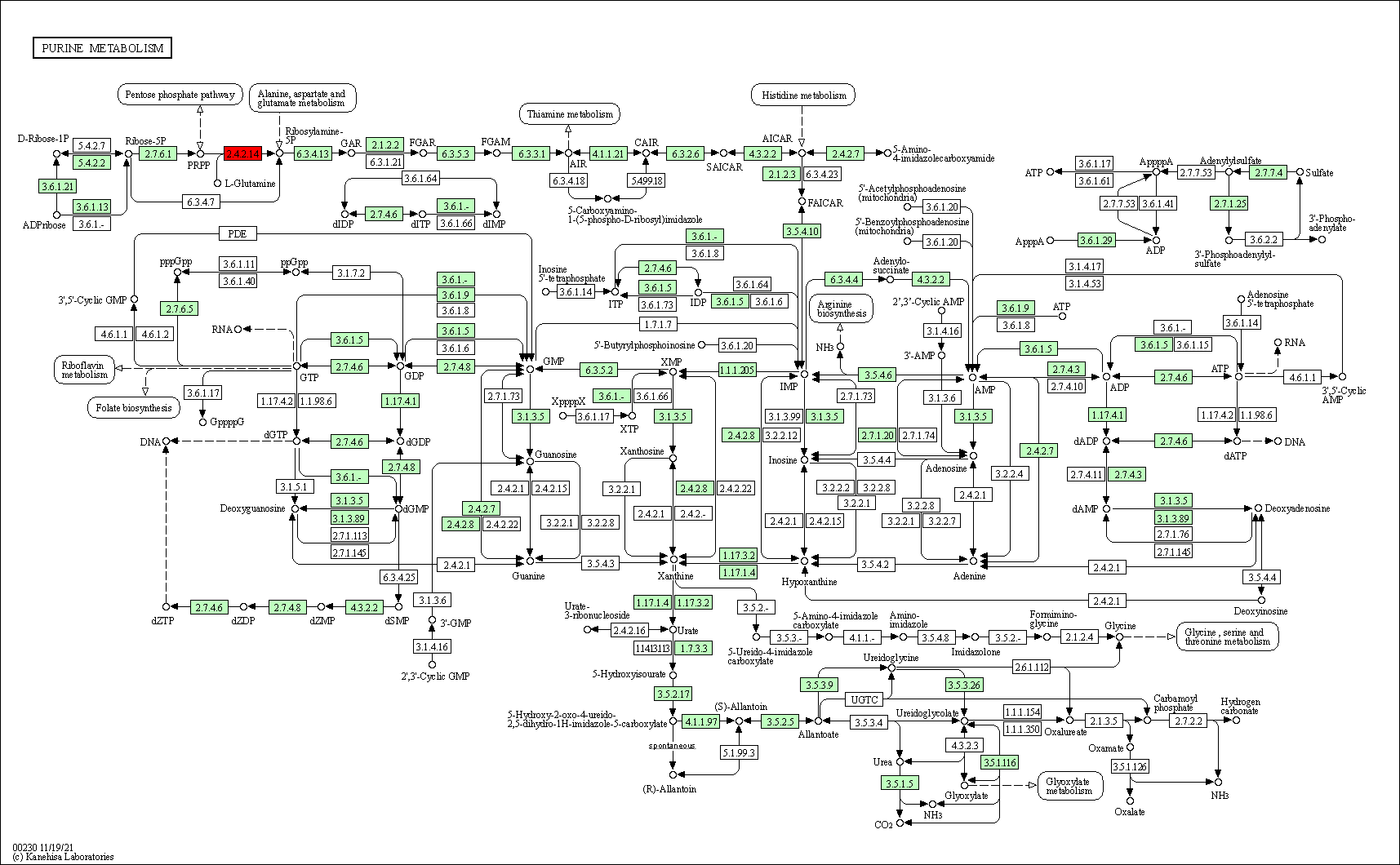


**Supplementary Fig 3.** The KEGG pathway of purine metabolism.

- Reference pathway: this is the original version; white boxes are hyperlinked to KO, ENZYME, and REACTION entries in metabolic pathways; they are hyperlinked to KO entries in non-metabolic pathways.
- Reference pathway (KO): blue boxes are hyperlinked to KO entries that are selected from the original version.
- Reference pathway (EC): blue boxes are hyperlinked to ENZYME entries that are selected from the original version.
- Reference pathway (Reaction): blue boxes are hyperlinked to REACTION entries that are selected from the original version.
- Organism-specific pathway: green boxes are hyperlinked to GENES entries by converting K numbers (KO identifiers) to gene identifiers in the reference pathway, indicating the presence of genes in the genome and also the completeness of the pathway.


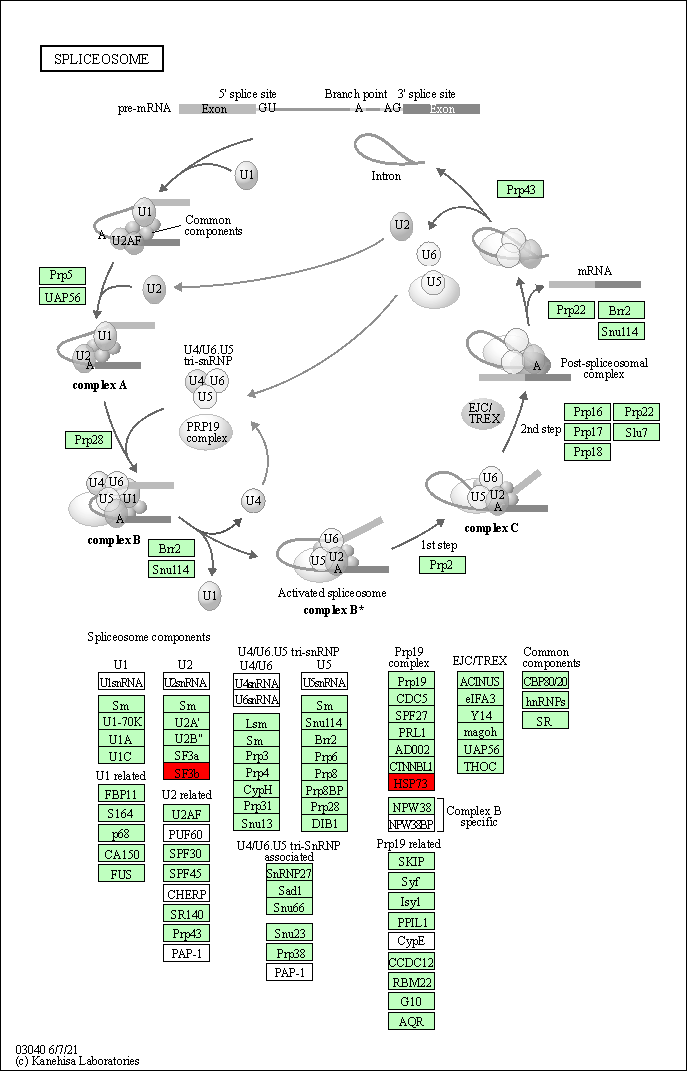


**Supplementary Fig 4.** The KEGG pathway of spliceosome.

- Reference pathway: this is the original version; white boxes are hyperlinked to KO, ENZYME, and REACTION entries in metabolic pathways; they are hyperlinked to KO entries in non-metabolic pathways.
- Reference pathway (KO): blue boxes are hyperlinked to KO entries that are selected from the original version.
- Reference pathway (EC): blue boxes are hyperlinked to ENZYME entries that are selected from the original version.
- Reference pathway (Reaction): blue boxes are hyperlinked to REACTION entries that are selected from the original version.
- Organism-specific pathway: green boxes are hyperlinked to GENES entries by converting K numbers (KO identifiers) to gene identifiers in the reference pathway, indicating the presence of genes in the genome and also the completeness of the pathway.


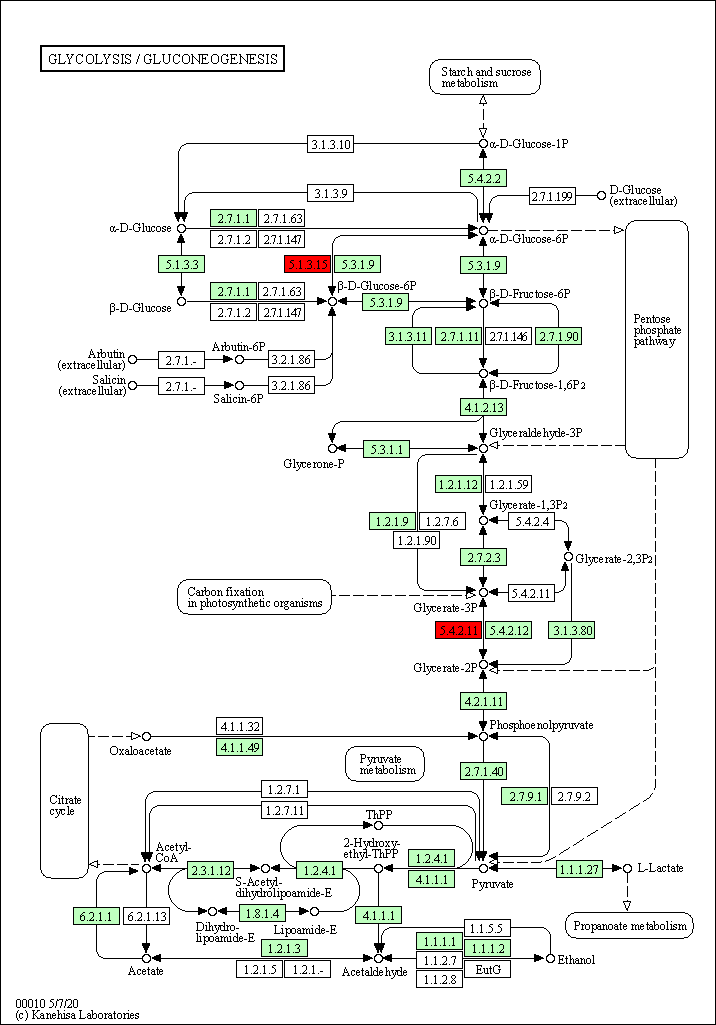


**Supplementary Fig 5.** The KEGG pathway of glycolysis/gluconeogenesis.

- Reference pathway: this is the original version; white boxes are hyperlinked to KO, ENZYME, and REACTION entries in metabolic pathways; they are hyperlinked to KO entries in non-metabolic pathways.
- Reference pathway (KO): blue boxes are hyperlinked to KO entries that are selected from the original version.
- Reference pathway (EC): blue boxes are hyperlinked to ENZYME entries that are selected from the original version.
- Reference pathway (Reaction): blue boxes are hyperlinked to REACTION entries that are selected from the original version.
- Organism-specific pathway: green boxes are hyperlinked to GENES entries by converting K numbers (KO identifiers) to gene identifiers in the reference pathway, indicating the presence of genes in the genome and also the completeness of the pathway.
